# Taxonomy, morphology, masticatory function and phylogeny of heterodontosaurid dinosaurs

**DOI:** 10.3897/zookeys.223.2840

**Published:** 2012-10-03

**Authors:** Paul C. Sereno

**Affiliations:** 1 Department of Organismal Biology and Anatomy and Committee on Evolutionary Biology, University of Chicago, Chicago, Illinois, USA

**Keywords:** Dinosauria, Heterodontosauridae, Heterodontosaurinae, *Heterodontosaurus*, tooth replacement, tooth wear, herbivory

## Abstract

Heterodontosaurids comprise an important early radiation of small-bodied herbivores that persisted for approximately 100 My from Late Triassic to Early Cretaceous time. Review of available fossils unequivocally establishes *Echinodon* as a very small-bodied, late-surviving northern heterodontosaurid similar to the other northern genera *Fruitadens* and *Tianyulong*. *Tianyulong* from northern China has unusual skeletal proportions, including a relatively large skull, short forelimb, and long manual digit II. The southern African heterodontosaurid genus *Lycorhinus* is established as valid, and a new taxon from the same formation is named *Pegomastax africanus* **gen. n., sp. n.** Tooth replacement and tooth-to-tooth wear is more common than previously thought among heterodontosaurids, and in *Heterodontosaurus* the angle of tooth-to-tooth shear is shown to increase markedly during maturation. Long-axis rotation of the lower jaw during occlusion is identified here as the most likely functional mechanism underlying marked tooth wear in mature specimens of *Heterodontosaurus*. Extensive tooth wear and other evidence suggests that all heterodontosaurids were predominantly or exclusively herbivores. Basal genera such as *Echinodon*, *Fruitadens* and *Tianyulong* with primitive, subtriangular crowns currently are known only from northern landmasses. All other genera except the enigmatic *Pisanosaurus* have deeper crown proportions and currently are known only from southern landmasses.

## Introduction

**Figure 1.**
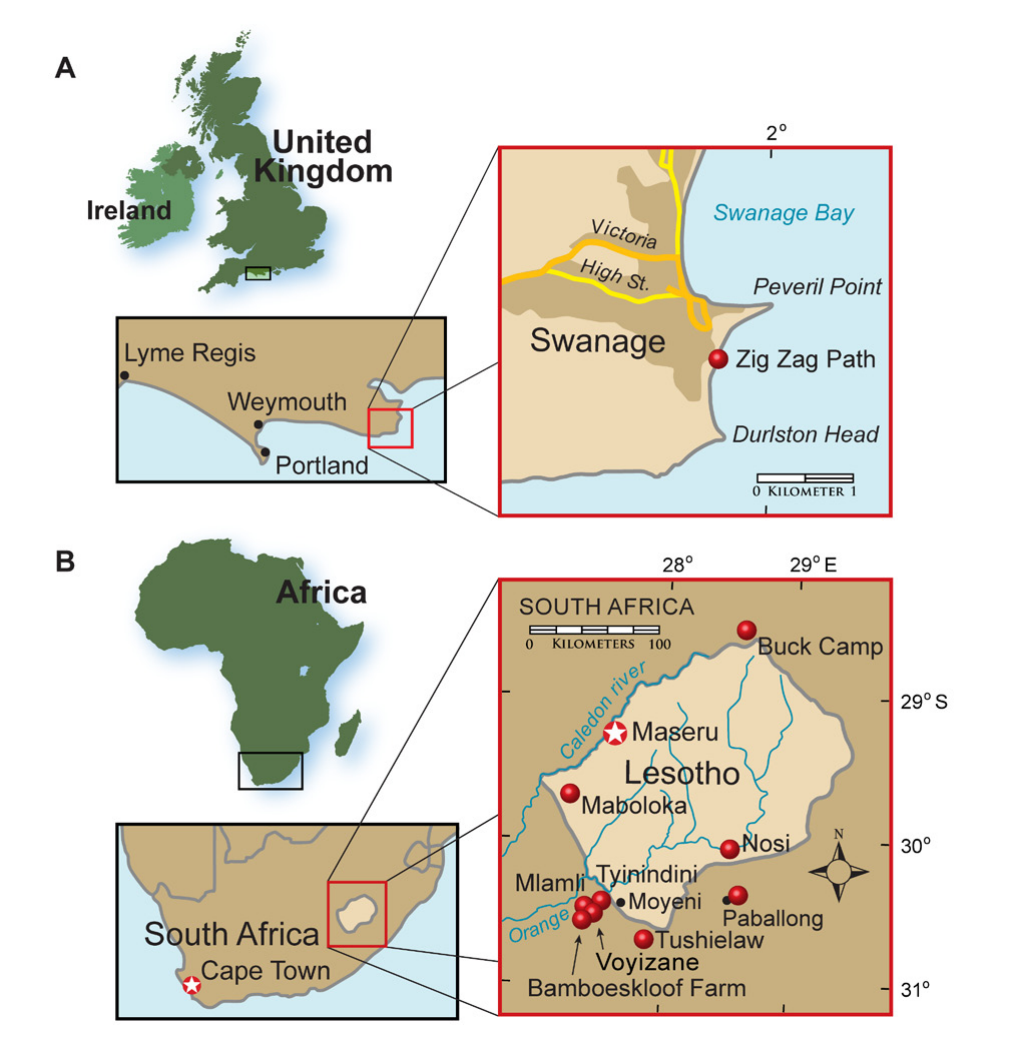
Heterodontosaurid localities. **A** Locality (red dot) for *Echinodon becklesii* on the southern coast of England **B** Heterodontosaurid localities in South Africa and Lesotho. Locality(ies)/taxon identification: Nosi/*Abrictosaurus consors*; Mlamli, Tushielaw, Tyinindini/*Heterodontosaurus tucki*; Bamboeskloof Farm, Buck Camp, Paballong/*Lycorhinus angustidens*; Maboloka/Heterodontosauridae incertae sedis; Voyizane/ *Pegomastax africanus* gen. n. sp. n.

In 1861 Owen described diminutive heterodontosaurid jaws a few centimeters in length as *Echinodon*, one of the first dinosaurs ever named. Initially thought to pertain to an extinct lizard, these specimens were discovered in outcrops on the southern coast of England (Fig. 1A). Despite considerable prospecting along the coast near the site of the initial find, no additional remains of this taxon have been unearthed, and its status as a heterodontosaurid was not recognized until recently (Sereno 1997; Norman and Barrett 2002).

Current knowledge of the morphology of heterodontosaurids is based largely on the South African genus *Heterodontosaurus* (Crompton and Charig 1962), a complete skull and nearly complete postcranial skeleton of which were discovered in 1966 (SAM-PK-K1332; Charig and Crompton 1974; Santa Luca et al. 1976; Santa Luca 1980; Weishampel 1984; Norman et al. 2011). The majority of heterodontosaurid specimens have been discovered in southern Africa (Fig. 1B), but their taxonomic status has remained unclear for several reasons. Some, such as *Geranosaurus*, were described on the basis of fragmentary, non-diagnostic jaw material (Broom 1911; Norman et al. 2011). Others, such as specimen NHMUK RU A100, were described with important bones misidentified (Thulborn 1970). Many specimens collected were not described or assessed until recently (Porro et al. 2011; Norman et al. 2011). And finally, the taxonomic basis of heterodontosaurid genera and species has been obscured by the inclusion of primitive character states in differential diagnoses rather than a shorter list of putative autapomorphies alone (Sereno 1990).

Heterodontosaurid remains have been found in recent years in continental areas other than southern Africa or the southern coast of England, including Argentina (Bonaparte 1976; Pol et al. 2011), western North America (Callison and Quimby 1984; Sereno 1986; Butler et al. 2010, 2012), and China (Zheng et al. 2009). These specimens have greatly broadened the taxonomic, morphological, temporal and paleobiogeographic range for heterodontosaurids (Table 1).

Despite their critical role in early dinosaur evolution as the most diverse subclade of ornithischians (Sereno 1999; Butler et al. 2007), heterodontosaurids have never been subject to a comprehensive taxonomic review. *Heterodontosaurus tucki* (Crompton and Charig 1962), which came to light in 1961, exactly one century after Owen’s report on *Echinodon*, has long functioned as the sole or primary reference point for heterodontosaurids (Santa Luca 1980; Norman et al. 2011), despite the fact that it remains one of the most derived members of the group. The diversity of form and function among these early small-bodied herbivores is only now gaining attention.

In this paper I attempt to clarify the generic and specific taxonomy of heterodontosaurids and important aspects of their dental, cranial and postcranial morphology. Then I address heterodontosaurid body size, skeletal proportions, tooth replacement, tooth wear, and jaw mechanics. Finally, I present new character data bearing on heterodontosaurid phylogenetic and paleobiogeographic history.

## Institutional and collections abbreviations

**AMNH** American Museum of Natural History, New York, New York, USA

**BP **Bernard Price Institute for Palaeontological Research, Johannesburg, South Africa

**CPBA** Cátedra de Paleontología de la Facultad de Ciencias Exactas de la Universidad de Buenos Aires, Buenos Aires, Argentina

**DORCM** Dorset County Museum, Dorchester, United Kingdom

**IVPP** Institute of Vertebrate Paleontology and Paleoanthropology, Beijing, PRC

**LACM **Natural History Museum of Los Angeles County, Los Angeles, California, USA

**MCZ** Museum of Comparative Zoology, Harvard University, Cambridge, Massachussets, USA

**MNA** Museum of Northern Arizona, Flagstaff, Arizona, USA

**MNBH** Musée national Boubou Hama, Niamey, République du Niger

**MPEF** Museo Paleontológico Egidio Feruglio, Trelew, Argentina

**NHMUK** Natural History Museum, London, United Kingdom

**NM** National Museum, Bloemfontein, South Africa

**PVL **Instituto Miguel Lillo, Tucumán, Argentina

**SAM** Iziko South African Museum, Cape Town, South Africa

**STMN **Shandong Tianyu Museum of Nature, Pingyi, Shandong Province, PRC

**UCRC** University of Chicago Research Collection, Chicago, Illinois, USA

## Heterodontosaurid fossil record

### Early heterodontosaurid discoveries

**Figure 2.**
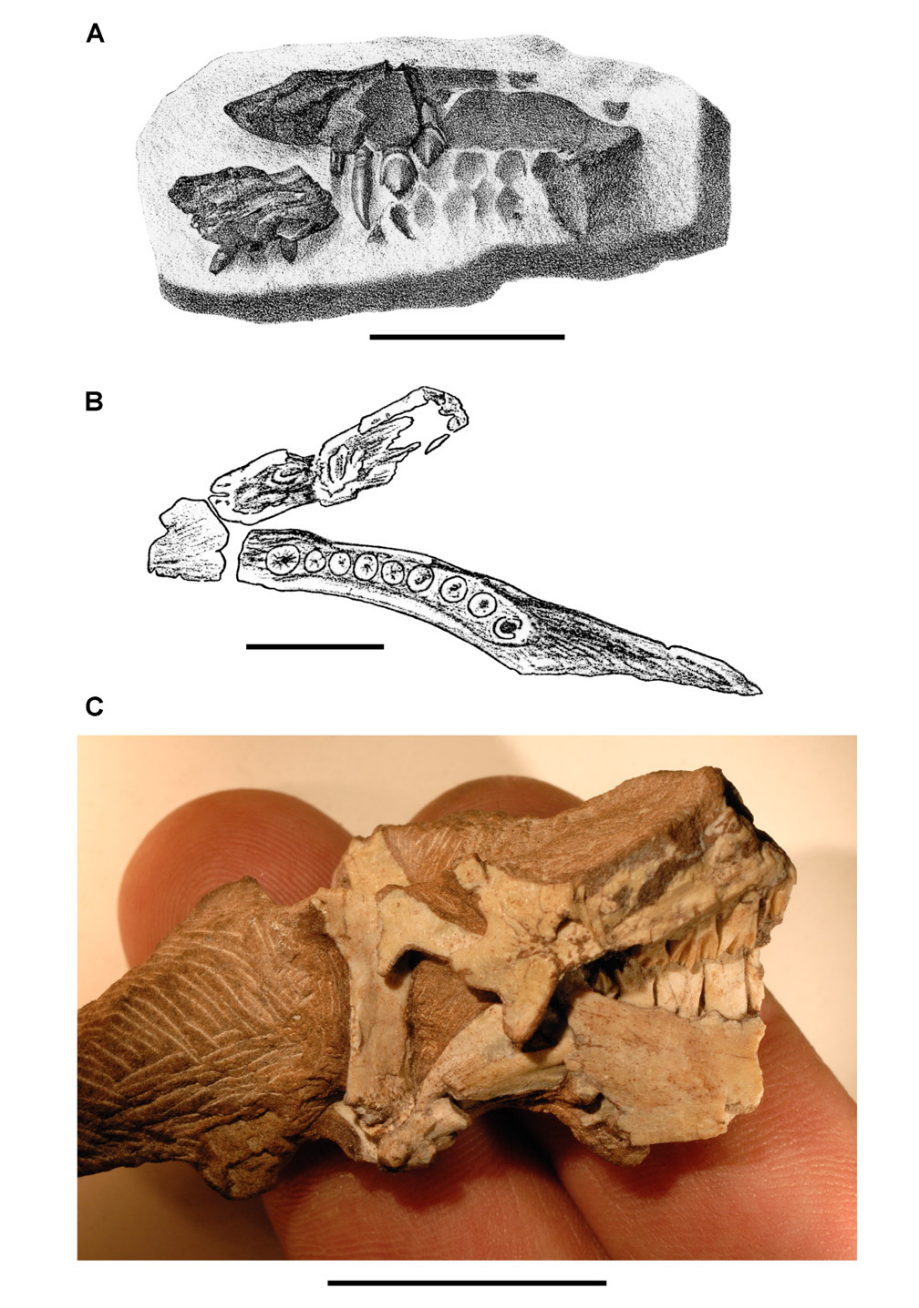
Early heterodontosaurid discoveries. **A** Lithographic drawing of the right and left premaxillae and the anterior portion of the left maxilla in lateral view of *Echinodon becklesii* (NHMUK 48209; from Owen 1861) **B** Drawing of lower jaws in dorsal view of *Geranosaurus atavus* (SAM-PK-K1871; from Broom 1911) **C** Photograph of the posterior portion of a subadult skull in right lateral view of *Heterodontosaurus tucki* (AMNH 24000). Scale bars equal 1 cm in **A** and 2 cm in **B** and **C**.

***Echinodon*.** Owen (1861) described *Echinodon becklesii* based on a series of fragmentary jawbones from a quarry in strata of the Lower Cretaceous (Berriasian) Purbeck Limestone Formation on the southern coast of England (Fig. 2A; Table 1). Although he placed *Echinodon becklesii* within Lacertilia in his original account, Owen underscored similarities in the dentition to that of *Scelidosaurus*, one of the few ornithischian dinosaurs described at that time. He also cited *Echinodon* as the “Purbeck dinosaur” in later work (Norman and Barrett 2002). Owen’s detailed lithographs provide important evidence in the evaluation of *Echinodon*, as some damage and loss have occurred in the ensuing 150 years.

**Table 1.** Specimens currently known for established heterodontosaurid species. Asterisks indicate holotypic, lectotypic and paralectotyopic specimens. Localities in England and southern Africa are shown in Figure 1. Erroneous spellings for some of the localities in southern Africa are given in parentheses.

**Taxon**	**Specimen**	**Locality**	**Brief Description**
*Abrictosaurus consors*	*NHMUK RU B54	Nosi (“Noosi“)	Skull and partial skeleton
	NHMUK no number	“ “	Partial fragmentary skeleton
*Echinodon becklesii*	*NHMUK 48209, 48210	Mammal Pit	Partial skull (lectotypes)
	*NHMUK 48211	“ “	Right maxilla
	*NHMUK 48212	“ “	Right maxilla
	*NHMUK 48213	“ “	Left dentary
	*NHMUK 48214	“ “	Right edentulous dentary
	*NHMUK 48215a	“ “	Right dentary
	*NHMUK 48215b	“ “	Left dentary
	NHMUK 48229	“ “	Jaw fragment
	NHMUK 40723	“ “	Dentary fragment
	DORCM GS 1164-5, 1167, 1171	Lovell’s Quarry	Isolated teeth
	DORCM GS 1194, 1212-6, 1222-3	Sunnydown Farm	Isolated teeth
*Fruitadens haagarorum*	*LACM 115747	Fruita Paleontological Area	Partial jaws and postcranial skeleton
	LACM 115727	“ “	Partial postcranial skeleton
	LACM 120478	“ “	Partial fore- and hind limbs of a subadult
	LACM 120602	“ “	Distal caudal vertebrae, distal limb bone
	LACM 128258	“ “	Premaxilla, maxilla, dentaries and vertebrae of a subadult
	LACM 128303	“ “	Partial left dentary
*Heterodontosaurus tucki*	*SAM-PK-K337	Tyinindini (“Tyindini“);	Adult skull and partial skeleton
	SAM-PK-K1332	Voisana	Adult skull and nearly complete skeleton
	SAM-PK-K10487	“ “	Anterior portion of juvenile skull
	SAM-PK-K1334	“ “	Partial left maxilla
	SAM-PK-K1326	“ “	Partial maxilla
	SAM-PK-K1328	southern Africa	Vertebrae, partial pelvic girdle and parts of forelimb and hind limb;
	NM QR 1788	Tushielaw	Fragmentary snout from an adult
	AMNH 24000	southern Africa	Posterior portion of juvenile skull
			
*Lycorhinus angustidens*	*SAM-PK-K3606	Paballong	Partial left dentary (now a natural mold)
	*UCRC PVC10	*—*	Silicone cast from natural mold of holotypic specimen
	NHMUK RU A100	Paballong	Partial disarticulated skull
	BP/1/4244	Buck Camp	Maxilla
	BP/1/5253	Bamboeskloof Farm	Maxilla
*Manidens condorensis*	MPEF-PV 3211	Queso Rallado	Partial disarticulated skull and skeleton lacking limbs
	MPEF-PV 1718, 1719, 1786, 3810, 3811;	“ “	Isolated teeth
*Pegomastax africanus* gen. n. sp. n.	SAM-PK-K10488	Voisana	Postorbital, right and left dentaries, and predentary
*Pisanosaurus mertii*	*PVL 2577	Agua de Las Catas	Right maxilla and dentary, a few vertebrae, an impression of the central portion of the right pelvic girdle, and partial right hind limb
*Tianyulong confuciusi*	*STMN 26-3	Liaoning	Partial articulated skeleton with skull
	IVPP V17090	“ “	Partial articulated skeleton with skull

Several of the dentaries preserve a large alveolus for a lower caniniform tooth (Sereno 1997), one of several heterodontosaurid features overlooked by Owen and subsequent descriptive accounts (Galton 1978; Norman and Barrett 2002). As described in more detail below, the postcaniniform cheek teeth are low crowned and exhibit extensive wear facets from tooth-to-tooth shearing. Although long underappreciated, the heterodontosaurid *Echinodon* was among the first dinosaurs ever discovered and remains one of the smallest ornithischians on record.

***Geranosaurus*.** Broom (1911) described a partial left maxilla and lower jaws including a partial predentary as the ornithischian *Geranosaurus atavus* (Fig. 2B). The specimen (SAM-PK-K1871) comes fromthe Lower Jurassic Clarens Formation (formerly Cave Sandstone) (Kitching and Raath 1984; Smith 1990; Knoll 2005). The left dentary preserves the roots of an anterior caniniform tooth and eight postcaniniform teeth. Some partial maxillary crowns, which Broom (1911: 307) described as “flat chisel-shaped” with the “outer face feebly ridged”, were originally present (Norman et al. 2011: fig. 37A). Many years ago, however, all of the partial crowns in the maxilla were lost (Hopson 1980: 94). Fragmentary vertebrae and hindlimb bones (now cataloged as SAM-PK-K1857) were found nearby, although Broom (1911: 306) doubted their association with the holotype. There does not appear to be any basis for referral of these fragmentary postcranial bones to *Geranosaurus atavus* (*contra* Porro et al. 2011: Table 1).

**Figure 3.**
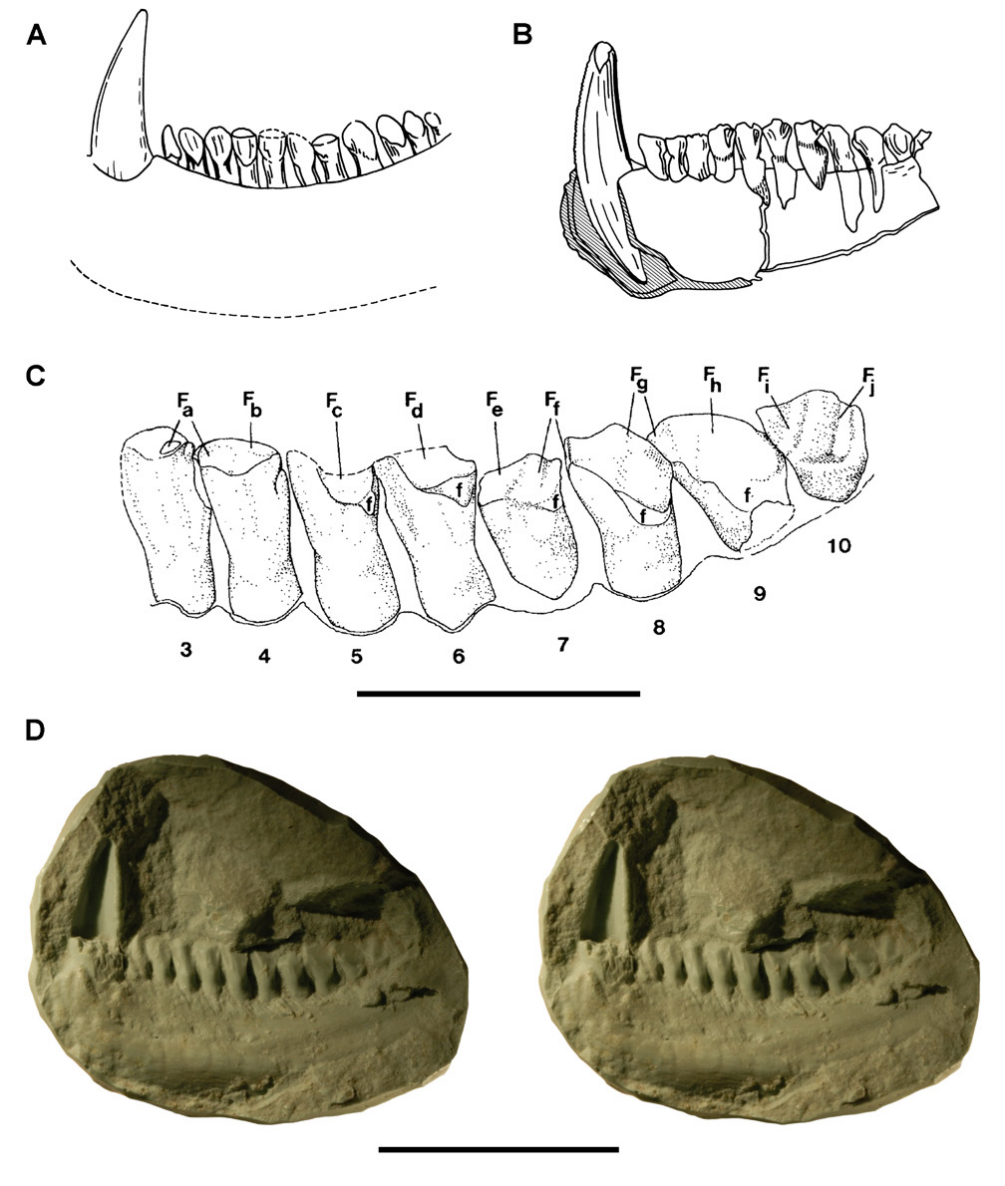
Early heterodontosaurid discoveries from southern Africa. **A** Drawing of the left dentary of the holotype of *Lycorhinus angustidens* (SAM-PK-K3606) in lateral view (from Haughton 1924) **B** Drawing of the left dentary of the holotype of *Lycorhinus angustidens* (SAM-PK-K3606) in medial view (reversed from Haughton 1924) **C** Left dentary teeth 3-10 in lateral view based on a natural cast (UCRC PVC10) of the holotype of *Lycorhinus angustidens* (from Hopson 1980) **D** Stereopair of a natural silicone cast of the holotype of *Lycorhinus angustidens* (UCRC PVC10). Abbreviations: ***3-10*** dentary tooth 3-10 ***F_a-j_*** tooth-to-tooth wear facet a-j ***f*** accessory facet. Scale bars equal 1 cm in **C** and 3 cm in **D**.

The damaged holotype and only known specimen of *Geranosaurus atavus* offers scant morphological evidence to distinguish the species. Thulborn (1974: 160) regarded *Geranosaurus atavus* as a *nomen dubium*, the material of which was “probably ornithischian”. Hopson (1980) also regarded *Geranosaurus atavus* as a *nomen dubium* but went farther to identify the jaw fragments as heterodontosaurid, an opinion supported here. The wedge-shaped predentary, transversely thick (rather than spout-shaped) anterior end of the dentary, enlarged anterior dentary tooth, and absence of active tooth replacement suggest that the holotypic material of *Geranosaurus atavus* is a heterodontosaurid, and as such stands as the first specimen pertaining to this group to be described from southern Africa.

*Geranosaurus atavus*, nonetheless, differs in several regards from *Heterodontosaurus tucki*, which also occurs in the Clarens Formation, and from heterodontosaurids from the underlying Upper Elliot Formation. The dentary tooth row in *Geranosaurus atavus* appears to be composed of eight subequal postcaniniform teeth arranged along a medially bowed tooth row (Fig. 2B). In *Heterodontosaurus*, in contrast,there are 11 or 12 dentary teeth that increase in size toward the center of a relatively straight tooth row. *Abrictosaurus* also has a higher tooth count and size differential along the dentary tooth row, as well as a relatively smaller caniniform tooth. Unlike *Heterodontosaurus* and *Lycorhinus*, a postcaniniform diastema is not present in *Geranosaurus atavus*, as the second tooth positioned adjacent to the caniniform tooth (Fig. 2B). Crompton and Charig (1962) cited the absence of an arched premaxilla-maxillary diastema in *Geranosaurus atavus* to differentiate *Geranosaurus atavus*, although this portion of the upper jaw does not appear to have been preserved.

Recently Norman et al. (2011) concluded that *Geranosaurus atavus* cannot be distinguished by a “unique combination of characters”. Although not adequate to justify taxonomic recognition, the features exhibited by *Geranosaurus atavus* and discussed above clearly suggest there is a second heterodontosaurid taxon in the Clarens Formation in addition to *Heterodontosaurus tucki*.

***Heterodontosaurus*.** Sometime prior to 1913, Broom discovered an important heterodontosaurid specimen probably somewhere in the Clarens Formation (Fig. 2C). It consists of the posteroventral portion of a subadult skull and is referred below to *Heterodontosaurus tucki* (AMNH 24000) (Table 1). Although this was the first specimen of *Heterodontosaurus tucki* collected, it was not identified until recently. The specimen was sold to the American Museum of Natural History in 1913 as part of the Broom Collection, which consisted almost entirely of synapsids (Broom 1915). The author discovered the specimen embedded in a small block of matrix among Broom’s synapsid specimens. The oversight was understandable as only a few of the left maxillary teeth were exposed in cross-section. Subsequent preparation exposed the posteroventral portion of an intact subadult skull, which is approximately one-half the size of the well preserved adult skull of *Heterodontosaurus tucki* (SAM-PK-K1332). The subadult skull preserves many diagnostic features of *Heterodontosaurus tucki* and provides important new information on tooth replacement and wear. The ventral portion of several cervical vertebrae are preserved in articulation posterior to the skull, suggesting that the preserved skull block may have originally been associated with at least a partial skeleton.

***Lycorhinus*.** Haughton (1924) described a left dentary with teeth as *Lycorhinus angustidens*, which he misidentified as a carnivorous therapsid (Fig. 3A, B). The specimen came from Paballong in the Upper Elliot Formation (formerly part of Red Beds) (SAM-PK-K3606; Table 1). Nearly forty years would pass before *Lycorhinus angustidens* was re-identified as a heterodontosaurid, after a reasonably complete skull of *Heterodontosaurus tucki* was discovered (Crompton and Charig 1962). By that time, only the caniniform crown remained of the holotypic specimen (Broom 1932; Hopson 1975: fig. 2; Hopson 1980: fig. 3; Gow 1990: fig. 7). As a result, taxonomic assessment of the original material of *Lycorhinus angustidens* (Hopson 1975, 1980; Fig. 3C) has been based on Haughton’s figures of the holotype and a silicone cast taken from the natural mold (UCRC PVC10; Fig. 3D). One aim of the present study is to resolve the taxonomic status of *Lycorhinus angustidens*, which has been subject to several conflicting interpretations (Table 2).

**Table 2. T2:** Published taxonomic opinion regarding heterodontosaurids from southern Africa. Authors included in the table have made specific taxonomic inferences on the basis of available material.

**Author(s)**	**Taxonomy**
Thulborn (1970, 1974)	*Lycorhinus* (= *Heterodontosaurus*); *Lycorhinus angustidens* (includes NHMUK RU A100); *Lycorhinus consors*; *Lycorhinus tucki*
Charig and Crompton (1974)	“*Lycorhinus angustidens*”; BMNH A100 (indeterminate); *Heterodontosaurus tucki*
Hopson (1975)	*Lycorhinus angustidens; Abrictosaurus consors* (includes NHMUK RU A100)*; Heterodontosaurus tucki*
Gow (1990)	*Lycorhinus angustidens*; (= *Lanasaurus scalpridens*; includes NHMUK RU A100)
Norman et al. (2011)	*Lycorhinus angustidens*; *Abrictosaurus consors*; *Lanasaurus scalpridens* (includes NHMUK RU A100)*; Heterodontosaurus tucki*
this paper	*Lycorhinus angustidens*; (= *Lanasaurus scalpridens*; includes NHMUK RU A100); *Abrictosaurus consors*; *Heterodontosaurus tucki*; *Pegomastax africanus* gen n. sp. n.

## More recent finds from southern locales

**Geological setting.** Africa and South America have both yielded important heterodontosaurid remains since the 1950s. African heterodontosaurids come from formations collectively known as the “Stormberg Group,” which straddles the Triassic-Jurassic boundary. Pollen, footprints and overlying lavas suggest that the “Stormberg Group” was deposited from the latest Triassic (Norian-Rhaetian) to the earliest Jurassic (Hettangian-Sinemurian) or approximately 210-197 Ma (Bristow and Saggerson 1983; Olsen and Sues 1986; Smith 1990; Knoll 2005; Gradstein and Ogg 2009). Various names have been coined for subunits within the “Stormberg Group,” which can be divided into Lower Elliot, Upper Elliot and Clarens formations (following Knoll 2005). Heterodontosaurids are known from the upper two formations, their distribution described briefly here.

African heterodontosaurids (*Abrictosaurus*, *Lycorhinus*, *Heterodontosaurus*, *Pegomastax* gen. n. sp. n.) are known mainly from the predominantly red fluvial-aeolian Upper Elliot Formation (formerly part of Red Beds), the age of which is regarded as earliest Jurassic (202-197 Ma, Hettangian) (Knoll 2005; Gradstein and Ogg 2009). The Upper Elliot Formation corresponds to the “*Massospondylus* Range Zone” of Kitching and Raath (1984), characterized by “smaller light-limbed forms” living under drier conditions (Smith 1990: 131) as compared to the heavier-bodied saurischians in the “*Euskelosaurus* Range Zone” of the Upper Triassic Lower Elliot Formation.

*Heterodontosaurus* and the fragmentary *Geranosaurus* were collected in lower levels of the overlying predominantly cream-colored, playa-aeolian Clarens Formation (formerly Cave Sandstone), the age of which is regarded as Early Jurassic (ca. 195 Ma, Sinemurian) (Knoll 2005; Gradstein and Ogg 2009). Unlike the diverse fauna from the well exposed Upper Elliot Formation, the cliff-forming Clarens Formation is less accessible and less fossiliferous. Recent identification of material as *Lycorhinus* sp. (Porro et al. 2011) from the Clarens Formation is poorly established.

South American heterodontosaurids are known from two formations in Argentina. The fluvial-overbank sequences in the fossiliferous Ischigualasto Formation in San Juan and La Rioja Provinces (Currie et al. 2009) are Late Triassic (late Carnian-early Norian) in age and have yielded the fragmentary skeleton of *Pisanosaurus mertii* (Casamiquela 1967; Bonaparte 1976). Unlike the other dinosaurs in the formation (*Herrerasaurus*, *Sanjuansaurus*, *Panphagia*, *Eoraptor*, *Eodromaeus*), which all come from fossiliferous lower members of Carnian age (ca. 230 Ma) (Martinez et al. 2011) in San Juan Province, *Pisanosaurus* was discovered farther to the northwest in La Rioja in less fossiliferous beds higher in the section in the Ichigualasto Formation and possibly of early Norian age (ca. 225 Ma; Martinez et al. 2009). In 1991 an Argentine-American team co-led by the author revisited the type locality (Agua de Las Catas), which was not particularly fossiliferous and did not yield additional dinosaurian remains.

The Middle Jurassic Cañadón Asfalto Formation of Chubut Province in Patagonia recently has yielded a partial skeleton and isolated teeth of the heterodontosaurid *Manidens* (Pol et al. 2011). Although current specimens do not include limb bones, more material may be forthcoming from a formation that has yielded thus far the most diverse Middle Jurassic vertebrate fauna from any southern continent (Escapa et al. 2008).

**Figure 4.**
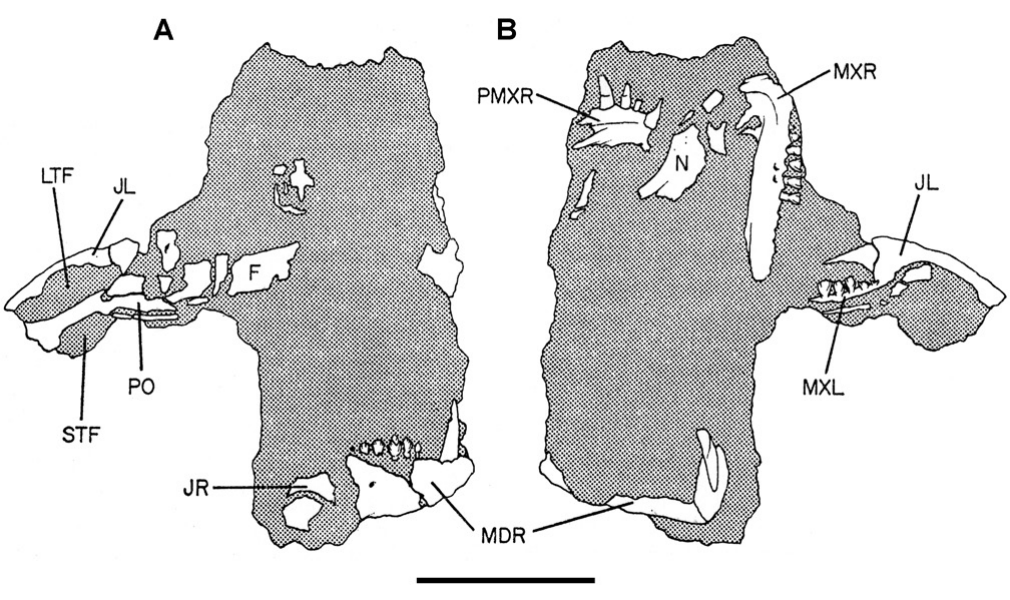
Controversial early heterodontosaurid specimen from southern Africa. Drawing of specimen NHMUK RU A100 as embedded in matrix in **A** top and **B** bottom views (from Thulborn 1970). Abbreviations: ***F*** frontal ***JL*** left jugal ***JR*** right jugal ***LTF*** left lateral temporal opening ***MDR*** right mandible ***MXL*** left maxilla ***MXR*** right maxilla ***N*** left nasal ***PMXR*** right premaxilla ***PO*** postorbital ***STF*** supratemporal fossa. Scale bar equals 5 cm.

***Lycorhinus*.** In 1960-1961 an expedition from University College London (Kermack 1962) discovered a partially disarticulated skull in the Transkei (Herschel) District of the South Africa in the vicinity of the type locality of *Lycorhinus angustidens* (Haughton 1924). The specimen, now catalogued as NHMUK RU A100 (Fig. 4), was initially referred to *Lycorhinus angustidens* (Thulborn 1970). Later authors have regarded the specimen either as taxonomically indeterminate (Charig and Crompton 1974; Porro et al. 2011), referable to *Abrictosaurus consors* (Hopson 1975), or referable to the *Lanasaurus scalpridens* (Norman et al. 2011) (Table 2). New information on the specimen provided later in this study supports referral to *Lycorhinus angustidens* as originally proposed by Thulborn.

Two additional maxillae with worn teeth were collected by C. Gow and J. Kitching from the Transkei (Herschel) District of South Africa. The first (BP/1/4244) was discovered in the early 1970s at Buck Camp and originally described as *Lanasaurus scalpridens* (Gow 1975; Hopson 1980), and the second (BP/1/5253) was found in 1984 at Bamboeskloof Farm (Gow 1990; Fig. 1B; Table 1). Both are referable to *Lycorhinus angustidens* as discussed below, *Lanasaurus scalpridens* isregarded here as a junior synonym. Two weathered specimens from Maboloka in the Clarens Formation (NHMUK RU C68, C69; Fig. 1B) were recently described as “*Lycorhinus* sp.” (Porro et al. 2011), a poorly established referral based on material regarded here as Heterodontosauridae *incertae sedis*.

***Heterodontosaurus*.** In 1961–1962 a joint expedition between the South African Museum and British Museum discovered the holotypic skull and partial skeleton of *Heterodontosaurus tucki* in the Transkei (Herschel) District of South Africa at a locality called Tyinindini just south of Lesotho (Crompton and Charig 1962; Fig. 1B; Table 1). A manuscript on the skull was drafted by Crompton and Charig and recently revised, greatly expanded, and published as a monograph on the skull of this taxon (Norman et al. 2011). The hematite-covered skull is fairly complete, although damaged by application of a diamond saw during its preparation (SAM-PK-K337; Norman et al. 2011: Figs 1–3, Appendix 3). The postcranial remains originally associated with the holotypic skull (Crompton and Charig 1962: 1076, 1077) were never described or figured and appear to have been lost (Norman et al. 2011: 189).

In 1966-1967 an expedition composed of members from four institutions (South African Museum, British Museum, Yale University, University College London) returned to this area and discovered a nearly complete skull and skeleton of *Heterodontosaurus tucki* (SAM-PK-K1332) in the Upper Elliot Formation (formerly Upper Red Beds) in the Transkei (Herschel) District of South Africa at a locality known as Voyizane (= Voisana) (Crompton 1968; Santa Luca et al. 1976; Norman et al. 2011; Fig. 1B; Table 1). Initial publications on this very complete individual included skull and dental reconstructions (Charig and Crompton 1974; Weishampel 1984; Crompton and Attridge 1986) and a description of the postcranium (Santa Luca et al. 1976; Santa Luca 1980). A detailed descriptive account of the skull has recently been published (Norman et al. 2011).

Four additional specimens referable to *Heterodontosaurus tucki* were collected during the 1966-1967 expedition not far from skeleton SAM-PK-K1332 at the locality Voyizane (= Voisana). These include the anterior portion of a juvenile skull (SAM-PK-K10487; Butler et al. 2008), a fragmentary maxilla (SAM-PK-K1326), a portion of the left maxilla with teeth from an adult skull (SAM-PK-K1334; Norman et al. 2011: Figs 30–33), and adult postcrania including vertebrae, a partial pelvic girdle, and parts of fore and hindlimbs (SAM-PK-K1328). In 1975, a fifth specimen consisting of a partial snout of a large individual (NM QR 1788) was discovered on Tushielaw Farm, located approximately 60 kms east and slightly south of Voyizane (Fig. 1B; Table 1). As reported by Porro et al. (2011), until recently the specimen was identified as the sauropodomorph *Massospondylus* in the collections of the National Museum.

**Figure 5.**
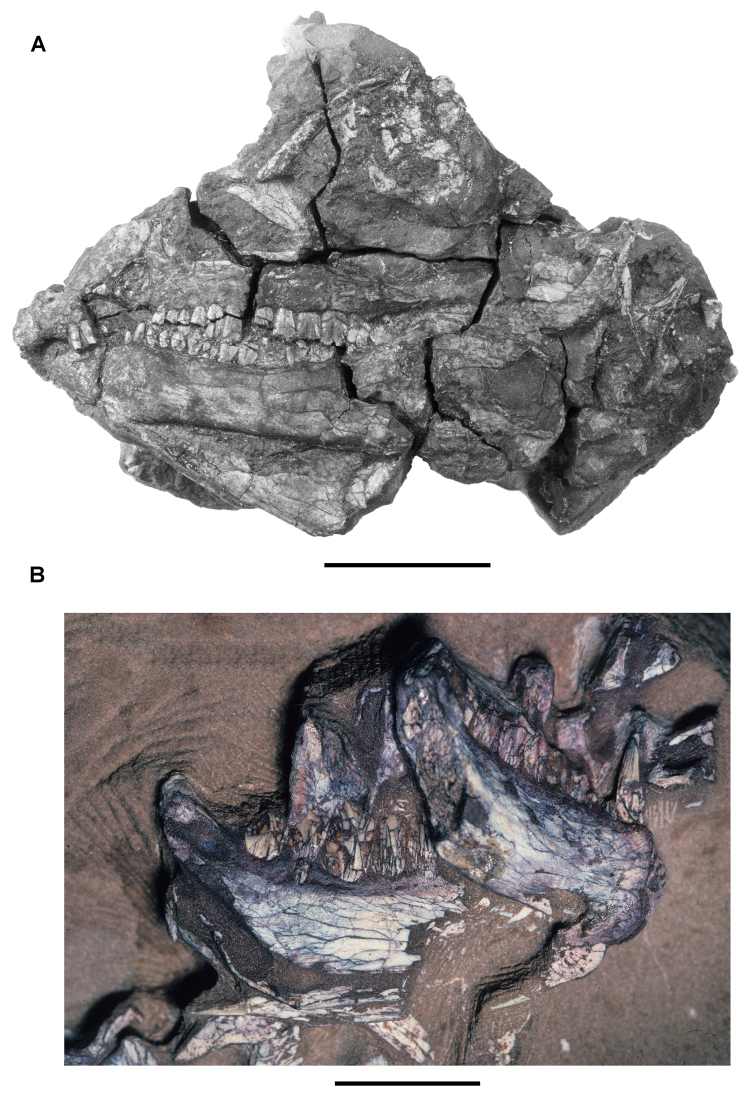
More recent heterodontosaurid discoveries from southern Africa. **A** Partial skull of *Abrictosaurus consors* in left lateral view (NHMUK RU B54) **B** Lower jaws of *Pegomastax africanus* gen. n. sp. n. (SAM-PK-K10488) in right ventrolateral view. Scale bars equal 2 cm in **A** and 1 cm in **B**.

***Abrictosaurus*.** In 1963–1964 K. Kermack and F. Mussett of University College London collected an articulated skull and skeleton (now catalogued as NHMUK RU B54) in the Upper Elliot Formation at the locality Nosi (= “Noosi”) in southern Lesotho (formerly Basutoland) (Figs 1B, 5A; Table 1). The specimen was originally referred to the genus *Lycorhinus* as a new species *Lycorhinus consors* (Thulborn 1974). Shortly thereafter Hopson (1975) transferred the holotype and only known specimen to a new genus as *Abrictosaurus consors* (Table 2).

**Elliot heterodontosaurid.** The 1966-1967 expedition to the Transkei (Herschel) District of South Africa (Crompton 1968) collected a partial disarticulated skull of a new heterodontosaurid (SAM-PK-K10488) from the locality Voyizane in the Upper Elliot Formation (Figs 1B, 5B; Table 1). Prepared at Harvard University and recognized by the author as a distinctive taxon in the 1980s, the specimen was recently listed as a heterodontosaurid of uncertain affinity (Porro et al. 2011). The specimen preserves the postorbital, dentaries, predentary and lower dentition and is described below as *Pegomastax africanus* gen. n. sp. n.

**Figure 6.**
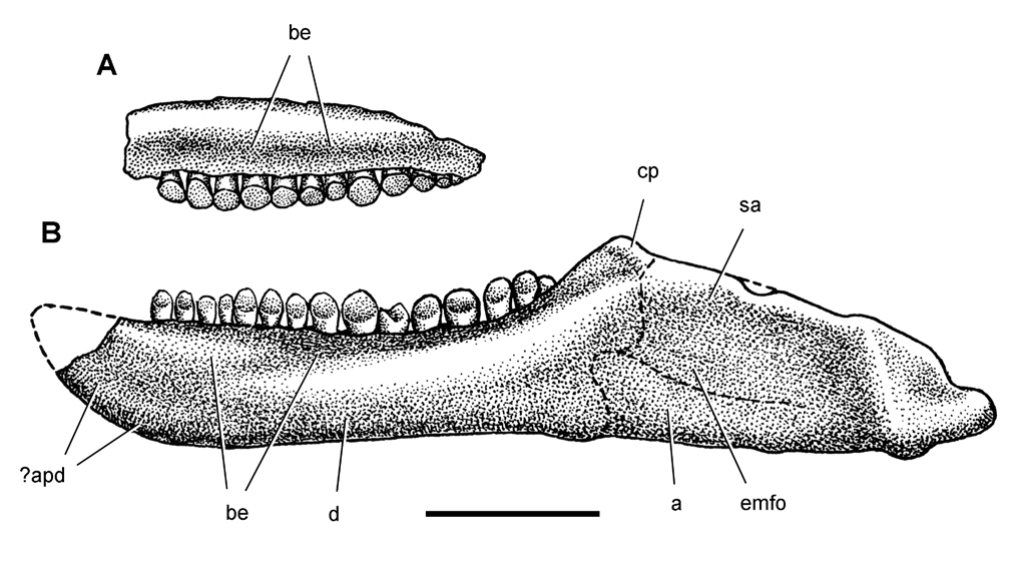
Cranial remains of *Pisanosaurus mertii* from the Upper Triassic Ischigualasto Formation of Argentina. Drawings of a partial right maxilla in medial view (**A**) and right lower jaw (reversed) in lateral view (**B**) (from Bonaparte 1976). Dashed lines indicate estimated sutures and edges. Scale bar equals 2 cm in **B**. Abbreviations: ***a*** angular ***apd*** articular surface for the predentary ***be*** buccal emargination ***cp*** coronoid process ***d*** dentary ***emfo*** external mandibular fossa ***sa*** surangular.

**Figure 7.**
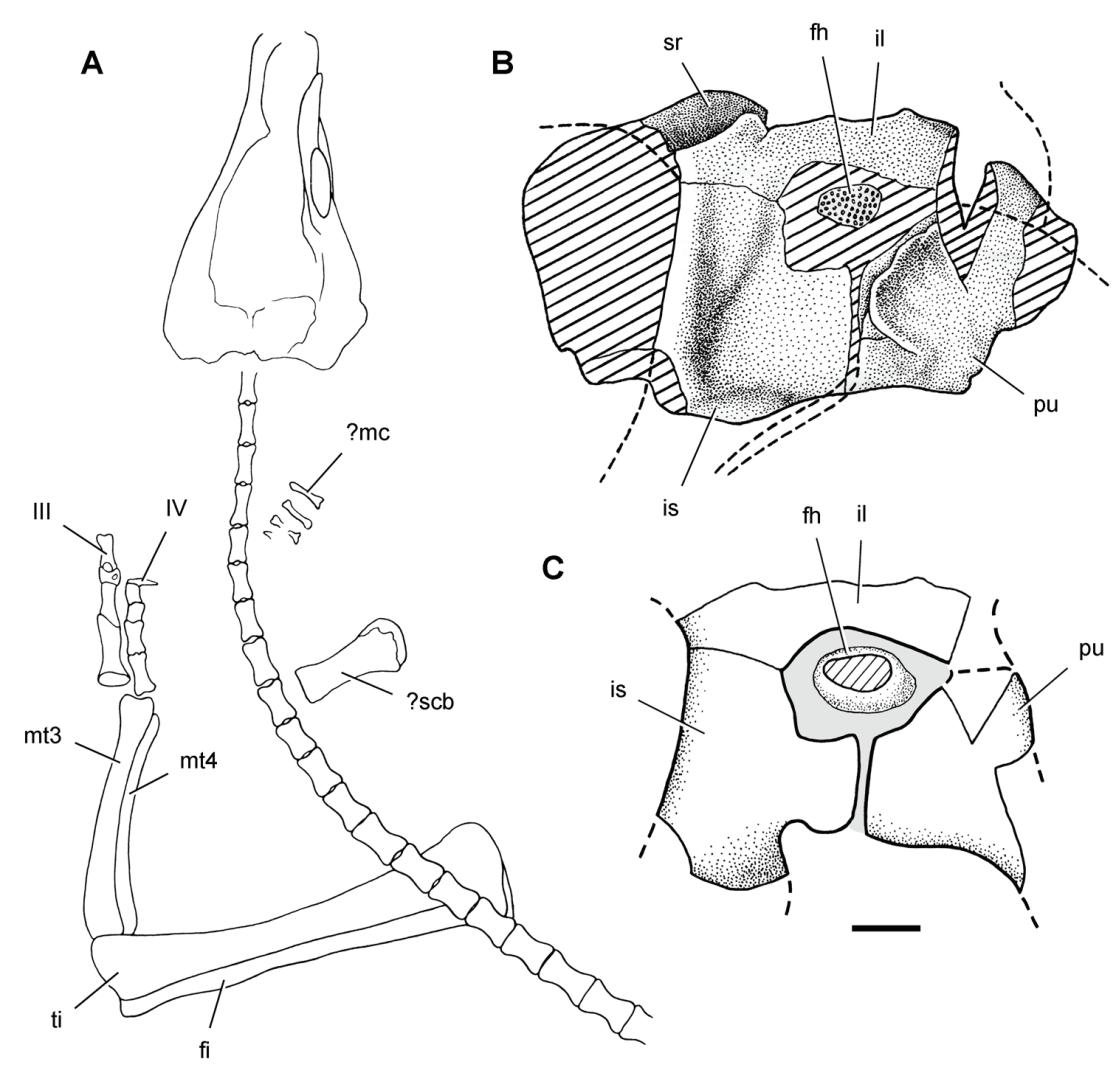
Postcranial remains of *Pisanosaurus mertii* from the Upper Triassic Ischigualasto Formation of Argentina. Drawings of the holotype (PVL 2577) in the field (from Bonaparte 1976) (**A**) and impression of central portion of the right pelvic girdle and head of the femur in medial view as previously interpreted (from Bonaparte 1976) (**B**) and in this study (**C**). Dashed lines indicate estimated edges; hatching in **B **indicates broken bone and matrix; hatching in **C** indicates broken bone; tone in **C** indicates matrix. Scale bar for **B** and **C** equals 1 cm. Abbreviations: ***III***,***IV*** pedal digit III, IV ***fh*** femur head ***fi*** fibula ***il*** ilium ***is*** ischium ***mc*** metacarpal ***mt3***,***4*** metatarsal 3, 4 ***pu*** pubis ***scb*** scapular blade ***sr*** sacral rib ***ti*** tibia.

***Pisanosaurus*.** *Pisanosaurus mertii* was found in the Upper Triassic (Carnian-Norian) Ischigualasto Formation of northwest Argentina (Casamiquela 1967; Martinez et al. 2011) and may represent the earliest heterodontosaurid on record (Bonaparte 1976; Sereno 1991). The holotype and only known specimen consists of a fragmentary skeleton including partial upper and lower jaws, a series of seven articulated dorsal vertebrae, and an articulated partial hindlimb including the tibia, fibula, proximal tarsals, and pedal digits III and IV (Figs 6, 7). Of less certain association are a few fragmentary vertebrae initially identified as caudal vertebrae (Casamiquela 1967; as cervical vertebrae by Bonaparte 1976), the impression of the central portion of the pelvis and femoral head (Fig. 7B, C), a small flat plate identified as a left scapular blade (Fig. 7A), and the partial impression of some small long bones identified as metacarpals (Fig. 7A).

Several of the bones originally part of the holotypic specimen were lost since their description by Casamiquela (1967). The impression of the anterior axial column and supposed partial metacarpals (Fig. 7A) were lost in part from the “corrosive effects of salt” (Bonaparte 1976: 809). The relative proportions of the cervicodorsal centra figured by Bonaparte (Fig. 2A) are interesting, as the posterior cervical centra are not reduced in relative length as occurs in other heterodontosaurids (e.g., *Heterodontosaurus*). Other bones lost since their original description include a small plate-shaped bone identified as a left scapular blade (Fig. 7A), a distal tarsal, and the proximal end of metatarsal 2 (Casamiquela 1967: pl. IV). As with the cervical centra, the broad proportions of the scapular blade and the short (shorter than cervical centrum length), subequal length of the supposed metacarpal impressions do not resemble the shape or relative size of comparable bones in several other heterodontosaurids.

Sereno (1991: 174) questioned their association, stating “the fragmentary scapula and other assorted postcrania are too small.” Irmis et al. (2007: 11) countered “There is no evidence to support claims that the holotype might be a chimaera of several individuals or taxa”, because no other vertebrates were described near the holotype and because the bones were similar in color and style of preservation. This is weak justification for the association of bones and impressions, now lost, which differ markedly from those in other heterodontosaurids. Furthermore, perusal of the collection reveals that other small, fragmentary postcranial bones were assigned to the same collection number (PVL 2577) but never described. This material includes a small partial femur with an aliform (not pendant) fourth trochanter and a small proximal tibia. Mixed and disarticulated vertebrate remains are commonplace in the Ischigualasto Formation. The partial articulation of the holotype and the presence of extraneous small postcranial bones under the same collection number cast doubt on the identity and association of the supposed girdle and forelimb elements in *Pisanosaurus mertii*.

Although Irmis et al. (2007) confirmed most of the additional descriptive detail on *Pisanosaurus* provided by Sereno (1991), they suggested that the external mandibular fenestra is closed. The area of the mandible in question was broken when initially described (Casamiquela 1967), although both Casamiquela and Bonaparte (1976) also suggested there was no fenestra in the sidewall of the mandible. Irmis et al. (2007) provided additional photographs of the jaw, but these were not accompanied by interpretive drawings showing broken edges or surfaces. The curved margin of a small external mandibular fenestra appears to be preserved in two short sections in the wall of the adductor fossa. The presence of a fenestra, thus, is possible, although better preservation is required to be certain. An external mandibular fenestra is present in some heterodontosaurids (e.g., *Heterodontosaurus*) and absent in others (e.g., *Manidens*).

Irmis et al. (2007) also suggested, contrary to Sereno (1991), that there is no trace of sacral ribs or centra that can be identified as pertaining to the sacrum in the block preserving pelvic fragments and impressions (Fig. 7B, C). The impression of the distal end of a sacral rib, however, is present near the ischial peduncle of the ilium. This rib impression projects away from the impression of a centrum that expands abruptly in transverse width near its anterior margin. The primordial pair of sacral centra in ornithischians is usually swollen anteriorly in this manner for articulation laterally with sacral ribs. This centrum impression and the succeeding (most posterior) centrum impression are aligned and adjacent, suggesting that at least one and more likely two, sacral centra are recorded on the pelvic block. No evidence exists, however, regarding central fusion or clues to the total number of sacral vertebrae (here defined as vertebrae in direct contact with the pelvic girdle).

The basal constriction between crown and root, the buccal emargination on the maxilla and dentary, and the coronoid process on the dentary suggest ornithischian affinity (Sereno 1999; Irmis et al. 2007). The relatively robust fibula has a minimum shaft diameter that is approximately 70% that of the tibia (Sereno 1991: fig. 14). This is a relatively more robust fibular shaft than is present in all other heterodontosaurids for which the fibula is known (*Tianyulong*, *Fruitadens*, Kayenta heterodontosaurid, *Abrictosaurus*, *Heterodontosaurus*). Derived features that suggest heterodontosaurid affinity include the absence of replacement foramina, a broad external mandibular fossa, and a transversely narrow, disc-shaped calcaneum. If the pelvis impression is properly associated, the acetabulum appears to be open as in heterodontosaurids rather than partially backed by a flange of the ilium as in *Lesothosaurus* and some other basal ornithischians (Sereno 1991). Other aspects of the pelvis, however, do not resemble heterodontosaurids, such as the broad puboischial contact under the acetabulum and the apparent absence of a postpubic process (Fig. 7C).

The relatively short crowns, limited variation in crown size, and well developed low-angle wear facets resemble the condition in the heterodontosaurid *Lycorhinus* (= *Lanasaurus*), although tooth wear is difficult to score as a character in phylogenetic analysis given variation within and among specimens. The dentary in *Pisanosaurus* is robust anteriorly as in heterodontosaurids rather than tapering, but its anterior end is broken away (Fig. 6B). The maxilla likewise is not complete anteriorly or posteriorly (Fig. 6A). Thus it is impossible to determine if there were caniniform upper or lower teeth, an arched upper diastema, or a wedge-shaped predentary. There does not appear to be any features that unambiguously link *Pisanosaurus* with more advanced neornithischians.

Based on the foregoing, *Pisanosaurus* appears to represent a basal ornithischian and possibly a basal heterodontosaurid. Although Irmis et al. (2007: 14) suggested that improved phylogenetic analysis with more dinosaurian outgroups and basal dinosaurs may yield “a robust phylogenetic hypothesis for the relationships of *Pisanosaurus*”, the surviving portions of the holotype are simply too incomplete to support an unambiguous phylogenetic interpretation. A more specific phylogenetic interpretation will require the discovery of additional specimens referable to *Pisanosaurus*.

**Figure 8.**
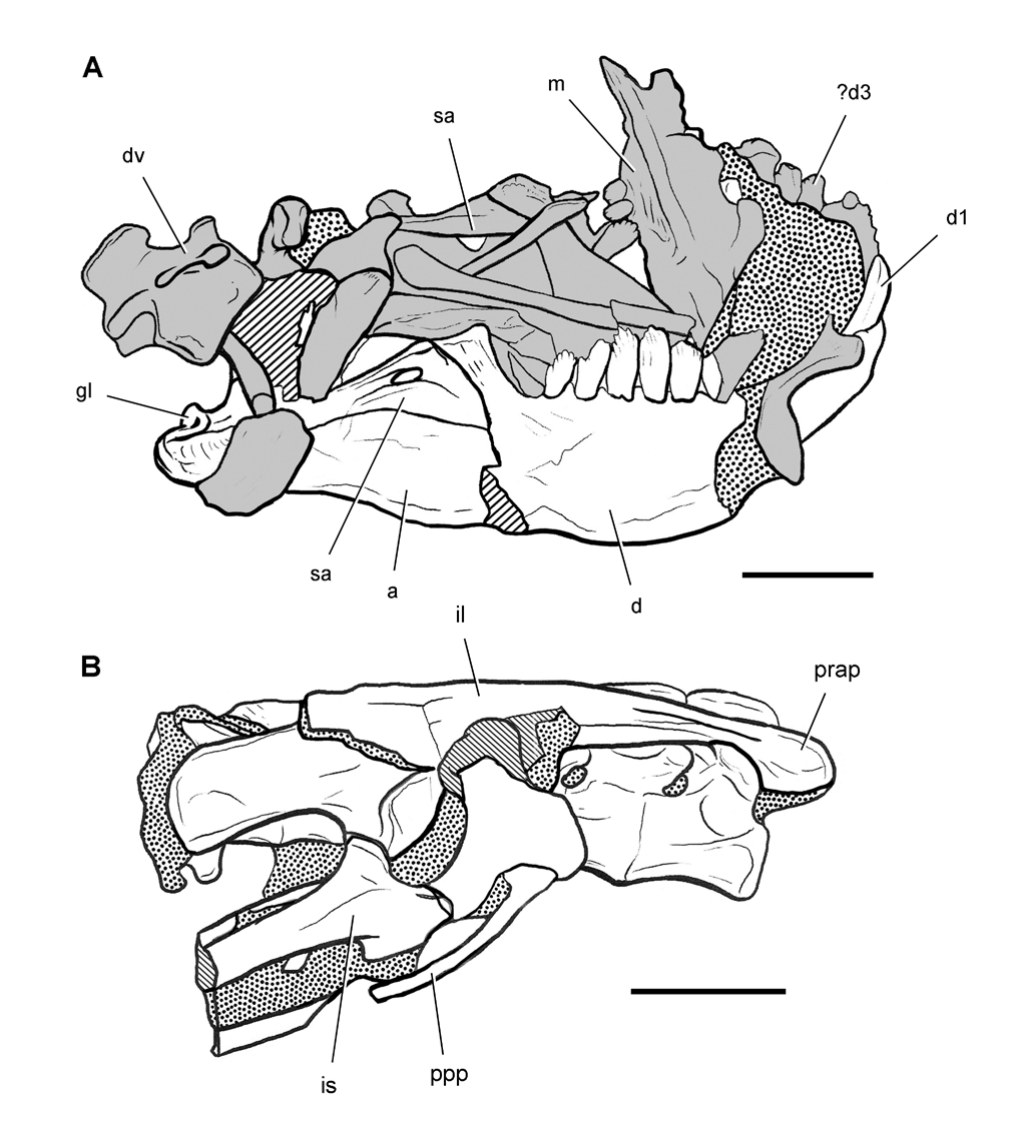
Skeletal remains of *Manidens condorensis* from the Middle Jurassic Cañadón Asfalto Formation of Chubut Province, Argentina. Drawings of major cranial (**A**) and postcranial (**B**) blocks (from Pol et al. 2011). Stipple indicates matrix; hatching indicates broken bone; grey tone in A shades the right maxilla, left lower jaw and other bones to highlight the right lower jaw. Scale bars equal 1 cm. Abbreviations: ***a*** angular ***d*** dentary ***d1***,***3*** dentary tooth 1, 3 ***dv*** dorsal vertebra ***gl*** glenoid ***il*** ilium ***is*** ischium ***m*** maxilla ***ppp*** postpubic process *prap* preacetabular process ***sa*** surangular.

***Manidens.**Manidens condorensis*, a small-bodied heterodontosaurid from the Middle Jurassic Cañadón Asfalto Formation in central Patagonia (Chubut Province) in Argentina (Pol et al. 2011), represents the first diagnostic ornithischian material recovered from Jurassic rocks of South America (Fig. 8). Previously known Jurassic ornithischian remains are limited to isolated teeth and bones pertaining to more advanced neornithischians from Venezuela (Barrett et al. 2008). Although lacking limbs, the partial holotypic skeleton of *Manidens* constitutes the most completely known southern heterodontosaurid exclusive of Africa. The dentary ramus has deep, short proportions, and the dentary crowns curve distally (Fig. 8A), features shared by a new heterodontosaurid (*Pegomastax africanus* gen. n. sp. n.) from southern Africa.

## More recent finds from northern locales

**Figure 9.**
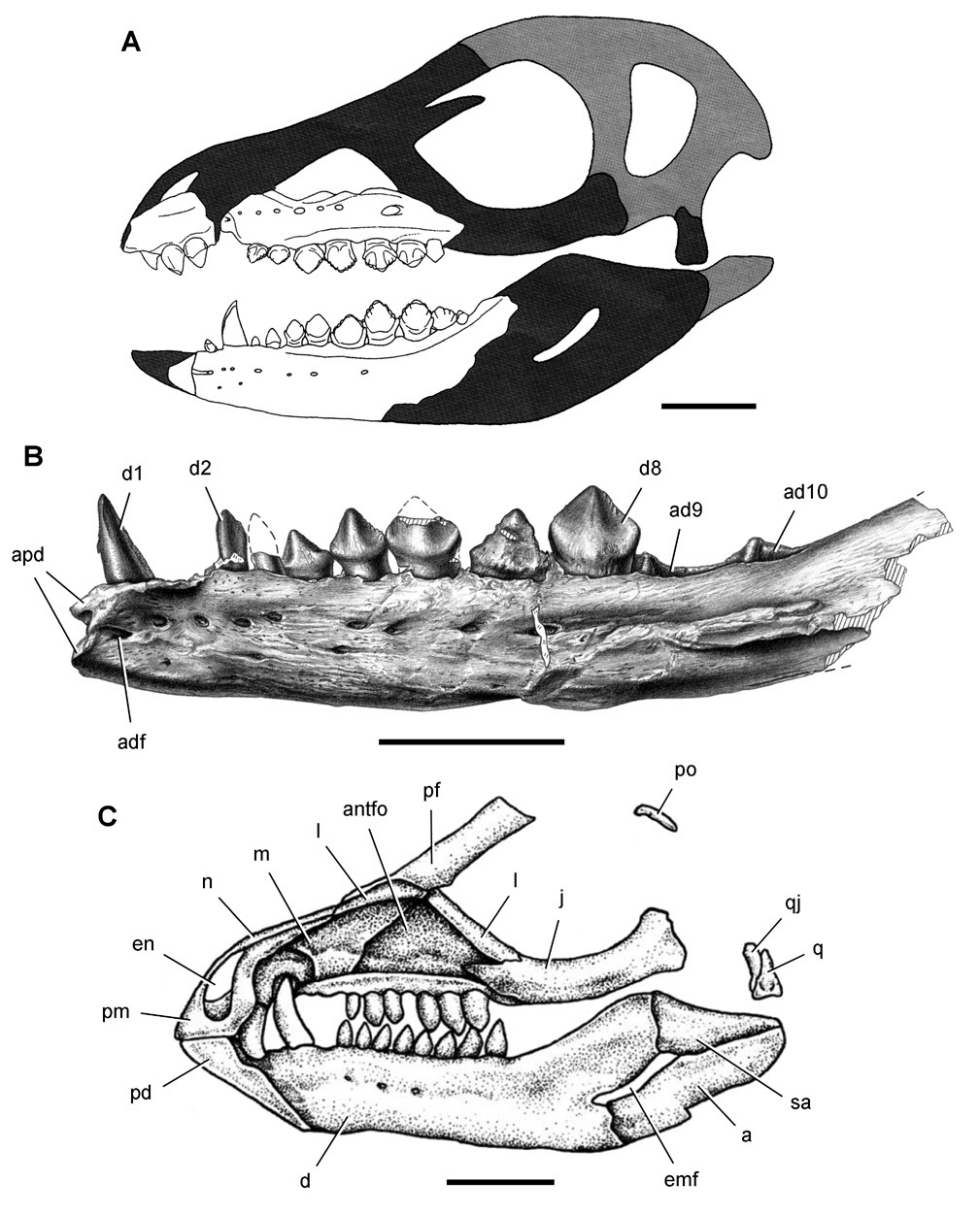
More recent heterodontosaurid discoveries from northern locales. **A** Jaws of *Fruitadens haagarorum* from the Upper Jurassic Morrison Formation in Colorado, USA (based on LACM 115747, 128258; reversed from Butler et al. 2010) **B** Left dentary in lateral view of an undescribed heterodontosaurid from the Lower Jurassic Kayenta Formation of Arizona (from Sereno et al. unpublished) **C** Partial skull of *Tianyulong confuciusi* from the Yixian Formation of Liaoning Province, PRC (STMN 26-3; reversed from Zheng et al. 2009). Abbreviations: ***a*** angular ***ad 9***,***10*** alveolus for dentary tooth 9, 10 ***adf***  anterior dentary foramen ***antfo*** antorbital fossa ***apd*** articular surface for the predentary ***d*** dentary ***d1***, ***2***, ***8*** dentary tooth 1, 2, 8 ***emf*** external mandibular fenestra ***en*** external nares ***j*** jugal ***l*** lacrimal ***m*** maxilla ***n*** nasal ***pd*** predentary ***pf*** prefrontal ***pm*** premaxilla ***po*** postorbital ***q*** quadrate ***qj*** quadratojugal ***sa*** surangular. Scale bar equals 1 cm in **A** and **C** and 5 mm in **B**.

**Geological setting.** With the exception of *Echinodon* (Owen 1861), discovery of heterodontosaurids from northern locales first began in western North America in the mid 1970s and early 1980s. Like *Echinodon* these heterodontosaurids rank among the smallest of ornithischian dinosaurs (Table 3). In 1975 a field party led by George Callison from California State University at Long Beach discovered disarticulated small vertebrate remains on a visit to the Upper Jurassic Morrison Formation in west-central Colorado near the town of Fruita. Over the next decade, a diverse microvertebrate fauna was recovered, which included the remains of a heterodontosaurid recently named *Fruitadens haagarorum* (Butler et al. 2010, 2012; Fig. 9A; Table 1). The specimens were preserved in crevasse splay deposits near the base of the Brushy Basin Member in the Upper Jurassic (Tithonian) Morrison Formation. The particular locality yielding the majority of the *Fruitadens* specimens, “Callison’s Main Quarry”, preserves a transported and mixed assemblage of microvertebrates buried in fluvial-paludal sediment (Kirkland et al. 2005; Kirkland 2006).

**Table 3.** Skull, axial, and long bone lengths (mm, above) and proportions (%, below) in the best known heterodontosaurids. Measurements average long bone lengths when both sides are available. Parentheses indicate estimated length or proportion.

		STMN 26-3*Tianyulong*	*Tianyulong* IVPP V17090	LACM 120478*Fruitadens*	LACM 115747*Fruitadens*	NHMUK 48215*Echinodon*	Kayenta taxon; MCZ9092	NHMUK RU A100*Lycorhinus*	MPEF-PV 3211*Manidens*	SAM-PK-K10488*Pegomastax*	NHMUK RU B54*Abrictosaurus*	*Heterodontosaurus*; SAM-PK-K1332
Length; (mm)	Skull^1^	(67)	65	(60)^6^	(75)^8^	(62)^9^	(53)^9^	(145)^9^	(71)^9^	(73)^9^	(82)	115^10^
	Humerus	33	(27)	37	(46)^8^		—		—	—	50	83
	Radius	—	17	—	—		—		—	—	(36)	58
	Metacarpal 3	—	5	—	—		—		—	—	15	22
	Femur	(54)^5^	51	(62)^7^	(78)^8^		—		—	—	78	112
	Tibiotarsus	82	73	74	(93)^8^		—		—	—	100	145
	Metatarsal 3	(44)	(43)	—	—		—		—	—	53	68
	Body length^2^	(450)	—	—	—		—		—	—	—	(1080)
	Neck & trunk (precaudal column)	(102)	—	—	—		—		—	—	—	(324)
	Caudal column	(296)	—	—	—		—		—	—	—	(659)
Proportion; (%)	Skull/body length	(12)	—	—	—		—		—	—	—	(9)
	Skull/femur	(125)	127	(97)	(97)		—		—	—	(105)	103
	Precaudal/body length	(30)	—	—	—		—		—	—	—	(23)
	Caudal/body length	(65)	—	—	—		—		—	—	—	(61)
	Humerus/forelimb^3^	—	53	—	—		—		—	—	(50)	51
Proportion; (%)	Radius/forelimb	—	36	—	—		—		—	—	(36)	36
	Metacarpal 3/forelimb	—	10	—	—		—		—	—	(15)	14
	Tibiotarsus/femur	152	143	(119)	(119)		—		—	—	128	130
	Femur/hind limb^4^	30	31	—	—		—		—	—	34	35
	Tibiotarsus/hind limb	46	44	—	—		—		—	—	43	45
	Metatarsal 3/hind limb	24	26	—	—		—		—	—	23	21
	Humerus/femur	61	53	(60)	(60)		—		—	—	64	74
	Forelimb/hind limb	—	29	—	—		—		—	—	(44)	50

^1^Skull length is measured or estimated from the tip of the premaxilla to the posterior edge of the squamosal.; ^2^Body length is composed of three successive lengths: functional skull length (measured from the premaxilla to the occipital condyle) + precaudal column length (as measured with natural curves) + caudal column.; ^3^Forelimb length equals the sum of humerus + radius + metacarpal 3.; ^4^Hind limb length equals the sum of femur + tibia + metatarsal 4.; ^5^The reported length of the left femur (51 mm) is more reliable than the considerably shorter length estimate (40 mm) for the incomplete right femur (Zheng et al. 2009). The left femur, in addition, is missing the proximal portion of the head (Zheng et al. 2009; Supplemental Information), and so the length estimate used here is 54 mm.; ^6^Based on the dentary of a subadult specimen (LACM 128258) with a length of approximately 24 mm (Butler et al. 2010: fig. 2d). The dentary is missing its anteriormost end and the coronoid process, which when restored yields a total length estimate of approximately 27 mm. The dentary in *Heterodontosaurus* is approximately 45% of total skull length, which suggests approximately 60 mm for total skull length in subadult *Fruitadens*.; ^7^Femur length was estimated by adding the preserved portion (42.2 mm; Butler et al. 2010: fig. 2j) to an estimate of the missing portion of the shaft proximal to the fourth trochanter, based on the femora of *Heterodontosaurus* (Santa Luca 1984: fig. 18B) and *Abrictosaurus* (NHMUK RU B54). The preserved portion of the femur in LACM 120478 is estimated to be 68% of femur length.; ^8^Skull and long bone estimated measurements for adult *Fruitadens* are based on two overlapping tibial dimensions (transverse width of proximal and distal ends, 79% and 81% of the adult), which suggest the subadult (LACM 120478) is approximately 80% the size of the adult (LACM 115747). All adult estimated measurements are scaled up accordingly from the subadult specimen, as there are no complete long bones known in adult material (LACM 115727, 115747).; ^9^Skull length estimated from dentary length (*Echinodon*, 28 mm; Kayenta taxon, ~24 mm; *Lycorhinus*, 65 mm; *Manidens*, ~32 mm; *Pegomastax*, ~33 mm), assuming that dentary length (dentary anterior extremity to tip of coronoid process) is approximately 45% of total skull length (tip of the premaxilla to the posterior edge of the squamosal) as in *Heterodontosaurus*.; ^10^The jaws of a large heterodontosaurid, tentatively referred to *Heterodontosaurus tucki* (NM QR 1788; Porro et al. 2011), are approximately 175% the size of SAM-PK- K1332, resulting in an estimated skull length of approximately 200 mm (the skull length estimate of 166 mm by Porro et al. 2010 is for a different measure of skull length).

Starting in the mid 1970’s, field parties led by Farish Jenkins, Jr. from Harvard University discovered a locality rich in microvertebrate remains about 50 kms southeast of Tuba City in north-central Arizona (Jenkins et al. 1983). In 1981 a very small, partially articulated ornithischian skeleton was recovered in the silty facies of the Lower Jurassic (Sinemurian-Pliensbachian) Kayenta Formation. The specimen was later identified as a new juvenile heterodontosaurid (Attridge et al. 1985; Sereno 1986, 1997; MCZ 9092; Fig. 9B), the first heterodontosaurid to be recognized from a Laurasian landmass (*contra* Butler et al. 2010: 375).

An exceptionally preserved, small heterodontosaurid named *Tianyulong confuciusi* with filamentous integumentary structures extending away form the skeleton has been discovered in northern China (Zheng et al. 2009; Fig. 9C; Table 1). Recently, the provenance of *Tianyulong* has been determined definitively to be an upper horizon of the Lanqi (= Tiaojishan) Formation in Liaoning Province radioisotopically dated to the latest Middle Jurassic (Callovian) approximately 160 Mya (Liu et al. 2012). The horizon yielding *Tianyulong* is approximately 100 m above and slightly younger than horizons yielding many skeletons of the feathered paravian *Anchiornis huxleyi* (Hu et al. 2009; Liu et al. 2012).

***Fruitadens*.** Initially cited as a small “fabrosaurid” ornithopod (Callison and Quimby 1984) and later as an unnamed species of *Echinodon* (Galton 2002, 2006), the four known specimens have been recently described as the heterodontosaurid *Fruitadens haagarorum* (Butler et al. 2010, 2012; Fig. 9A). Much of the holotypic specimen (LACM 115747; Table 1) was found in place and removed in a field jacket (G. Callison, pers. comm.). Three referred specimens were surface collected, one providing some evidence of limb proportions (LACM 120478; Table 1). The association of these specimens and their morphology and body size are discussed further below.

**Kayenta heterodontosaurid.** The specimen (MCZ 9092; Fig. 9B), originally reported by Attridge et al. (1985) and Sereno (1986, 1997), preserves complete upper and lower dentitions, many other portions of the skull, vertebrae from all portions of the axial column, and portions of fore and hind girdles and limbs (Attridge et al. 1985; Sereno 1986, 1997; MCZ 9092; Fig. 9B). The Kayenta heterodontosaurid, the most completely preserved heterodontosaurid from North America, will be described in detail elsewhere.

***Tianyulong*.** *Tianyulong confuciusi* was initially described on the basis of an articulated, ash-covered skeleton laying flat on a slab of lacustrine rock (Zheng et al. 2009; STMN 26-3; Fig. 9C; Table 1). The specimen is noteworthy not only because of the preservation of integumental structures external to the skeleton but also because of the low diversity of ornithischians within the Jehol fauna (Xu and Norell 2006). There are at least five additional partial skeletons of similar small body size in collections in China, one of which (IVPP V17090) is described in more detail below. The holotypic and referred skeletons are nearly identical in size (Table 3). Given the high level of articulation and skeletal fusion and the grossly similar size of all known specimens, these skeletons are probably representative of maximum adult body size.

### Doubtful heterodontosaurids

**Santa Cruz material.** A partial maxilla and an isolated caniniform crown were recently recovered from the Upper Triassic (Norian) Laguna Colorado Formation in southern Patagonia in Argentina and referred to *Heterodontosaurus* sp. (Báez and Marsicano 2001). The maxillary fragment (CPBA-V-14091a) contains four worn teeth of unusual form. The crowns are shaped as curved columns in mutual contact and truncated by low-angle, transversely cupped wear facets. In ventral view, the crowns are subrectangular and broader labiolingually than mesiodistally. Impressions of the labial side of some crowns suggest that the apical margin of the crown is denticulate. There is some indication that there is a buccal emargination on the maxilla and no evidence of replacement foramina or erupting replacement teeth. The caniniform tooth has serrations on mesial and distal edges and could belong to a heterodontosaurid.

In *Heterodontosaurus*, in contrast, the crowns are not in mutual contact except near the wear surface, the roots are not as curved in mesial or distal view, the crown cross-section is mesiodistally longer than wide labiolingually, and there is a prominent median ridge on labial and lingual crown surfaces. The angle of wear facets in *Heterodontosaurus*, as described below, varies from glancing to high-angle. In sum, the material may represent an ornithischian or even a heterodontosaurid, and its Late Triassic age places it among the oldest ornithischian specimens known. There is no justification at present, however, for referral of this material to the genus *Heterodontosaurus*.

**Yunnan material.** Fragmentary material from the Lower Jurassic Lufeng Formation in southern China (Yunnan Province) described as *Dianchungosaurus lufengensis* has sometimes been referred to the Heterodontosauridae (Weishampel and Witmer 1990). Re-examination of the holotype has shown it to be a chimera of basal crocodyliform and basal sauropodomorph material (Barrett and Xu 2005).

## Methods

**Preparation.** Fossil material was prepared using pin vice, pneumatic air scribe, and airbrasive techniques.

**Imaging.** Computed tomography was used to reveal internal details on a subadult skull of *Heterodontosaurus tucki* (AMNH 24000) and a worn maxillary tooth of the ornithopod *Ouranosaurus nigeriensis* (MNBH GAD28). The specimens were scanned at the High-Resolution X-ray Computed Tomography Facility at The University of Texas at Austin.

**Anatomical orientation.** The standard directional terms of comparative anatomy or “Romerian”terms are used over veterinarian alternatives for reasons outlined elsewhere (Wilson 2006). In reference to the skeleton, for example, *anterior* and *posterior* are employed rather than “rostral”, ”cranial” or “caudal.” In reference to the dentition, *mesial*, *distal*, *labial*, *lingual*, *basal*, and *apical* are used as directional terms rather than “anterior”, “posterior”, “lateral”, “medial”, “ventral”, and “dorsal”. The former terms, which are standard for mammalian dentitions, unambiguously describe features in teeth arranged along a curved dental arcade and can be applied to both upper and lower dentitions.

**Figure 10.**
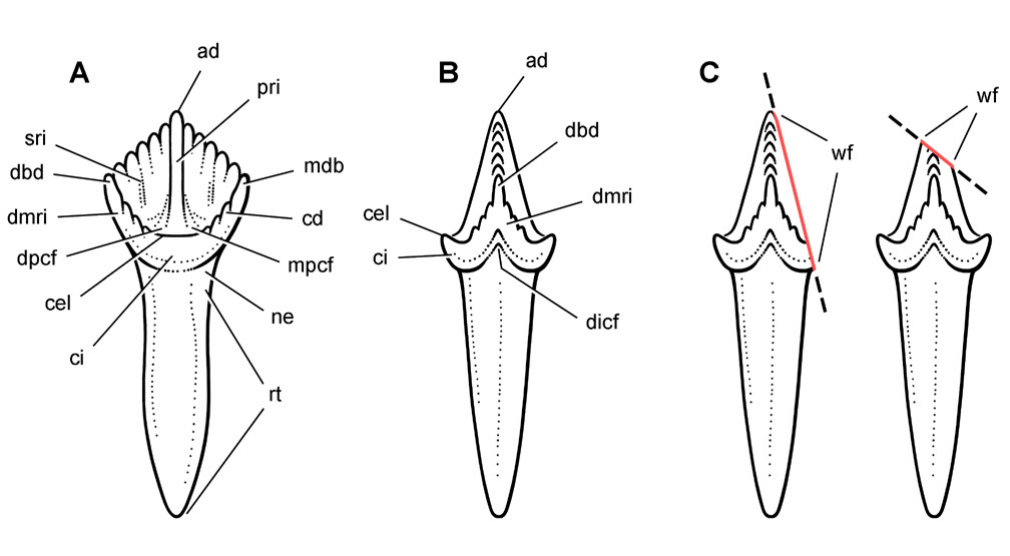
Cheek tooth terminology. **A** Postcaniniform maxillary or dentary tooth in labial or lingual view, respectively. **B** Postcaniniform maxillary or dentary tooth in distal view. **C** Pair of worn postcaniniform maxillary or dentary teeth in proximal or distal view showing low-angle (left) and high-angle (right) wear facets (red line). The angle of incidence (dashed line) of each wear facet (red line) is measured away from the vertical axis of the crown. Abbreviations: ***ad*** apical denticle ***cd*** cingular denticle ***cel*** cingular ectoloph ***ci*** cingulum ***dbd*** distal basal denticle ***dicf*** distal intercingular fossa ***dmri*** distal marginal ridge ***dpcf***  distal paracingular fossa ***mbd*** mesial basal denticle ***mpcf*** mesial paracingular fossa ***ne*** neck ***pri*** primary ridge ***rt*** root ***sri*** secondary ridge ***wf*** wear facet.

Wear facets tend to be approximately planar, and so the task at hand is to describe the angle of the plane of wear relative to a frame of reference. The terms *low-angle* and *high-angle* have been used as descriptors for the general orientation of wear facets. *Low-angle* and *high-angle*, of course, are measures relative to a particular axis or plane of reference. In this paper, wear facet angle is measured away from the *vertical*, whether the crown of a tooth or the skull is used as a frame of reference. The crown of a tooth is a useful frame of reference, allowing measurement of the angle of a wear facet in isolated jaws or individual teeth away from a vertical axis or plane through the apical margin of the crown (Fig. 10C). The crown is often less subject to distortion than the skull as a whole. On the other hand, sometimes the vertical axis or apical margin is difficult to establish because of the cover of matrix or wear. The skull is another useful frame of reference, especially if the aim is to evaluate occlusion or masticatory function.

For both of these frames of reference, *low-angle* wear facets refer to wear surfaces that glance the crown whereas *high-angle* wear facets truncate the crown—the former nearly parallel to the crown axis and the latter set at a greater angle from the apical plane of the crown (Fig. 10C). Hopson (1975, 1980) and Gow (1990) estimated the angle of wear facets in heterodontosaurids in this manner, measuring away from the vertical, the perceived dorsoventral axis of the crown. These studies described wear facets in isolated jaws, the exact orientation of which in an articulated skull remains unknown.

Norman et al. (2011), in contrast, use the terms *low-angle* and *high-angle* wear facets relative to the horizontal plane of the cranium, reversing the meaning of the terms as used above. In this work, a “low-angle” wear facet is one that is nearly horizontal, truncating the crown at a sharp angle, which is equivalent to “high-angle” as outlined above. No reasons were given for measuring the angle of wear facets away from a horizontal plane. A vertical axis or plane is preferable, as it is the main axis of the tooth and dominant direction of masticatory movement. For purposes of discussion, “low” and “high” angle wear facets are divided here about a 45° angle to a vertical axis; facets with an orientation from the vertical less than, or more than, a 45° are described as “low” and “high” angle wear facets, respectively.

**Anatomical terms.** For antorbital structures, I adopt and extend the terminology of Witmer (1997). As observed on the skull of *Heterodontosaurus* (Figs 59, 90), the large invaginated opening on the snout sidewall surrounding the antorbital fossa is identified as the *external antorbital fenestra*. The two fenestrae within the antorbital fossa include the *internal antorbital fenestra* (= “antorbital fenestra”, Norman et al. 2011: 204) and a more anteriorly positioned opening here termed the *accessory antorbital fenestra* (= “anterior maxillary fenestra”, Norman et al. 2011: 204). A blind recess within the antorbital fossa near its anterior corner is here identified as the *promaxillary fossa*.

Tooth identification uses a letter abbreviation for location in the dentary (d), premaxilla (pm), or maxilla (m) and a number for position (e.g., “pm4” = fourth premaxillary tooth). For tooth shape, *caniniform* and *postcaniniform* are used in species with differentiated dentitions to avoid the use of mammalian terms to connote specific positional homology (e.g., “canine”). *Cheek teeth* refer collectively to postcaniniform maxillary and dentary teeth.

Anatomical terms for teeth are described here using “apical” and “basal”, rather than dorsal or ventral, with reference to the crown of the tooth, so that these terms may be applied with similar meaning to both upper or lower tooth rows. The tooth is divided into *crown* and *root*, their junction described as *waisted* when there is a *neck* between crown and root (Fig. 10A). Recently Norman et al. (2011) restricted the term “neck” to refer to mesiodistal crown-root constriction, although there does not appear to be an advantage to doing so. The crown may expand strongly from the root on one or more sides of the tooth. The crown has a *cingulum* (= “girdle” in Latin) when there is a marked constriction below the base of the crown, clearly defining the boundary between the base of the crown and the root. Norman et al. (2011: 234) defined “cingulum” slightly differently, restricting it to labiolingual swelling of the crown base from the root. The cingulum is often more pronounced labiolingually than mesiodistally in ornithischians, but the term as defined here applies to the expanded crown base on one or more sides of a tooth.

The cingulum can round smoothly onto lingual and labial crowns faces, or it can have a well-defined apical margin here termed a *cingular loph*, or crest (Fig. 10A, B). A *cingular ectoloph* and *entoloph* are terms introduced here for apical crests on the cingulum on labial or lingual sides of the crown, respectively. Sometimes these cingular lophs have *cingular denticl*e*s*, and they often curve apically to terminate in the first denticles on the carina, here termed *mesial* and *distal basal denticles*. In *Tianyulong*, for instance, the largest crowns have cingular ectolophs that curve to enlarged basal denticles.

For structures along the carina of the crown, *denticle* and *serration* are used for apicallydirected subconical or tongue-shaped projections versus wedge-shaped projections perpendicular to the carina, respectively. A *denticule* is a denticle-like structure at a finer level of ornamentation; denticules ornament the edge of the tongue-shaped denticles in some ornithischians. Although denticules are generally restricted to larger-bodied euornithopods such as *Ouranosaurus* (Fig. 53B), the heterodontosaurid *Manidens* was recently described with denticulate ornamentation on individual denticles (Pol et al. 2011). A centrally located ridge on a crown surface is termed a *primary ridge* and usually terminates in the apical denticle; ridges to either side, which are often shorter and less prominent, are termed *secondary ridges*. Some crowns have mesial or distal *marginal ridges* that extend from the cingulum to the first mesial or distal marginal denticles as in *Lycorhinus*. Other crowns are distinctly recessed just apical to the cingulum, here termed a *paracingular fossa*. In *Heterodontosaurus*, for example, mesial and distal paracingular fossae are present to each side of the base of the primary ridge in most cheek teeth (Fig. 10A, B).

The teeth in heterodontosaurids are anchored in individual sockets. Mesial and distal extremities of the crowns sometimes overlap *en echelon*, and wear on adjacent crowns can approximate the same in species with significant tooth wear. No heterodontosaurid, however, possesses true “tooth batteries”, despite recent use of this term for the dentition of *Heterodontosaurus* by Norman et al. (2011). A *tooth battery* refers to a more advanced condition, in which the alveoli coalesce into a confluent trough filled with teeth that are locked together as a single tooth-to-tooth supported, composite structure. This has long been the general understanding of this term, with acknowledgement that heterodontosaurids including *Heterodontosaurus* do not exhibit this level of dental integration (Crompton and Charig 1962). Tooth batteries are now known to occur in neoceratopsians, iguanodontians and rebbachisaurid sauropods (Sereno and Wilson 2005). Heterodontosaurid teeth, in contrast, erupt independently within separate alveoli.

**Taxonomic terms.** Autapomorphies, or character states that are derived for a species or monotypic genus, are key to taxonomic diagnosis. These features constitute the evidential basis for recognition at the finest taxonomic level. Traditional taxonomic practice is less stringent, with other kinds of features added to taxonomic diagnoses that merely help to “differentiate” a taxon. If a particular species lacks the derived attributes of another species, for example, that absence might also be included. The traditional “differential diagnosis” of a taxon, thus, aims to differentiate rather than solely to distinguish (Sereno 1990). The problem in this connection is that distinguishing autapomorphies can get lost in the shuffle. Yet it is this subset of features of a taxon that provides the evidence for grouping specimens under a taxonomic name. In this paper, taxonomic diagnoses for species and monotypic genera (currently all heterodontosaurid genera are monospecific) are limited to potential *autapomorphies*; diagnoses for suprageneric taxa are similarly limited to potential *synapomorphies*.

*Phylogenetic definitions*are used to clarify the meaning of the few suprageneric taxa formally considered in this study. For heterodontosaurids that is limited to phylogenetic definitions for Heterodontosauridae and one new subfamily. Proposed or revised phylogenetic definitions are viewed as mutable recommendations rather than more permanent constructs requiring a formal code of nomenclature (Sereno 2005a). Background information including historical usage of taxa and previous definitions is available online for all cited suprageneric taxa (Sereno 2005b; Sereno et al. 2005).

## Results

### Systematic Paleontology. Systematic hierarchy. Dinosauria Owen, 1842. Ornithischia Seeley, 1888

#### Heterodontosauridae

 Kuhn, 1966

http://species-id.net/wiki/Heterodontosauridae

##### Emended diagnosis.

Small-bodied ornithischians with the following features that may constitute heterodontosaurid synapomorphies in phylogenetic context: (1) three or fewer premaxillary teeth; (2) premaxillary teeth increase in size distally; (3) dentary caniniform tooth associated with an arched premaxilla-maxilla diastema; (4) nasal fossa, dorsomedian with rounded lateral margins; (5) jugal flange, ventral embayment of jugal-quadratojugal embayment; (6) jugal horn below orbit, laterally directed and dorsoventrally compressed; (7) postorbital body, arcuate fossa with raised anterior rim; (8) quadrate head included within laterotemporal fossa; (9) quadrate condyle, articular surface ventrolaterally inclined at approximately 30°; (10) quadratojugal T-shaped; (11) predentary processes (lateral, ventral) rudimentary; (12) dentary ramus stoutly proportioned, substantial depth at mid ramus compared to length; (13) fibular mid-shaft and distal end reduced.

##### Phylogenetic definition.

The most inclusive clade containing *Heterodontosaurus tucki* Crompton and Charig 1962 but not *Parasaurolophus walkeri* Parks 1922, *Pachycephalosaurus wyomingensis* (Gilmore 1931), *Triceratops horridus* Marsh 1889, *Ankylosaurus magniventris* Brown 1908.

This stem-based phylogenetic definition (Sereno 2005b) includes, but does not reach beyond, all currently known heterodontosaurids under all proposed phylogenetic interpretations of the position of heterodontosaurids within Ornithischia (e.g., Sereno 1999; Butler et al. 2008). The first and only previous phylogenetic definition proposed for Heterodontosauridae (Sereno 1998: 61) is similar but lacks the negative specifiers of the present definition that stabilize its taxonomic content under alternative phylogenetic relationships.

##### Temporal and geographic range.

Late Triassic (Norian) to Early Cretaceous (Barremian-Aptian), ca. 216–125 Ma (Gradstein and Ogg 2009; Martinez et al. 2011); global distribution includes northern localities (northern China, western North America, Europe) and southern localities (southern South America, southern Africa) (Fig. 1). The record of heterodontosaurids from the Late Triassic currently depends upon the interpretation of the poorly known *Pisanosaurus mertii* (Bonaparte 1976; Sereno 1991) and other fragmentary remains from Upper Triassic rocks elsewhere in Argentina (Báez and Marsicano 2001).

##### Comments.

Kuhn (1966) is identified as the author of the taxon Heterodontosauridae, although Romer (1966) independently proposed the same taxon in the same year (synchronous publication noted by Kuhn 1967: 77, 122). In the literature, some cite Romer as the author of the taxon (e.g., Smith 1997; Sereno 1998; Sereno 2005b), some Kuhn (e.g., Norman et al. 2004, 2011), and some Kuhn and Romer with one author in parentheses (e.g., Steel 1969). Establishing priority by publication date in this case is no longer possible, and, unlike Romer, Kuhn also briefly diagnosed the family-level taxon. Here Kuhn is recognized as the author of Heterodontosauridae (P. Galton, pers. comm.).

Many of the cranial and postcranial apomorphies listed in the emended diagnosis were known previously only in *Heterodontosaurus tucki* but now are known in at least one other heterodontosaurid. When coded into a phylogenetic analysis, some of these features might be repositioned at nodes within Heterodontosauridae (under delayed transformation), given the large amount of missing data in known taxa. The list, nonetheless, attempts to capture as many skeletal modifications that are shared by *Heterodontosaurus tucki* and at least one other basal heterodontosaurid and may characterize the group. The features listed are discussed in more detail below (under Heterodontosaurid monophyly) and in Appendix I.

#### Echinodon becklesii

Owen, 1861

http://species-id.net/wiki/Echinodon_becklesii

Figs 2A 11 [Fig F12] [Fig F13] [Fig F14] [Fig F15] [Fig F16] [Fig F17] [Fig F18] 19 Tables 1 3

Echinodon becklesii Owen, 1861 – Owen (1861, pl. 8, Figs 1, 2); Owen (1874, pl. 2, fig. 22); Galton (1978, Figs 1, 2J-O); Galton (1986, 16.6b-e); Benton and Spencer (1995, fig. 7.16e); Galton (2007, Figs 2.6I-K, 2.7H-K); Norman and Barrett (2002, Figs 7, 8, pls. 1, 2)

##### Lectotype.

**Figure 11.**
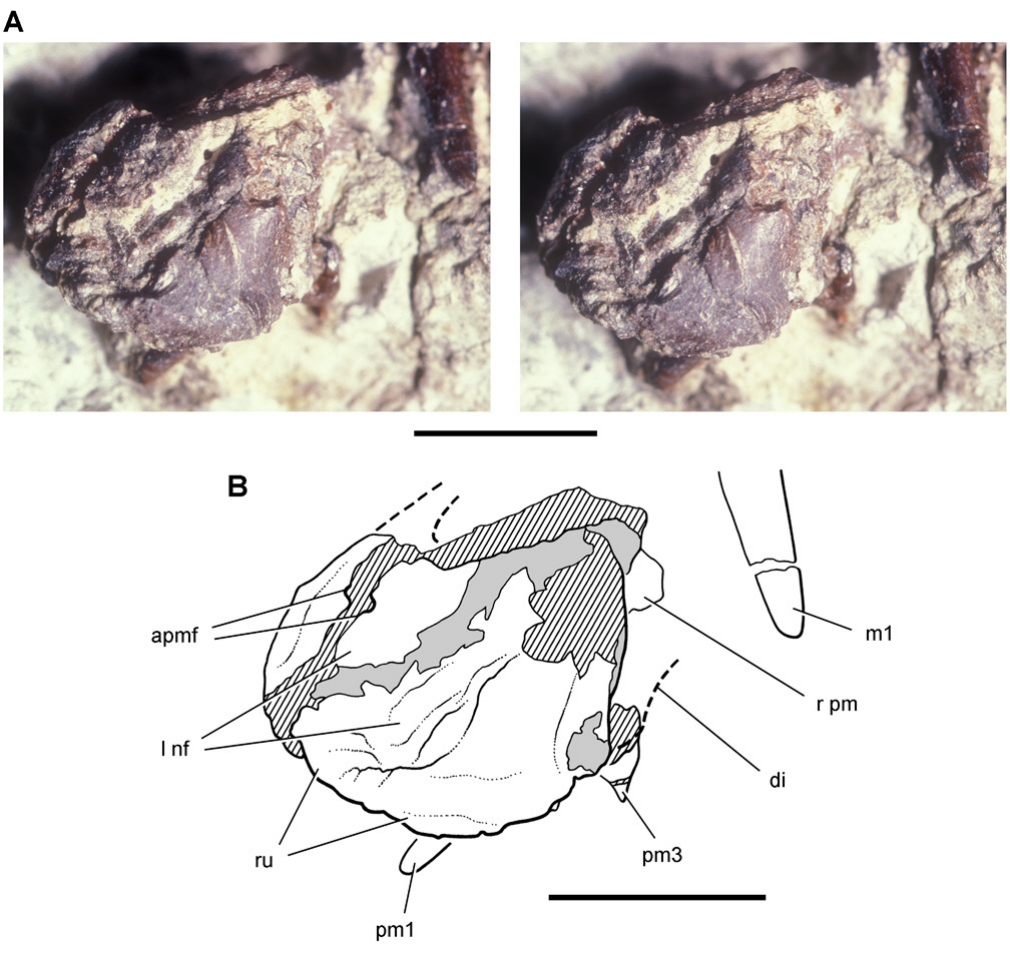
Premaxilla of *Echinodon becklesii* from the Lower Cretaceous Purbeck Formation of England. Left premaxilla in anterdorsolateral view (NHMUK 48209). Stereopair (**A**) and line drawing (**B**). Hatching indicates broken bone; dashed lines indicate estimated edges; tone indicates matrix. Scale bars equal 5 mm. Abbreviations: ***apmf*** anterior premaxillary foramen ***di*** diastema ***l*** left ***m1*** maxillary tooth 1 ***nf*** narial fossa ***pm*** premaxilla ***pm1***, ***3*** premaxillary tooth 1, 3 ***r*** right ***ru*** rugosity.

NHMUK 48209 (Figs 11, 13C, D, part of the left and right premaxillae, anterior part of left maxilla with the caniniform tooth and maxillary teeth 2 and 3, and an impression of the lateral aspect of the posterior ramus of the maxilla and maxillary teeth 4 and 5; NHMUK 48210 (Figs 13A, B, 14), posterior ramus of the maxilla with 6 alveoli and 5 complete or partial crowns, the ventral end of the left lacrimal, the anterior end of the left jugal, and most of the left ectopterygoid. Both specimens belong to the anterior end of a single, partially disarticulated snout embedded in a block that split between the maxillae during, or shortly after, its collection (Owen 1861: pl. 8, Figs 1, 2; Galton 1978: Figs 1A, B; Norman and Barrett 2002: pl. 1, Figs 1, 2).

##### Paralectotypes.

**Figure 12. F12:**
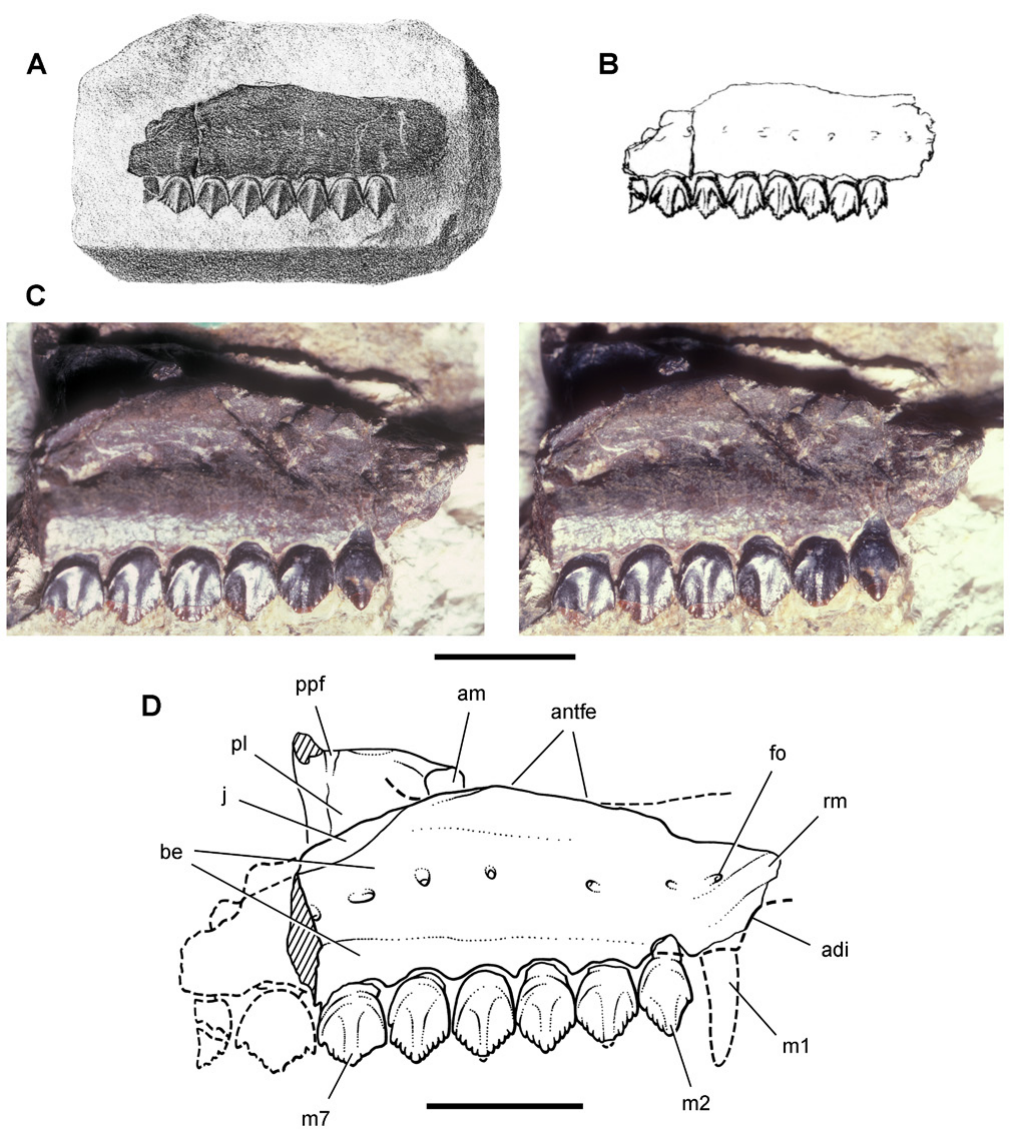
Maxilla of *Echinodon becklesii* from the Lower Cretaceous Purbeck Formation of England. Right maxilla in lateral view (NHMUK 48211). Lithograph (**A**) and line drawing (**B**) from Owen (1861). Stereopair (**C**) and line drawing (**D**). Hatching indicates broken bone; dashed lines indicate estimated edges. Scale bars equal 5 mm. Abbreviations: ***adi*** arched diastema ***am*** articular surface for the maxilla ***antfe*** antorbital fenestra ***be*** buccal emargination ***fo*** foramen ***j*** jugal ***m1***, ***2***, ***7*** maxillary tooth 1, 2, 7 ***pl***  palatine ***ppf*** postpalatine foramen ***rm*** rim.

NHMUK 48211 (Fig. 12), partial right maxilla with maxillary teeth 2-7 with the tip of the right jugal and part of the right palatine; NHMUK 48212, partial right maxilla with 6 teeth; NHMUK 48213 (Fig. 18), partial left dentary with 8 alveoli and 7 teeth; NHMUK 48214, partial right dentary without teeth; NHMUK 48215a, right dentary with 10 alveoli and 9 teeth; NHMUK 48215b (Figs 15–17), left dentary with 10 alveoli and 5 teeth.

*Referred material*. NHMUK 48229, jaw fragment; NHMUK 40723, dentary fragment; DORCM GS 1164-5, 1167, 1171, 1194, 1212-6, 1222-3, isolated teeth.

##### Type locality.

“Mammal Pit” located high on the coastal cliff section near the Zig Zag Path at Durlston Bay, Isle of Purbeck, Dorset, southern England (Fig. 1A); N50°35', W1°55' (Melville and Freshney 1982; Salisbury 2002).

##### Horizon.

Either the Marly or the Cherty Freshwater Member, Middle Purbeck Beds of the Purbeck Formation; Lower Cretaceous, Berriasian, ca. 146-140 Ma (Owen 1861; Clements 1993; Salisbury 2002; Gradstein and Ogg 2009). Although Galton (1978: 151) stated that the lectotype and paralectotype material came from the Mammal Bed (= “dirt bed”; Durlston Bay 83) of the Middle Purbeck Beds (Marly Freshwater Member), no evidence exists to link the fossils to that particular horizon. They may have come from the Feather Bed (Durlston Bay 108) slightly higher in the section (Cherty Freshwater Member).

##### Derivation of the name.

The species name *becklesii*, coined by Owen (1861: 35)after Samuel H. Beckles who found the fossils, has been misspelled several ways in the literature (Norman and Barrett 2002), first as “*becclesii*” by Owen himself (1861: pl. 8) and later as “*becklesi*” (Lydekker 1888) and “*becklessii*” (Galton 1978). S. H. Beckles spelled his surname with a “k” (e.g., Beckles 1854), although variants on his surname have persisted as well (e.g., “Beccles”; Salisbury 2002).

##### Revised diagnosis.

Heterodontosaurid ornithischian characterized by the following six autapomorphies: (1) slender, nearly straight caniniform first maxillary tooth with unornamented anterior and posterior carinae; (2) edentulous anterior dentary margin (as long as two alveoli); (3) only 9 dentary teeth posterior to the caniniform tooth; (4) dentary crowns in the middle of the tooth row that are proportionately taller than opposing maxillary crowns (the apical 50% of middle dentary crowns are denticulate versus 25% of mid maxillary crowns); (5) anteroposteriorly elongate dentary symphysis (maximum length approximately 3 times maximum depth); (6) symphyseal flange ventral to primary dentary symphysis.

##### Description.

The original description of *Echinodon becklesii* is insightful and accurate in most regards (Owen 1861: 35–39, pl. 8). During the ensuing 150 years, the specimens have undergone further preparation and also have sustained some damage and loss (Norman and Barrett 2002: 173). Only a few new fragments and isolated teeth have come to light that may be tentatively referred to *Echinodon becklesii* (Norman and Barrett 2002), and so further information about this heterodontosaurid depends on the original materials. This review attempts to bring together published information and figures (Owen 1861; Galton 1978; Sereno 1991; Norman and Barrett 2002) and new observations on *Echinodon* in order to resolve conflicting statements and gain a better understanding of its morphology and status as a heterodontosaurid. The initial interpretation of *Echinodon* as a heterodontosaurid (Sereno 1991, 1997) was based on several of the observations documented below.

##### Premaxilla.

**Figure 13. F13:**
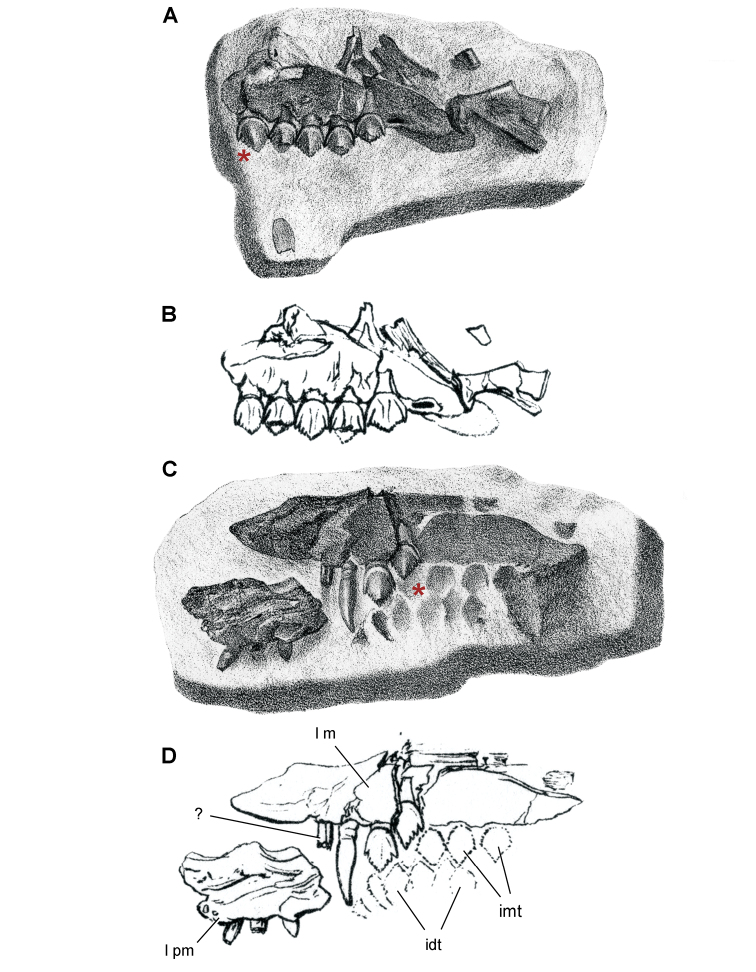
Maxilla of *Echinodon becklesii* from the Lower Cretaceous Purbeck Formation of England. Part (NHMUK 48210) and counterpart (NHMUK 48209) of a block preserving portions of the snout of a skull. **A, B** Part preserving the posterior portion of the left maxilla and portions of the left lacrimal, jugal and ectopterygoid in medial view (NHMUK 48210; reversed from Owen 1861). **C, D** Counterpart preserving portions of the right and left premaxillae and the anterior portion of the left maxilla in lateral view (NHMUK 48209; from Owen 1861). A red asterisk marks a crown on the part (**A**) and its impression on the counterpart (**C**). Abbreviations: ***idt*** impressions of dentary teeth ***imt*** impressions of maxillary teeth ***l*** left ***m*** maxilla ***pm*** premaxilla.

The ventral portions of the left and right premaxillae were originally preserved in mutual articulation in NHMUK 48209 (Owen 1861: pl. 8, fig. 1; Galton 1978: fig. 1A; Fig. 13C, D). Galton (1978: 140, fig. 1J) removed the left premaxilla (accidentally identifying it as the left “maxilla”) to expose the palate of the right premaxilla (also Norman and Barrett 2002: pl. 1, fig. 1). The premaxillae are figured here in their original position disarticulated from the associated left maxilla, both lacking anterodorsal and posterodorsal processes (Figs 11, 13C, D).

Despite two diagonal fractures and some crushing, several details of the left premaxilla have not been described previously. An anterior premaxillary foramen is present and split in two by a fracture with each half slightly separated (Fig. 11). The anterior premaxillary foramen is a good landmark for the anterior margin of the narial fossa, which is preserved as a broad depression extending ventrally from the foramen toward the a rugose rounded alveolar margin. The location of the anterior premaxillary foramen and ventral extension of the narial fossa is very similar to that in *Heterodontosaurus* but unlike the configuration in some other ornithischians such as *Hypsilophodon* (Galton 1974a). The rugose anterior portion of the alveolar margin is edentulous, such that the first premaxillary tooth is set in from the front margin of the premaxilla by a distance equal to two or three alveoli (Fig. 11). The edentulous margin was previously reconstructed (Galton 1978: fig. 2J) somewhat shorter in length (Fig. 19A). The longer edentulous margin more closely resembles the condition in *Heterodontosaurus* and some neornithischians than in basal ornithischians such as *Lesothosaurus* (Sereno 1991), in which the margin is only approximately one alveolus in length.

The posterior end of the alveolar margin is broken away along with part of the root of the third premaxillary tooth, as the specimen is now preserved (Fig. 11). When this portion of the premaxilla is preserved in other heterodontosaurids, it forms the anterior portion of an arched diastema with an inset medial wall. A similar recessed wall appears to be present on the left premaxilla in Owen’s figure (Fig. 13C, D), but this portion of the bone was broken away by the time Galton figured the specimen (1978: fig. 1A, A’; Fig. 11).

The dorsal portion of right and left premaxillae is broken away. Owen (1861: 36) remarked that part of the “boundary of the external nostril” (= external nares) is preserved, but this does not appear to be a natural margin (Fig. 11). The right premaxilla (exposed with removal of the left premaxilla) preserves a flat palatal surface as mentioned by Galton (1978) and Norman and Barrett (2002). A relatively flat secondary palate, however, is common among basal ornithischians and likely primitive (e.g., *Lesothosaurus*; Sereno 1991).

Finally, the position of the premaxilla relative to the maxilla is unknown, as the premaxillae are disarticulated and displaced anteroventral to the maxillae in the only partial cranium known for *Echinodon* (NHMUK 48209; Fig. 13C, D). The anteromedial process of the left maxilla (now only an impression) lies just above the palatal surface of the right premaxilla, not far from its original articulation. Tooth impressions figured by Owen suggest that a dentary was originally present immediately below the maxillary tooth row (Fig. 13C, D). None of the preserved dentary tooth rows have a crown configuration that matches these impressions. This missing dentary, if present, must have been lost during, or shortly after, collection of NHMUK 48209.

Thus it is likely that much of the anterior end of the snout was originally preserved in NHMUK 48209. In *Heterodontosaurus*, *Abrictosaurus*, and *Tianyulong*, the end of the snout is preserved intact, and the premaxillary alveolar margin is offset ventral to that of the maxilla. In an initial reconstruction of *Echinodon*, the alveolar margin of the premaxilla was drawn offset dorsally to that of the maxilla (Fig. 19A). The preserved location and form of the premaxilla in *Echinodon* (NHMUK 48209), to the contrary, suggests that there may have been some ventral offset of the premaxillary alveolar margin as in other heterodontosaurids (Fig. 19B).

##### Maxilla

**Figure 14. F14:**
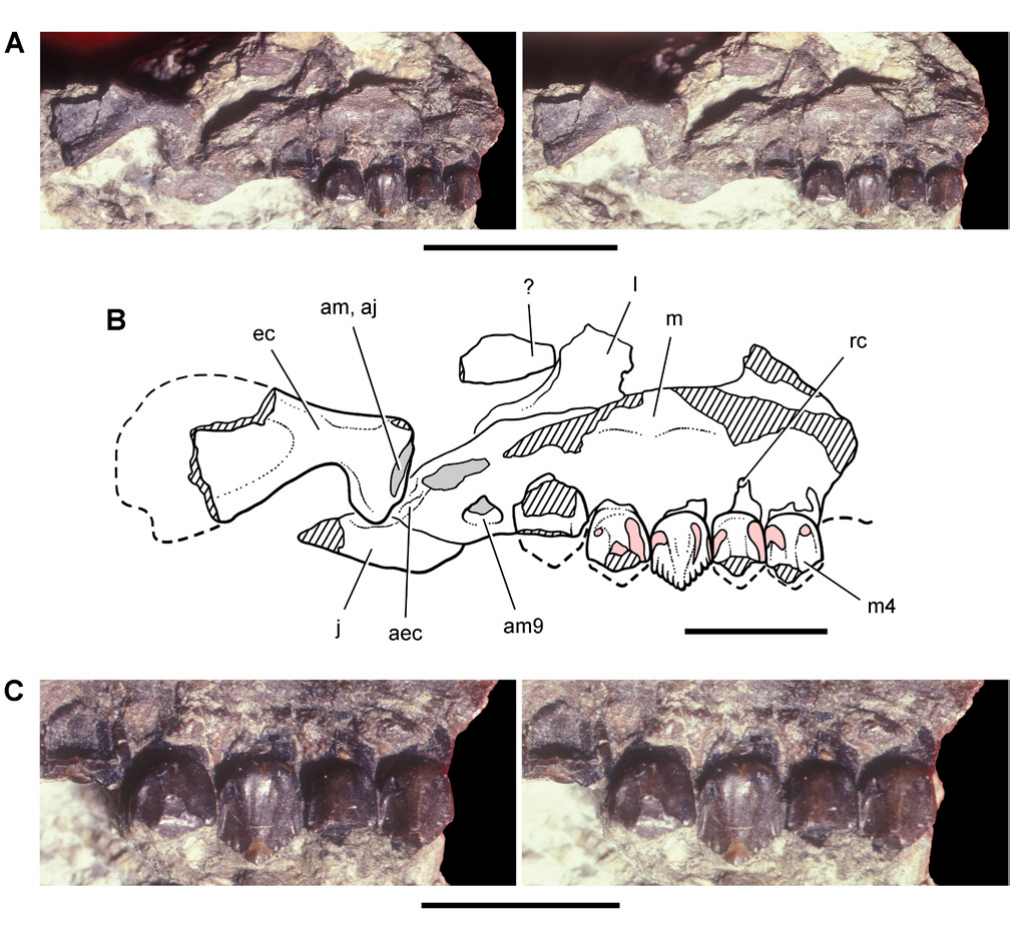
Partial skull of *Echinodon becklesii* from the Lower Cretaceous Purbeck Formation of England. Left maxilla and portions of the lacrimal, jugal and ectopterygoid in medial view (NHMUK 48210). Stereopairs of bones (**A**), line drawing of bones (**B**), and stereopairs of teeth (**C**). Hatching indicates broken bone; dashed lines indicate estimated edges; grey tone indicate matrix; pink tone indicates wear facets. Scale bar equals 1 cm in **A** and 5 mm in **B** and **C**. Abbreviations: ***aec*** articular surface for the ectopterygoid ***aj*** articular surface for the jugal ***am*** articular surface for the maxilla ***am9*** alveolus for maxillary tooth 9 ***ec*** ectopterygoid ***j*** jugal ***l*** lacrimal ***m*** maxilla ***m4*** maxillary tooth 4 ***rc*** replacement crown.

**.** The maxilla is known from three individuals, including the lectotypic left maxilla (NHMUK 48209, 48210; Figs 13, 14) and two partial right maxillae (NHMUK 48211, 48212; Fig. 12). These specimens allow a more complete description of this bone. Preserved sutural contacts of the maxilla include the premaxilla anteriorly, the lacrimal dorsally, the jugal posteriorly, and the palatine and ectopterygoid medially, (Figs 12–14).

A buccal emargination, or cheek embayment, is present along the entire length of the tooth row (Fig. 12). The emargination has been described as “shallow” (Galton 1978: 140), “gentle” (Sereno 199: 1761), and “very shallow” (Norman and Barrett 2002: 177). Galton (1978: 140, fig. 1G’) described and identified the upper border of the buccal emargination as a “slight horizontal ridge just above the tooth row”, and this is doubtless the eminence understood to define the upper boundary of the embayment by all three of the authors cited.

Close inspection of the single maxilla exposing this feature, however, casts doubt on this interpretation (NHMUK 48211; Fig. 12). A gently arched row of neurovascular foramina opens *within* the ornithischian buccal emargination on the maxilla. In *Heterodontosaurus* this row of foramina is present dorsally near the everted upper rim of the maxilla ventral to the antorbital fenestra. The row of large foramina in the maxilla in *Echinodon* represents the same neurovascular openings, which are located ventral to the margin of the antorbital fenestra. The maxilla thus has been flattened postmortem, reducing the depth of the buccal emargination. This transverse compression also has partially collapsed the internal canals associated with the row of foramina and reduced the eversion of the ventral margin of the antorbital fenestra. The opposing dentary emargination has deep proportions and includes a row of neurovascular foramina near the edge of the emargination (Figs 16, 17). The maxilla of *Echinodon*, in sum, appears to have had a buccal emargination (Fig. 19B) similar to that in *Heterodontosaurus* (Crompton and Charig 1974) and *Lycorhinus* (Gow 1990).

The anterior end of the same maxilla provides key evidence for the presence of an arched diastema to accommodate the apical end of a lower caniniform tooth (Fig. 13C, D). The laterally protruding, rounded, arched rim of the diastema is clearly preserved and is very similar to that in *Heterodontosaurus*. Ventral to that rim, the maxilla is inset and forms the posterior portion of a fossa within the diastema for reception of the tip of a dentary caniniform tooth. The margin of the maxilla within the fossa is not complete (*contra* Galton 1978: fig. 1G’). The short section of this margin that is preserved indicates that it arched above the maxillary tooth row as in *Heterodontosaurus* and *Lycorhinus* (NHMUK RU A100). The anterior end of the left maxilla (NHMUK 48210) may have originally included the region of the diastema (Owen 1861: pl. 8, fig. 1), but this region is broken away anterior to the first caniniform tooth (Norman and Barrett 2002: pl. 1, fig. 1).

Norman and Barrett (2002: 182) criticized my previous observation of an arched diastema in *Echinodon* (Sereno 1997), stating “it is impossible to determine whether the premaxilla-maxilla diastema was arched” and that a diastema of any kind is “absent from the available material of *Echinodon*”. Enough of this region is preserved in NHMUK 48211 to remove any doubt that an arched diastema is present in *Echinodon* (Fig. 12), despite the loss of bone fragments and crowns from two of the maxillae since Owen figured them.

##### Lacrimal and jugal.

Portions of both of these bones are preserved attached to two of the maxillae. In NHMUK 48210, the ventral ramus of the left lacrimal, including a portion of the orbital margin, is preserved posterodorsal to the left maxilla (Fig. 13A, B; Galton 1978: fig. 1G’). This portion of the lacrimal was identified previously as the “ascending process” of the maxilla (Norman and Barrett 2002: fig. 14A).

The anterior end of the jugal is preserved in articulation in two specimens. The first is preserved posterior to the antorbital fenestra (Fig. 12C, D; NHMUK 48211), and the second is located at the posterior end of the maxilla (Fig. 13A, B; NHMUK 48210). This portion of the jugal was identified previously as the “overhanging part of maxilla forming the base of lower temporal bar” (Galton 1978: fig. 1B’).

##### Palatine and ectopterygoid.

The right palatine is preserved in partial disarticulation from its lateral contact with the maxilla (Fig. 12C, D; NHMUK 48211). It appears to have rotated dorsally from its natural articulation exposing its ventral surface. The posterior margin has an embayment for a palatal fenestra.

In NHMUK 48210 the palatal bone posteromedial to the maxilla may be a left ectopterygoid. Immediately adjacent to this bone is an articular scar running across the maxilla-jugal suture. This is the lateral anchor for the ectopterygoid in many ornithischians (Fig. 13A, B). The posteromedial margin of the bone is preserved as an impression, the principal exposed surface is concave, and one of its margins is rounded and thickened as seen in cross-section. The position of the bone and its features best match that of an ornithischian ectopterygoid, a tentative identification. Galton (1978: 140, fig. 1B’) identified this bone as a right quadrate with a “twisted” shaft and “incomplete” distal condyles; Owen (1861: 37) and Norman and Barrett (2002: fig. 7B) identified the bone as the left pterygoid. The potential identifications of this bone could be tested by exposure of its opposing side, which is currently obscured by matrix, or via computed tomographic imaging.

##### Predentary.

Although the predentary is unknown in *Echinodon*, its presence is indicated by features on the anterior end of the dentary (Fig. 16A). A large anterior dentary foramen opens anteriorly and passes into an anterodorsally inclined, impressed vessel tract. In other ornithischians, this foramen provides passage for the principal vascular supply to the predentary (e.g., *Lesothosaurus*; Sereno 1991). The predentary in most ornithischians has lateral and ventral processes, and the vascular supply enters the bone near the junction between these processes. In *Echinodon* the vascular tract is deeply incised in a similar location, which is also the case in *Fruitadens* (Figs 9A, 17).

The anterior end of the dentary in *Echinodon* is intermediate between the condition typical of ornithischians (e.g., *Lesothosaurus*, *Hypsilophodon*; Sereno 1991; Naish and Martill 2001) and that in most heterodontosaurids. In the former, the end of the dentary is V-shaped, with articular surfaces for the lateral and ventral processes facing dorsally (or dorsomedially) and ventrolaterally, respectively. In most heterodontosaurids, in contrast, the end of the dentary is slightly expanded dorsoventrally and has a single well-defined, arcuate predentary articular surface that faces anterolaterally. In *Echinodon* the anterior end of the dentary is more rounded than in *Lesothosaurus* (Sereno 1991) or *Hypsilophodon* (Naish and Martill 2001) but lacks a well-defined, arcuate articular surface for the predentary.

**Figure 15. F15:**
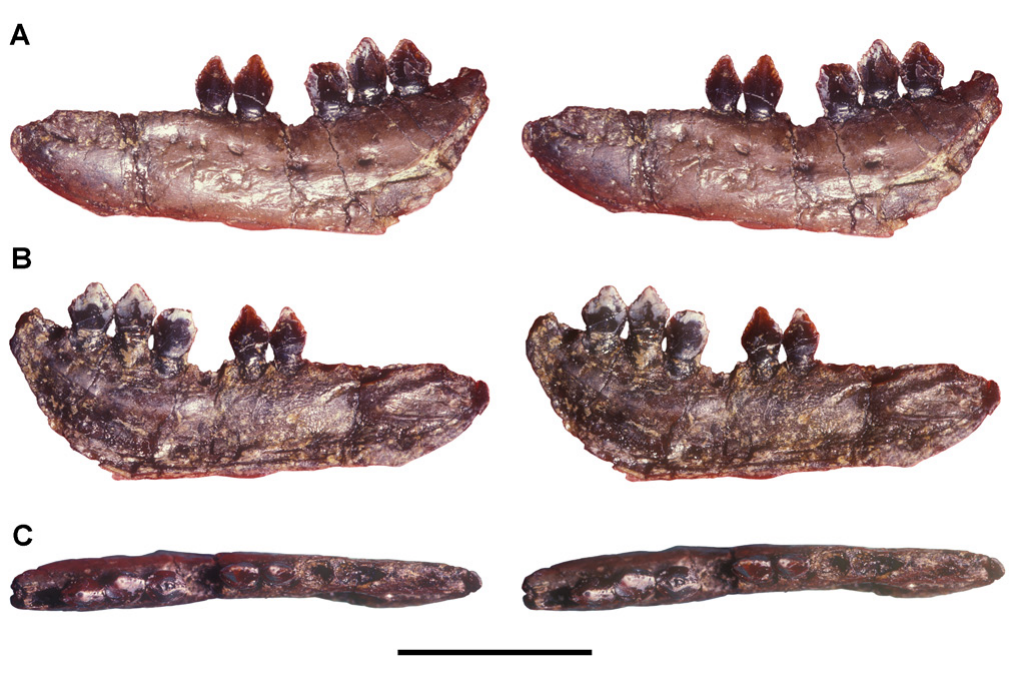
Dentary of *Echinodon becklesii* from the Lower Cretaceous Purbeck Formation of England. Stereopairs of left dentary (NHMUK 48215b) in lateral (**A**), medial (**B**), and dorsal (**C**) views. Scale bar equals 1 cm.

**Figure 16. F16:**
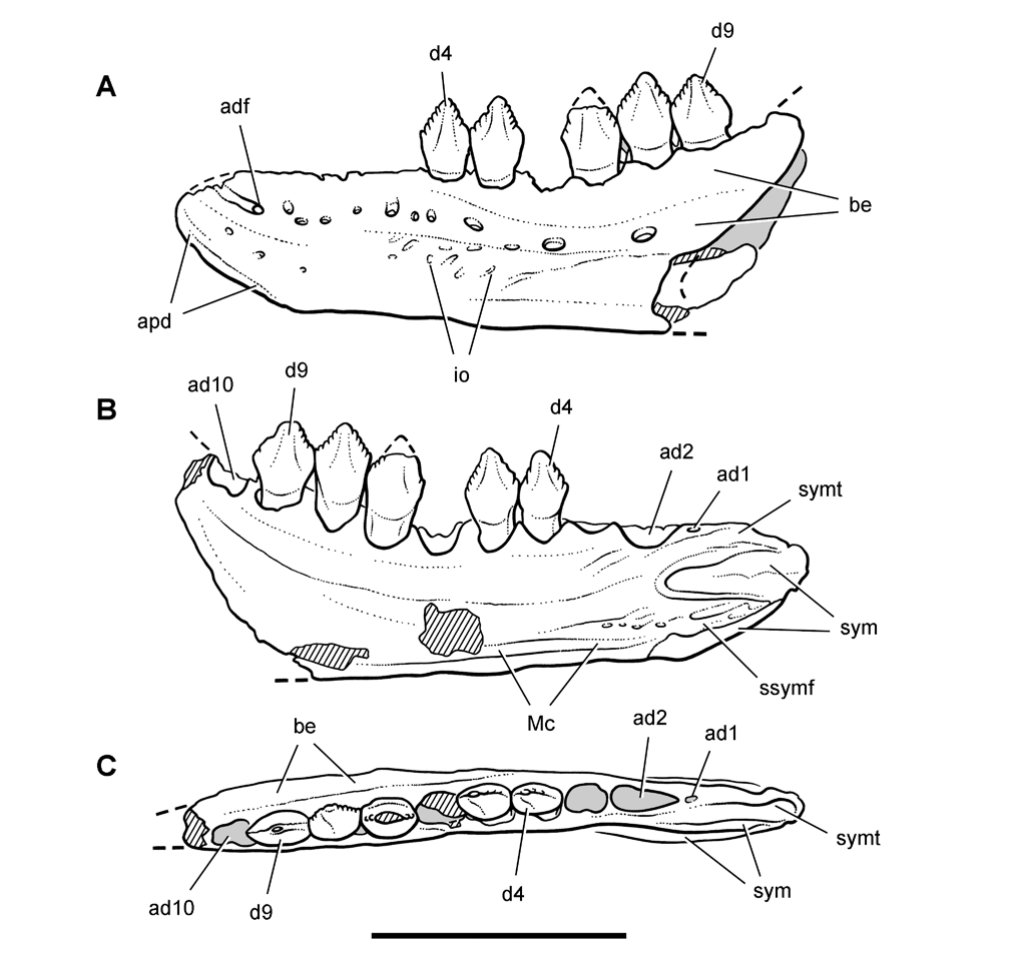
Dentary of *Echinodon becklesii* from the Lower Cretaceous Purbeck Formation of England. Line drawings of left dentary in lateral (**A**), medial (**B**), and dorsal (**C**) views (NHMUK 48215b). Hatching indicates broken bone; dashed lines indicate estimated edges; tone indicates matrix. Scale bar equals 1 cm. Abbreviations: ***ad1***, ***2***, ***10*** alveolus for dentary tooth 1, 2, 10 ***adf*** anterior dentary foramen ***apd*** articular surface for the predentary ***be*** buccal emargination ***d4***, ***9*** dentary tooth 4, 9 ***io*** impressed ornamentation ***Mc*** Meckel’s canal ***ssymf*** subsymphyseal flange ***sym*** symphysis ***symt*** symphyseal trough.

**Figure 17. F17:**
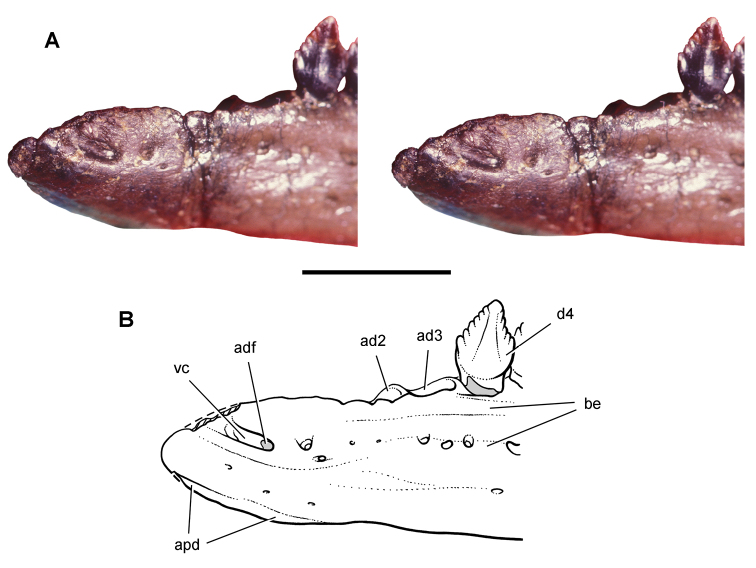
Dentary end of *Echinodon becklesii* from the Lower Cretaceous Purbeck Formation of England. Anterior end of the left dentary in lateral view (NHMUK 48215b). Stereopair (**A**) and line drawing (**B**). Scale bar equals 5 mm. Hatching indicates broken bone; dashed lines indicate estimated edges; tone indicates matrix. Abbreviations: ***ad2***, ***3*** alveolus for dentary tooth 2, 3 ***adf*** anterior dentary foramen ***apd*** articular surface for the predentary ***be*** buccal emargination ***d4***dentary tooth 4 ***vc*** vascular canal.

The dentary does not have a well-defined surface dorsal to the foramen for articulating with the predentary, and so a projecting lateral predentary process probably was not present. The ventral aspect of the anterior end of the dentary has as a subtle smooth articular facet (Figs 15–17). It is not as well marked as in basal ornithischians, where the ventral edge of the dentary is strongly beveled for the median ventral process (e.g., *Lesothosaurus*; Sereno 1991). Nor is it comparable to that in some heterodontosaurids such as *Heterodontosaurus*, in which the articular surface for the predentary is well-defined and trough-shaped. Several small neurovascular foramina are present between dorsal and ventral articular areas for the predentary, an area that clearly would not have been covered by the predentary (Fig. 17). In sum, the predentary in *Echinodon* appears to have a fairly loose articular relation with the dentaries.

Previously Galton (1978: Figs 1C’, 2J) inferred the presence of a predentary in *Echinodon* but misinterpreted the anterior dentary foramen and its associated impressed vessel channel as an articular slot for a prong-shaped lateral predentary process (Fig. 19A). Although a lateral predentary process is present in *Hypsilophodon* (Galton 1974a: fig. 10) and other ornithischians, the dentary in *Echinodon* does not show a facet for such a process. Norman and Barrett (2002: 177) stated that they could not locate any articular surface for the ventral predentary process, but a smooth, flattened articular surface runs along the anteroventral edge at the anterior end of both right and left dentaries (Fig. 17).

##### Dentary.

The dentary is best known from paired right and left sides (Figs 15–17; NHMUK 48215a, b) and partial left and right dentaries (NHMUK 48213, 48214). Originally exposed on slabs of rock, all four dentaries were prepared free of matrix prior to their re-description by Galton (1978). Only a fragmentary dentary has survived from a fourth specimen, which originally included a portion of the left lower jaw (Owen 1861: pl. 8, Figs 6–8; NHMUK 48214).

The dentary in *Echinodon* has particularly stout proportions, even when compared to other heterodontosaurids. Its depth at mid-length is approximately 30% of its length (from anterior extremity to the anterior margin of the external mandibular fenestra) and approximately 40% of the length of the postcaniniform tooth row (Figs 15A, C, 16A, C). In lateral view, the anterior end of the dentary has a subtriangular shape with a strongly beveled anteroventral margin for contact with the predentary (Fig. 17). The middle section of the denary ramus is nearly parallel-sided, expanding only slightly in depth posteriorly. An arched row of relatively large neurovascular foramina opens along the ventral margin of a deep buccal emargination. As in other heterodontosaurids, this emargination tapers in depth near the alveolus for the caniniform tooth in advance of the anterior end of the dentary (Figs 15A, 16A, 17).

The coronoid process is distinctly expanded at mid-length, resulting in a diamond-shaped, rather than tapered, process (NHMUK 48215a, 48213; Owen 1861: pl. 8, fig. 8; Galton 1978: fig. 1D; Barrett 2002: pl. 2, fig. 3). The central axis of the coronoid process angles posterodorsally at about 45° to the long axis of the dentary, as best preserved in NHMUK 48215b and NHMUK 48213 (Fig. 19B). Postmortem crushing has increased the inclination of the coronoid process in one dentary (NHMUK 48215a). In basal ornithischians such as *Lesothosaurus* (Sereno 1991), in contrast, the coronoid process is narrow and tapered (finger-shaped) in lateral view, and the coronoid process is less strongly upturned, angling posterodorsally at about 30°.

The symphysis at the anterior end of the dentary is V-shaped (Figs 15B, 16B). The main articular surface is an oval, raised and textured platform with its long axis horizontal. This articular surface is located almost entirely anterior to the dentary caniniform tooth rather than directly ventral to the caniniform tooth as in *Heterodontosaurus*. A subtriangular fossa lies between the main symphyseal articulation and a secondary flat symphyseal surface, which is located along the ventral margin. This flat surface may have served as a median buttress or stop, as it is not textured for ligament attachment like the main symphyseal surface.

The anterior ends of the dentary are not inturned to form a spout shape as in most ornithischians. The symphysis in *Echinodon*, nevertheless, does not lie on the medial plane of the dentary ramus, but rather is elevated from that plane toward the midline. As a result, there is a narrow dorsoventrally concave surface dorsal to the symphysis and medial to the mesialmost teeth (Figs 15C, 16C). A narrow trough-shaped surface thus is present dorsal to the symphysis as in *Heterodontosaurus*. The symphysis also projects medially in *Lycorhinus* (NHMUK RU A100) and *Heterodontosaurus* (SAM-PK-K1332). The main derived features in *Echinodon* include the elongate symphyseal area, the anterior position of the symphysis relative to the dentary tooth row, and the accessory ventral symphyseal surface near the ventral margin of the dentary.

Galton (1978: 140, fig. 1C’) described and figured the buccal emargination of the dentary as a “small cheek” excluding the row of major neurovascular foramina. Norman and Barrett (2002: 177) also described the buccal emargination of both the dentaries and maxillae as “very shallow.” Transverse compression of several of the dentaries, however, has reduced the depth of the emargination, which must incorporate the principal row of neurovascular foramina. An area of impressed ornamentation is present just below the row of neurovascular foramina, suggesting the presence of tightly adhering integument below the buccal emargination (Figs 15A, 16A).

Galton (1978: 140) described the coronoid process as “prominent”, whereas Norman and Barrett (2002: 177) described it as “low”. One very appropriate comparison is the basal ornithischian *Lesothosaurus* (Sereno 1991: fig. 13F). *Echinodon* more closely resembles the more strongly upturned, transversely expanded process in *Heterodontosaurus* (see below) than the tapered process of shallow inclination in *Lesothosaurus* (Fig. 19B).

##### Premaxillary teeth.

There are three premaxillary teeth in middle and posterior portions of the premaxilla, preceded by an edentulous margin several alveoli in length (Figs 11, 19B). The first and third crowns are preserved with some breakage and loss since Owen first figured them (Fig. 13C, D). Only small fragments of root and crown of the second tooth remain, the base of which was originally preserved. The crowns are slightly swollen, the mesial side of the crown base more bulbous than the distal side, and have smooth surfaces without denticles or serrations. The crowns are gently recurved with apices set slightly distal to the center of the crown base. As in other heterodontosaurids, the third premaxillary tooth is slightly larger than the first. In other ornithischians, premaxillary crowns tend to be subequal in size and have denticulate mesial and distal carinae (e.g., *Lesothosaurus*, *Hypsilophodon*; Naish and Martill 2001; Sereno 1991). Little else can be said about the premaxillary teeth given their state of preservation.

Galton (1978) reconstructed the premaxillary tooth row with crowns of equal size (Fig. 19A), and Butler et al. (2012; 6) reported that crown size does not increase posteriorly in the premaxillary series. The preserved portions of pm1 and 3 (Figs 11, 19B), however, clearly show an increase in size as occurs in most other heterodontosaurids.

##### Maxillary teeth.

There are nine maxillary teeth (Figs 12–14), the first a nearly straight, transversely compressed caniniform tooth lacking any ornamentation on mesial or distal carinae (Fig. 11). Preserved only in the lectotypic left maxilla, the caniniform tooth has a relatively straight and slender crown that extends only a short distance below more distal maxillary crowns (Figs 12D, 13C, D). The maxillary caniniform tooth is preceded by an arched diastema for reception of the dentary caniniform tooth. The maxillary caniniform tooth is smaller than the dentary caniniform tooth (Fig. 19B), judging from the upper diastema and lack of an opposing lower diastema between the caniniform and third dentary tooth (Figs 15C, 16C). In *Echinodon*, thus, the upper caniniform tooth is positioned distal to a lower caniniform. In other heterodontosaurids, in contrast, the large third premaxillary crown is positioned mesial to a lower caniniform tooth (e.g., *Lycorhinus*, *Heterodontosaurus*).

Owen briefly described a second more mesial caniniform tooth in the maxilla based on a fragment and possible impression (Fig. 13C, D). Galton (1978) regarded the fragment as a basal piece of the relatively complete caniniform tooth. Norman and Barrett (2002: fig. 7A), however, added to the diagnosis the possibility of a second caniniform tooth based on Owen’s figures. Neither the tooth fragment nor the potential impression has survived. Available specimens, nonetheless, clearly indicate that only one caniniform is present at the anterior end of the maxilla. There is only a single empty alveolus for a caniniform tooth distal to the arched diastema in a right maxilla (Fig. 12), as correctly described by Galton (1978: 143).

There are seven or eight postcaniniform teeth. Owen described the two lectotypic specimens that represent part and counterpart of a single specimen that was split from a single block of matrix. He indicated how the tooth rows on the opposing pieces should be aligned (Fig. 13). In the anterior piece (NHMUK 48209), there are two postcaniniform teeth; in the posterior piece (NHMUK 48210), there are five teeth plus one empty alveolus documenting the distal end of the tooth row. Owen correctly identified one of the teeth in the anterior piece as a replacement crown erupting beneath the anteriormost tooth in the posterior piece. Following this alignment, the total number of teeth in this complete maxillary series is eight (one caniniform tooth followed by seven postcaniniform teeth) (Fig. 19B). Based on the same specimens, Galton (1978) suggested there are as many as 10 or 11 maxillary teeth, although his reasons for this higher number were not given. A second maxilla preserves the anteriormost alveolus for a caniniform tooth followed by six postcaniniform crowns (Fig. 12). A posterior piece with one and one-half crowns was originally present (Owen 1861: pl. 8, fig. 3), suggesting that this individual had one additional postcaniniform tooth. On the basis of the available collection, thus, *Echinodon* has eight or nine teeth in the maxilla, including a mesial caniniform tooth followed by seven or eight teeth with denticulate crowns (Fig. 19B).

The first postcaniniform tooth (second maxillary tooth) has taller crown proportions than succeeding maxillary teeth; the crown is narrower and the denticulate portion of the crown is approximately 45% total crown height (Fig. 12C, D). Although the distalmost crown is not preserved, all of the remaining maxillary crowns are very similar in size and shape. In other heterodontosaurids, crown size is more variable and often substantially larger toward the middle of the maxillary series. Except for marginal ridges along mesial and distal crown edges, there are no ridges on either labial or lingual crown surfaces. There is a rounded median eminence, which is low on both sides but possibly slightly stronger on the lingual side (Figs 12, 14). There are approximately 8 to 10 denticles to each side of the apical denticle in most crowns. This denticle count is best observed in a nonfunctioning replacement crown in the second alveolus (NHMUK 48209). Galton (1978) identified the median eminence as a “ridge” and suggested that there are fewer (only 5 or 6) denticles to each side of the crown apex.

The enamel is symmetrical on each side of the maxillary crowns as is visible in the cross-section of several crown tips. Wear facets are present on raised areas of the lingual face of the crowns of all fully erupted maxillary crowns that are well preserved and exposed in lingual view (Fig. 14B, C). These facets are more fully described below (see Discussion, Wear).

##### Dentary teeth.

The dentary tooth row has 11 teeth, based on evidence from three dentaries with nearly complete alveolar margins (NHMUK 48214, 48215a, 48215b). The first dentary tooth must have had a very small peglike crown as in *Lycorhinus*, as the size of the alveolus is much smaller than any other in the dentary (Figs 15C, 16C). The root and base of the crown of this small tooth is preserved in one dentary (NHMUK 48214), and the small alveolus is preserved in the other two dentaries. The second dentary tooth, in contrast, is larger than all others, and judging from the elongate alveolus housed a caniniform tooth (Figs 15C, 16C). A substantial edentulous margin precedes both of these alveoli, a feature that distinguishes *Echinodon* from other heterodontosaurids and other basal ornithischians (Fig. 19B).

The small first dentary alveolus has never been described. Galton (1978: Figs 1C’, F’, 2J) figured the large second alveolus and indicated the presence of a more mesial alveolus in one of the specimens (NHMUK 48215b). He did not comment on the size of these teeth and reconstructed the dentary tooth row without caniniform or peglike anterior teeth (Fig. 19A). The initial suggestion that *Echinodon* has a caniniform dentary tooth (Sereno 1997) was criticized by Norman and Benton (2002: 182) who stated “no known specimen displays evidence of either *in situ* caniniforms or natural moulds thereof”. Technically speaking, of course, the caniniform tooth itself is not preserved in any available specimens. Nonetheless, a caniniform tooth was surely present in *Echinodon*, given the size and shape of the alveolus in three available dentaries and the evidence of an opposing arched diastema in the maxilla. Like the caniniform teeth in other heterodontosaurids (e.g., *Fruitadens*; Butler et al. 2010: fig. 3c, d), the alveolus and root are larger than adjacent crowns and angle ventrodistally rather than vertically. The enlarged alveolus in *Echinodon*, thus, should exhibit these features in a computed tomographic scan (Figs 15, 16).

**Figure 18. F18:**
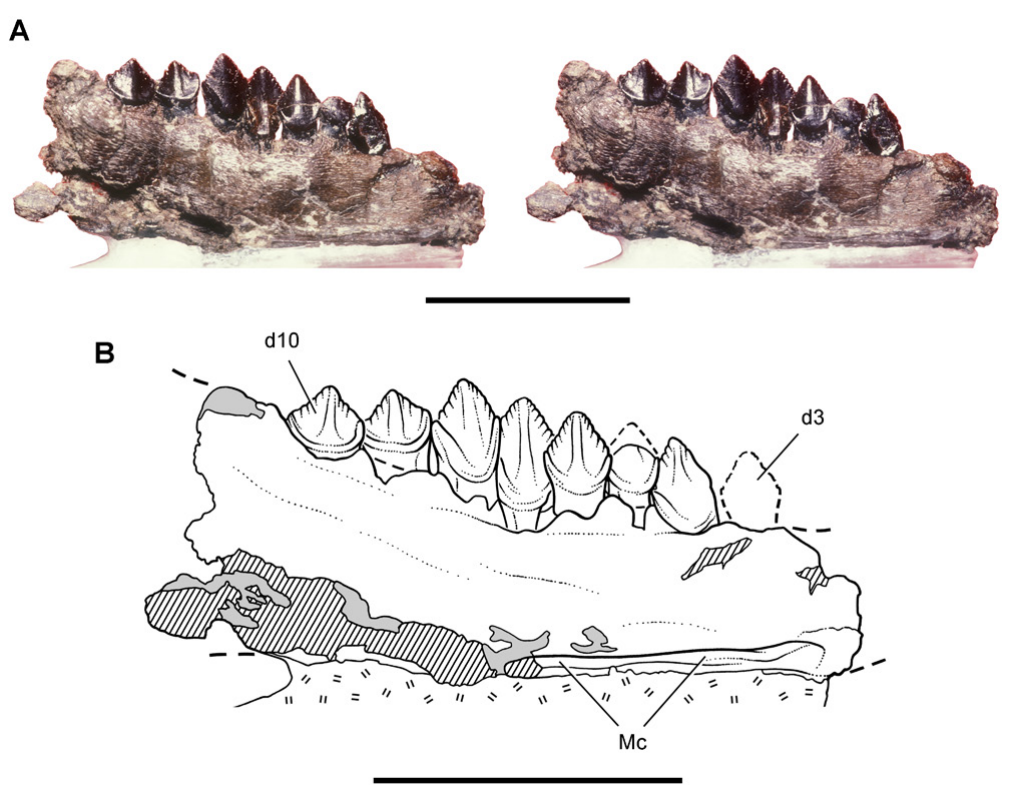
Dentary of *Echinodon becklesii* from the Lower Cretaceous Purbeck Formation of England. Portion of the left dentary (NHMUK 48213) in medial view. Stereopair (**A**) and line drawing (**B**). Hatching indicates broken bone; dashed lines indicate estimated edges; tone indicates matrix; hash marks indicate carbowax support. Scale bars equal 1 cm. Abbreviations: ***d3***, ***10*** dentary tooth 3, 10 ***Mc*** Meckel’s canal.

**Figure 19.**
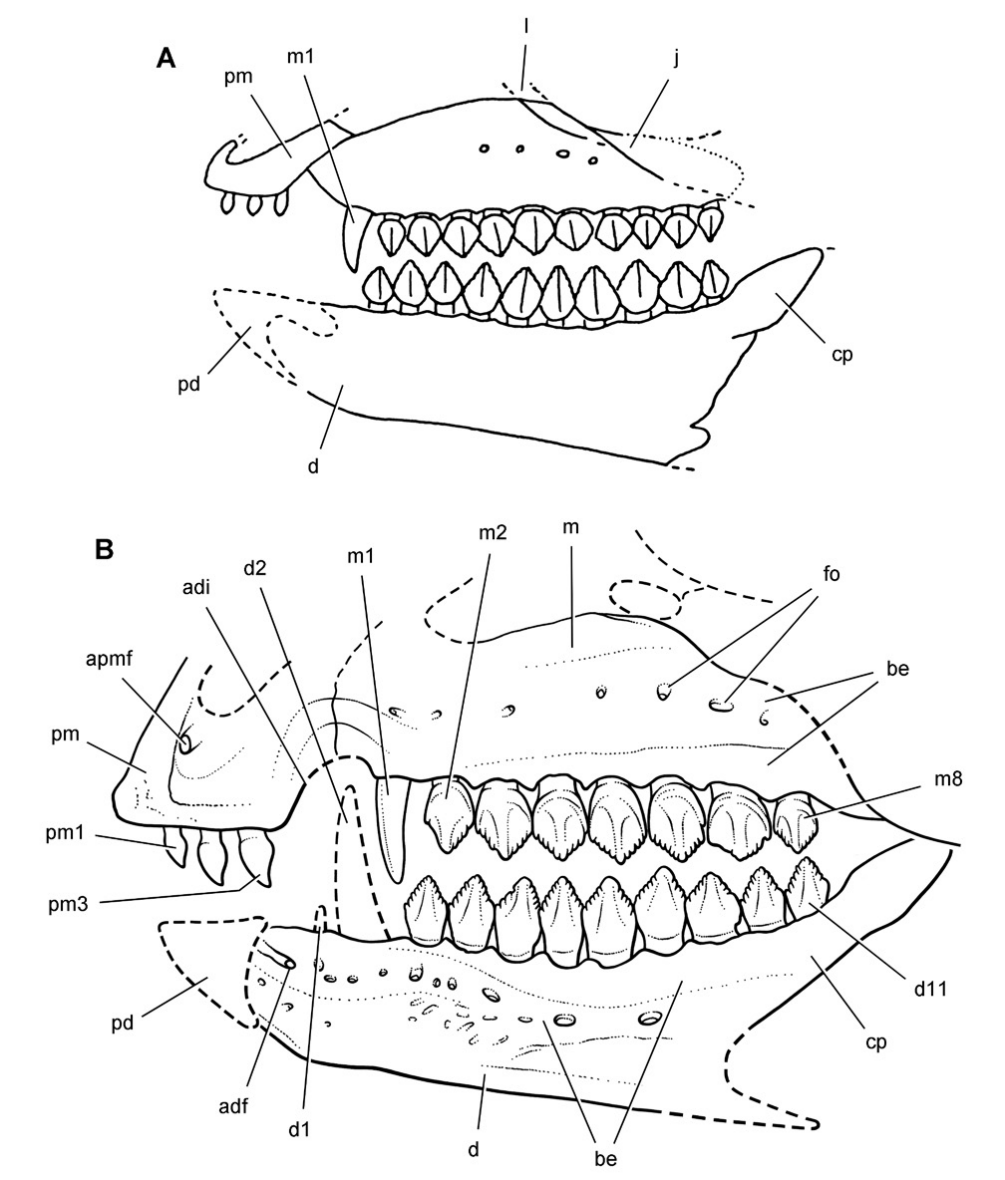
Skull of *Echinodon becklesii* from the Lower Cretaceous Purbeck Formation of England. Skull reconstructions in left lateral view. **A** From Galton (1978). **B** This study. Dashed lines indicate estimated edges and sutures. Abbreviations: ***adf*** anterior dentary foramen ***adi*** arched diastema ***apmf*** anterior premaxillary foramen ***be*** buccal emargination ***cp*** coronoid process ***d*** dentary ***d1***, ***2***, ***11*** dentary tooth 1, 2, 11 ***fo*** foramen ***j*** jugal ***l*** lacrimal ***m*** maxilla ***m1***, ***2***, ***8*** maxillary tooth 1, 2, 8 ***pd*** predentary ***pm*** premaxilla ***pm1***, ***3*** premaxillary tooth 1, 3.

There are eight and nine postcaniniform dentary teeth, respectively, in the left and right dentaries of NHMUK 48215. The left dentary, however, is incomplete posteriorly (Figs 15, 16) and appears to be missing the smaller distalmost tooth preserved on the right side. In the right series, dentary teeth 10 and 11 (postcaniniform teeth 8, 9) have progressively smaller crowns unlike the last two subequal alveoli on the left side. A total of 11 dentary teeth is also consistent with the teeth and alveoli in two other relatively complete dentaries (NHMUK 48213, 48214; Fig. 18). Galton’s (1978) estimate of 10 dentary teeth, therefore, is one too few (Fig. 19A), as he did not count the peg-shaped first dentary tooth.

The crowns of postcaniniform dentary teeth are well separated from their roots and have taller proportions than opposing maxillary crowns (Fig. 19B). All but the posterior three crowns are diamond-shaped, rather than subtriangular. The dorsal 50% of each of these crowns is denticulate, as opposed to approximately 25% in opposing maxillary crowns. Norman and Barrett (2002: 177) stated that the denticles of both dentary and maxillary teeth are “confined to the apical one-third of the tooth crown”, but this is not true for crowns in the middle of the dentary series. As in postcaniniform maxillary crowns, postcaniniform dentary crowns typically have about 8 to 10 denticles to each side of the apical denticle. Likewise, only a median eminence is present on labial and lingual crown faces, and the roots are subconical and tapered where they are exposed (NHMUK 48215a).

The enamel is symmetrically distributed on each side of the dentary crowns as in maxillary crowns. Enamel also appears to cover a flattened subtriangular area on the mesial and distal sides of the crown base between the crown faces and root. This surface, which is present only on the largest crowns, was described previously as exposed dentine and highlighted as unique to *Echinodon* (Owen 1861; Galton 1978). I am unable to verify the absence of enamel or the fact that this surface is a unique, diagnostic feature of *Echinodon*.

##### Skull reconstruction.

The reconstruction of the snout and dentition of *Echinodon* (Fig. 19B) is based on the original specimens and figures in Owen (1861) and differs from a previous reconstruction (Figure 19A) in several regards. The premaxilla is shown with its alveolar margin offset ventral to the maxillary tooth row and with a significant edentulous border anterior to the first premaxillary tooth. The maxilla has a shorter series of postcaniniform teeth, above which is a significantly deeper buccal emargination. Dentary tooth 1 is rudimentary and followed by a large caniniform tooth, the tip of which inserts into an inset, arched diastema between the premaxilla and maxilla. The dentary also has a deeper buccal emargination, which dissipates anteriorly as it passes near the base of the caniniform tooth. Finally, the anterior end of the dentary, which articulates with a reconstructed predentary lacking processes, has an anterior dentary foramen with an incised vascular canal, above which is a significant edentulous margin. The new reconstruction removes any reasonable doubt about the heterodontosaurid status of *Echinodon*.

#### Fruitadens haagarorum

Butler et al., 2010

http://species-id.net/wiki/Fruitadens_haagarorum

Fig. 9A Tables 1 3

Fruitadens haagarorum Butler et al., 2010 – Callison and Quimby (1984: fig. 3B, C); Callison (1987, fig. 4); Kirkland (2006: fig. 22A); Galton (2006: fig. 2.7A–G); Galton (2007: Figs 2.6E–G, 2.7A–G); Butler et al. (2010: Figs 1–3); Butler et al. (2012: Figs 1–7, 8A, B, 9–16)

##### Holotype.

LACM 115747, adult with partial maxillae and dentaries, cervical, dorsal, sacral and caudal vertebrae, proximal right femur, proximal and distal ends of the tibiae, and partial right metatarsal 1 (Butler et al. 2010: fig. 2b, e, i; Butler et al. 2012: Figs 1, 2–4, 8A, 9–12, 13A–E, 14G, H).

##### Referred material.

LACM 115727, adult partial postcranial skeleton with partial cervical, dorsal and caudal vertebrae, partial right and left femora, and an articulated distal left tibia and coossified astragalocalcaneum; LACM 120478, subadult with left humerus, distal left femur, and an articulated left tibia, fibula and coossified astragalocalcaneum; LACM 120602, distal caudal vertebra, left astragalocalcaneum, partial metatarsus and pes; LACM 128258, subadult with right premaxilla, partial left maxilla, partial left and right dentaries, and one dorsal and one distal caudal vertebra; LACM 128303, anterior left dentary (Butler et al. 2010, 2012).

##### Type locality.

Fruita Paleontological Area, approximately 10 kms southwest of Fruita, Mesa County, west-central Colorado, USA; approximately N39°10', W108°48'.

##### Horizon.

Just above the “clay change” near the base of the Brushy Basin Member and about 100 m from the base of the Morrison Formation (Kirkland et al. 2005: fig. 6; Kirkland 2006); Upper Jurassic (early Tithonian), ca. 153 Ma (Kirkland 2006; Gradstein and Ogg 2009). The boundary between the Salt Wash and Brushy Basin Members has undergone revision in the Fruita Paleontological Area (Kirkland 2006). Galton (2002) reported that the fossils were found in the Salt Wash Member but later correctly cited the overlying Brushy Basin Member as the unit of origin (Galton 2007).

##### Revised diagnosis.

Heterodontosaurid with (1) a discordantly small dentary tooth immediately distal to the caniniform dentary tooth and (2) a prominent anteromedial flange on the distal end of the tibia.

##### Comments.

Butler et al. (2010: 376) listed nine features in the original diagnosis for *Fruitadens haagarorum*, indicating that some were primitive and others “autapomorphic” at different phylogenetic levels. Butler et al. (2012: 3) added one character, “small foramen on the anteroventral aspect of the medial dentary”, for a total of ten features.

This suite of features constitutes a differential diagnosis—a unique combination of the features that describes a monospecific genus rather than a set of autapomorphies hypothesized to have arisen in the immediate ancestry of the taxon (Sereno 1990). In the revised diagnosis above, a higher bar is applied that restricts listed features to those that are plausibly unique to *Fruitadens* or derived for *Fruitadens* within Heterodontosauridae. One of the features stands out as plausibly unique to *Fruitadens* and present in the holotype—the prominent anteromedial flange on the distal end of the tibia (Butler et al. 2010: fig. 2i). Comparison to other heterodontosaurids, however, is limited to *Heterodontosaurus*, which does not exhibit the condition. The small medial dentary foramen, which was added by Butler et al. (2012) as an autapomorphy, is not present in one of the two dentaries that preserve the region (Butler et al. 2012: fig. 5D).

One dental feature listed in the revised diagnosis may be an autapomorphy but is homoplasious among heterodontosaurids. The dentary tooth immediately distal to the caniniform tooth in *Fruitadens* is unusually small, its crown apparently somewhat smaller than the rudimentary first dentary tooth (Fig. 9A). *Heterodontosaurus* and a new taxon from southern Africa, *Pegomastax* gen. n. sp. n., are the only other heterodontosaurids with a discordantly small tooth immediately distal to the caniniform tooth.

The other features listed in the diagnosis by Butler et al. (2010, 2012) are demonstrably primitive within Heterodontosauridae or may be artifacts of preservation. The lack of a maxillary caniniform tooth, for example, is the common condition; only *Echinodon* has a caniniform tooth in the maxillary series among heterodontosaurids. Denticles that extend along more than one-half of the crown occur in *Tianyulong* and in ornithischian outgroups such as *Lesothosaurus*. Other features have broader distributions within Heterodontosauridae, such as the small peglike first dentary tooth, which is present in *Echinodon*, *Lycorhinus*, and *Abrictosaurus*. The dentary caniniform tooth was said to be shorter than in some heterodontosaurids and more nearly equal in depth to noncaniniform dentary crowns. The relative depth of individual crowns, however, depends to a great extent on the stage of replacement, which can be difficult to estimate in the case of caniniform teeth. The dentary caniniform tooth in the Kayenta heterodontosaurid, for example, has a similar relative depth to that in *Fruitadens*, although it is clearly undergoing eruption and would be considerably larger when fully functional (Fig. 9A, B). Most of the caniniform tooth in question (LACM 128258), furthermore, seems to have broken away by the time the specimen was described by Butler et al. (2010: fig. 2d; compare Galton 2007: fig. 2.7B). The pair of foramina on the anterior aspect of the astragalus can be compared only in *Heterodontosaurus* among heterodontosaurids, and there is some evidence the condition is present (SAM-PK-K1332).

The separation of the ascending process of the astragalus as a separate ossification was listed among the autapomorphies. The suture separating the distal tip of the astragalar ascending process, however, seems to continue laterally as a fracture line across the distal shaft of the fibula. The ascending process had been viewed as a separate ossification in the theropod *Dilophosaurus* (Welles 1984); review of this specimen, however, suggests that it also appears to be a postmortem fracture passing through vascular foramina. Persistence of such a suture in *Fruitadens*, in addition, seems unlikely in a taxon distinguished by coossification in this particular region of the limb skeleton (e.g., the tibiotarsus).

##### Discovery.

The six available specimens of *Fruitadens haagarorum* were collected between 1975 and 1980 in the Fruita Paleontological Area from four separate localities above a distinctive horizon (“clay change”) near the base of the Brushy Basin Member of the Morrison Formation (Kirkland et al. 2005; Kirkland 2006). The localities were not discovered in a single horizon but rather within a zone perhaps 10-15 m in thickness above the “clay change” (G. Callison, pers. comm.). Four specimens were described in the initial description (Butler et al. 2010); two additional fragmentary specimens were included more recently (Butler et al. 2012; LACM 120602, 128303).

*Fruitadens* is a small-bodied heterodontosaurid, the adult specimens of which appear to be slightly larger than two other small-bodied heterodontosaurids, *Echinodon* and *Tianyulong* (Table 3). The sole specimen known of the Kayenta heterodontosaurid is the smallest heterodontosaurid on record (skull length estimate of 53 mm), but histologic evidence suggests that it is a subadult roughly the same size as half-grown individuals of *Heterodontosaurus* (e.g., AMNH 24000; Fig. 2C).

##### Association.

The holotype of *Fruitadens* and three referred specimens were described as associated individuals, the supportive evidence limited to the lack of duplicate bones and consistent state of preservation within each specimen (Butler et al. 2010: suppl. info.; Butler et al. 2012). A first-hand account of the discovery of the holotype (LACM 115747) and a referred subadult specimen (LACM 120478) confirms their association as individuals. The first pieces of the holotype were surface-collected on a slope, which led to an *in situ* portion of the specimen that was recovered in a small field jacket (G. Callison, pers. comm.). The subadult specimen LACM 120478, which preserves the most complete long bone lengths, was also found in a confined space by quarrying at a nearby locality 5572 (“Main Callison Quarry”). A referred adult specimen was found at locality 5576 (“George’s Coelurosaur site”), and there is no specific site information available for a referred subadult with the most complete set of jaws (LACM 128258), except that it comes from the same general area (Fruita Paleontological Area).

##### Jaws.

The dentary in *Fruitadens* has a vascularized buccal emargination, presumably as an aid to the processing plant materials within the oral cavity (Fig. 9A). The anterior end of the dentary in *Fruitadens* is subrectangular, whereas in as *Echinodon* the ventral side of the anterior end of the dentary is strongly beveled. Both *Fruitadens* and *Echinodon* have a well-demarcated vessel tract passing from the anterior dentary foramen toward the predentary.

A jaw fragment housing three teeth was identified as a right premaxilla (Butler et al. 2010: fig. 2a). Only the middle tooth preserves the entire crown; only the base of the distal crown is preserved, and the first crown in the series appears to have been lost. Butler et al. (2010: 376) noted that all of these teeth have waisted, subtriangular crowns; there is no development of a caniniform crown. Butler et al. (2012), further, suggested that a small portion of the left premaxilla might be attached across the median palatal suture. A portion of an ascending ramus has been shown as preserved on the premaxilla (Fig. 9A), but no part of this ramus appears to be preserved on the actual specimen (Butler et al. 2012: fig. 7A). In other heterodontosaurids, the narial fossa extends close to the ventral margin of the premaxilla, whereas in *Fruitadens* the narial fossa is not evident in lateral view. The identification of this jaw fragment as a portion of the right premaxilla may be correct, but it exhibits several unusual features as compared to more completely known premaxillae in *Echinodon*, *Tianyulong*, *Lycorhinus*, and *Heterodontosaurus*.

The maxilla in *Fruitadens* has a deep margin between the antorbital fenestra and the maxillary tooth row, although this depth can appear greater with postmortem compression (Fig. 9A). As preserved it most closely resembles the condition in *Echinodon* and *Lycorhinus*. The buccal emargination is approximately one-half as deep in *Tianyulong*, *Abrictosaurus*, and *Heterodontosaurus*. Butler et al. (2010: 376, fig. 2c) correctly identified a partial left maxilla in a subadult (LACM 128258), whereas Galton (2007: Figs 2.6E, 2.7A) identified the same specimen as a right maxilla.

##### Dentition.

The location, size and shape of the anterior dentary teeth differ between *Fruitadens* and *Echinodon*. In *Fruitadens* (Fig. 9A) the first dentary tooth is closer to the anterior end of the dentary than in *Echinodon*, in which a short edentulous margin precedes the first dentary tooth (Galton 2007: fig. 2.7B; Butler et al. 2010: fig. 2d). Both *Fruitadens* (LACM 115747) and *Echinodon* have a small peg-shaped first dentary tooth. Although described as unique to *Fruitadens* (Butler et al. 2010: 376, 378), a rudimentary first dentary tooth (or tiny alveolus) is also known in *Echinodon*, *Lycorhinus* and *Abrictosaurus*. In some heterodontosaurids, including the Kayenta heterodontosaurid, *Pegomastax* gen n. sp. n., and *Heterodontosaurus*, there are no teeth mesial to the caniniform tooth.

The third dentary tooth in *Fruitadens*, or the first “cheek” tooth, is the smallest tooth in the dentary series (Fig. 9A). Although described as present in “other heterodontosaurids” (Butler et al. 2010: 378), such a diminutive tooth is known only in *Pegomastax* gen n. sp. n. and possibly in *Heterodontosaurus*. In other heterodontosaurids such as *Echinodon*, *Lycorhinus*, and *Abrictosaurus*, the first cheek tooth is subequal to successive crowns at the anterior end of the dentary tooth series.

The premaxillary teeth described by Butler et al. (2010) would be unusual in form for heterodontosaurids, but identification of the jaw fragment as a right premaxilla may be problematic as discussed above. Galton (2007: 28) reported the presence of five premaxillary teeth in *Fruitadens*, but there appears to be no evidence in support of this statement.

The largest maxillary crowns in the distal portion of the series have a bulbous cingulum with well-defined basal and apical edges (Butler et al. 2010: fig. 2b: Galton 2007: fig. 2.7C, F, G). The apical edge of the cingulum is maintained in the center of the crown base, where the median eminence joins the base of the crown (Fig. 9A). This well-defined cingulum is present only in the largest crowns and resembles the condition in the cheek teeth of thyreophoran ornithischians. A similar condition is present in *Tianyulong*. The apical edge of the cingulum merges with the crowns face in *Echinodon* and most other heterodontosaurids.

Computed-tomographic scans show active tooth replacement in *Fruitadens* (Butler et al. 2010: Figs 2f, 3d, 3a-c; Butler et al. 2012: Figs 3, 4). Wear facets from tooth-to-tooth occlusion, however, have not been identified. The presence or absence of wear facets is difficult to determine with so few available crowns. Some of these specimens, in addition, are from younger individuals, which may not show wear typical of adults. The low-angle wear facets in *Echinodon* are less obvious and sometimes absent on newly erupted crowns.

#### Tianyulong confuciusi

Zheng et al., 2009

http://species-id.net/wiki/Tianyulong_confuciusi

Figs 9C 20 [Fig F21] [Fig F22] [Fig F23] [Fig F24] [Fig F25] [Fig F26] [Fig F27] [Fig F28] [Fig F29] 30 Tables 1 3 [Table T4] 5

Tianyulong confuciusi Zheng et al. (2009, Figs 1, 2)

##### Holotype.

STMN 26-3, partial skeleton laying on its left side preserving most of the skull in left lateral view, the ventral portion of a skull and articulated skeleton lacking the mid and distal caudal vertebrae, right coracoid, left carpus, portions of the left manus, and portions of the right hindlimb (Table 3; Zheng et al. 2009).

##### Referred material.

**Figure 20.**
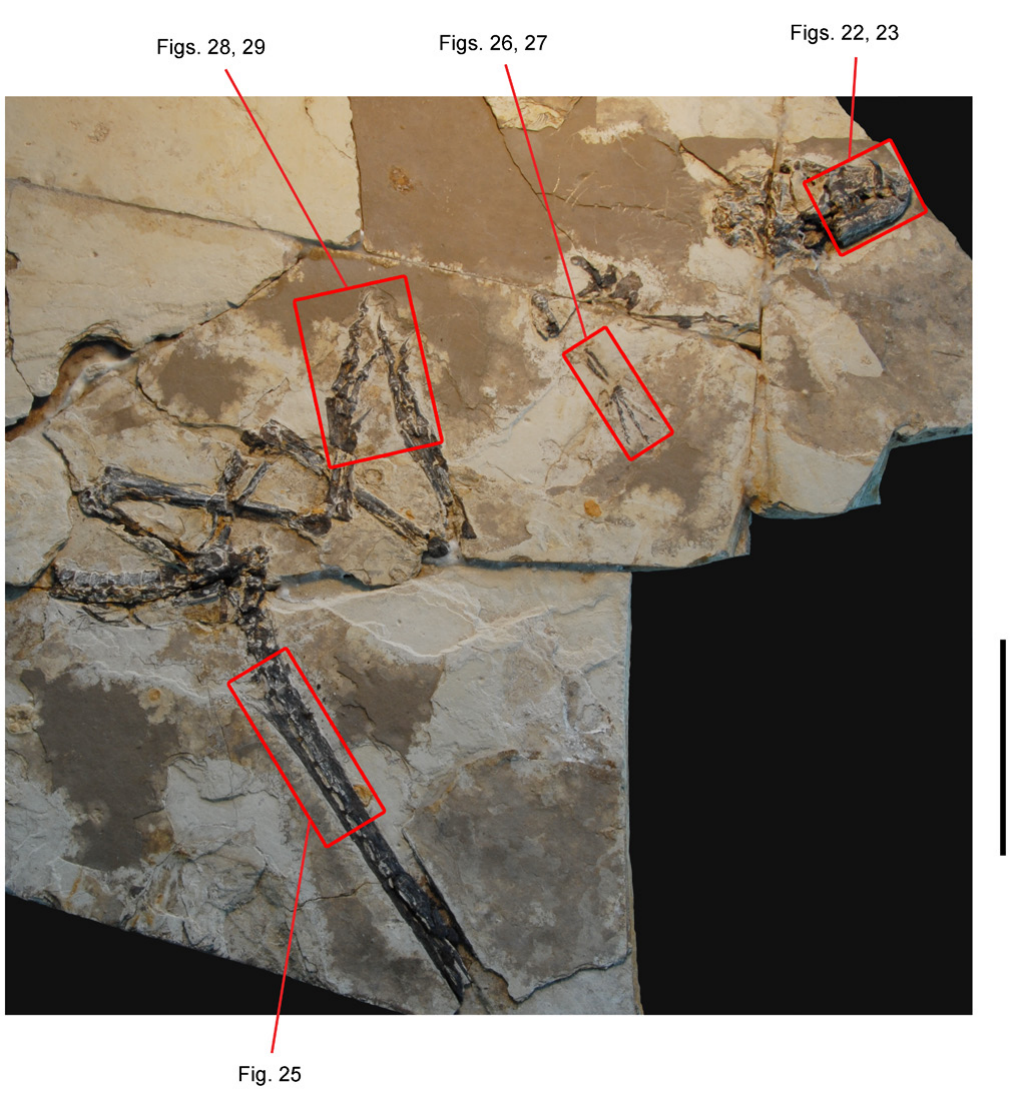
Partial skeleton of the heterodontosaurid *Tianyulong confuciusi* from the Lower Cretaceous Jehol Group of China. Partial skeleton mainly in right lateral view (IVPP V17090). Enlargements of subsequent figures are shown in red. Scale bar equals 10 cm.

IVPP V17090 (Fig. 20), partial skeleton laying on its left side preserving a nearly complete skull, an articulated portion of the column including the posterior dorsal vertebrae, sacral vertebrae, and proximal one-half of the tail with numerous, aligned epaxial and hypaxial ossified tendons, proximal portions of both scapulae, both coracoids, most of both forelimbs including an articulated left carpus and manus in ventral view, and both hindlimbs with right pes mostly in ventral view and left pes mostly in dorsal view (Tables 4, 5).

##### Type locality.

Jianchang County, Liaoning Province, PRC; collected privately but localities are probably in the vicinity N41°20', E119°40'.

##### Horizon.

Probably from the Lujiatun Beds of the Yixian Formation, Jehol Group; Lower Cretaceous (Barremian-Aptian), ca. 125 Ma (Xu and Norell 2006; Gradstein and Ogg 2009).

##### Revised diagnosis.

Heterodontosaurid with (1) only two premaxillary teeth, (2) rectangular dentary ramus with parallel dorsal and ventral margins, (3) extremely reduced forelimb that is less than 30% the length of the hindlimb, (4) manual digit III and metacarpal 3 shorter than manual digit II and metacarpal 2, respectively, (5) tail increased in length, (6) subtriangular chevrons in mid caudal vertebrae, and (7) numerous parallel ossified epaxial and hypaxial ossified tendons in the mid and distal regions of the tail.

##### Comments.

The holotype is a mature skeleton as evidenced by fusion of sacral centra, fusion or tight articulation of the neural arch and centrum of all other preserved vertebrae, and fusion between the tibia and proximal tarsals and between the bases of the metatarsals. The stratigraphic origin and geological age of *Tianyulong* remain uncertain, because all currently known specimens were collected privately (X. Xu, pers. comm.). The initial description of *Tianyulong* only reported a general area (“Jianchang County, Liaoning Province”) and horizon (“Jehol Group, Early Cretaceous”) (Zheng et al. 2009: 333). Upper Jurassic formations underlying the Jehol Group also have yielded specimens with integument preservation and a similar taphonomic attributes (Hu et al. 2009), and so the assignment of *Tianyulong* to the Yixian Formation with a Lower Cretaceous (Barremian-Aptian) age awaits confirmation.

##### Description.

The following brief description is based on two skeletons, the holotype (STMN 26-3; Fig. 9C) and a referred specimen (IVPP V17090; 20–30). Four additional skeletons of varying completeness are catalogued in the collections of the Shandong Tianyu Museum of Nature. Further preparation and study of all of these specimens of *Tianyulong* is needed before attempting a reliable skull reconstruction. The present description focuses on the most important features, culminating in a skeletal reconstruction (Fig. 30) and a set of comparative measurements (Tables 3–5).

##### Cranium.

**Figure 21. F21:**
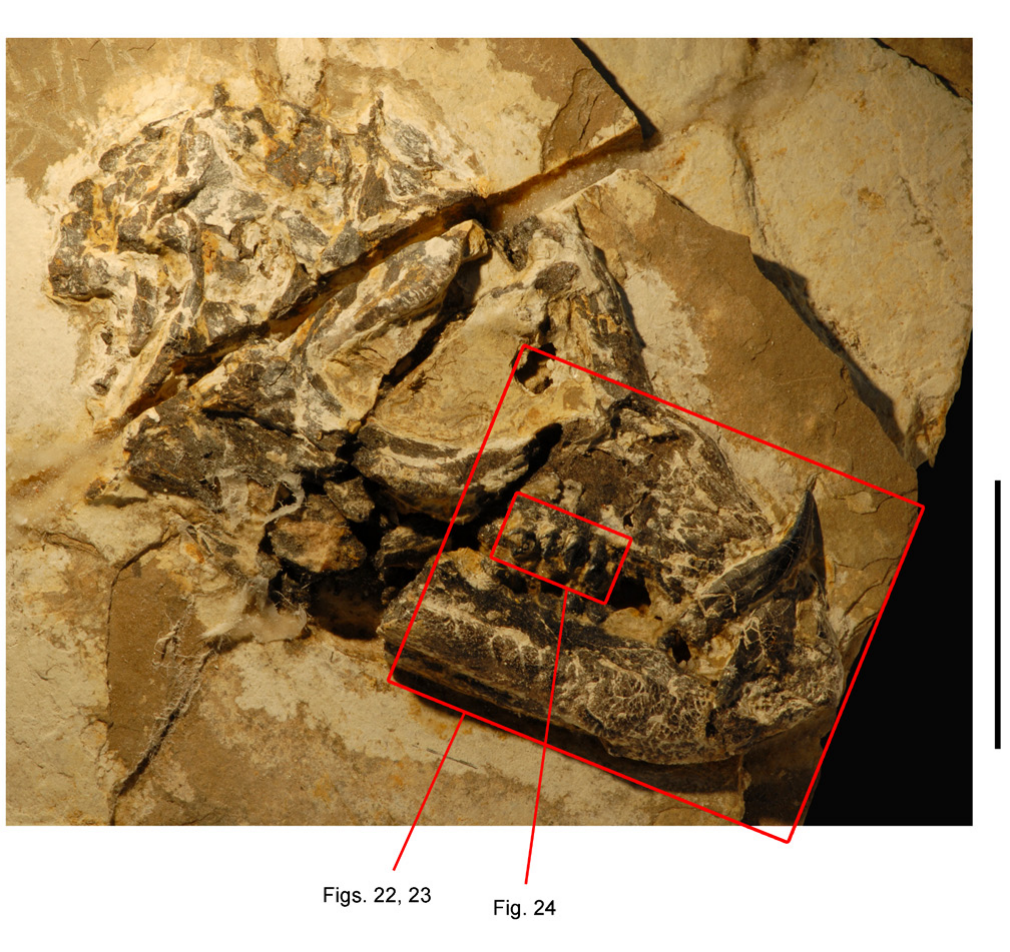
Skull of the heterodontosaurid *Tianyulong confuciusi* from the Lower Cretaceous Jehol Group of China. Skull in right lateral view (IVPP V17090). Enlargements of subsequent figures are shown in red. Scale bar equals 2 cm.

**Figure 22. F22:**
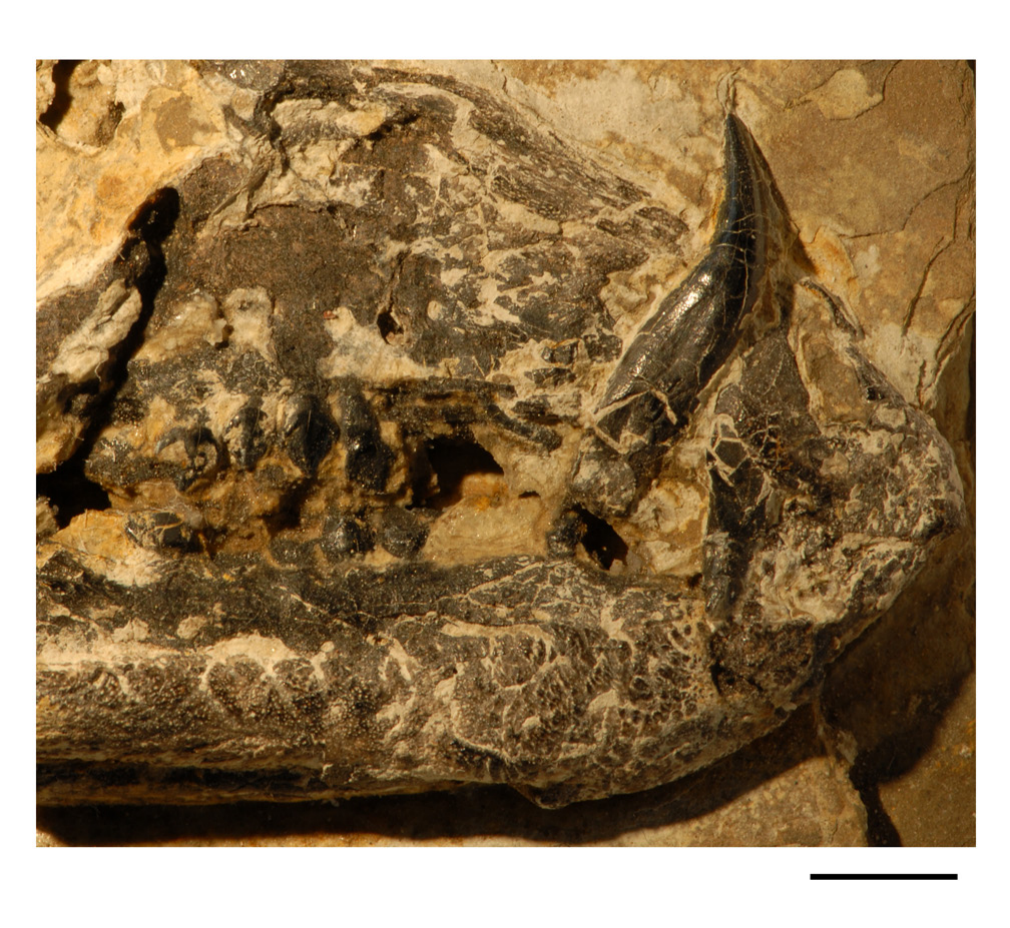
Anterior portion of skull of the heterodontosaurid *Tianyulong confuciusi* from the Lower Cretaceous Jehol Group of China. Snout in right lateral view (IVPP V17090). Scale bar equals 5 mm.

**Figure 23. F23:**
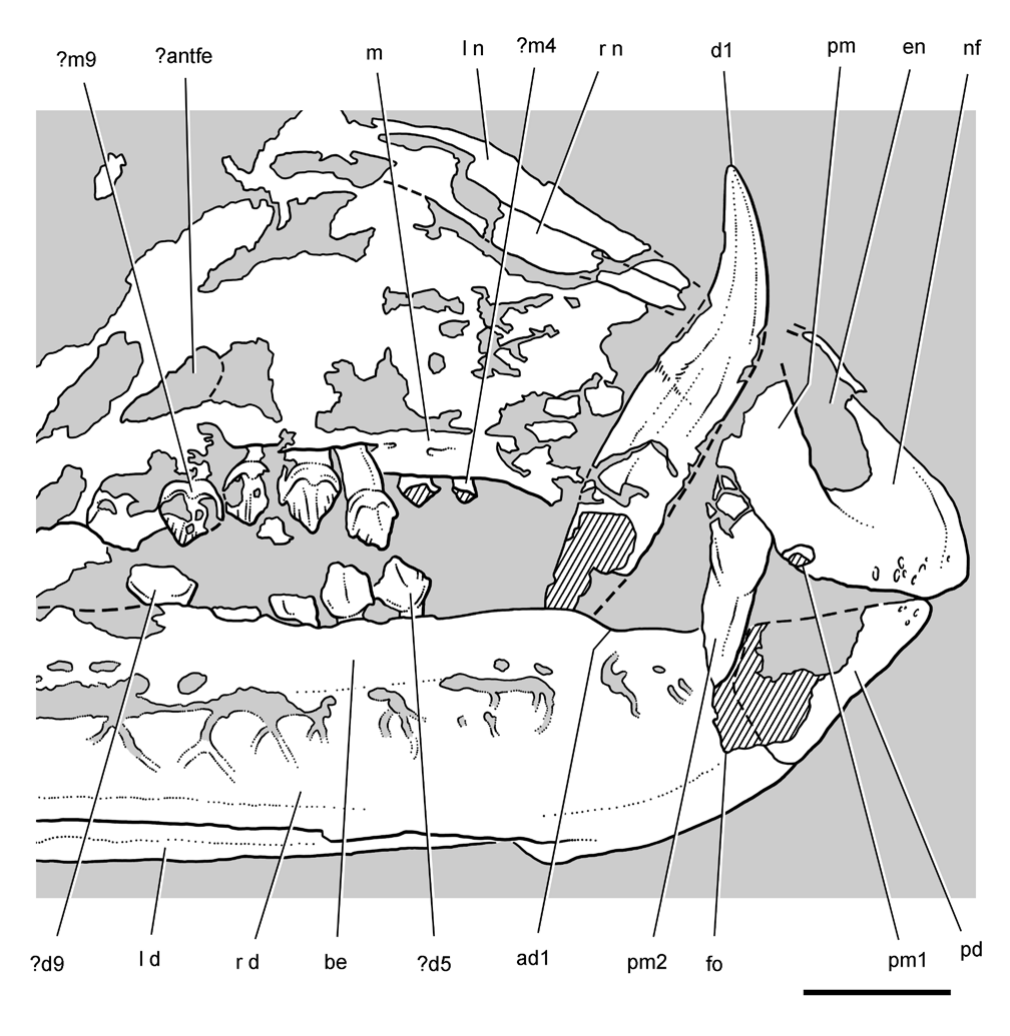
Anterior portion of skull of the heterodontosaurid *Tianyulong confuciusi* from the Lower Cretaceous Jehol Group of China. Snout in right lateral view (IVPP V17090). Hatching indicates broken bone; dashed lines indicate estimated edges; tone indicates matrix. Scale bar equals 5 mm. Abbreviations: ***ad1*** alveolus of dentary tooth 1 ***antfe*** antorbital fenestra ***be*** buccal emargination ***d*** dentary ***d1***, ***5***, ***9*** dentary tooth 1, 5, 9 ***en*** external naris ***fo*** foramen ***l*** left ***m*** maxilla ***m4***, ***9*** maxillary tooth 4, 9 ***n*** nasal ***nf*** narial fossa ***pd*** predentary ***pm*** premaxilla ***pm1***, ***2*** premaxillary tooth 1, 2 ***r*** right.

The cranium is well represented in the holotype and referred specimens, although several portions remain poorly understood (Figs 20–23). Little is known of the posterodorsal portion of the cranium, which is broken away in the holotype (STMN 26-3; Zheng et al. 2009; Fig. 9C) and disarticulated and only partially prepared in the referred specimen (Fig. 21). Likewise, virtually nothing is known about the palate and braincase. The shape of the skull is similar to that in *Heterodontosaurus*; both are subtriangular in lateral view with a gently concave roof over the antorbital region. Both crania also are preserved in a similar manner, with jaws in occlusion and the premaxilla and predentary in close approximation (Fig. 21).

In both skulls the alveolar margin of the premaxilla is tilted slightly anteroventrally and is positioned ventral to the maxillary tooth row (Figs 22, 23). A horizontal line along the maxillary crowns at mid height passes above the premaxillary alveolar margin and closer to the ventral margin of the external naris. Approximately 60% of the length of the premaxillary alveolar margin is edentulous, the posterior 40% of its length accommodating two premaxillary teeth. In both skulls the posterior end of the alveolar margin curves posterodorsally, exposing a portion of the root of the caniniform second premaxillary tooth. Dorsal to this root, the premaxilla forms the anterior portion of an inset, arched diastema for the large dentary caniniform tooth. The narial fossa extends ventrally near the alveolar margin, and the external naris is dorsoventrally elongate as in *Heterodontosaurus*. The posterolateral process of the premaxilla also is very similar in shape and articular contacts to that in *Heterodontosaurus*. This process expands in width above the caniniform tooth and then tapers to a narrow tip, which in the holotype appears to establish point contact with the anterior tip of the lacrimal (STMN 26-3; Zheng et al. 2009).

The subtriangular maxilla forms the posterior portion of the inset, arched diastema (Fig. 9C). Most of the lateral aspect of the maxilla is occupied by the subtriangular antorbital fossa, which is bordered ventrally by a sharp, slightly arched rim. The buccal emargination ventral to this rim is narrow compared to that in *Echinodon* and *Lycorhinus*. The jugal appears to lack the horn and flange that characterizes *Heterodontosaurus* and *Manidens*.

##### Lower jaw.

The predentary is a small, wedge-shaped bone that lacks discrete processes (Figs 22, 23). The predentary had been shown with a long ventral process (Zheng et al. 2009: fig. 1d; Fig. 9C), but a portion of this process is actually the ventral margin of the dentary. All but the posterodorsal corner of the predentary is positioned anterior to the premaxillary caniniform tooth.

The dentary ramus is straight and parallel-sided for most of its length (Fig. 21) in contrast to most heterodontosaurids, which exhibit a posterior deepening of the ramus. At its anterior end, a ventral protuberance is present in both the holotypic and referred skulls (Figs 22, 23). Anterior to the protuberance, the dentary end is strongly beveled as in *Echinodon* (Fig. 17). The ventral rim of the well developed buccal emargination is marked by diverging impressed vessel tracts (Figs 22, 23).

The sutures between the postdentary bones are poorly preserved in the two available specimens. The coronoid process rises well above the level of the dentary crowns as in *Heterodontosaurus*, but the jaw articulation is not dropped relative to the occlusal plane (Figs 9C, 21). As best seen in the holotypic skull, a line drawn through the zone of occlusion between the cheek tooth rows passes just ventral to the jaw articulation (Fig. 9C). The coronoid process of the dentary was shown as a deep ramus rather than a more slender process (Zheng et al. 2009), an interpretation that may have incorporated portions of the adjacent surangular (Fig. 9C). An external mandibular fenestra appears to have been present (Zheng et al. 2009). There is no evidence for the presence of an external mandibular fossa around the fenestra or for an incised vessel tract to a large anterior surangular foramen, both of which characterize *Lycorhinus*, *Manidens* and *Heterodontosaurus*.

##### Premaxillary teeth.

There are two premaxillary teeth, the first a small tooth known only from its broken base and the second a large caniniform tooth (Figs 22, 23). The caniniform premaxillary tooth, the base of which is better preserved in the holotype skull, has a gentle posterior recurvature. Mesial and distal carinae are present, at least the latter with serrations. Only the larger distal premaxillary tooth was shown in the initial drawing of the holotypic skull (Fig. 9C). As in all heterodontosaurids and neornithischians, there is a substantial edentulous border preceding the first premaxillary tooth.

##### Dentary teeth.

The total number of dentary teeth in *Tianyulong* cannot be established with certainty based on the holotype, although referred skulls suggest there are 10 dentary teeth. An enlarged caniniform is the first tooth in the dentary series; no trace of a leading rudimentary crown is present. The dentary caniniform tooth is followed by a postcaniniform crown without a significant intervening gap, similar to the condition in *Echinodon* and *Fruitadens*. The first postcaniniform tooth in the holotypic skull can be seen in the photographs slightly dislodged toward the caniniform tooth (Zheng et al. 2009: fig. 1e). It was omitted in a drawing of the holotypic skull (Fig. 9C), leaving the impression that a postcaniniform diastema may be present in *Tianyulong*. The first dentary tooth has a subconical crown that is slightly smaller than that of more distal dentary teeth. This differs from the substantially smaller tooth immediately distal to the caniniform tooth in *Fruitadens* (Fig. 9A). A referred specimen in the Shandong Tianyu Museum of Nature collections confirms the morphology, size and position of the first postcaniniform dentary tooth.

Successive dentary crowns 3-10 become slightly larger in the holotypic and referred skulls. In these postcaniniform dentary teeth, the bulbous cingulum has well-defined margins, a cingular ectoloph raised above the remainder of the crown surface including the primary ridge. The cingulum curves apically, terminating mesially and distally in prominent apically projecting denticles. Toward the posterior end of the dentary series, the prominence of the apical, mesial and distal basal denticles gives the crown a tricuspid appearance. The penultimate tooth, dentary tooth 9, is the largest in the series. The most distal tooth, dentary tooth 10, has a considerably smaller crown, as seen in several referred skulls.

##### Maxillary teeth.

The total number of maxillary teeth is currently unknown given the available evidence in the holotypic and referred skulls. In the right maxillary series of the holotypic skull, the crowns of the smallest teeth just behind the caniniform dentary tooth are broken at their bases (Fig. 9C). This pair is followed by three crowns, a gap for a missing sixth maxillary tooth, and a final set of three teeth, all of which have crowns that are progressively larger in size (Zheng et al. 2009: fig. 1d). Probably at least two additional teeth with progressively smaller crowns were present at the distal end of the maxillary series. Taking these two into account, a total of 11 maxillary teeth likely were present in the holotypic skull.

**Figure 24. F24:**
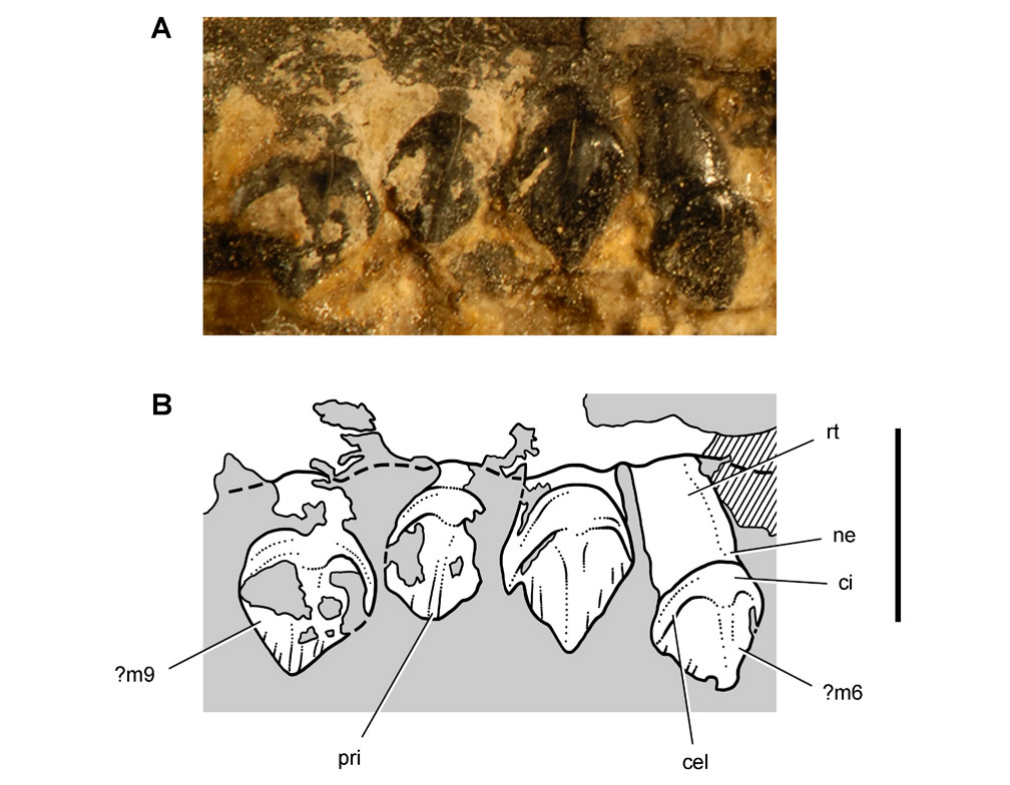
Maxillary dentition of the heterodontosaurid *Tianyulong confuciusi* from the Lower Cretaceous Jehol Group of China. Right maxillary teeth ?6-9 in lateral view (IVPP V17090). Hatching indicates broken bone; dashed lines indicate estimated edges; tone indicates matrix. Scale bar equals 3 mm. Abbreviations: ***cel*** cingular ectoloph ***ci*** cingulum ***m6*** ***9*** maxillary tooth 6 9 ***ne*** neck ***pri*** primary ridge ***rt*** root.

Maxillary and dentary crowns are subtriangular, the dentary crowns somewhat deeper than opposing maxillary crowns as in *Echinodon*. All have similar features in labial view (Fig. 24). The bulbous cingulum is well-defined from the remainder of the crown surface as in the largest crowns in *Fruitadens*, and the root is tapered rather than swollen. The cingulum curves apically as a prominent cingular ectoloph along the mesial and distal crowns edges, terminating in prominent basal denticles mesially and distally. Arching of the alveolar margins is not pronounced as in *Abrictosaurus* and *Heterodontosaurus*. The dentary alveolar margin is straight; the opposing maxillary margin is gently arched in both holotypic and referred skulls (Figs 22, 23).

##### Axial skeleton.

Theholotype preserves the posterior one-half of the cervical series (C5-9), and much of the dorsal column is preserved in the referred skeleton. Centrum length is nearly constant, measuring approximately 5 mm (Fig. 30; Table 4). In *Heterodontosaurus*, in contrast, centrum length decreases in posterior cervical vertebrae by approximately 20% (Fig. 72, Table 7). Posterior dorsal centra have deeply concave sides and join as a gently arched series (Fig. 20). Their hatchet-shaped neural spines are longer than deep and nearly touch at their anterior and posterior extremities (IVPP V17090). In *Heterodontosaurus*, in contrast, the neural spines of the posterior dorsal vertebrae are deeper than long and well separated (Fig. 72). The sacral vertebrae are fused in IVPP V17090, and it is possible to measure only the centrum length of S1 (Table 4).

**Figure 25. F25:**
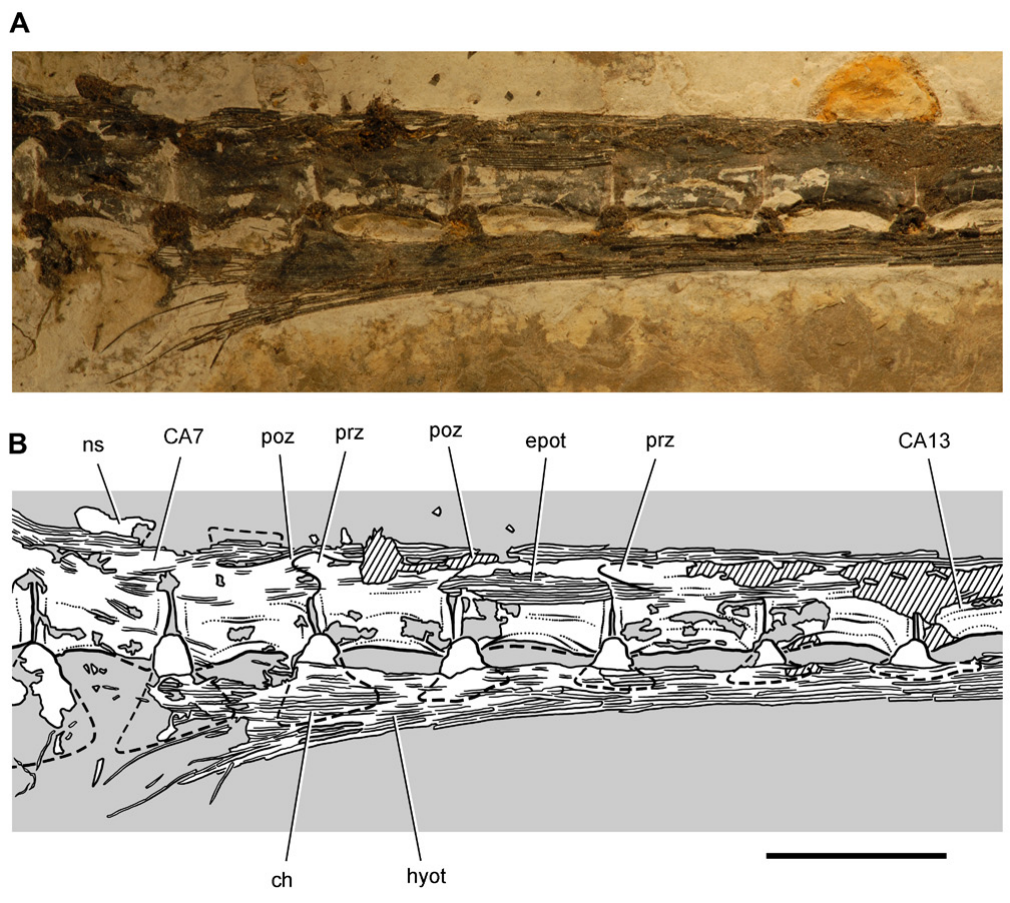
Tail of the heterodontosaurid *Tianyulong confuciusi* from the Lower Cretaceous Jehol Group of China. Anterior caudal vertebrae and ossified tendons in left lateral view, from the seventh to the thirteenth caudal vertebra. Hatching indicates broken bone; dashed lines indicate estimated edges; tone indicates matrix. Scale bar equals 1 cm. Abbreviations: ***CA*** caudal ***ch*** chevron ***epot*** epaxial ossified tendons ***hyot*** hypaxial ossified tendons ***ns*** neural spine ***poz*** postzygapophyses ***prz*** prezygapohpysis.

The proximal one-half of the caudal series is preserved in the holotypic skeleton and nearly as much in the referred skeleton (Fig. 25). The tail is very long, exceeding the length of the precaudal column by the twentieth caudal vertebra (Fig. 30). Caudal centra increase in length by approximately 40% from the first to the tenth caudal vertebra. By the seventh caudal vertebra, the neural arch is low and hatchet-shaped and the opposing chevrons subtriangular rather than strap-shaped (Fig. 25). At this point in the tail, a sheath of parallel ossified tendons are present spanning the neural spines and chevrons. By the thirteenth caudal vertebra, the neural spines are reduced to a ridge, and the chevrons are boat-shaped (Fig. 25). Although a few epaxial ossified tendons are present near the neural arches of the posterior dorsal, sacral and anterior caudal vertebrae (IVPP V17090), the sheath of ossified tendons starting around the seventh caudal vertebra would have stiffened mid and distal portions of the tail. The caudal structure in *Tianyulong* is remarkably similar to that in dromaeosaurid theropods (Ostrom 1969), the neural spines and chevrons changing in a similar manner distally and the parallel sheath of ossified tendons equivalent to the parallel, attenuated processes of the prezygapophyses and chevrons. Probably the more mobile tail base and stiffened mid and distal tail functioned in a similar manner as a balancing beam to enhance maneuverability.

##### Pectoral girdle.

Both scapulocoracoids are preserved in opposition in the referred skeleton (Fig. 20; IVPP V17090). The right forelimb is disarticulated dorsally, the humerus displaced from its articulation in the glenoid. The proximal end of the right humerus is exposed, the remainder of the bone damaged or embedded as it crosses a crack in the slab to a small corner of bone, the medial epicondyle. The right humerus has an estimated length of 28 mm. The left humerus, with an estimated length of 26 mm, lies in articulation with the glenoid of the scapulocoracoid proximally and the remainder of the left forelimb distally. The holotypic skeleton preserves both scapulocoracoids and humeri but little of the distal forelimb (Zheng et al. 2009: suppl. info.). Overlapping limb bone measurements suggest that the holotypic skeleton (STMN 26-3) is approximately 15% larger than the referred skeleton (IVPP V17090; Table 3).

Proximally, the scapula broadens gradually to the acromial process as in *Heterodontosaurus* (Santa Luca 1984). The neck is narrow as is most of the slender, straight strap-shaped blade (STMN 26-3; Zheng et al. 2009: suppl. info.). The distal end of the blade is not well exposed in either specimen. Expansion in width of the distal end of the blade, if present, would be proportionately less than in *Heterodontosaurus*. The coracoid has a subquadrate body and a prominent hook-shaped posterior process (STMN 26-3).

##### Forelimb.

**Figure 26. F26:**
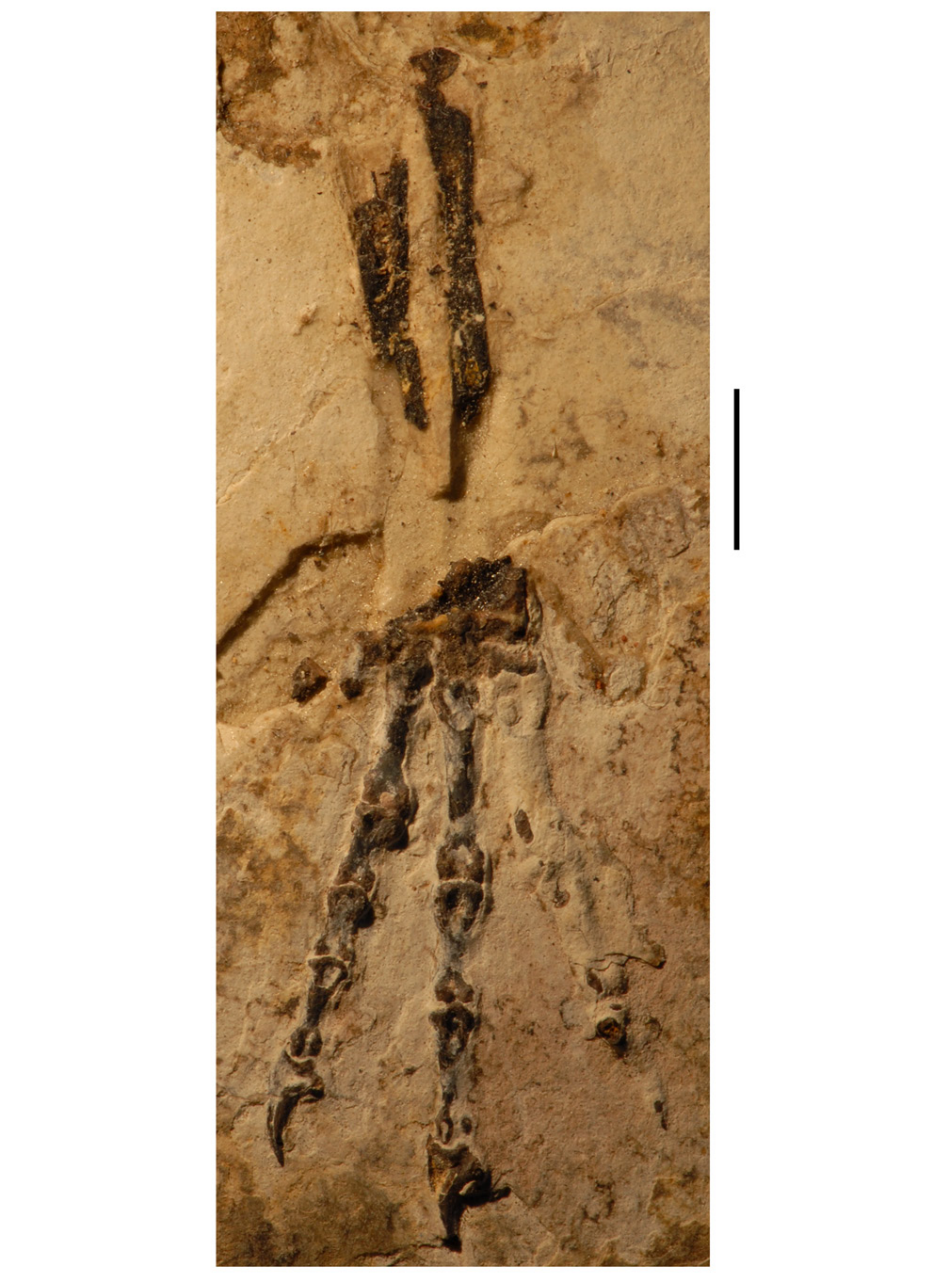
Forearm and manus of the heterodontosaurid *Tianyulong confuciusi* from the Lower Cretaceous Jehol Group of China. Left ulna, radius and manus for the most part in anterior or ventral view. Scale bar equals 5 mm.

**Figure 27. F27:**
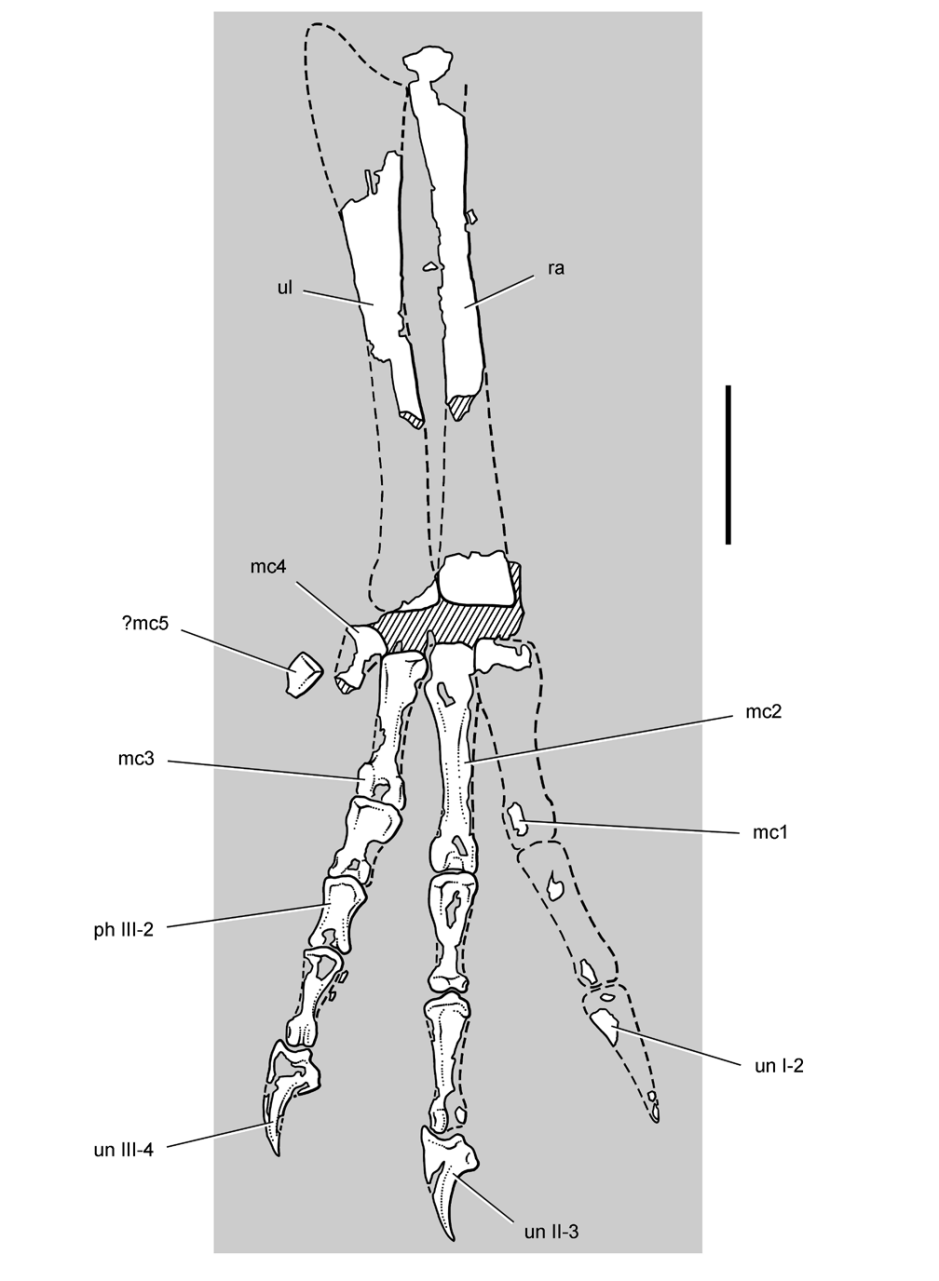
Forearm and manus of the heterodontosaurid *Tianyulong confuciusi* from the Lower Cretaceous Jehol Group of China. Left ulna, radius and manus for the most part in anterior or ventral view. Hatching indicates broken bone; dashed lines indicate estimated edges; tone indicates matrix. Scale bar equals 5 mm. Abbreviations: ***I-III*** digits I-III ***mc1-5*** metacarpal 1-5 ***ph*** phalanx ***ra*** radius ***ul*** ulna ***un*** ungual.

The humerus, which is best preserved in STMN 26-3, has a bulbous head, prominent deltopectoral crest, gently curved shaft and prominent distal condyles as in *Heterodontosaurus*. The olecranon process of the ulna, also best exposed in STMN 26-3, is very prominent proximally as in *Heterodontosaurus*. The radius tapers at mid-shaft and expands distally as in *Heterodontosaurus* to broadly contact a well ossified, but poorly preserved, carpus (Figs 26, 27).

The manus probably retained all five digits as in *Heterodontosaurus*, although only fragments of digits IV and V remain (Figs 26, 27; Table 5; IVPP V17090). The proportions of the manual digits are most unusual among ornithischians including *Heterodontosaurus*. Digit II and metacarpal 2 are longer than digit III and metacarpal 3. This mismatch in length is due to the shortening of all bones in digit III, as metacarpal 1 is also longer than metacarpal 3 (Figs 26, 27). The block-shaped bases and divided distal condyles of metacarpals 1-3 are well exposed. Metacarpal 1 has a particularly broad base as in *Heterodontosaurus*. The phalangeal formula 2-3-4-?-? is typical for the inner three digits of basal dinosaurs and archosaurs in general.

The two phalanges of manual digit I diverge medially from the others. The nonungual phalanges have proximal intercondylar processes articulating between paired distal condyles with well-formed collateral ligament pits as in *Heterodontosaurus*. The penultimate phalanges in digits II and III, in addition, are longer than the preceding phalanges, suggesting an enhanced grasping function for the manus in *Tianyulong* as in *Heterodontosaurus*. The unguals are transversely compressed and trenchant, that on digit I longer than those on digits II and III (Figs 26, 27).

##### Pelvic girdle.

Little can be said about the ilium, which is poorly preserved in both skeletons. The ischia and pubes are preserved in lateral view (STMN 26-3; Zheng et al. 2009: suppl. Info). The ischial shaft is gently dorsally bowed, a curve not present in *Heterodontosaurus*. The ischial shaft is transversely compressed and lacks any flanges or processes, including the lateral flange of *Heterodontosaurus* (Santa Luca 1984) or the obturator process present in many ornithischians.

Of the pubis, only the postpubic process is preserved (STMN 26-3; Zheng et al. 2009). Its shaft is rod-shaped and very slender as in *Heterodontosaurus* but only one-half its length. The process tapers to a slender tip just beyond the midpoint of the ischial shaft.

##### Hindlimb.

The femoral head is large and round, and the femoral shaft is robust with a proximally placed pendant fourth trochanter (IVPP V17090). Whether the anterior trochanter is separate as in the Kayenta heterodontosaurid and *Abrictosaurus* or fused with the greater trochanter as in *Fruitadens* cannot be determined. The tibia is extremely elongate, measuring more than 140% the length of the femur (Table 3). The cnemial crest is lower than in *Heterodontosaurus*, so that in medial view the anterior margin of the proximal end of the tibia is straight rather than convex (Fig. 20). The posterior condyles expand farther away from the central axis of the shaft. A fibular crest on the tibia supports the proximal end of the fibula, which has a swollen anterior trochanter (STMN 26-3; Zheng et al. 2009: suppl. info.). Distally, the fibula tapers to a slender rod. The tibia, fibula and proximal tarsals appear fused as a tibiotarsus as in most specimens of *Heterodontosaurus*. The distal tarsals are dislodged on one side of the referred skeleton and thus may not have coossified with the metatarsus.

**Figure 28. F28:**
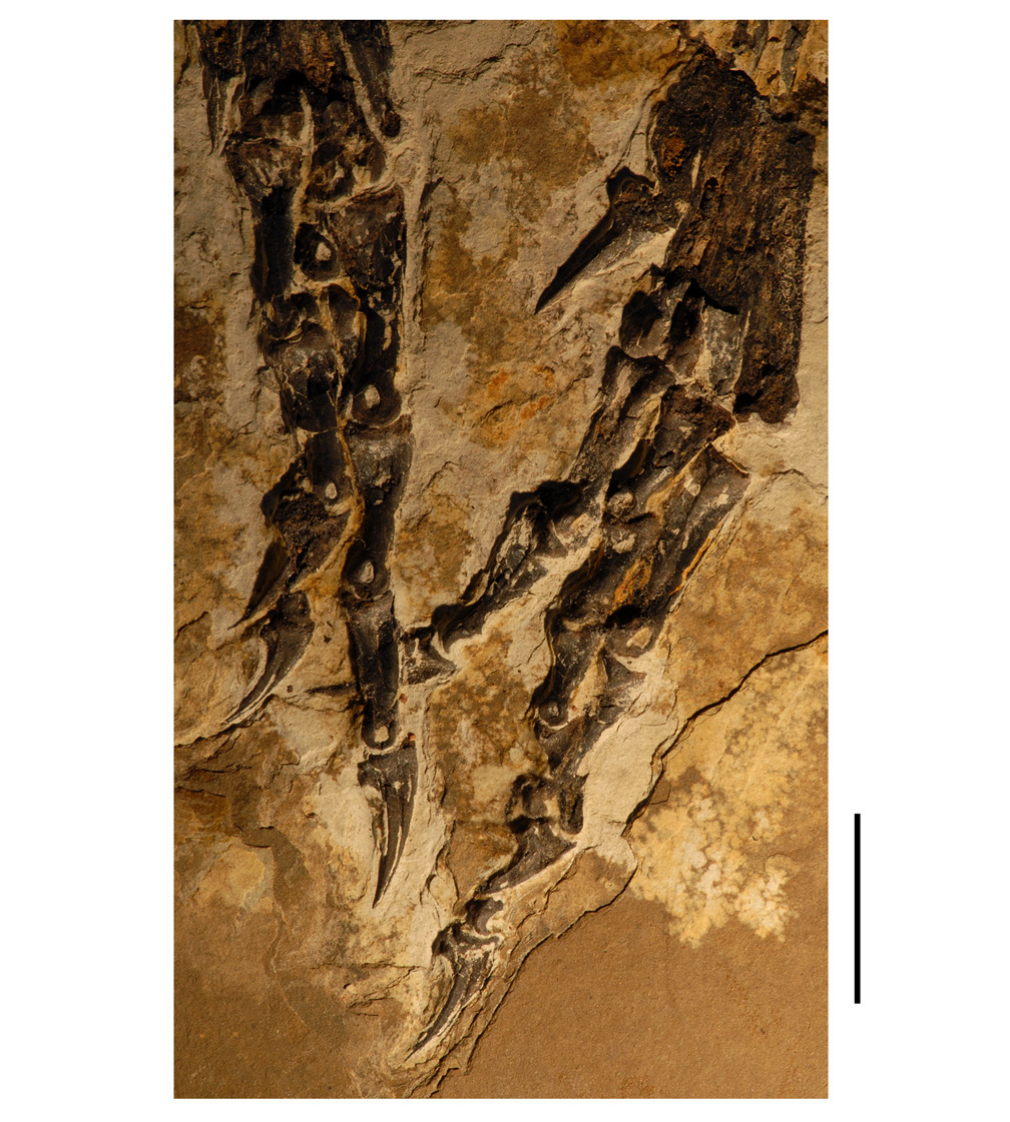
Pes of the heterodontosaurid *Tianyulong confuciusi* from the Lower Cretaceous Jehol Group of China. Left pedal phalanges (left) in dorsal and medial views; right pedal phalanges (right) in ventral and lateral views. Scale bar equals 1 cm.

**Figure 29. F29:**
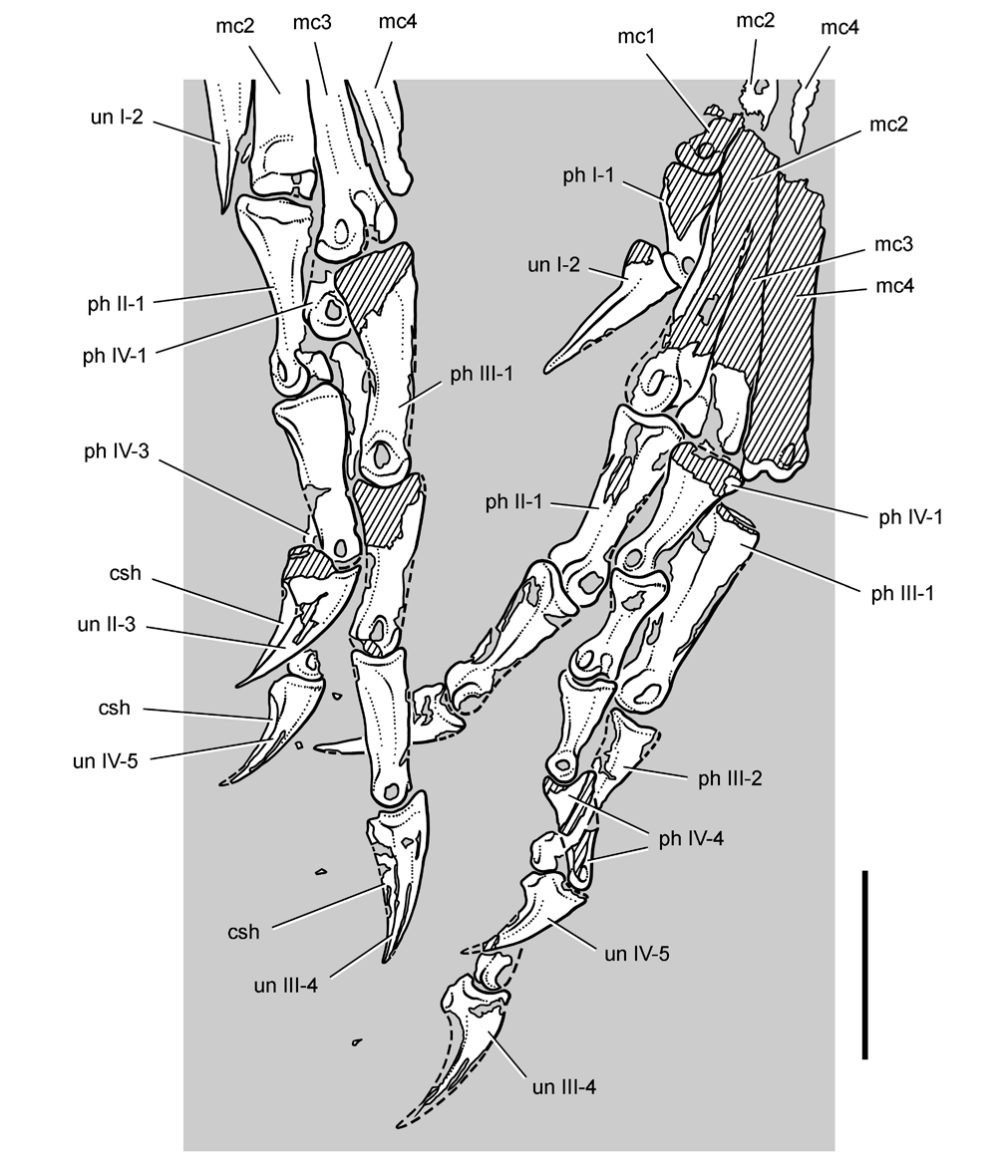
Pes of the heterodontosaurid *Tianyulong confuciusi* from the Lower Cretaceous Jehol Group of China. Left pedal phalanges (left) in dorsal and medial views; right pedal phalanges (right) in ventral and lateral views. Hatching indicates broken bone; dashed lines indicate estimated edges; tone indicates matrix. Scale bar equals 1 cm. Abbreviations: ***I-IV*** digits I-IV ***csh*** claw sheath ***mc1-4*** metacarpal 1-4 ***ph*** phalanx ***un*** ungual.

In the pes, the proximal ends of metatarsal 1-4 appear to be coossified. Pedal digit I is very short, the tip of its ungual extending just beyond the condyles of metatarsal 2 (Figs 28, 29). That ungual, however, is quite large, equaling the length of the ungual on digit II and exceeding the length of the ungual on digit IV. Pedal digit III, in contrast, is slightly longer relative to pedal digits II and IV, a proportion that fits with other cursorial adaptations of the hindlimb. There is no trace of pedal digit V, which may owe its absence in *Tianyulong* to postmortem loss. The unguals are transversely compressed and taper to slender tips (Table 5). Several of the unguals preserve portions of the keratinous claw sheath (Figs 28, 29).

##### Skeletal reconstruction.

**Figure 30.**
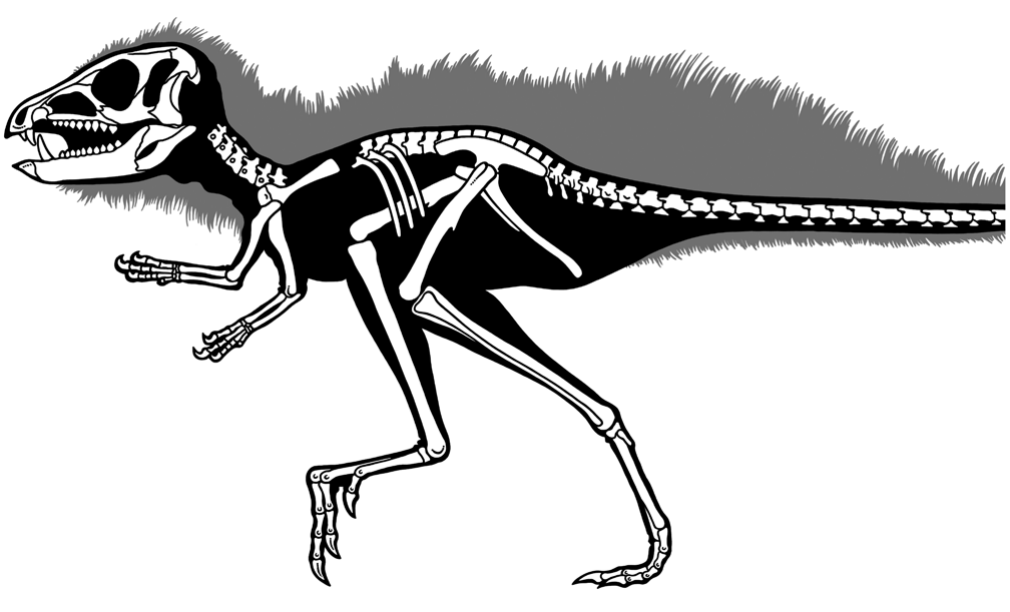
Skeleton of the heterodontosaurid *Tianyulong confuciusi* from the Lower Cretaceous Jehol Group of China. Silhouette skeletal reconstruction in lateral view showing preserved bones and integumental fibers (based on IVPP V17090). Distal most caudal vertebrae unknown.

*Tianyulong* has very unusual skeletal proportions that differ from those in *Heterodontosaurus* and, to the extent that comparisons are possible, other heterodontosaurids (Figs 30, 72; Tables 3–9). The skull is proportionately very large and the hindlimbs are very long, whereas the neck and trunk are proportionately small and the forelimbs are reduced in length. Using *Archaeopteryx* for initial comparison (Wellnhofer 1993), the skull of *Tianyulong* is 25% longer than in the Eichstätt specimen, which has a nearly identical femoral length (Table 5). The tibiofemoral ratio in this specimen of *Archaeopteryx* is high (140%), although still slightly lower than in *Tianyulong* (143%). The humerus, in contrast, is approximately 48% the length of that in *Archaeopteryx*. As discussed further below (see Discussion, Body size and proportions), these unusual proportions give this small-bodied heterodontosaurid the appearance of a large-headed, short-armed, long-legged dwarf (Fig. 30) with very different proportions than in *Heterodontosaurus* (Fig. 72).

**Table 4. T4:** Measurements (mm) of the cranium and axial skeleton of the Early Cretaceous heterodontosaurid *Tianyulong confuciusi* (IVPP V17090). Parentheses indicate estimated measurement. Identification of dorsal vertebral number based on the vertebral count in *Heterodontosaurus*.;

**Region**	**Measurement**	
Cranium	Length, premaxilla to posterior edge of squamosal	65
	Preorbital length, anterior rim of orbit to tip of premaxilla	30
	Occiput height, quadrate condyle to parietal occipital flange	(23)
	Antorbital fossa maximum length	15
	Antorbital fossa maximum height	9
	Quadrate height	(15)
Dorsal Vertebrae	D7 centrum length	5
	D8 centrum length	5
	D9 centrum length	5
	D10 centrum length	5
	D11 centrum length	5
	D12 centrum length	5
Sacral Vertebrae	S1 centrum length	5
Caudal Vertebrae	CA1 centrum length	—
	CA2 centrum length	5
	CA3 centrum length	5
	CA4 centrum length	6
	CA5 centrum length	6
	CA6 centrum length	6
	CA7 centrum length	7
	CA8 centrum length	7
	CA9 centrum length	8
	CA10 centrum length	8
	CA11 centrum length	8
	CA12 centrum length	8
	CA13 centrum length	8

**Table 5.** Measurements (mm) of the girdles and limb bones of the Early Cretaceous heterodontosaurid *Tianyulong confuciusi* (IVPP V17090). Measurements are from the right side for paired bones except as indicated. Parentheses indicate estimated measurement.

**Structure**	**Measurement**	
Pectoral girdle	Scapula length	(23)
	Scapular blade, minimum neck width	2
	Scapular blade, distal width	—
	Coracoid length	4
	Coracoid height	7
Proximal forelimb	Humerus length	(27)
	Radius + carpus + manus (radial head to tip of digit II)	38^1^
	Radius length	17
	Ulna length	(19)^1^
Manual digit I	Manual digit I length	16^1^
	Metacarpal 1 length	6^1^
	Phalanx I-1 length	5^1^
	Ungual I-2 length	4^1^
Manual digit II	Manual digit II length	21^1^
	Metacarpal 2 length	7^1^
	Phalanx II-1 length	3.5^1^
	Phalanx II-2 length	4^1^
	Ungual II-3 length	4^1^
Manual digit III	Manual digit III length	16^1^
	Metacarpal 3 length	5^1^
	Phalanx III-1 length	2.5^1^
	Phalanx III-2 length	2^1^
	Phalanx III-3 length	3^1^
	Ungual III-4 length	3^1^
Proximal hind limb	Femur length	51
	Tibiotarsus length	73
	Tibiotarsus proximal end, anteroposterior length	11
	Tibiotarsus mid shaft, anteroposterior diameter	5
Pedal digit I	Metatarsal 1 length	25^1^
	Phalanx I-1 length	6^1^
	Ungual I-2 length	9^1^
Pedal digit II	Metatarsal 2 length	(39)^1^
	Phalanx II-1 length	10.5^1^
	Phalanx II-2 length	9.5^1^
	Ungual II-3 length	9^1^
Pedal digit III	Metatarsal 3 length	(43)^1^
	Phalanx III-1 length	12^1^
	Phalanx III-2 length	9^1^
	Phalanx III-3 length	8^1^
	Ungual III-4 length	8^1^
Pedal digit IV	Metatarsal 4 length	(40)^1^
	Phalanx IV-1 length	8
	Phalanx IV-2 length	6
	Phalanx IV-3 length	5
	Phalanx IV-4 length	6
	Ungual IV-5 length	6

^1^Left.

#### Heterodontosaurinae

Kuhn, 1966

##### Diagnosis.

Small-bodied ornithischians with the following features that likely would constitute synapomorphies in phylogenetic context: (1) cheek tooth crowns that are taller than wide, (2) jaw joint set below the axis of occlusion between maxillary and dentary teeth.

##### Phylogenetic definition.

The most inclusive clade containing *Heterodontosaurus tucki* Crompton and Charig 1962 but not *Tianyulong confuciusi* Zheng et al. 2009, *Fruitadens haagarorum* Butler et al. 2010, *Echinodon becklesii* Owen 1861. This stem-based phylogenetic definition for this new heterodontosaurid subgroup includes *Heterodontosaurus tucki* and other derived heterodontosaurid species but specifically excludes basal species with less modified teeth that retain subtriangular crowns enveloped in symmetrical enamel.

##### Temporal and geographic range.

Earliest Jurassic (Hettangian-Sinemurian) to Middle Jurassic or earliest Late Jurassic (Aalenian-Callovian), ca. 197-165 Ma (Gradstein and Ogg 2009; Pol et al. 2011); distribution limited to southern localities including Argentina and southern Africa (Fig. 1B).

##### Comments.

Authorship of family group names is given to the author of the first name coined within the family group (ICZN 1999), which in this case is Heterodontosauridae Kuhn 1966.

#### Abrictosaurus consors

(Thulborn, 1974)

http://species-id.net/wiki/Abrictosaurus_consors

Figs 5A 31 [Fig F32] [Fig F33] [Fig F34] [Fig F35] [Fig F36] 37 Tables 1 [Table T2] 3 6

Abrictosaurus consors (Thulborn, 1974) – Thulborn (1974, Figs 2–4); Hopson (1975, fig. 3c, d); Galton (1986, fig. 16.6m); Smith (1997, fig. 3c, d); Norman et al. (2011, fig. 39A, B)

##### Holotype.

NHMUK RU B54, ventral portion of a skull and articulated skeleton lacking the mid and distal caudal vertebrae, right coracoid, left carpus, portions of the left manus, and portions of the right hindlimb (Table 1). The author and other researchers have examined fragmentary postcranial material of a second individual catalogued with the holotypic specimen before their transfer to the NHMUK collection. At present there is no evidence that this material pertains to *Abrictosaurus consors* (Norman et al. 2011: 236–237).

##### Type locality.

Stream-side exposure by the town Nosi (= “Noosi”), 8.2 km east of Whitehill, southern Lesotho; S30°03', E28°32' (Thulborn 1974; Kitching and Raath 1984) (Fig. 1B).

##### Horizon.

Top unit (dull red sandstone) of the Upper Elliot Formation; Lower Jurassic, Hettangian, ca. 202-197 Ma (Thulborn 1974; Knoll 2005; Gradstein and Ogg 2009).

##### Revised diagnosis.

Heterodontosaurid ornithischian characterized by the following autapomorphies: (1) premaxillary tooth 2 and 3 with tall, subcylindrical crowns, the latter possibly representing a reduced caniniform tooth; (2) dentary tooth 2 with subcylindrical crown, possibly representing a reduced caniniform tooth; (3) maxillary and dentary teeth with flat lateral and medial crown surfaces lacking discrete marginal or median ridges; and (4) maxillary crowns in the middle of the tooth row have deep parallel-sided crowns that do not expand in mesiodistal width toward their apex. Unlike other heterodontosaurids, there is no dentary or premaxillary caniniform teeth, although both the dentary and premaxillary have teeth with subcylindrical crowns that may be reduced caniniform teeth.

##### Comments.

**Figure 31.**
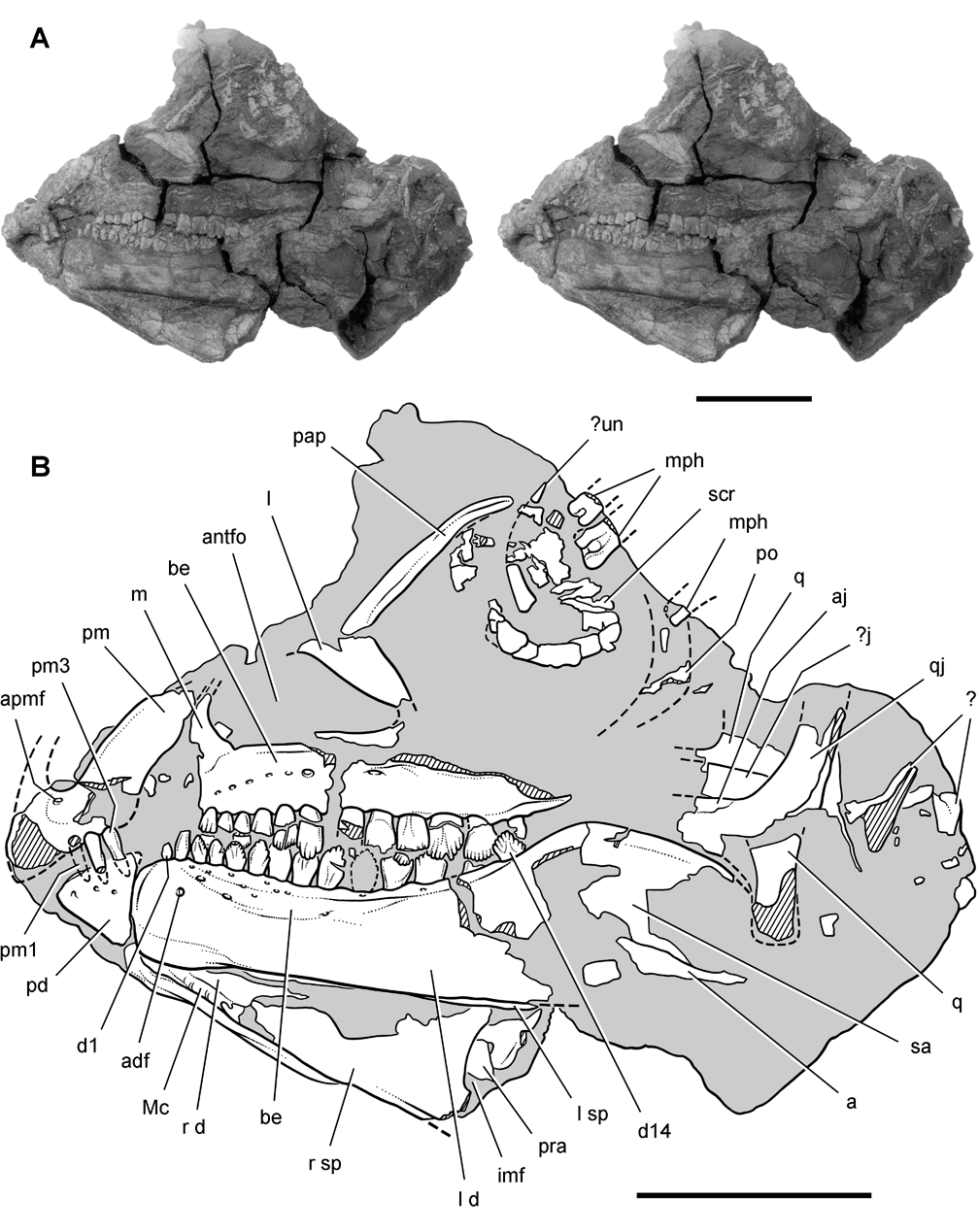
Skull of *Abrictosaurus consors* from the Lower Jurassic Upper Elliot Formation of South Africa. Stereopair (**A**) and line drawing (**B**) of skull in left lateral view (NHMUK RU B54). Hatching indicates broken bone; dashed lines indicate estimated edges; tone indicates matrix. Scale bars equal 2 cm. Abbreviations: ***a*** angular ***adf*** anterior dentary foramen ***aj*** articular scar for the jugal ***antfo*** antorbital fossa ***apmf*** anterior premaxillary foramen ***be*** buccal emargination ***d*** dentary ***d1***, ***d14*** dentary tooth 1, 14 ***imf*** internal mandibular fenestra ***j*** jugal ***l*** lacrimal or left ***m*** maxilla ***Mc*** Meckel’s canal ***mph*** manual phalanges ***pap*** palpebral ***pd*** predentary ***pm*** premaxilla ***pm1***, ***3*** premaxillary tooth 1, 3 ***po*** postorbital ***pra***  prearticular ***q*** quadrate ***qj*** quadratojugal ***r*** right ***sa*** surangular ***scr*** scleral ring ***sp*** splenial ***un*** ungual.

A small set of autapomorphies diagnose this genus and species, which otherwise closely resembles *Heterodontosaurus tucki*. The lengthy initial diagnosis for *Abrictosaurus consors* (as *Lycorhinus consors*; Thulborn 1974: 153–154) amounted to an abbreviated description. The only uniquely derived feature in the diagnosis (two premaxillary teeth) is erroneous, as the base of a third premaxillary tooth is present (Fig. 31). The revised diagnosis in Hopson (1975: 304) is problematic, as it draws on features from the holotype of *Abrictosaurus consors* as well as from a specimen (NHMUK RU A100) that is referred below to *Lycorhinus angustidens* (Table 2).

The recent revision of the diagnosis by Norman et al. (2011: 236) included five features. Two of these partially overlap those enumerated in the diagnosis above, namely the absence of enlarged caniniform teeth and reduced ornamentation on cheek tooth crowns. One feature listed by Norman and colleagues (12-14 dentary teeth) is primitive with a broader distribution than *Abrictosaurus consors*. Another feature (dorsoventrally expanded anterior end of the dentary) is regarded here as a synapomorphy for derived heterodontosaurids including *Heterodontosaurus tucki*. The absence of a projecting maxillary ridge and buccal emargination, the final feature cited in the revised diagnosis of Norman et al. (2011), is regarded here as an artifact of preservation. The neurovascular openings, everted rim on the maxilla, and form of the opposing depression on the dentary clearly indicate that *Abrictosaurus consors* had a buccal emargination comparable in depth to that in other heterodontosaurids (Figs 34A, 35). Postmortem transverse compression of the skull has brought the mandibles together and flattened prominent facial features.

*Abrictosaurus consors* is most similar in cranial and postcranial morphology to *Heterodontosaurus tucki*. They differ most noticeably in the absence of well-formed caniniform teeth in *Abrictosaurus consors* and in the shape and ornamentation of noncaniniform teeth. The presence and size of caniniform teeth may be the product of sexual dimorphism (Thulborn 1974), although the rarity of reduced caniniform teeth among the many specimens of heterodontosaurids now in collections argues against this interpretation (Norman et al. 2011).

##### Description.

The holotypic skeleton of *Abrictosaurus consors* (NHMUK RU B54), which is preserved for the most part in natural articulation, is the most complete specimen of a heterodontosaurid with the single exception of a nearly complete skeleton of *Heterodontosaurus tucki* (SAM-PK-K1332; Santa Luca et al. 1976; Santa Luca 1980). The following brief description of the holotypic specimen clarifies aspects of the morphology critical to the taxonomy and phylogenetic relationships of this important heterodontosaurid. The skull and skeleton require further preparation before a more detailed description is possible. The skull and femur of the single known specimen of *Abrictosaurus consors* are approximately 71% and 68% of the length of the skull and femur in *Heterodontosaurus tucki* (SAM-PK-K1332; Table 3), respectively. This suggests that *Abrictosaurus consors* probably grew to a comparable adult body size.

Preserved in two pieces, the skull is transversely compressed and better exposed in left lateral view (Fig. 31). Much of the dorsal skull roof, braincase, and posterior end of the lower jaws are broken away. The postcranial skeleton is preserved on separate blocks, one preserving an articulated right forelimb (Fig. 36) and the other the ilia, an ischium, sacrum and left hindlimb (Fig. 37). The phalanges of the right manus cross onto the skull block and are partially exposed near the scleral ring in the left orbit (Fig. 31).

##### Premaxilla.

Both premaxillae are partially preserved with the right shifted slightly ventral to the left. Portions that are broken on both sides include sections of the alveolar border, the internarial processes, and the distal end of both posterolateral processes (Fig. 31). The posterior end of the alveolar margin on the left side was originally shown as complete (Fig. 34A) but now is damaged (Fig. 31). The premaxillary tooth row is set below the maxillary tooth row, although the “overhanging and hood-like” positioning of the premaxilla (Thulborn 1974: 154; Fig. 34A) appears to have been enhanced by postmortem displacement. The form of the premaxilla is very similar to that in *Heterodontosaurus*. Although incomplete, the narial fossa is broad and extends toward the alveolar margin (Figs 31, 35).

**Figure 32. F32:**
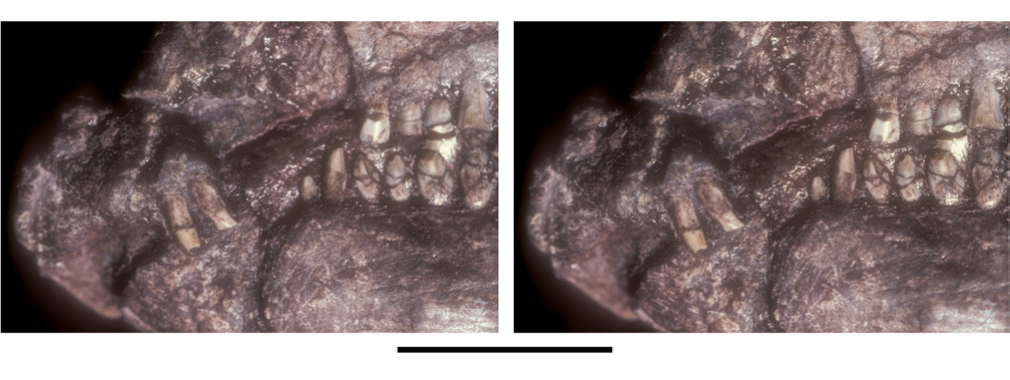
Snout of A*brictosaurus consors* from the Lower Jurassic Upper Elliot Formation of South Africa. Stereopair of anterior end of skull in left lateral view (NHMUK RU B54). Scale bar equals 5 mm.

The posterolateral process of the premaxilla narrows slightly posterior to the external naris and then broadens in transverse width distally, which in *Heterodontosaurus* is directly related to the anterior margin of the arched premaxilla-maxilla diastema (Fig. 59). In *Abrictosaurus* the region of a potential arched diastema, however, is incomplete on both sides (Figs 31, 32). The lower dentition would not have necessitated a diastema, as there is no development of the typical heterodontosaurid dentary caniniform tooth (Fig. 35).

Thulborn suggested, nonetheless, that caniniform crowns in heterodontosaurids might be sexually dimorphic, that NHMUK RU B54 may represent a female, and that a deep arched diastema may have been present in *Abrictosaurus* without an opposing lower caniniform tooth. Norman et al. (2011) presented a similar interpretation (Fig. 34B). Although sexual dimorphism remains a plausible hypothesis, details in the region of the diastema on both sides suggest that only a small diastema could have been present in *Abrictosaurus*. First, the alveolar margin of the right maxilla, which is preserved farther anteriorly than the left, extends anteriorly as a horizontal border as far as the anterior end of the dentary. There is no hint of an arched embayment on the right side. Second, the left dentary tooth 2, which is positioned below the proposed diastema, is truncated by an oblique wear facet (Fig. 32), indicating tooth-to-tooth contact with an opposing anterior maxillary crown (Figs 31, 32). Although no maxillary crowns are preserved in place above this tooth on either side, this planar wear facet suggests that a maxillary crown dorsal to this tooth was originally present on the anterior maxillary alveolar margin and that it has broken away (Fig. 35). It seems doubtful, finally, that the hypertrophied tooth in a caniniform-diastema complex would be reduced, while the recessed fossa that houses such a crown upon jaw closure would be maintained.

##### Maxilla, lacrimal, postorbital, and palpebral.

The maxilla has a dorsoventrally deep buccal emargination, which is bordered dorsally by an arched row of large neurovascular foramina and the everted and gently arched external rim of the antorbital fossa. The narrow width of the anterodorsal ramus brings the premaxilla in close proximity to the antorbital fossa as in *Heterodontosaurus*. At the base of this maxillary ramus, the anterior corner of the external opening of the antorbital fossa has a broader arc than in *Heterodontosaurus*. The external opening of the antorbital fossa is subtriangular (Fig. 35) as in *Heterodontosaurus*. As seen in lateral view (Figs 31, 35), the dorsally arched alveolar margin of the maxillary tooth row also resembles the condition in *Heterodontosaurus*.

The subtriangular lacrimal may be slightly broader dorsally than in *Heterodontosaurus*, its lateral surface lacking the subtriangular external fossa present in the latter genus. A slender, dorsoventrally flattened palpebral is preserved near its natural articulation with the lacrimal. In cross-section and length, the palpebral closely resembles that in *Heterodontosaurus* (Figs 31, 35). This bone was previously identified as the prefrontal (Fig. 34).

##### Quadratojugal and quadrate.

The partially preserved quadratojugal and ventral end of the quadrate form a T-shaped junction in *Abrictosaurus* as in *Heterodontosaurus*. The jaw joint appears to be preserved in natural articulation (Fig. 31). In *Abrictosaurus* the jaw articulation is set below the tooth row to the level of a horizontal line passing through the middle of the dentary ramus (Figs 31, 34).

##### Lower jaw.

The dentary has a deep, arched buccal emargination that dissipates anteriorly near the subconical second tooth (Figs 31, 35). An anterior dentary foramen is located farther ventrally under the same tooth as in *Heterodontosaurus* (Figs 31, 35). The anterior end of the dentary is more strongly expanded dorsoventrally than in *Heterodontosaurus*, although both are similar in form. The articular surface for the predentary, as in *Heterodontosaurus* and *Pegomastax* gen. n. sp. n., is saddle-shaped; in lateral view the articular surface is dorsoventrally convex and transversely concave (Fig. 32). In addition, a rugose swelling here termed a dentary boss is present on the lateral aspect of the anterior end (Figs 32, 59).

The posterior margin of the dentary is not well preserved; the notched posterior margin of the right dentary suggests that an external mandibular fenestra of moderate size may have been present as in *Heterodontosaurus tucki* (*contra* Thulborn 1974: 158). An internal mandibular foramen is also present between the splenial and prearticular in medial view of the right mandibular ramus (*contra* Thulborn 1974: 158; Fig. 31). The splenial tapers anteriorly before reaching the symphysis, where a section of Meckel’s canal is exposed. The canal is represented by a narrow trough running just above the ventral margin of the dentary (Fig. 31) as in *Echinodon*, *Heterodontosaurus* and several other heterodontosaurids.

##### Premaxillary teeth.

There are three premaxillary crowns as in other heterodontosaurids (rather than two, *contra* Thulborn 1974: 15) (Fig. 31, 32). The first and smallest of the premaxillary teeth is preserved on the left side, its crown broken away flush with the alveolar margin (Figs 31, 32). This tooth (pm1) is inset a good distance from the anterior end of the premaxilla. Pm2 and 3 are subconical and slightly recurved, their apical ends broken away. Judging from the preserved portions of the crowns, more of the crown tip of pm3 is broken away than in pm2. Their smooth crowns are bounded mesially and distally by low, but distinct, edges. The crown merges with the root without an intervening neck (Fig. 10A), unlike the premaxillary teeth in *Echinodon* and the first two premaxillary crowns in *Heterodontosaurus*. In *Abrictosaurus* and other heterodontosaurids, pm3 is the largest tooth in the premaxillary series, although in *Abrictosaurus* the size differential is minor. Pm3 is not transversely compressed, and there is no development of serrations on mesial or distal edges of the crown (Fig. 31).

##### Maxillary teeth.

A complete maxillary series probably included 14 teeth, as estimated from the 12 preserved maxillary teeth in the left maxilla and the space between these and the anterior end of the maxilla on the right side (Figs 31, 35). At least two small teeth may have been present at the mesial end of the series, indicating there were more than 12 maxillary teeth (*contra* Thulborn 1974: 158). The teeth are numbered accordingly, accounting for two missing anterior crowns. In *Abrictosaurus*, *Heterodontosaurus* and *Echinodon*, the tooth rows are nearly straight in apical view, whereas in *Lycorhinus* and “*Geranosaurus”* they are bowed medially (Gow 1990; Broom 1911). Opposite to the condition in *Echinodon*, the maxillary teeth are slightly taller than opposing dentary teeth along the tooth row (Figs 31, 35).

*Abrictosaurus* has very distinctive maxillary crowns, best exemplified in the middle portion of the tooth rows. The maxillary crowns are extremely tall, their height more than twice their maximum width in the anterior center of the tooth row (teeth 5-10). Crown proportions (height versus width) decrease distally toward the end of the tooth row. Some of the extra crown height of the central teeth is accommodated by an upward arching of the alveolar margin as in *Heterodontosaurus*, which keeps the axis of occlusion horizontal (Fig. 35). In *Echinodon* and *Lycorhinus*, in contrast, the crowns in the tooth row are more similar in size and the alveolar margins less arched.

The cingulum is reduced in *Abrictosaurus* with the crown in most teeth expanding gently from the root. The best-developed cingulum occurs in smaller teeth at the distal end of maxillary and dentary tooth rows. In these teeth, as in *Heterodontosaurus*, the crown expands more abruptly from the root (Figs 31, 32).

In labial view, mesial and distal crown edges are nearly parallel, with no development of marginal ridges like those in *Echinodon*, *Lycorhinus*, and *Heterodontosaurus*. The denticulate margin near the top of the crown has approximately five to six denticles to each side. These margins slope at a low angle, about 30 degrees from the perpendicular to the crown axis. The denticulate portion of the crown, thus, is confined to the apical 25% of crown height. In cross-section, the crowns are blade-shaped and transversely narrower than in *Lycorhinus* and *Heterodontosaurus*. The enamel may have an asymmetrical distribution, thicker on the labial side of the crown, but this is not well established.

The maxillary crowns are canted at a slight angle to their roots, directing the crown lingually. Low-angle wear facets graze the lingual side of the maxillary crowns. As in *Heterodontosaurus* more than one facet is present on a single crown, as opposing cheek teeth are not aligned one-to-one. Contrary to Thulborn (1974: 154, 159), the wear facets do not lie in a single plane and two wear facets, rather than only one, occur adjacent to one another on several crowns. Maxillary and dentary teeth did not occlude one-to-one as implied by Thulborn, nor do they occlude in strict alternation as suggested by Weishampel (1984: 53).

**Figure 33. F33:**
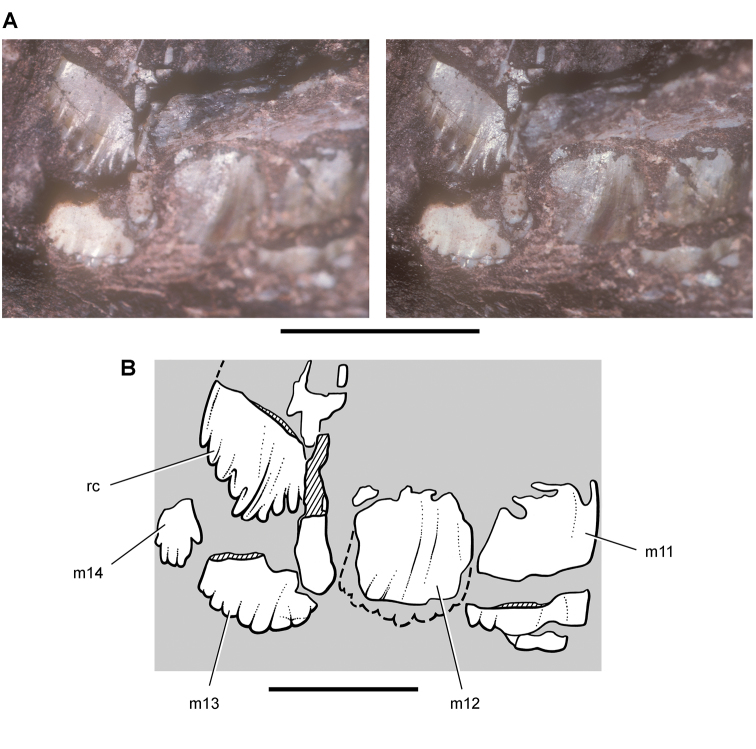
Maxillary teeth of *Abrictosaurus consors* from the Lower Jurassic Upper Elliot Formation of South Africa. Stereopair (**A**) and line drawing (**B**) of replacing crown medial to posterior right maxillary tooth in medial (lingual) view (NHMUK RU B54). Hatching indicates broken bone; dashed lines indicate estimated edges; tone indicates matrix. Scale bar in **A** equals 5 mm; scale bar in **B** equals 3 mm. Abbreviations: ***m11-14*** maxillary tooth 11–14 ***rc*** replacement crown.

There is indisputable evidence for active tooth replacement in *Abrictosaurus*. The base of the penultimate maxillary tooth on the right side has broken away to expose an erupting crown (Fig. 33). Thulborn (1974: 159) stated there is no evidence of active tooth replacement in *Abrictosaurus*, suggesting that the entire maxillary and dentary tooth rows were replaced simultaneously during aestivation (Thulborn 1978). The evidence from the dentition does not support this scenario.

##### Dentary teeth.

The left dentary has 14 teeth, which may comprise a complete tooth row (Figs 31, 35). A small distalmost dentary tooth, however, is preserved on the right side that may constitute dentary tooth 15, which could be obscured by matrix on the left side. The first two dentary teeth have crowns with an atypical shape (Figs 32, 35), and these may correspond to the peglike tooth and caniniform tooth in some heterodontosaurids such as *Lycorhinus*. The small first tooth has a smooth subconical crown that is swollen slightly above the root. The larger second crown, which also shows some swelling above the root, has a convex mesial margin and low unornamented distal carina (Fig. 32). This tooth may represent a reduced caniniform tooth, as it occupies a similar position at the anterior end of the buccal emargination.

The remaining dentary teeth have diamond-shaped crowns that are shorter than opposing maxillary crowns (Fig. 31). The first five dentary tooth crowns do not overlap one another. The remainder of the dentary crowns and all of the maxillary crowns are closely spaced or in contact. As much as 50% of the crown is bordered by mesial and distal denticulate margins, which are more steeply inclined than the denticulate margins of opposing maxillary crowns. *Echinodon* also exhibits a similar differential in the inclination of the denticulate margins between maxillary and dentary crowns, the latter also more steeply inclined.

##### Skull reconstruction.

**Figure 34. F34:**
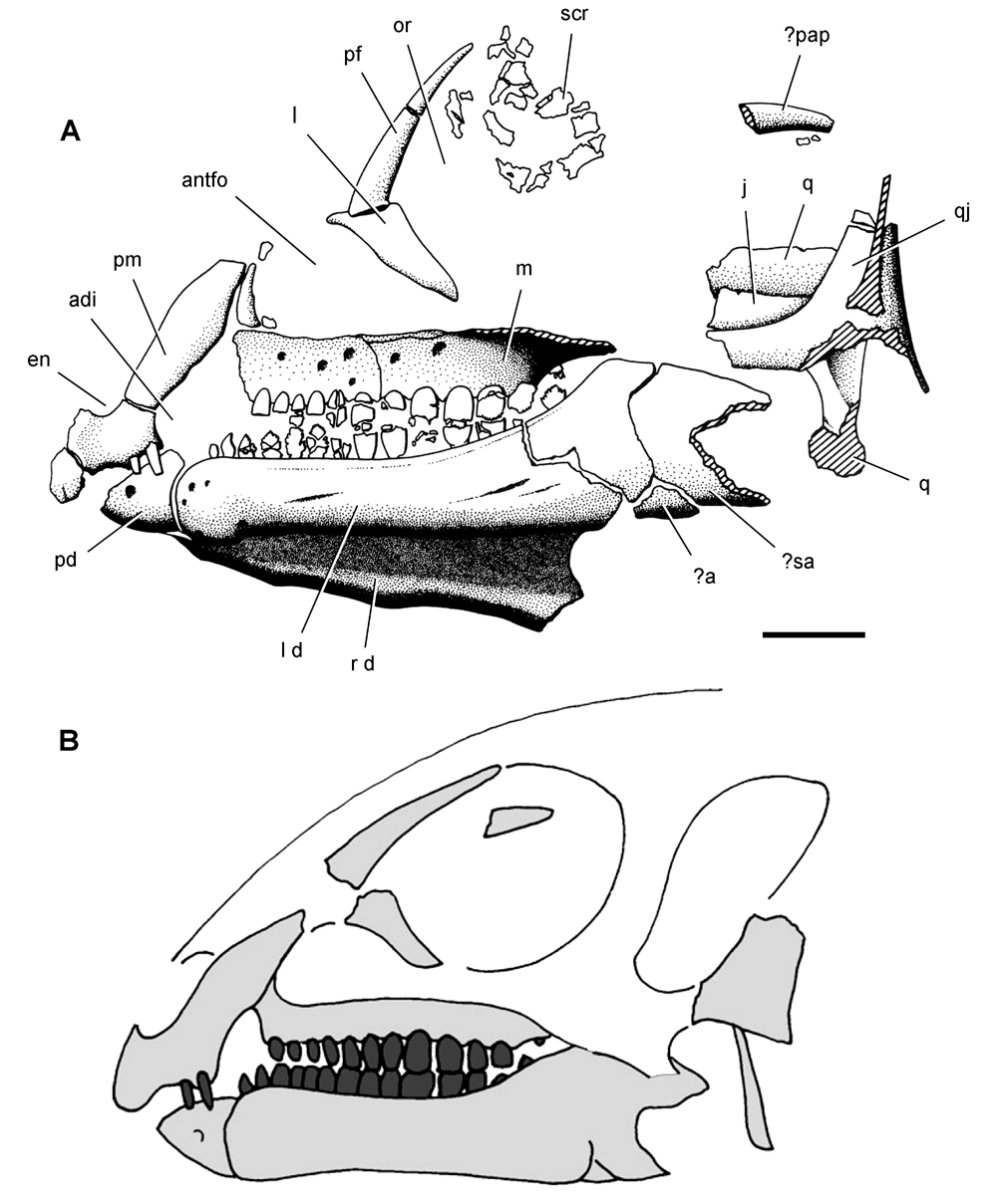
Skull of *Abrictosaurus consors* from the Lower Jurassic Upper Elliot Formation of South Africa. **A** Skull bones of NHMUK RU B54 in left lateral view as initially identified (from Thulborn 1974). **B** Diagrammatic skull reconstruction in left lateral view based on NHMUK RU B54 (from Norman et al. 2011). Scale bar equals 1 cm in **A**. Abbreviations: ***a*** angular ***adi*** arched diastema ***antfo*** antorbital fossa ***d*** dentary ***en*** external nares ***j*** jugal ***l*** lacrimal or left ***m*** maxilla ***or*** orbit ***pap*** palpebral ***pd*** predentary ***pf*** prefrontal ***pm*** premaxilla ***q*** quadrate ***qj*** quadratojugal ***r*** right ***sa*** surangular ***scr*** scleral ring.

**Figure 35. F35:**
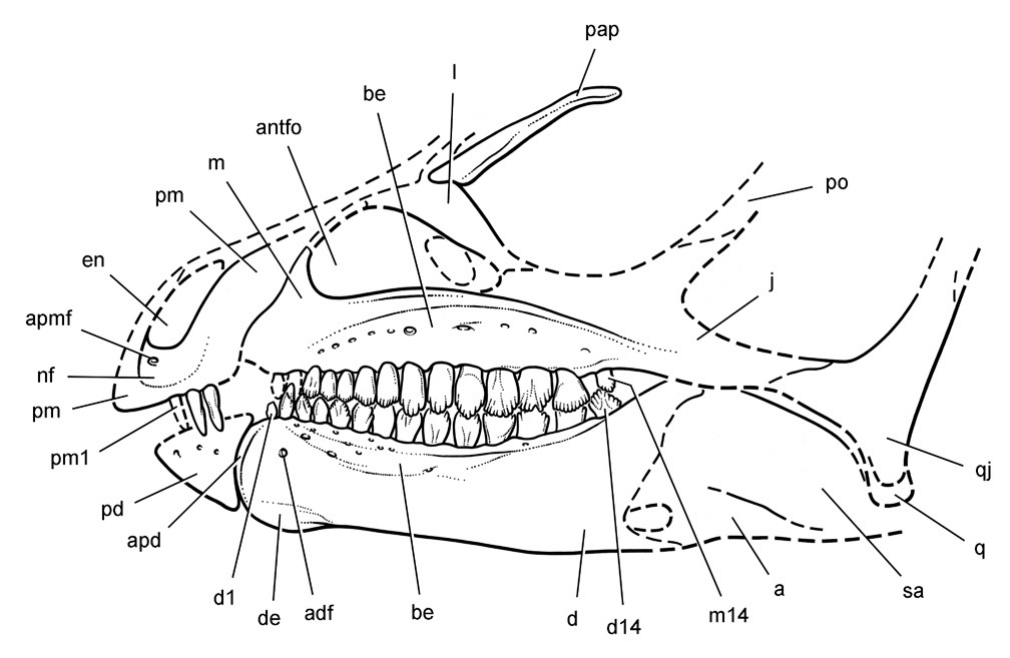
Skull of *Abrictosaurus consors* from the Lower Jurassic Upper Elliot Formation of South Africa. Skull reconstruction in left lateral view based on NHMUK RU B54. Dashed lines indicate estimated edges and sutures. Abbreviations: ***a*** angular ***adf*** anterior dentary foramen ***antfo*** antorbital fossa ***apd*** articular surface for the predentary ***apmf*** anterior premaxillary foramen ***be*** buccal emargination ***d***  dentary ***d1***, ***d14*** dentary tooth 1, 14 ***de*** dentary expansion ***en*** external nares ***j*** jugal ***l*** lacrimal ***m*** maxilla ***m14***  maxillary tooth 14 ***nf*** narial fossa ***pap*** palpebral ***pd*** predentary ***pm*** premaxilla ***pm1*** premaxillary tooth 1 ***po*** postorbital ***q*** quadrate ***qj*** quadratojugal ***sa*** surangular.

The reconstruction of the partial skull and dentition of *Abrictosaurus* (Fig. 35) differs in several regards from previous reconstructions (Fig. 34; Thulborn 1974; Norman et al. 2011). In the present reconstruction, the premaxilla is rotated in a clockwise direction to restore its position relative to the maxilla and bring the premaxillary tooth row, now with three teeth rather than two, closer to a horizontal orientation. Based on evidence from the right side of the skull, the anterior end of the maxilla is restored, the diastema reduced, and two small teeth added to the anterior end of the tooth row (Fig. 35). The alveolar margins of the dentary and maxillary tooth rows are arched ventrally and dorsally, respectively, as preserved on the skull and as in *Heterodontosaurus*. Using the better-preserved skulls of *Heterodontosaurus* as a guide, the palpebral is rotated clockwise to align it with the dorsal margin of the snout. The external opening of the antorbital fenestra is enlarged, following preserved margins on the maxilla and lacrimal. Finally, the ventral margins of the predentary and anterior end of the dentary are reduced and expanded, respectively, as preserved (Fig. 34).

##### Axial skeleton.

The articulated cervical series is only partially exposed. The atlas and axis are incomplete, and C3-9 are exposed in dorsal view. Judging from their neural arches, length decreases markedly from C6-9 as in *Heterodontosaurus*. The neural arches of C8 and C9 are only approximately one-half that of C3.

In C3 the neural spine is low and ridgelike and the postzygapophyses have smooth dorsal surfaces lacking any development of epipophyseal processes. In *Heterodontosaurus*, in contrast, a well developed epipophysis projects as a subconical process from the postzygapophysis of C3. The longer, arched postzygapophyses of C5 and shorter postzygapophyses of C6 also lack discrete epipophyseal processes. A low crest at the base of each of these postzygapophyses joins the low neural spine. In C6 the tab-shaped neural spine is located at the anterior end of the neural arch. Although the form of these vertebrae is similar to that in *Heterodontosaurus*, important differences include the absence of the unusual anterodorsally inclined neural spines in C5 and C6.

Ossified tendons are associated with the neural arches of the dorsal and sacral vertebrae, but as in *Heterodontosaurus* few of these are in natural position. Ossified tendons appear to be limited to dorsal and sacral regions of the vertebral column, and none is present in the proximal portion of the caudal series (Fig. 37).

##### Pectoral girdle.

The scapula, the only bone of the pectoral girdle that is exposed, shows a prominent acromial expansion and strap-shaped proximal scapular blade. Little else can be said without further preparation.

##### Forelimb.

**Figure 36. F36:**
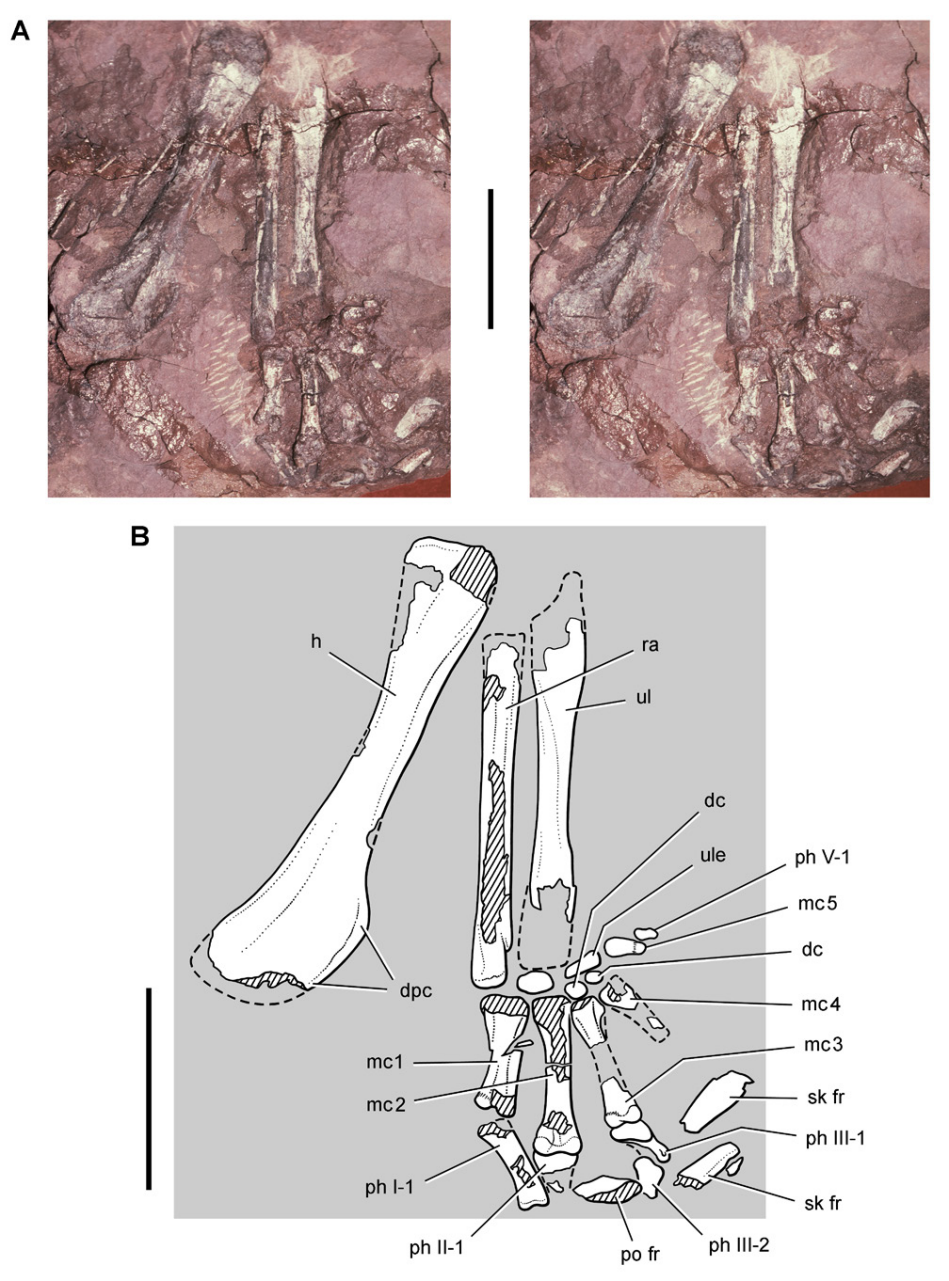
Forelimb of *Abrictosaurus consors* from the Lower Jurassic Upper Elliot Formation of South Africa. Stereopair (**A**) and line drawing (**B**) of the right forelimb in anterior and ventral views (NHMUK RU B54). Hatching indicates broken bone; dashed lines indicate estimated edges; tone indicates matrix. Scale bars equal 2 cm in **A** and **B**. Abbreviations: ***dc*** distal carpal ***dpc*** deltopectoral crest ***fr*** fragment ***h*** humerus ***mc1-5*** metacarpals 1-5 ***ph*** ***I-1*** phalanx 1 of manual digit I ***ph*** ***II-1*** phalanx 1 of manual digit II ***ph***  ***III-1*** phalanx 1 of manual digit III ***ph*** ***III-2*** phalanx 2 of manual digit III ***ph*** ***V-1*** phalanx 1 of manual digit V ***po*** postorbital ***ra*** radius ***sk*** skull ***ul*** ulna ***ule*** ulnare.

The right forelimb is nearly complete with most of its bones in articulation or near their natural location and with the manus in ventral view overlapping the left orbit of the skull (Fig. 36). The humerus, exposed in anterior view, is poorly preserved proximally and distally. The deltopectoral crest projects strongly from the proximal shaft. The length of the crest is about 34% of humeral length (Table 6). In *Heterodontosaurus*, the proximal end of the crest arches more abruptly from the shaft than in *Abrictosaurus*, and the relative length of the crest is greater (approximately 42% of the humerus) (Table 8).

The radius and ulna are preserved on both sides but are incomplete distally and only partially exposed (Fig. 36). In *Heterodontosaurus* the ulnar shaft thickens on its ventral aspect prior to the strong proximally projecting olecranon process. Although that process is not preserved or exposed in *Abrictosaurus*, the right ulna shows the ventral thickening of the proximal shaft.

The carpus is composed of many elements, which may have been assembled in articulation with a compact arrangement as in *Heterodontosaurus*. In the right carpus, three carpals are preserved proximal to metacarpals 3 through 5 (Fig. 36). The large ovoid bone distal to the ulna and closest to the base of metacarpal 5 may represent the ulnare, which in *Heterodontosaurus* is even larger and more subrectangular in shape. The pair of smaller subspherical elements associated with the bases of metacarpals 3 and 4 may represent distal carpals. In *Heterodontosaurus* the distal carpals in the lateral side of the carpus appear more lenticular in shape with an overlapping arrangement (Fig. 67C).

The metacarpals are proportionately elongate relative to either the bones of the forearm or humerus as in *Heterodontosaurus* (Fig. 36). Metacarpal 2, the most complete of the series, is approximately 32% of humeral length (Table 6). The same proportion in *Heterodontosaurus* is only 27% (Table 8), and thus the hand in *Abrictosaurus* appears to be proportionately longer. An unusual feature of the metacarpus in *Heterodontosaurus* is that metacarpal 2 is slightly longer and more robust than metacarpal 3 (Table 8). Their distal condyles, nevertheless, are nearly aligned, as the base of metacarpal 2 is inset slightly into the carpus relative to metacarpal 3 (Fig. 36). The slightly inset position of the base of metacarpal 2 relative to metacarpal 3 also is preserved on both sides in the articulated skeleton of *Heterodontosaurus* (Fig. 65). Metacarpals 1-3 are more robust and longer than metacarpals 4 and 5, the former measured from the left manus. Metacarpal 5 is the shortest metacarpal with a shorter length than in *Heterodontosaurus* (Tables 6, 8).

The proximal ends of the metacarpals show edges that appear to square the base of the bone as in *Heterodontosaurus* and the Kayenta heterodontosaurid. The squared bases articulate against each other in *Heterodontosaurus*, and a similar condition may hold for at least the medial metacarpals in *Abrictosaurus* (Fig. 36). In *Heterodontosaurus*, however, the metacarpus is exposed only in dorsal view (Figs 65, 67). In *Abrictosaurus*, in contrast, the metacarpus is currently exposed only in ventral view. The distal ends of metacarpals 1-3 are expanded, and thus it seems likely that distal extensor pits would be present dorsally for hyperextension of the phalanges as in *Heterodontosaurus*.

The right metacarpus, exposed in ventral view, provides the best information regarding the disposition of the digits (Fig. 36). Metacarpal 1 diverges medially only slightly from an axis established on the bones of the forearm. As in *Heterodontosaurus* (Fig. 67A), the distal condyles are strongly asymmetrical, which cants the phalanges of digit I medially. Digit V, preserved only in the right manus, diverges at nearly a right angle laterally as in *Heterodontosaurus* and other bipedal ornithischians such as *Hypsilophodon* (Galton 1974a). The articulation of the base of metacarpal 5 with the lateral aspect of the ulnare, which is also present in *Heterodontosaurus*, seems to be the natural disposition of this short digit (Figs 36, 67).

The phalangeal formula is poorly known in *Abrictosaurus*. In the left manus, digit IV has two phalanges, and in the right manus digit V has one (Fig. 36). The distal ends of phalanges IV-2 and V-1 are rounded, and so the presence of a diminutive nonungual terminal phalanx in each digit, as occurs in *Heterodontosaurus* (Fig. 67),cannot be ruled out. The phalanges of digits I-III are not well exposed. The proximal phalanges of digits I-III show a similar decrease in length to the lateral side as in *Heterodontosaurus*. The distal ends of phalanges I-1 and II-1 are preserved on the skull block over the left orbit (Fig. 31). It is possible that the remaining phalanges of digits I-III are embedded in matrix within the orbit. The edge of an extremely long recurved element may represent the edge of an ungual. Detailed preparation of the skull block is warranted.

##### Pelvic girdle.

**Figure 37.**
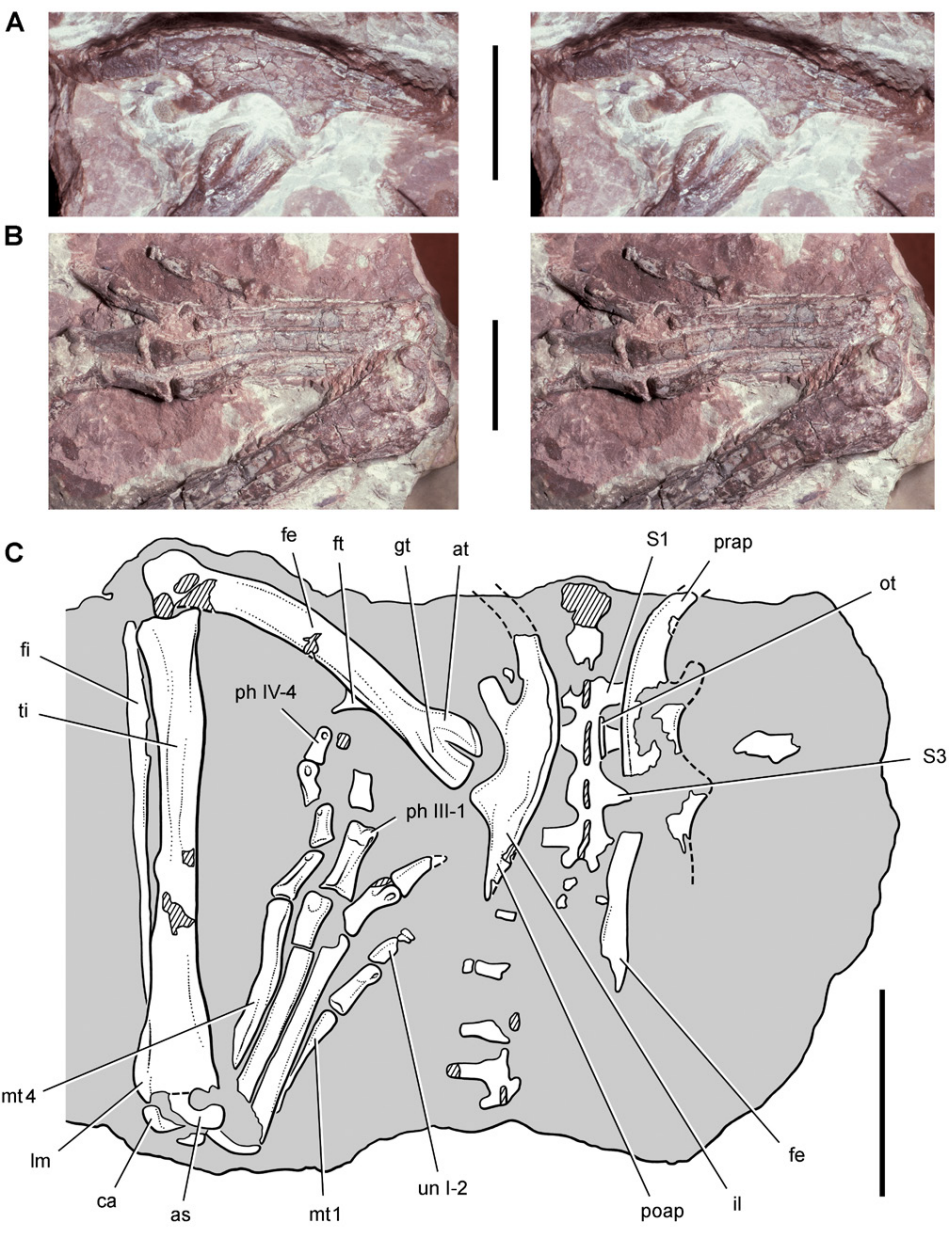
Ilium and hindlimb of *Abrictosaurus consors* from the Lower Jurassic Upper Elliot Formation of South Africa. Stereopairs of the left ilium in lateral view (**A**) and left pes in dorsal view (**B**) and line drawing of the sacrum, ilia and left hindlimb mostly in dorsal view (**C**) (NHMUK RU B54). Hatching indicates broken bone; dashed lines indicate estimated edges; tone indicates matrix. Scale bars equal 2 cm in **A** and **B**, 4 cm in **C**. Abbreviations: ***I***, ***III***, ***IV*** digits I, III, IV ***as*** astragalus ***at*** anterior trochanter ***ca*** calcaneum ***fe*** femur ***fi*** fibula ***ft*** fourth trochanter ***gt*** greater trochanter ***il*** ilium ***lm*** lateral malleolus ***mt1***, ***4*** metatarsals 1, 4 ***ot*** ossified tendon ***ph*** phalanx ***poap*** postacetabular process ***prap*** preacetabular process ***S1***, ***3*** sacral vertebra 1, 3 ***ti*** tibia ***un*** ungual.

The ilium, preserved in part on both sides, is very similar to that in *Heterodontosaurus* (Fig. 37, Table 6). The gently everted dorsal margin arches between the slender preacetabular process and the proportionately long postacetabular process. Although incomplete, the anterior end of the preacetabular process appears to taper gradually rather than terminating with a gentle lobe-shaped expansion as in *Heterodontosaurus* (Santa Luca 1980: fig. 18A). The acetabulum, also similar to that in *Heterodontosaurus*, is completely open with no development of a supraacetabular rim. The pubic peduncle is longer and narrower than the more robust ischial peduncle, which does not project laterally as prominently as in *Heterodontosaurus* (Fig. 68). Only the proximal shaft of the right ischium is preserved. No obturator process is visible, but the shaft is not completely exposed (Fig. 37).

**Table 6.** Measurements (mm) of the holotypic skeleton of the South African heterodontosaurid *Abrictosaurus consors* (NHMUK RU B54). Measurements are rounded to the nearest millimeter and average right and left sides except where indicated. Parentheses indicate estimated measurement.

**Structure**	**Measurement**	
Skull	Length	(100)
Scapula	Blade, minimum width	5
Humerus	Length	50
	Deltopectoral crest length	17
Ulna	Length	(39)
Radius	Length	(36)
Manus	Metacarpal 1 length	13
	Metacarpal 2 length	16
	Metacarpal 3 length	15^1^
	Metacarpal 4 length	(9)^1^
	Metacarpal 5 length	4
	Phalanx I-1 length	10
	Phalanx II-1 length	8
	Phalanx III-1 length	5
	Phalanx V-1 length	2
Ilium	Blade length	62^3^
	Blade, height dorsal to acetabular rim	11
	Preacetabular process length	21^2^
	Postacetabular process length	19
	Pubic peduncle length	11
	Ischial peduncle length	7
Femur	Length	78
	Minimum shaft diameter	8
	Head to distal end of fourth trochanter	30
Tibia	Length	100
	Minimum shaft diameter	7
Pes	Digit III length	(108)
	Metatarsal 1 length	31
	Metatarsal 2 length	47
	Metatarsal 3 length	53
	Metatarsal 4 length	(48)

^1^Left.; ^2^Right.; ^3^Composite measurement based on overlap of right and left sides.

##### Hindlimb.

The general form of the femur does not depart significantly from that in *Heterodontosaurus* (Santa Luca 1980, fig. 18B). The shaft is bowed with a pendant fourth trochanter located proximal to mid-shaft (Fig. 37) as in *Heterodontosaurus*. There is no development of an anterior intercondylar groove. The anterior trochanter, which extends to the level of the greater trochanter, is separated from the shaft of the femur by a deep cleft, as seen in lateral view (Fig. 37). In *Heterodontosaurus*, in contrast, the lesser trochanter projects dorsally alongside the shaft, to which it is fully coossified (Fig. 68). The greater trochanter in *Abrictosaurus* is proportionately narrow, subequal in anteroposterior width to the anterior trochanter (Fig. 37). In *Heterodontosaurus* and neornithischians in general, the greater trochanter is always broader than the lesser trochanter in lateral view. The pendant fourth trochanter is located on the proximal half of the femoral shaft as in *Heterodontosaurus*.

In *Heterodontosaurus* several features in the crus arose in parallel among coelurosaurian theropods, including an articular crest protruding from the shaft of the proximal end of the tibia to support the reduced shaft of the fibula, reduction of the fibular shaft to a narrow rod, and coossification of the distal ends of the tibia and fibula with the proximal tarsals. Distal coossification of the tibia, fibula, and proximal tarsals (as a “tibiofibulotarsus”), however, may be variable in *Heterodontosaurus*. A referred right tibia, fibula, and astragalocalcaneum do not exhibit obliterating coossification between the crus and tarsus (Fig. 70). In *Abrictosaurus* most of the fibular shaft is similarly reduced to a rod, but there does not appear to be any development of a lateral supporting flange on the tibial shaft or distal fusion between the crus and tarsus (Fig. 37).

The tarsus includes the astragalus, calcaneum, and lateral and medial distal tarsals, which do not appear to be coossified (Fig. 37). The flat, platelike medial distal tarsal is preserved in articulation over metatarsals 2 and 3, whereas the smaller cuboid lateral distal tarsal is displaced a short distance from its articulation over metatarsal 4.

The metatarsal shafts are not coossified, and the pes as preserved is less compact than in *Heterodontosaurus* (Fig. 37). The distal end of metatarsal 3 diverges laterally, and the shaft of metatarsal 4 follows a S-shaped curve. These plesiomorphic features are only weakly expressed in *Heterodontosaurus*. In *Abrictosaurus* metatarsal 1 tapers toward its proximal end as an extremely thin splint, which is very narrow both transversely and dorsoventrally. Metatarsal 3 is the longest, with metatarsals 2 and 4 subequal in length. An extensor pit above the distal condyles occurs only on metatarsal 3 rather than on both metatarsals 3 and 4 as in *Heterodontosaurus*. Extensor pits are also developed above the distal condyles of the proximal phalanges of digits I-III. Only pedal digits I and IV preserve complete phalangeal series, which are consistent with the primitive ornithischian phalangeal formula (2-3-4-5-0).

#### Heterodontosaurus tucki

Crompton and Charig, 1962

http://species-id.net/wiki/Heterodontosaurus_tucki

Figs 2C 38 [Fig F39] [Fig F40] [Fig F41] [Fig F42] [Fig F43] [Fig F44] [Fig F45] [Fig F46] [Fig F47] [Fig F48] [Fig F49] [Fig F50] [Fig F51] [Fig F52] [Fig F53] [Fig F54] [Fig F55] [Fig F56] [Fig F57] [Fig F58] [Fig F59] [Fig F60] [Fig F61] [Fig F62] [Fig F63] [Fig F64] [Fig F65] [Fig F66] [Fig F67] [Fig F68] [Fig F69] [Fig F70] [Fig F71] 72 88 [Fig F89] [Fig F90] [Fig F91] [Fig F92] [Fig F93] [Fig F94] [Fig F95] 96 101 Tables 1 [Table T2] 3 7 [Table T8] 9

Heterodontosaurus tucki Crompton and Charig, 1962 – Crompton and Charig (1962, fig. 1); Halstead Tarlo et al. (1963, fig. 1); Galton (1970, fig. 4C); Charig and Crompton (1974, Figs 10, 11); Santa Luca et al. (1976, Figs 1, 2); Thulborn (1978, Figs 1, 2A); Hopson (1980, Figs 4, 6); Santa Luca (1980, Figs 1-23, append. 1); Weishampel (1984, Figs 3, 12C); Crompton and Attridge (1986, Figs 17.9–17.11); Bakker and Galton (1974, fig. 1H); Bakker (1986, fig. on p. 453); Galton (1986, Figs 6R–T, 15.5B); Weishampel and Witmer (1990, Figs 23.3, 23.4); Norman and Weishampel (1991, fig. 7); Brett-Surman (1997, fig. 24.1B); Norman et al. (2004, Figs 18.1, 18.2D, 18.7); Langer and Benton (2006, fig. 8A); Butler et al. (2008, Figs 1-5); Smith (1997, fig. 1); Brusatte et al. (2010, fig. 3D); Porro et al. (2011, Figs 2, 3, 8a-c); Norman et al. (2011, Figs 1–36, append. 3–6)

##### Holotype.

SAM-PK-K337, nearly complete, articulated skull.

##### Referred material.

SAM-PK-K1332, articulated skull and skeleton lacking only a few mid and distal caudal vertebrae; SAM-PK-K10487, anterior portion of a juvenile skull including the left orbit with palpebral and anterior portion of the lower jaws with the predentary; SAM-PK-K1328, partial postcranial skeleton including dorsal and caudal vertebrae, forelimbs and hindlimbs; SAM-PK-K1334, left maxilla with six teeth and portions of adjacent bones preserving the antorbital fenestra, the ventral end of the lacrimal, and the anterior end of the jugal; AMNH 24000, posteroventral portion of skull with articulated lower jaws preserving the posterior half of maxillary and dentary tooth rows, parts of the right jugal, quadratojugal and quadrate, posterior half of the right lower jaw, and traces of the anterior cervical vertebrae.

##### Type locality.

On the mountainside behind the trading store in Tyinindini at an altitude of 1890 m, Transkei (Herschel) District, Cape Province, South Africa; S30°32', E27°32' (Crompton and Charig 1962; Kitching and Raath 1984) (Fig. 1B).

##### Horizon.

Upper Elliot Formation and Clarens Formation (Smith 1990; Knoll 2005); Lower Jurassic, Hettangian to Sinemurian, ca. 200-190 Ma (Crompton and Charig 1962; Santa Luca et al. 1976; Gradstein and Ogg 2009).

##### Revised diagnosis.

Heterodontosaurid ornithischian characterized by the following autapomorphies: (1) cheek tooth crowns subrectangular in cross-section; (2) prominent crown margins and primary ridge resulting in mesial and distal paracingular fossae on labial and lingual faces of maxillary and dentary crowns, respectively; (3) asymmetrical enamel on maxillary and dentary crowns (reduced in thickness on lingual and labial sides of maxillary and dentary crowns, respectively); (4) lacrimal with shallow lateral fossa; (5) jugal with extension of antorbital fossa onto the orbital ramus; (6) jugal flange posteroventrally inclined; (7) trapezoidal anterior surangular foramen; (8) axis and C3 neural spine with lateral flange; (9) C5 and C6 neural spines project anterodorsally; (10) C3-C7 with subcylindrical parapophyses; (11) mid dorsal vertebrae (D6-D10) with Y-shaped transverse processes (for di- and parapophyses); (12) scapulocoracoid foramen absent; (13) humeral epicondyles present; (14) lobe-shaped distal expansion of the iliac preacetabular process; (15) ischial peduncle narrow, columnar; (16) ischial shaft with laterally-directed crescentic flange at mid-length; (17) femoral anterior and greater trochanters coossified; (18) tibiofibulotarsus coossification (possibly variable); (19) tarsometatarsus coossification.

##### Comments.

The initial diagnosis of *Heterodontosaurus tucki* by Crompton and Charig (1962: 1075) listed 10 features, none of which now stand as autapomorphies particular to the species. Some of the listed features, such as an edentulous predentary, are plesiomorphies of broad distribution, whereas others (presence of three premaxillary teeth, arched diastema) now characterize other heterodontosaurids. In a review of ornithischian taxa, Steel (1969: 7-8) listed more than a dozen cranial features in a revised diagnosis, but again none are regarded here as autapomorphies given the diversity of heterodontosaurids now known. The reduction of the cingulum and the presence of a jugal flange, for example, are now known in the recently described South American heterodontosaurid *Manidens* (Pol et al. 2011). In his description of the postcranial skeleton, Santa Luca (1980: 197) presented a revised diagnosis based solely on postcranial features. Of the 20 features listed, 4 are regarded here as autapomorphies that are currently known in other heterodontosaurids (autapomorphies 13, 17-19; humeral epicondyles; femoral anterior and greater trochanters coossified, tibiotarsus and tarsometatarsus coossification).

The recent revision of the diagnosis in Norman et al. (2011: 187) added 19 cranial and dental autapomorphies to a revised list of postcranial features from Santa Luca for a total of 44 characters. The authors marked 30 of these as potential autapomorphies. Many of these features, however, are problematic as autapomorphies. Of the 12 cranial features cited by Norman et al. (2011), 4 are retained in the revised diagnosis above (autapomorphies 2, 5–7). Some of the features cited by these authors but excluded here are known in other heterodontosaurids, such as the everted rim on the maxilla, presence of a jugal horn and flange, T-shaped quadratojugal, the reduction of the cingulum, and the presence of an external mandibular fossa (Thulborn 1974; Pol et al. 2011). The form of the basal tubera and basipterygoid processes also appear to be similar to that in *Manidens* (Pol et al. 2011). One of the cited features, “two fingerlike rami” of the surangular, is based on a damaged surangular as discussed below. One cranial feature new to the diagnosis above is the shallow subtriangular fossa on the lateral aspect of the lacrimal (autapomorphy 4) (Fig. 59).

Two of the seven dental features cited by Norman et al. (2011) are comparable to autapomorphy 2 in the diagnosis above. Other dental features cited by these authors are difficult to differentiate from the condition in several other heterodontosaurids, such as the distribution of serrations on caniniform teeth and reduction of the cingulum. The presence of extensive tooth wear, another cited feature, is not regarded here as diagnostic, given the presence of broad wear facets in *Lycorhinus*, *Abrictosaurus* and *Pegomastax* gen. n. sp. n.Dental features new to the revised diagnosis (autapomorphies 1, 3) include the subrectangular cross-section of the cheek teeth (Fig. 49C) and asymmetrical enamel on maxillary and dentary crowns. The distribution and thickness of enamel on the crowns of *Heterodontosaurus tucki* and other heterodontosaurines is poorly established, and so this may eventually characterize other species. Basal heterodontosaurids such as *Echinodon* and *Fruitadens* do not appear to have asymmetrical enamel on the crowns of the cheek teeth.

Six of the 10 postcranial features cited by Norman et al. (2011) as autapomorphic are reformulated above (as autapomorphies 13, 16–19). Other postcranial features cited by these authors are difficult to defend as autapomorphies, such as the number of precaudal or sacral vertebrae (see discussion below), the presence of epipophyses in anterior cervical vertebrae (present in *Lesothosaurus*), and a narrow scapular blade (present in other heterodontosaurids). Postcranial features new to the revised diagnosis (autapomorphies 8-12, 14, 15) include unusual features of the axial skeleton, the absence of the scapulocoracoid foramen, and the shape of processes in the pelvic girdle.

##### Description.

**Figure 38.**
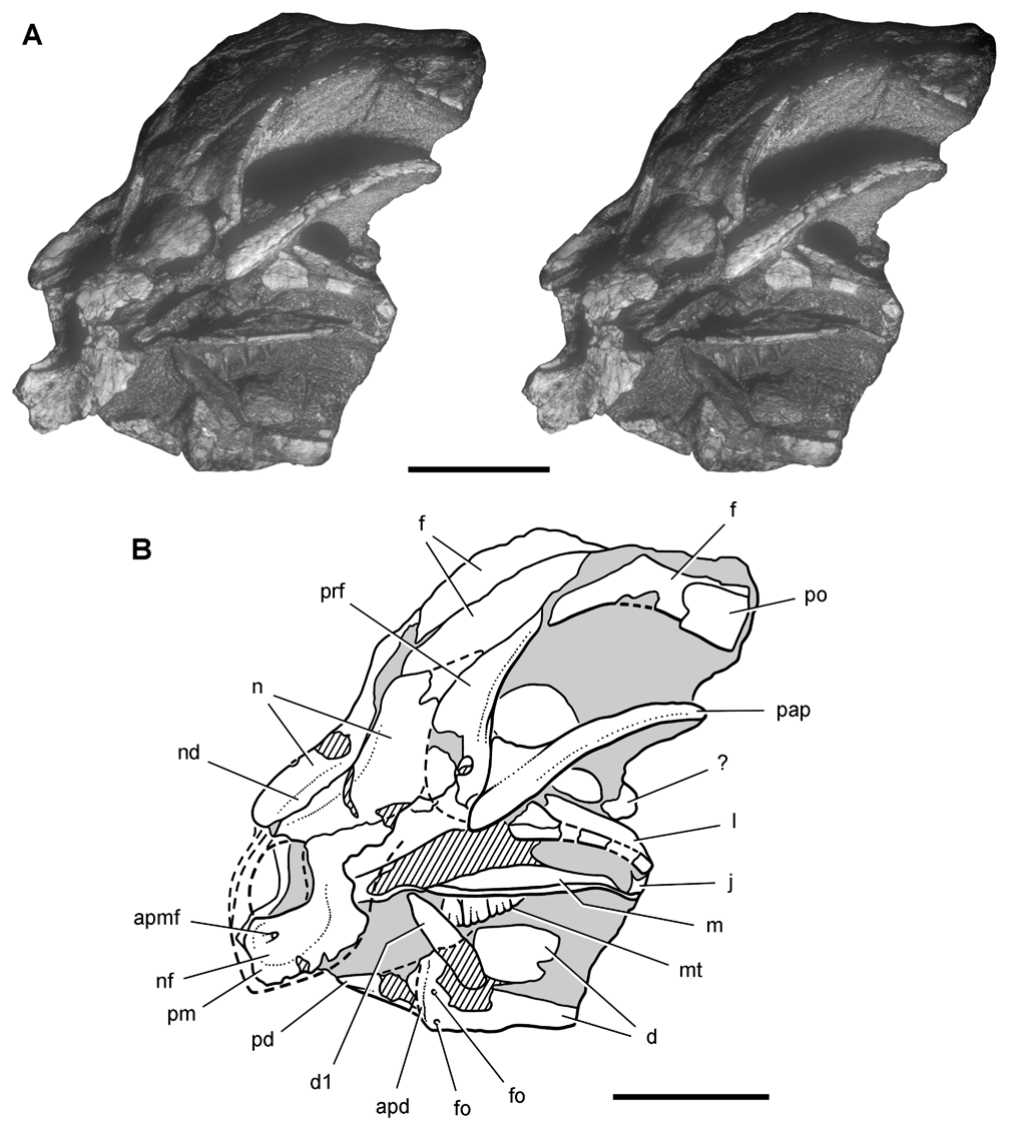
Snout of the heterodontosaurid *Heterodontosaurus tucki* from from the Lower Jurassic Upper Elliot and Clarens formations of South Africa. Anterior one-half of a juvenile skull (SAM-PK-K10487). Stereopair (**A**) and line drawing (**B**) in anterolateral view. Hatching indicates broken bone; dashed lines indicate estimated edges; tone indicates matrix. Scale bars equal 1 cm in **A** and **B**. Abbreviations: ***apd*** articular surface for predentary ***apmf*** anterior premaxillary foramen ***d*** dentary ***d1*** dentary tooth 1 ***f*** frontal ***fo*** foramen ***j*** jugal ***l*** lacrimal ***m*** maxilla ***mt*** maxillary teeth ***n*** nasal ***nd*** nasal depression ***nf*** narial fossa ***pap***  palpebral ***pd*** predentary ***pm*** premaxilla ***po*** postorbital ***prf*** prefrontal.

**Figure 39. F39:**
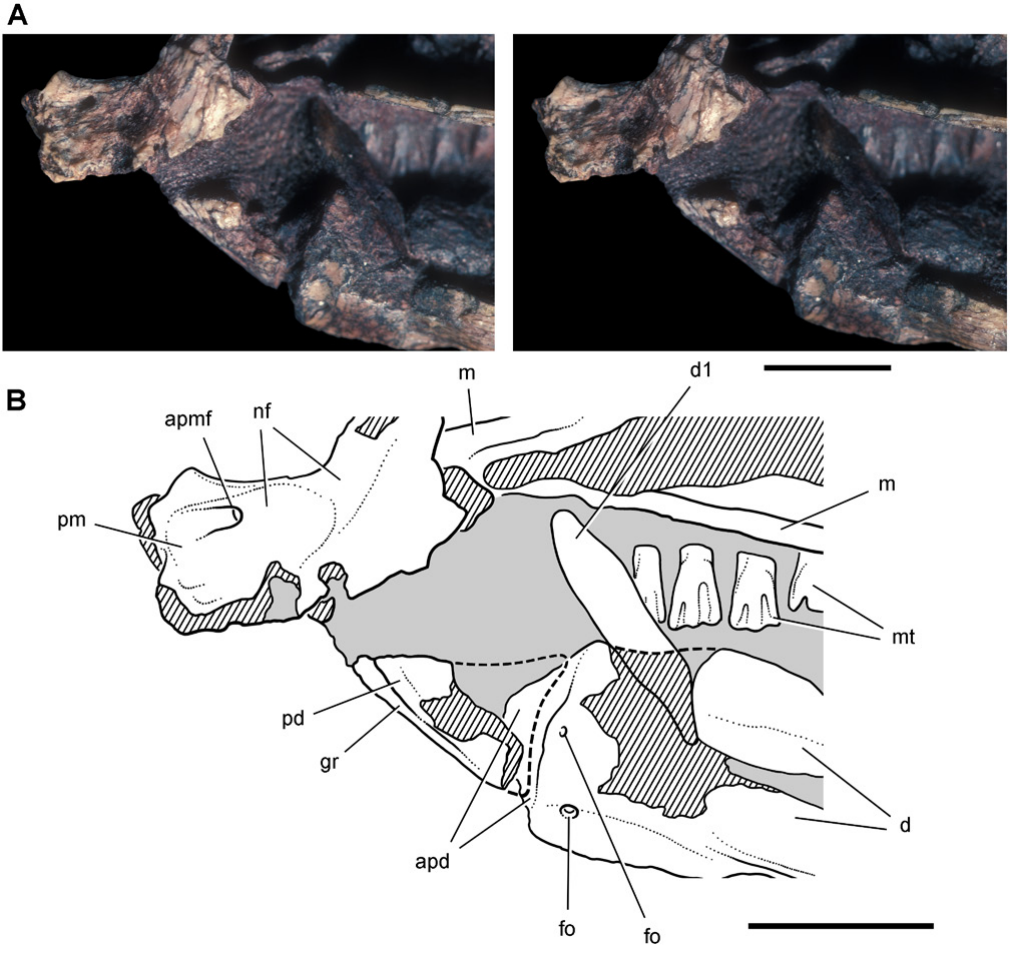
Snout end of *Heterodontosaurus tucki* from from the Lower Jurassic Upper Elliot and Clarens formations of South Africa. Close-up view of the anterior end of a juvenile skull (SAM-PK-K10487). Stereopair (**A**) and line drawing (**B**) in left lateral view. Hatching indicates broken bone; dashed lines indicate estimated edges; tone indicates matrix. Scale bars equal 5 mm in **A** and **B**. Abbreviations: ***apd*** articular surface for the predentary ***apmf*** anterior premaxillary foramen ***d*** dentary ***d1*** dentary tooth 1 ***fo***, foramen ***gr*** groove ***m*** maxilla ***mt*** maxillary teeth ***nf*** narial fossa ***pd*** predentary ***pm*** premaxilla.

**Figure 40. F40:**
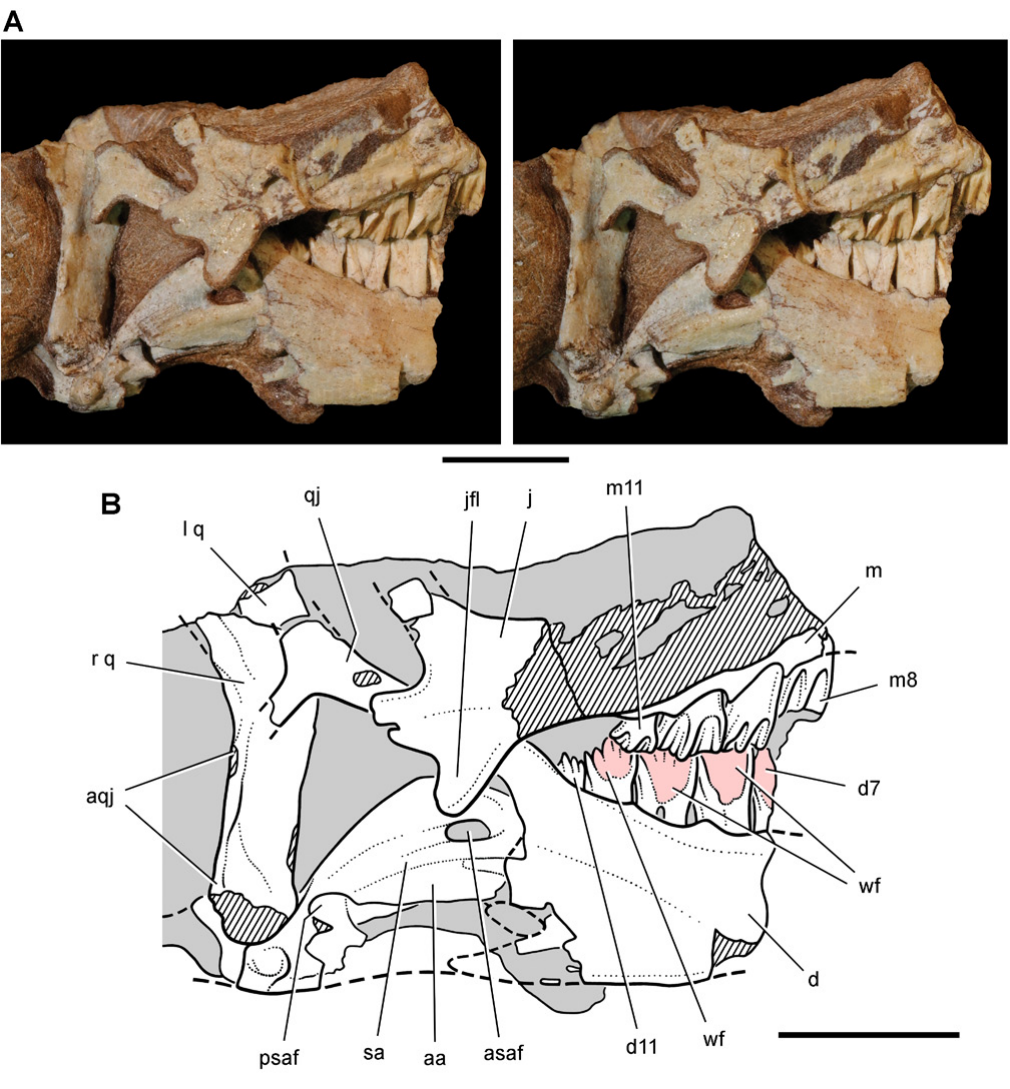
Posterior end of the skull of *Heterodontosaurus tucki* from from the Lower Jurassic Upper Elliot and Clarens formations of South Africa. Posterior portion of a juvenile skull (AMNH 24000). Stereopair (**A**) and line drawing (**B**) in right lateral view. Hatching indicates broken bone; dashed lines indicate estimated edges; grey tone indicates matrix; pink tone indicates wear facets. Scale bars equal 1 cm in **A** and **B**. Abbreviations: ***aa*** articular surface for angular ***aqj*** articular surface for quadratojugal ***asaf***  anterior surangular foramen ***d*** dentary ***d7***, ***11*** dentary tooth 7, 11 ***j*** jugal ***jfl*** jugal flange ***l*** left ***m*** maxilla ***m8***, ***11***  maxillary tooth 8, 11 ***psaf*** posterior surangular foramen ***q*** quadrate ***qj*** quadratojugal ***r*** right ***sa*** surangular ***wf*** wear facet.

**Figure 41. F41:**
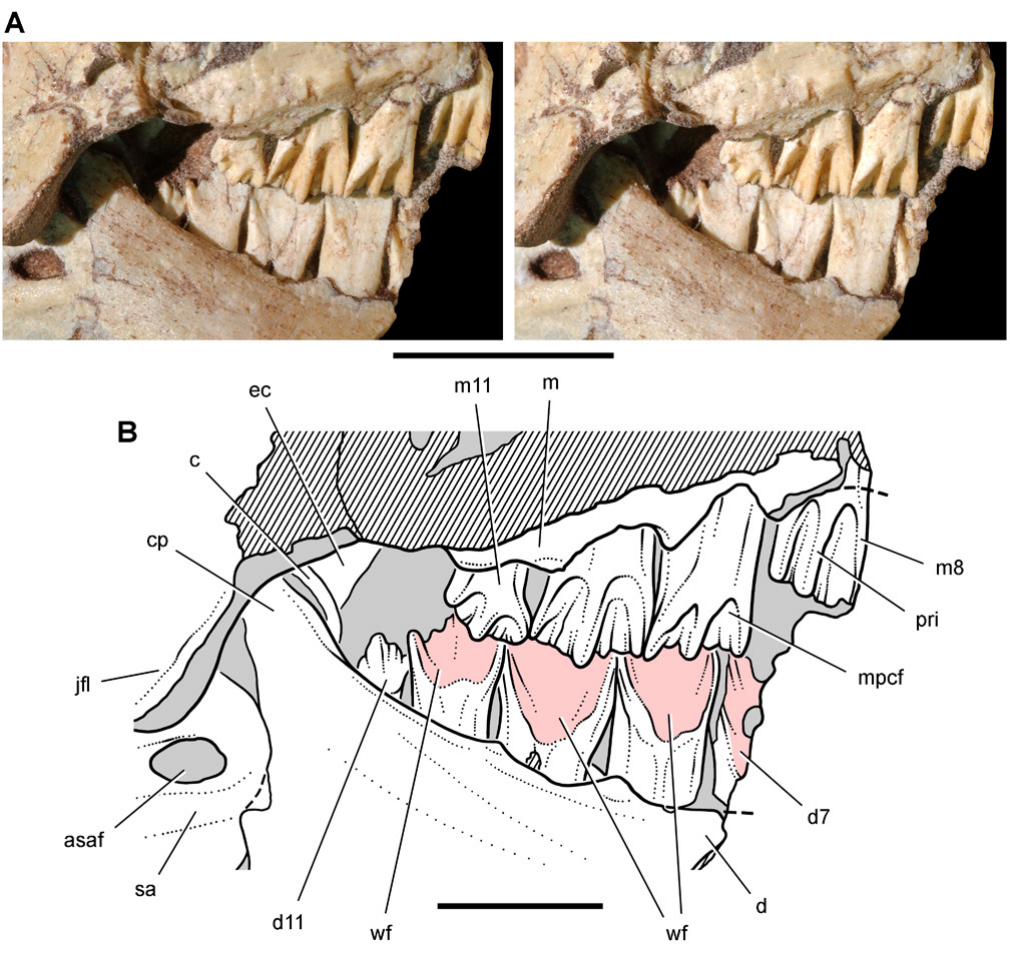
Posterior dentition of *Heterodontosaurus tucki* from from the Lower Jurassic Upper Elliot and Clarens formations of South Africa. Tooth wear and replacement in posterior maxillary and dentary teeth of a juvenile skull (AMNH 24000). Stereopair (**A**) and line drawing (**B**) in right lateral view. Hatching indicates broken bone; dashed lines indicate estimated edges; grey tone indicates matrix; pink tone indicates wear facets. Scale bars equal 2 cm in **A** and 1 cm in **B**. Abbreviations: ***asaf*** anterior surangular foramen ***c*** coronoid ***cp*** coronoid process ***d*** dentary ***d7***, ***11*** dentary tooth 7, 11 ***ec*** ectopterygoid ***jfl*** jugal flange ***m*** maxilla ***m8***, ***11*** maxillary tooth 8, 11 ***mpcf*** mesial paracingular fossa ***pri*** primary ridge ***sa*** surangular ***wf*** wear facet.

**Figure 42. F42:**
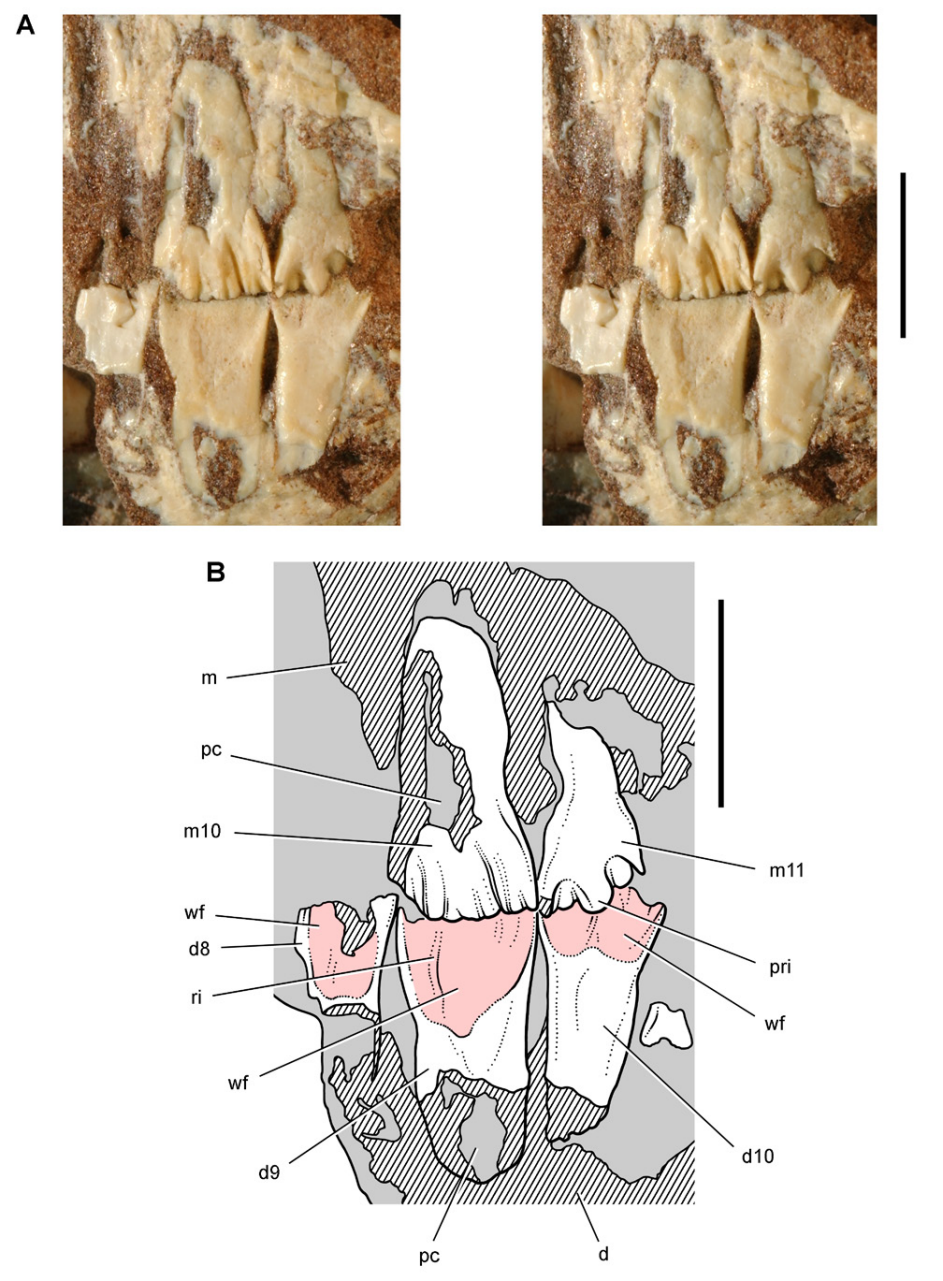
Posterior dentition of *Heterodontosaurus tucki* from the Lower Jurassic Upper Elliot and Clarens formations of South Africa. Tooth wear and replacement in posterior maxillary and dentary teeth in a juvenile skull (AMNH 24000). Stereopair (**A**) and line drawing (**B**) in left lateral view. Hatching indicates broken bone; grey tone indicates matrix; pink tone indicates wear facets. Scale bars equal 5 mm in **A** and **B**. Abbreviations: ***d*** dentary ***d8-10*** dentary tooth 8–10 ***m*** maxilla ***m10***, ***11*** maxillary tooth 10, 11 ***pc*** pulp cavity ***pri*** primary ridge ***ri*** ridge ***wf*** wear facet.

**Figure 43. F43:**
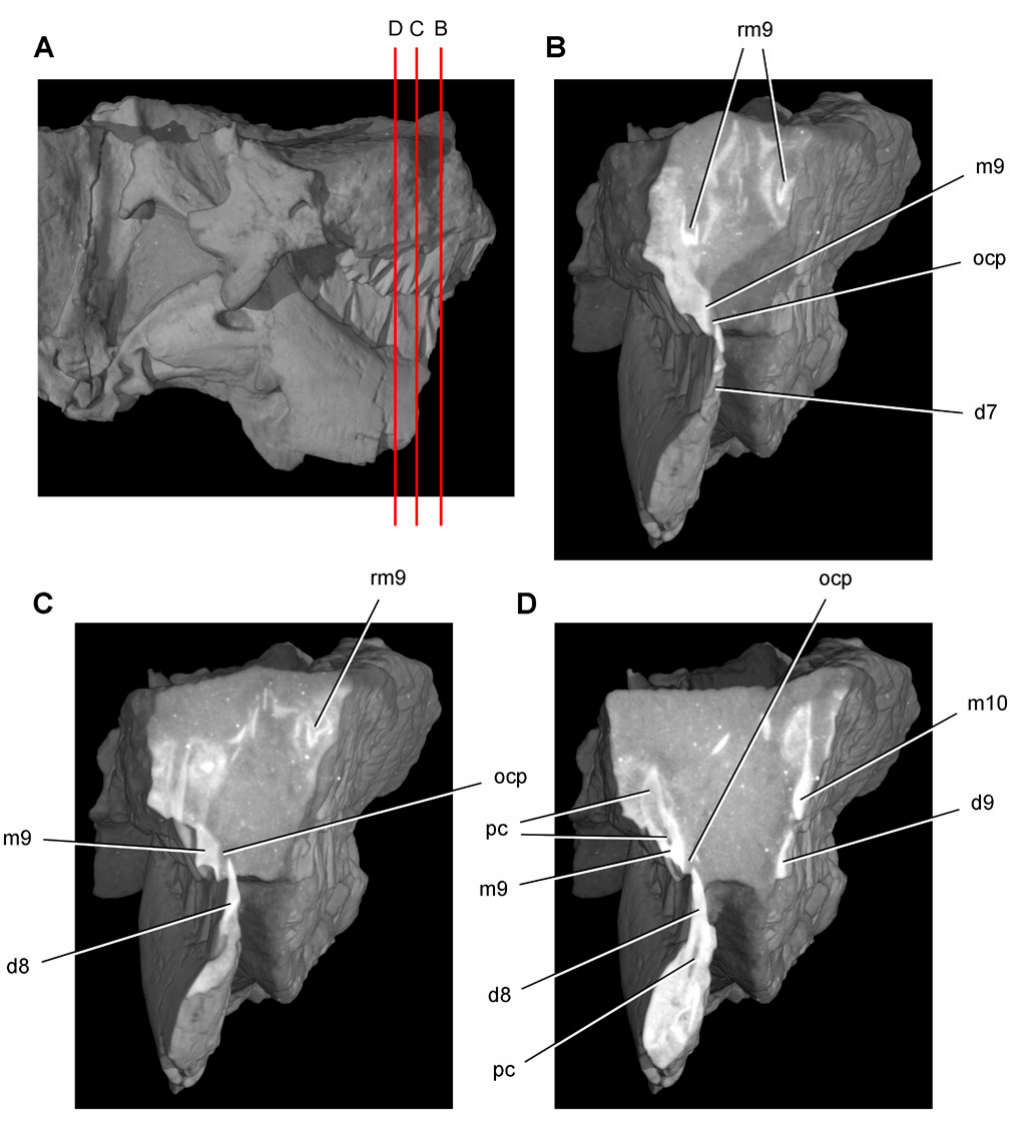
Tooth structure, occlusion, and replacement in *Heterodontosaurus tucki* from the Lower Jurassic Elliot and Clarens Formations of South Africa. Successive coronal computed-tomographic sections in cutaway view of a subadult skull (AMNH 24000). **A** Posterior portion of skull in right lateral view showing the location of coronal cross-sections **B** Cross-section through maxillary tooth 9 and dentary tooth 7 **C, D** Cross-sections through maxillary tooth 9 and dentary tooth 8. Abbreviations: ***d7-9*** dentary teeth 7-9 ***m9***, ***10*** maxillary tooth 9, 10 ***ocp*** occlusal plane ***pc*** pulp cavity ***rm9*** replacement maxillary tooth 9.

**Figure 44. F44:**
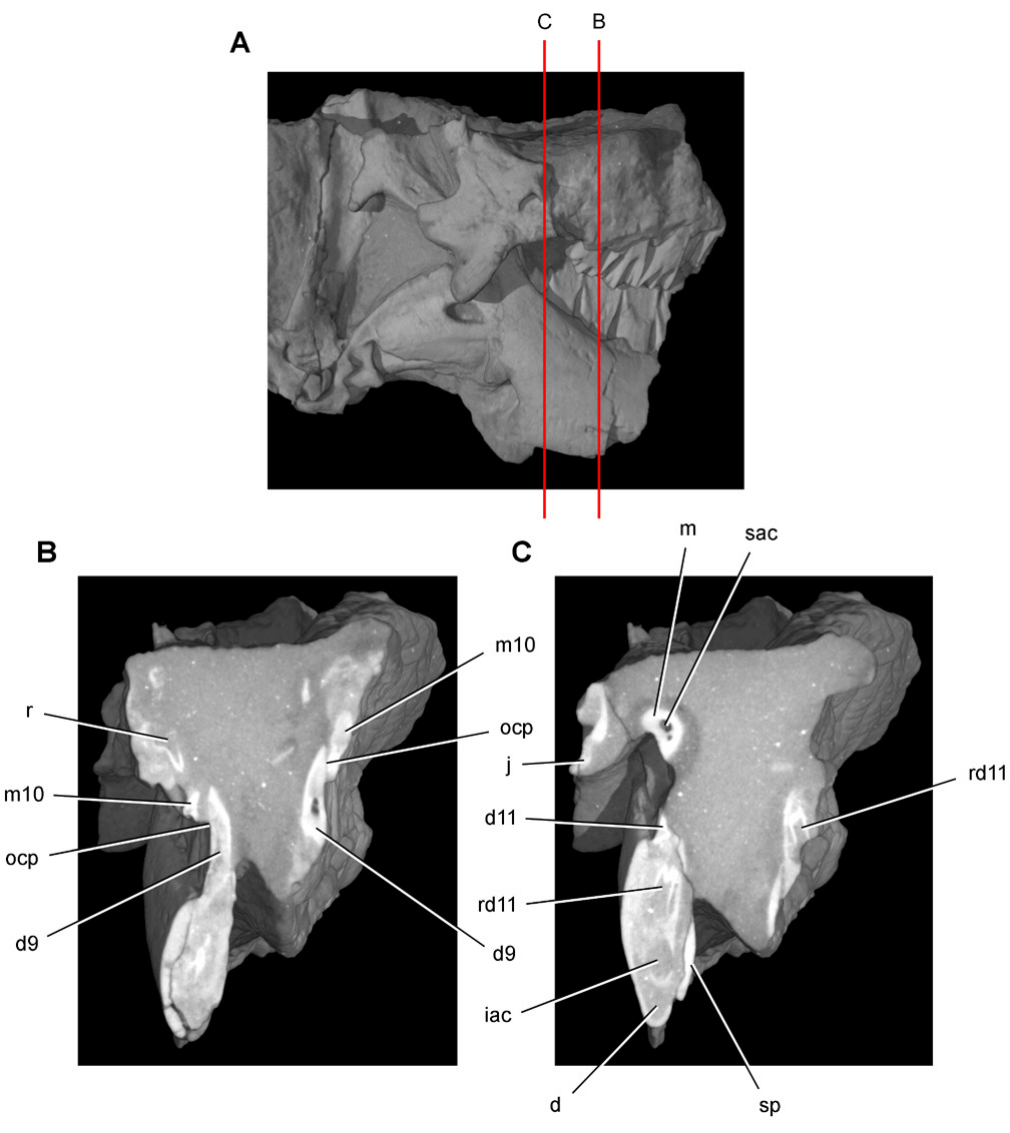
Tooth structure, occlusion, and replacement in *Heterodontosaurus tucki* from the Lower Jurassic Elliot and Clarens Formations of South Africa.Successive coronal computed-tomographic sections in cutaway view of a subadult skull (AMNH 24000). **A** Posterior portion of skull in right lateral view showing the location of coronal cross-sections **B** Cross-section through maxillary tooth 10 and dentary tooth 9 **C** Cross-section through dentary tooth 11. Abbreviations: ***d*** dentary ***d9***,***11*** dentary tooth 9, 11 ***iac*** inferior alveolar canal ***j*** jugal ***m*** maxilla ***m10*** maxillary tooth 10 ***ocp*** occlusal plane ***r*** replacement tooth ***rd11*** replacement dentary tooth 11 ***sac*** superior alveolar canal ***sp*** splenial.

**Figure 45. F45:**
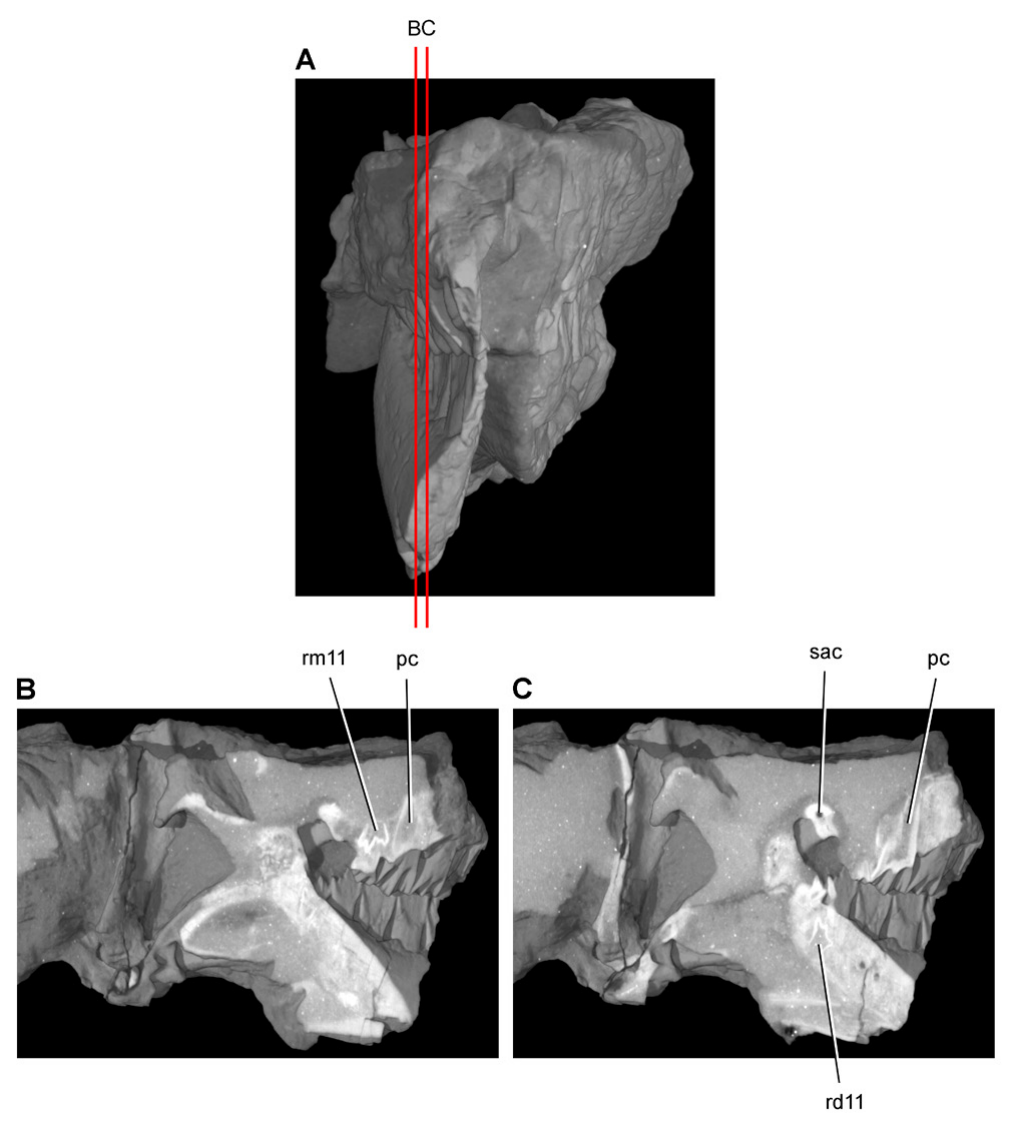
Tooth structure, occlusion, and replacement in *Heterodontosaurus tucki* from the Lower Jurassic Elliot and Clarens Formations of South Africa.Successive sagittal computed-tomographic sections in cutaway view of a subadult skull (AMNH 24000). **A** Posterior portion of skull in anterior view showing the location of sagittal cross-sections **B, C** Cross-sections in right lateral view through right maxillary and dentary rami. Abbreviations: ***pc*** pulp cavity ***rd11*** replacement dentary tooth 11 ***rm11*** replacement maxillary tooth 11 ***sac*** superior alveolar canal.

**Figure 46. F46:**
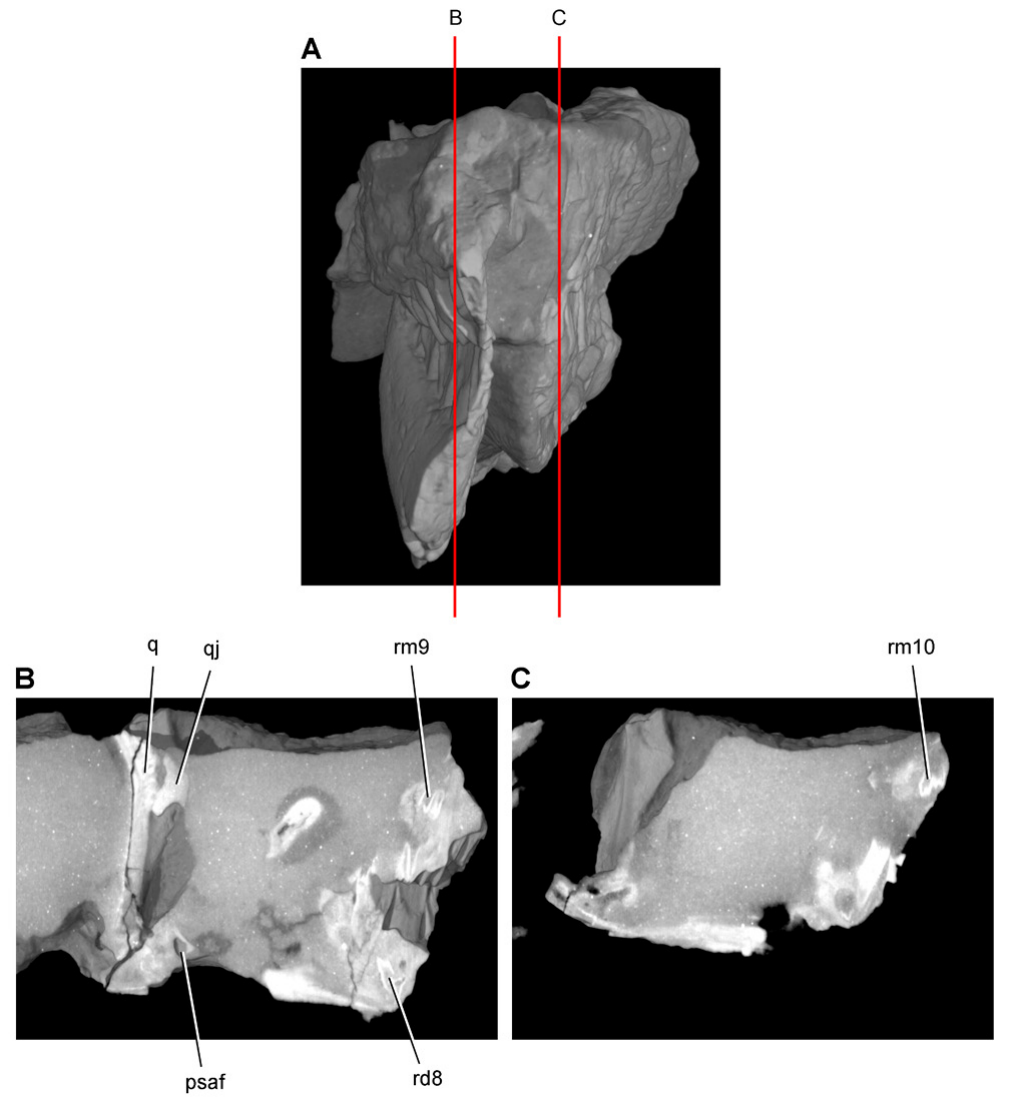
Tooth structure, occlusion, and replacement in *Heterodontosaurus tucki* from the Lower Jurassic Elliot and Clarens Formations of South Africa. Successive sagittal computed-tomographic sections in cutaway view of a subadult skull (AMNH 24000). **A** Posterior portion of skull in anterior view showing the location of sagittal cross-sections **B** Cross-section in right lateral view through right maxillary and dentary rami **C** Cross-section in right lateral view through left maxillary and dentary rami. Abbreviations: ***psaf*** posterior surangular foramen ***q*** quadrate ***qj*** quadratojugal ***rd8*** replacement dentary tooth 8 ***rm9***,***10*** replacement maxillary teeth 9, 10.

**Figure 47. F47:**
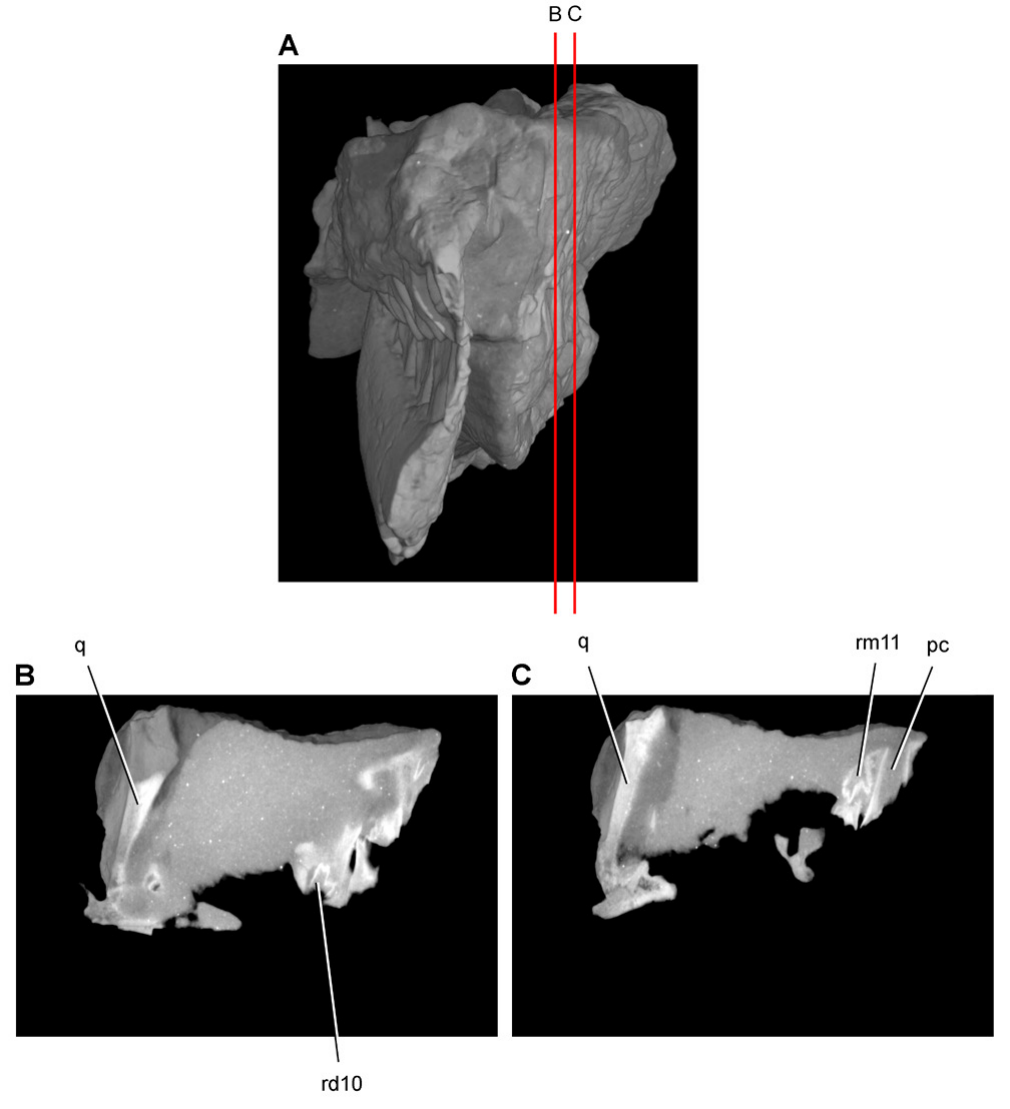
Tooth structure, occlusion, and replacement in *Heterodontosaurus tucki* from the Lower Jurassic Elliot and Clarens Formations of South Africa. Successive sagittal computed-tomographic sections in cutaway view of a subadult skull (AMNH 24000). **A** Posterior portion of skull in anterior view showing the location of sagittal cross-sections **B, C** Cross-sections in right lateral view through left maxillary and dentary rami. Abbreviations: ***pc*** pulp cavity ***q*** quadrate ***rd10*** replacement dentary tooth 10 ***rm11*** replacement maxillary tooth 11.

**Figure 48. F48:**
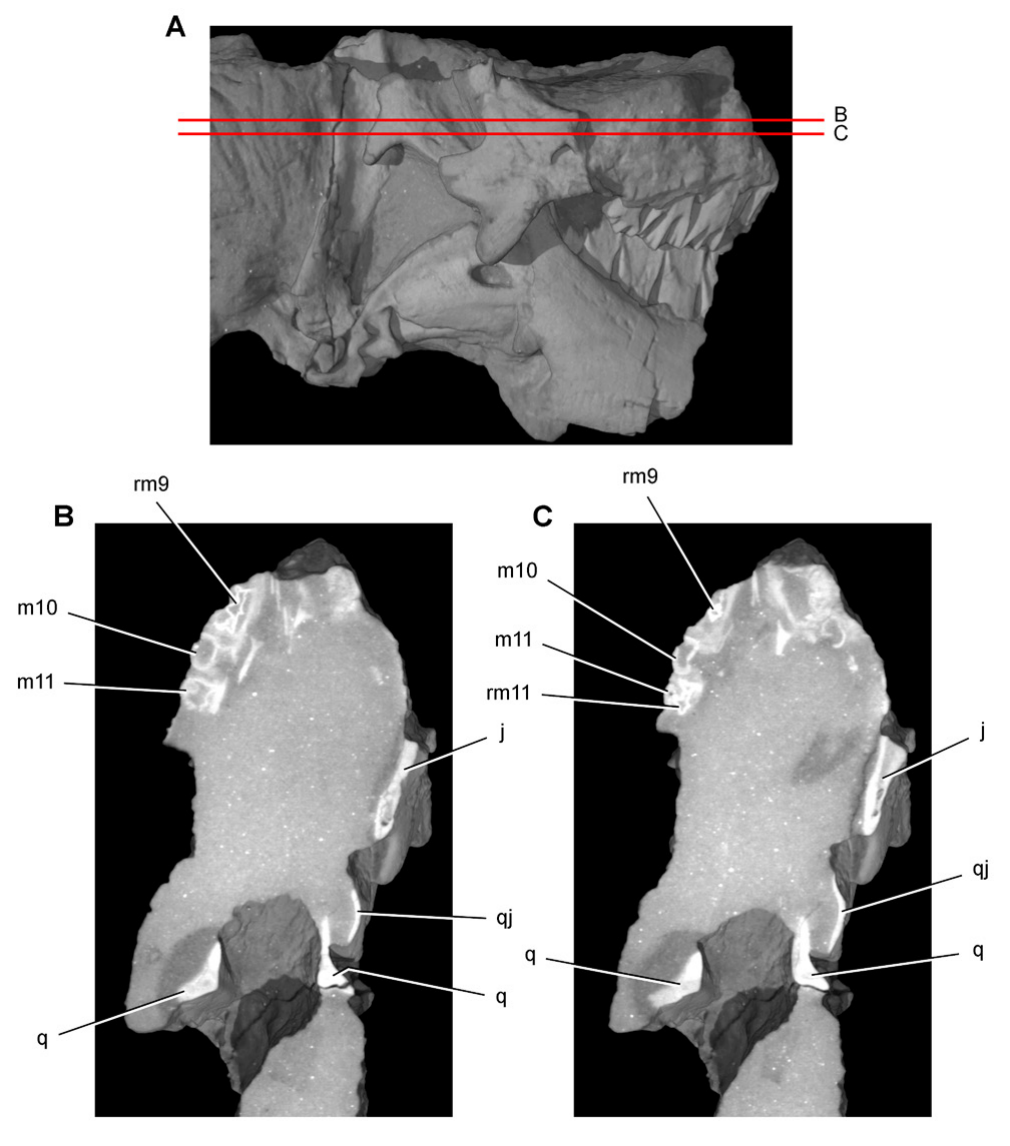
Tooth structure, occlusion, and replacement in *Heterodontosaurus tucki* from the Lower Jurassic Elliot and Clarens Formations of South Africa.Successive horizontal computed-tomographic sections in cutaway view of a subadult skull (AMNH 24000). **A** Posterior portion of skull in right lateral view showing the location of horizontal cross-sections **B, C** Cross-sections (anterior toward top of page) through the maxilla. Abbreviations: ***j*** jugal ***m10***,***11*** maxillary tooth 10, 11 ***q*** quadrate ***qj*** quadratojugal ***rm9***,***11*** replacement maxillary tooth 9, 11.

**Figure 49. F49:**
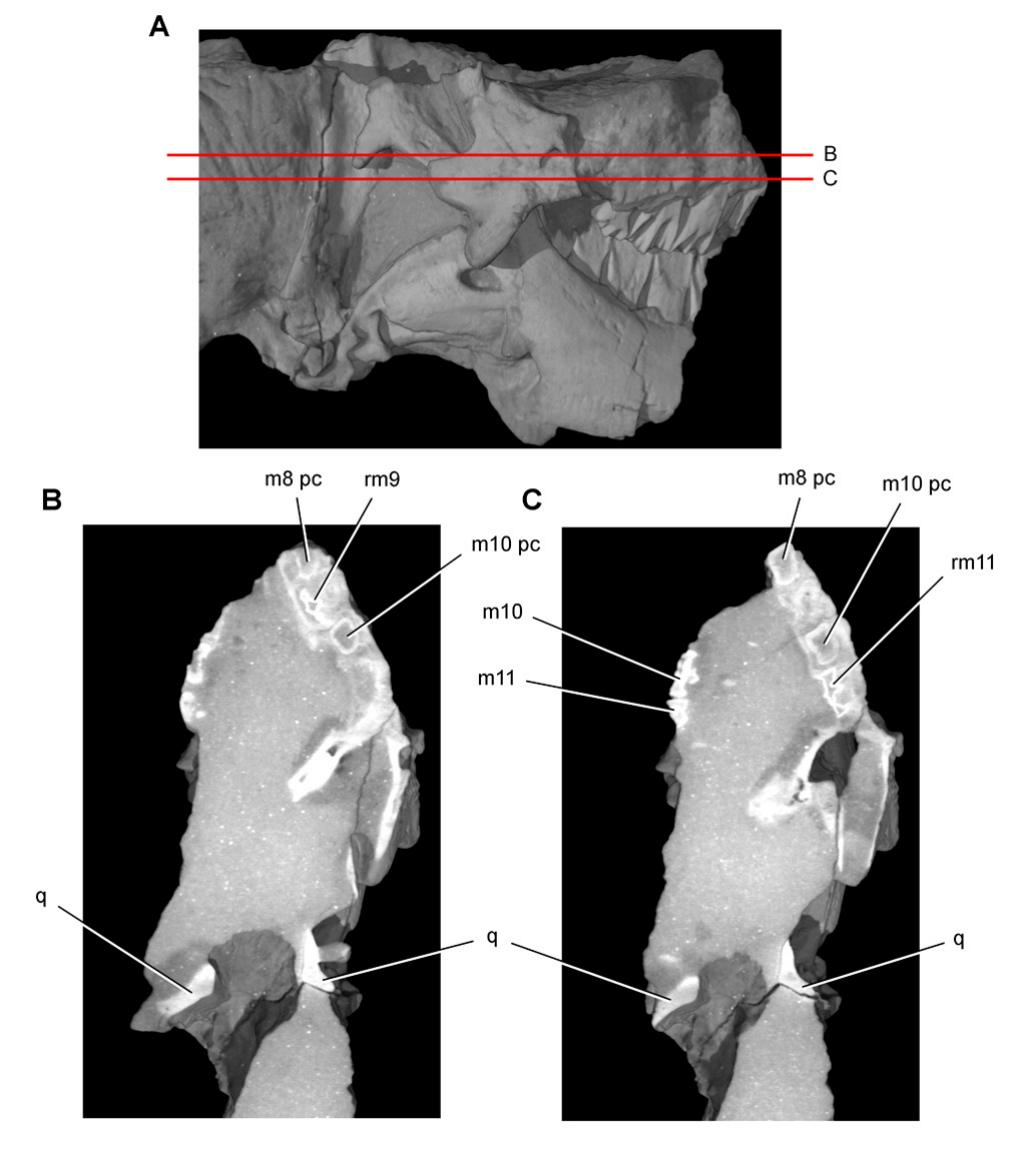
Tooth structure, occlusion, and replacement in *Heterodontosaurus tucki* from the Lower Jurassic Elliot and Clarens Formations of South Africa. Successive horizontal computed-tomographic sections in cutaway view of a subadult skull (AMNH 24000). **A** Posterior portion of skull in right lateral view showing the location of horizontal cross-sections **B** Cross-section (anterior toward top of page) through the maxilla **C** Cross-section (anterior toward top of page) through occluding portions of maxillary tooth rows.Abbreviations: ***m8***,***10**, **1**1* maxillary tooth 8, 10, 11 ***pc*** pulp cavity ***q*** quadrate ***rm9***,***11*** replacement maxillary tooth 9, 11.

**Figure 50. F50:**
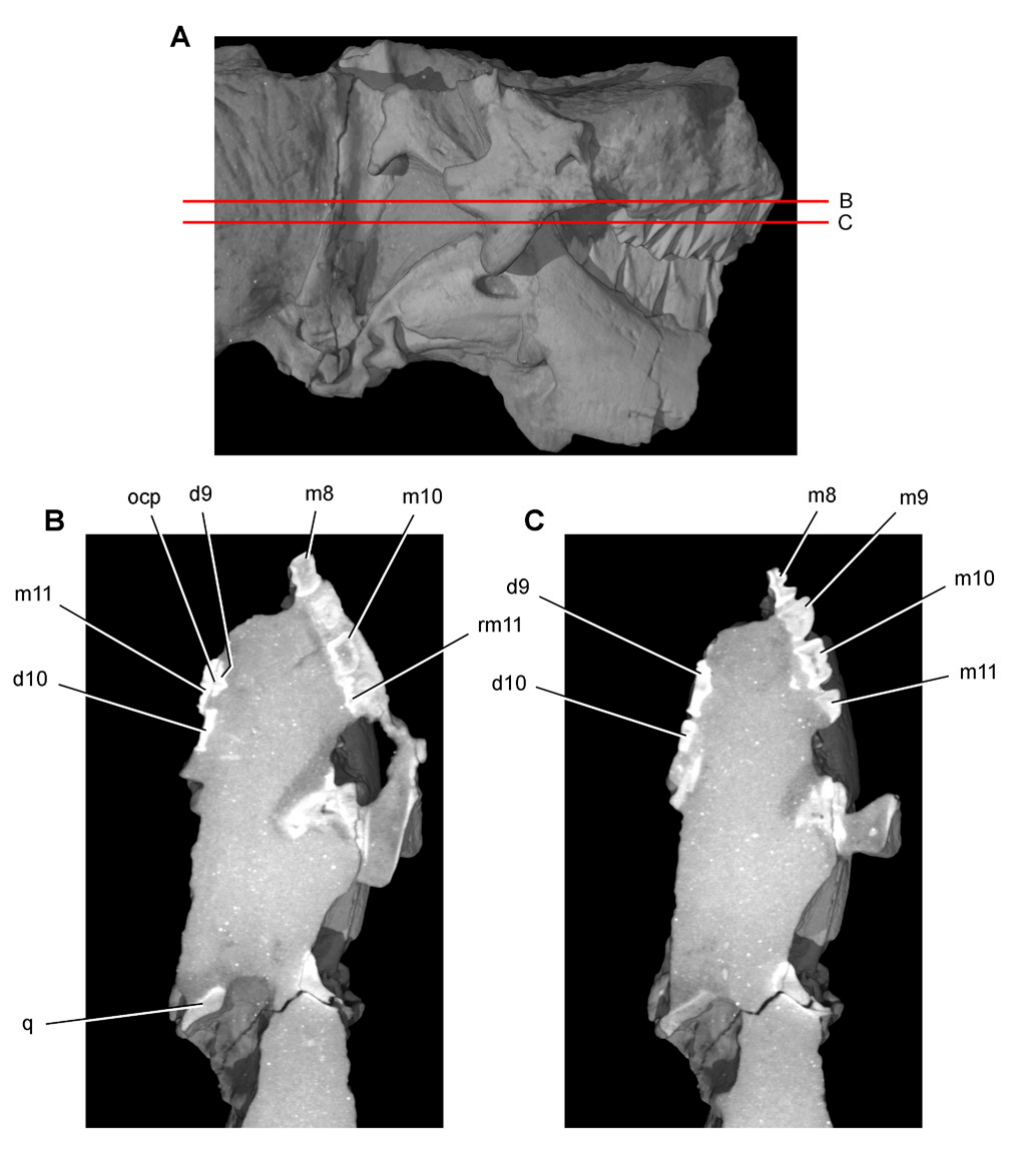
Tooth structure, occlusion, and replacement in *Heterodontosaurus tucki* from the Lower Jurassic Elliot and Clarens Formations of South Africa.Successive horizontal computed-tomographic sections in cutaway view of a subadult skull (AMNH 24000). **A** Posterior portion of skull in right lateral view showing the location of horizontal cross-sections **B, C** Cross-sections (anterior toward top of page) through occluding portions of maxillary and dentary tooth rows.Abbreviations: ***d9***, ***10***, dentary tooth 9, 10 ***m8-11*** maxillary teeth 8-11 ***ocp*** occlusal plane ***q*** quadrate ***rm11*** replacement maxillary tooth 11.

**Figure 51. F51:**
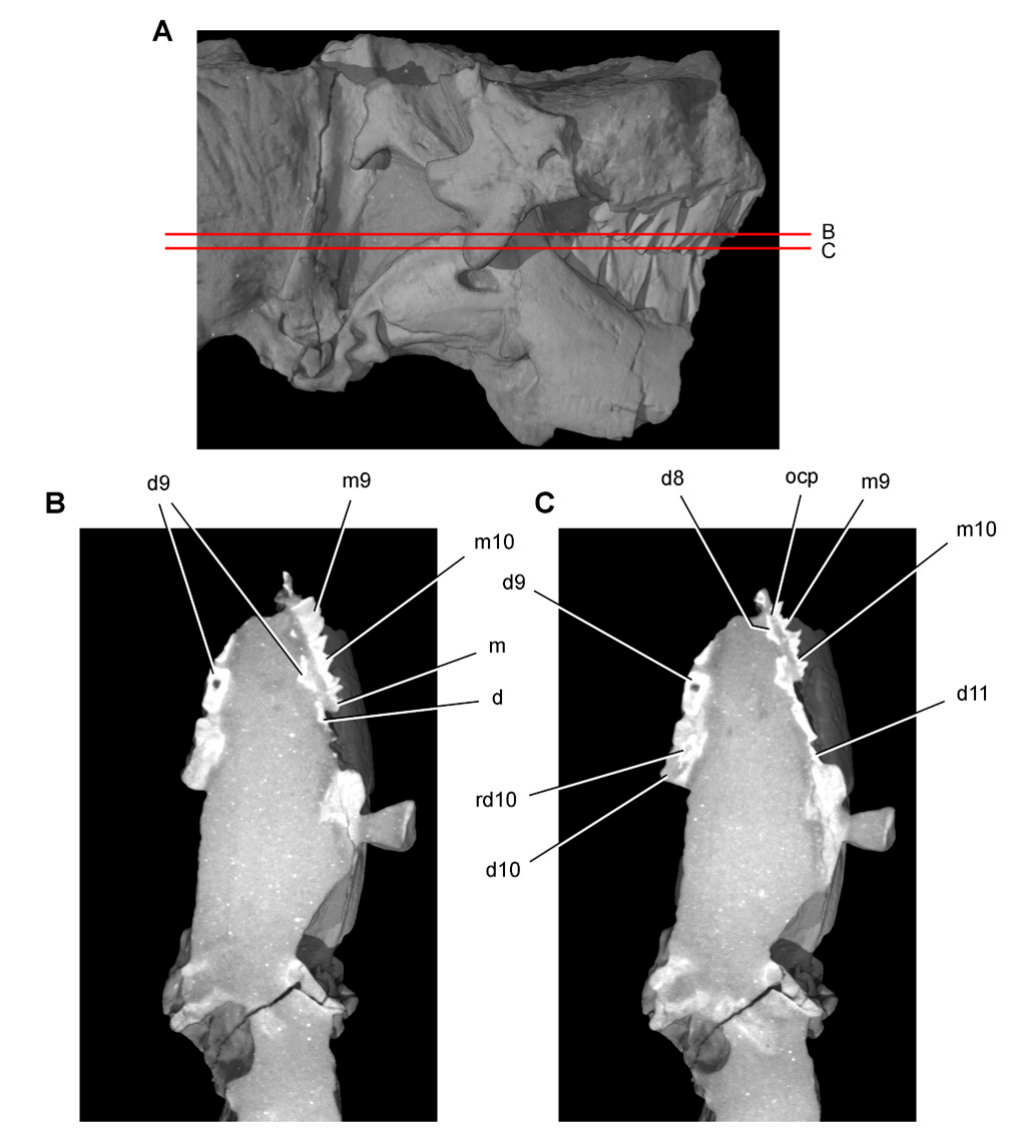
Tooth structure, occlusion, and replacement in *Heterodontosaurus tucki* from the Lower Jurassic Elliot and Clarens Formations of South Africa. Successive horizontal computed-tomographic sections in cutaway view of a subadult skull (AMNH 24000). **A** Posterior portion of skull in right lateral view showing the location of horizontal cross-sections **B, C** Cross-sections (anterior toward top of page) through occluding portions of maxillary and dentary tooth rows.Abbreviations: ***d*** dentary ***d8-11*** dentary teeth 8-11 ***m*** maxilla ***m9***,***10*** maxillary tooth 9, 10 ***ocp*** occlusal plane ***rd10*** replacement dentary tooth 10.

**Figure 52. F52:**
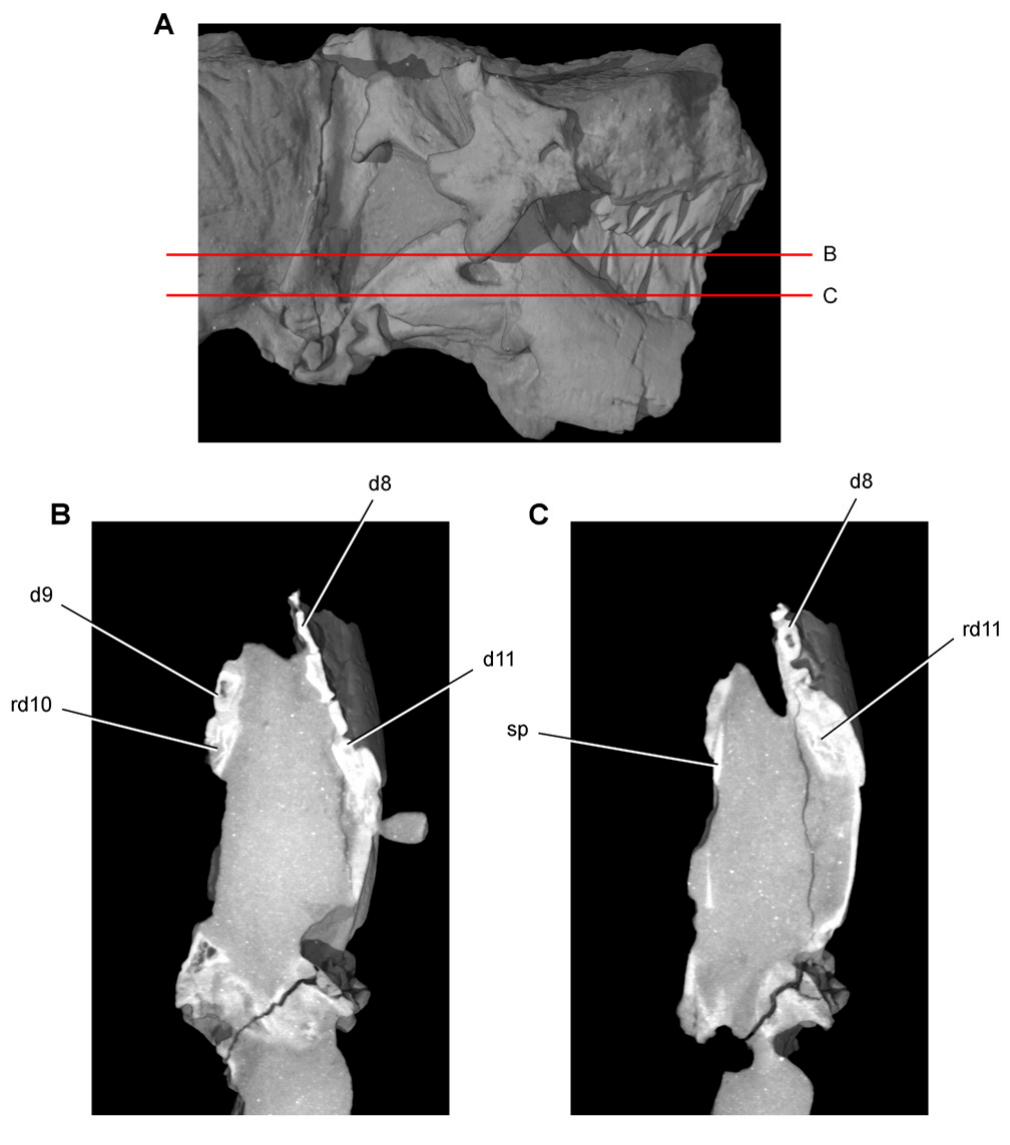
Tooth structure, occlusion, and replacement in *Heterodontosaurus tucki* from the Lower Jurassic Elliot and Clarens Formations of South Africa. Successive horizontal computed-tomographic sections in cutaway view of a subadult skull (AMNH 24000). **A** Posterior portion of skull in right lateral view showing the location of horizontal cross-sections **B, C** Cross-sections (anterior toward top of page) through the dentary. Abbreviations: ***d8***,***9**, **1**1* dentary tooth 8, 9, 11 ***rd10***,***11*** replacement dentary tooth 10, 11 ***sp*** splenial.

The aim of the following descriptive comments is to correct and extend where needed available accounts of the skull and skeleton of the best-known heterodontosaurid, *Heterodontosaurus tucki*. Initial accounts of the skull of *Heterodontosaurus* were based on two specimens, the holotype (SAM-PK-K337; Crompton and Charig 1962) and a better preserved referred specimen (SAM-PK-K1332; Charig and Crompton 1974; Weishampel 1984). Since that time, additional specimens have been prepared including an adult skull fragment containing a left maxilla (SAM-PK-K1334; Norman et al. 2011: Figs 30-33), the snout end of a subadult skull (SAM-PK-K10487; Butler 2008; Figs 38, 39), and the posterior half of another subadult skull (AMNH 24000; Figs 2C, 40-52, 61). All of these specimens except AMNH 24000 were considered in a recent more detailed description of the skull (Norman et al. 2011). The comments below extend that description and discuss aspects of cranial morphology where the interpretation here (Fig. 59) differs from either their text or figures.

The postcranium was originally described on the basis of a single nearly complete skeleton (SAM-PK-K1332; Santa Luca et al. 1976; Santa Luca 1980; Table 1). Postcranial bones originally associated with the holotype (SAM-PK-K337; Crompton and Charig 1962) apparently have been lost (Norman et al. 2011). Postcranial bones of a second adult individual were also collected and some of these are described below to document variation in coossification (SAM-PK-K1328; Fig. 70). Comments on the postcranium outline several anatomical areas where my interpretations differ from either the text or figures in Santa Luca (1980).

##### Cranium.

The premaxilla, best preserved in SAM-PK-K1332, exhibits several unusual features that also characterize other heterodontosaurids. The narial fossa is deep ventral to the external nares, and the fossa extends close to the alveolar margin of the premaxilla (Figs 57A, 59). The fossa was shown somewhat farther from the alveolar margin in some previous reconstructions (Norman et al. 2011; Fig. 58). In *Lesothosaurus* (Sereno, 1991: fig. 6C), in contrast, the narial fossa is shallow ventral to the external nares, and the external nares are positioned closer to the alveolar margin.

The internarial bar is broken in all specimens preserving the end of the snout, as reported by Norman et al. (2011). The internarial bar in SAM-PK-K1332, however, was more complete as originally preserved (Fig. 90A; compare Norman et al. 2011: append. 4A). Both premaxillary and nasal internarial processes extend toward one another, but their tips are broken. The internarial bar, thus, is depicted here as broken (Fig. 57A, 59). Norman et al. (2011: 20) suggested the opposing internarial processes were complete and separated by a gap (Fig. 57B, 58). Maintenance of at least a slender internarial bar, as preserved in *Tianyulong* (Figs 9C, 22, 23), is the common condition among nonavian archosaurs. An incomplete internarial bar, on the other hand, occurs commonly in mammals in association with more extensive development of narial soft tissues (narial cartilage, snout musculature; Witmer et al. 1999).

The posterolateral process of the premaxilla is quite broad at its base dorsal to the diastema in *Heterodontosaurus*, broader than the width between the narial fossa and anterior margin of the diastema. As a result, the posterolateral process appears somewhat swollen at its base in *Heterodontosaurus*, as also seen in *Abrictosaurus* and *Tianyulong* (Figs 23, 35). The posterior extremity of the posterolateral process was initially reconstructed as contacting the palpebral (Fig. 57A). Later reconstructions show it slightly shorter but inserting between the prefrontal and lacrimal (Fig. 57B) or coming to a point contact with the prefrontal (Fig. 58). The best-preserved specimen suggests the second option, that the process terminates between the lacrimal and prefrontal (Fig. 59), excluding external nasal-lacrimal contact as occurs in euornithopods.

The maxilla forms the ventral and anterodorsal borders of the triangular external antorbital fenestra (Figs 59, 90). The large invaginated opening on the snout sidewall is identified here as the *external antorbital fenestra* (following Witmer 1997), which is backed by an inset lamina of bone. The partially enclosed recess is termed the *antorbital fossa*, which is bounded by the maxilla, lacrimal and jugal. Two fenestrae within the antorbital fossa include the *internal antorbital fenestra* (= “antorbital fenestra”, Norman et al. 2011: 204), bounded by the maxilla and lacrimal, and a more anteriorly positioned opening here termed the *accessory antorbital fenestra* (= “anterior maxillary fenestra”, Norman et al. 2011: 204), which resembles an accessory opening of similar position in *Hypsilophodon* (Galton, 1974a: fig. 4). The accessory antorbital opening is fully exposed only in SAM-PK-K1332, where it is positioned on the right side a little more dorsolaterally under the rim of the external antorbital fenestra as compared to the left side. The dorsal margin of the fenestra, which is isolated in the maxilla, is rounded and beveled, suggesting that the opening may have served a pneumatic function for extension of an air sac from the nasal cavity into the antorbital fossa.

In addition to these openings, there is an additional blind recess within the antorbital fossa near its anterior corner, here labeled the *promaxillary fossa* (Fig. 90). In its location and anterior extension, this antorbital invagination resembles the promaxillary fossa previously described among theropods (Witmer 1997). Whether these structures are homologous remains an open question, as an invagination in a similar location is not known among basal sauropodomorphs such as *Eoraptor* (Martinez et al. 2011). In computed-tomographic scans of the skull of *Heterodontosaurus*, Norman et al. (2011: 204) observed an “opening” in the anterior corner of the antorbital fossa. This is probably the result of damage and loss after the original molding of the skull in question (SAM-PK-K1332). A blind recess is present in the anterior corner of the antorbital fossa as originally preserved (Fig. 90B).

The ventral border of the external antorbital fenestra is straight, relatively sharp-edged, and strongly everted, as in all heterodontosaurids preserving this region of the maxilla (Figs 59, 90). The rim around the anterior corner of the fenestra, by contrast, is slightly swollen. The buccal emargination extends anteriorly to the arched diastema ventral to the everted rim. Some reconstructions have depicted the ventral rim of the antorbital fossa as more rounded (Figs 57B, 58) or have shown the buccal emargination tapering toward the anterior end of the tooth row (Fig. 58) rather than extending anteriorly to the edge of the arched diastema.

The posteroventral corner of the antorbital fossa extends onto the jugal below the orbit, tapering to an end at the base of the jugal horn (Figs 57A, 59). The fossa on the jugal has been depicted previously as either considerably shorter (Fig. 57B) or longer, extending onto the jugal horn (Fig. 58). Likewise, various reconstructions have been given for the suture between the jugal and lacrimal along the orbital margin and the contribution of the jugal to the boundary of the external antorbital fenestra and wall of the antorbital fossa (Figs 57, 58). This region is best preserved in two specimens (SAM-PK-K1332, -K1334). The lacrimal-jugal suture is a scarf joint similar to that in many ornithischians; the jugal border of the external antorbital fenestra forms its arcuate posteroventral corner; and the jugal contribution to the medial wall of the antorbital fossa is limited to the extension of the fossa onto the external surface of the jugal (Fig. 59).

In dorsal view of the snout, a median fossa is present on the nasals (median “sulcus”; Norman et al. 2011: 202) in both subadult and adult skulls (SAM-PK-K1332, -K10487). This fossa, bounded on each side by a rounded rim formed by the nasals, characterizes all heterodontosaurids that preserve this portion of the skull as well as several other basal ornithischians such as *Agilisaurus* (Peng 1992). A subtriangular fossa with no apparent connection to the antorbital fossa is also present on the lateral aspect of the lacrimal (Fig. 59), a depression regarded here as a diagnostic character for *Heterodontosaurus tucki*.

The jugal horn has a flattened subrectangular shape and projects laterally (Fig. 59). The horn has been variously depicted as subconical (Fig. 58) or as directed ventrolaterally (Fig. 57). The postorbital has a broad fossa that excavates most of the lateral surface of the ventral and posterior rami (Fig. 59). The shape of these rami varies among heterodontosaurids. The posterior ramus in *Heterodontosaurus* is particularly slender (Fig. 59), although it has been reconstructed with broader proportions (Figs 57, 58).

The quadrate head is not expanded transversely in lateral view, nor is there a gap between the quadrate shaft and the squamosal and quadratojugal as sometimes reconstructed (Fig. 57). In lateral view of the skull, the ventral portion of the occipital condyle and basal tubera are visible posterior to the quadrate shaft and ventral to the paroccipital process, as preserved on the right side of SAM-PK-K1332 (Fig. 59). Some skull reconstructions have shown neither of these structures in lateral view or only the occipital condyle (Figs 57, 58).

The form of the jaw joint carried particular functional significance and is well preserved in SAM-PK-K1332 and AMNH 24000. In both specimens, an axis through the quadrate condyles angles anteromedially at about 45° as seen in ventral view (Figs 61C, 92B). In posterior view, the ventral articular surface of the quadrate condyles also angles approximately 45° ventrolaterally (Weishampel 1984: fig. 3a; Fig. 92A). The articular cup, or cotylus, for the quadrate condyles is deeply concave and shaped with a snug fit, such that fore-aft (propalinal) movement of the condyles relative to the lower jaw would not have been possible. The lateral edge of the cotylus, likewise, is prominent and shaped to receive the bulbous lateral condyle of the quadrate (Fig. 61C, D). Lateral movement of the condyles relative to the lower jaw thus would not have been possible. Previous reconstructions have shown the articular cup as more broadly open or loosely fitted to the condyles in a way that might allow either fore-aft or transverse movement of the quadrate relative to the lower jaw (Figs 57, 58).

##### Lower jaw.

The predentary is smaller relative to the anterior end of the dentary, which is expanded dorsoventrally (Figs 59-61). The predentary is triangular in lateral view, approximately as long anteroposteriorly as deep dorsoventrally, with sharp lateral margins and a rounded ventral keel. In dorsal view, the predentary is also triangular in shape and sharply pointed anteriorly. The lateral and ventral processes of the predentary are best understood only when the element is completely exposed (SAM-PK-K1332). The ventral process is the most reduced, extending as a short point in the midline that is not otherwise differentiated from the body of the predentary (Fig. 61B). The lateral processes extend posterodorsally as short prongs with a subtriangular cross-section. Their distal tips are positioned just anterior to the caniniform tooth (Figs 59, 60B). There is a deep median trough between the lateral processes, which gives the predentary a V-shaped cross-section at mid-length (Fig. 60B). Thus the predentary in *Heterodontosaurus* is not a solid wedge-shaped bone, but rather one with short lateral processes, a rudimentary ventral process, and a trough-shaped oral surface.

A well-defined, smooth articular surface for the predentary is present on the anterior end of the dentary, which is well preserved in SAM-PK-K1332 and SAM-PK-K10487 (Fig. 59). This surface is strongly concave anteroposteriorly and gently convex dorsoventrally (Figs 39, 59). These opposing curvatures create a smooth, vertically deep, saddle-shaped articular surface for the predentary. This surface is broadest anteroposteriorly at mid height, where the dentary projects anteriorly to a blunt end (Fig. 39). The predentary-dentary joint has been erroneously described as “spheroidal” (Weishampel 1984: 47). A fragment has been lost from the anterior end of the left dentary of SAM-PK-K1332, leaving an angular breakage surface, which has been erroneously interpreted as a depression for articulation with the predentary (Weishampel 1984: fig. 3f). A raised subtriangular area, here identified as the *dentary boss*, is present on the ventral aspect of the dentary immediately behind the predentary articulation and below the anterior dentary foramen (Figs 59, 61B).

The dentary symphysis is restricted to the ventral portion of the dentary, and is slightly thickened and rugose (Fig. 61A). It has an asymmetrical V-shape due to a small posterior embayment representing the anterior terminus of Meckel’s canal, a narrow trough near the ventral margin of the dentary ramus (Figs 56, 61A). The symphysis has been drawn and reconstructed as extending across most or all of the anterior end of the dentary (Norman et al. 2011: Figs 18B, 19B) rather than limited to its ventral one-half. The more restricted, ventrally positioned symphysis may well have important functional ramifications regarding potential long-axis rotation of the dentary during mastication.

Between the symphysis and the base of the caniniform tooth is a dorsoventrally concave surface with large vascular foramen that runs anteriorly toward the predentary (Fig. 61A). This concave surface, when joined by its opposite, forms a spout-shaped trough above the symphysis that continues anteriorly onto a similar surface on the predentary (Fig. 60B). Like other ornithischians, the symphysis is located at the anterior extremity of the dentary, Meckel’s canal extends into the symphysis resulting in a V-shaped posterior embayment, and a trough-shaped surface is located above the symphysis that extends onto the predentary (Sereno 1991). The ornithischian “spout-shaped” symphyseal region, thus, is not absent as reported previously (Butler et al. 2007: 19) but is proportionately narrower at least in *Heterodontosaurus*. The symphyseal surface between the dentaries, in addition, is more substantial than in other basal ornithischians. The symphyseal region in *Heterodontosaurus*, in summary, shares some features with ornithischians but appears to be less mobile. The predentary-dentary articulation, on the other hand, appears to be more mobile in advanced heterodontosaurids such as *Heterodontosaurus*. The symphysis in *Heterodontosaurus* does not resemble the plesiomorphic saurischian condition. In basal saurischians such as *Eoraptor* (Martinez et al. 2011), the dentary rami typically approach one another at a narrow angle of incidence, and the symphyseal surface is barely differentiated from the medial aspect of the dentary.

The arched buccal emargination is deepest below the center of the dentary tooth row and extends from the base of the caniniform tooth anteriorly to the base of the coronoid process posteriorly (Figs 59, 60B). A strong coronoid process extends posterodorsally at about 45° and is overlapped medially by a coronoid bone, which is preserved in articulation in SAM-PK-K1332 and AMNH 24000 (Figs 41, 56). In basal ornithischians such as *Lesothosaurus*, the coronoid runs anteriorly along the alveolar margin as an elongate strap-shaped bone with a tongue-shaped posterior end that curves upward. It is only weakly developed for insertion of adductor musculature, is not visible in lateral view of the lower jaw, and has only incidental sutural contact with the surangular (Sereno 1991: fig. 13G). In heterodontosaurids, in contrast, the posterior portion of the coronoid is expanded dorsally, is exposed in lateral view of the lower jaw, and is sutured to the surangular (Fig. 56, 59). The coronoid, thus, is expanded as a site of attachment for the adductor musculature, similar to the condition in basal euornithopods such as *Hypsilophodon* (Galton 1974a: fig. 10).

A large oval depression, here termed the *external mandibular fossa*, is present on the angular and surangular, bordered above by a strong surangular ridge and below by the everted ventral margin of the angular (Fig. 59). The angular-surangular suture courses across the middle of the external mandibular fossa, which is present in many heterodontosaurids. There are two surangular foramina, anterior and posterior. The parallelogram-shaped anterior surangular foramen, which tends to be the larger of the pair in heterodontosaurids, is absent in the basal ornithischian *Lesothosaurus* but present in some euornithopods such as *Hypsilophodon* (Galton 1974a; Sereno 1991). Neurovascular grooves extend from both foramina across the surangular, the groove passing posteriorly from the anterior surangular foramen dissipating as it nears the jaw joint. These features are well preserved in a referred subadult skull (Figs 40, 41).

Norman et al. (2011) erroneously described several aspects of this portion of the lower jaw due to damage and coossification in specimen SAM-PK-K1332 (Fig. 58). The angular-surangular suture was drawn just under the swollen dorsal margin of the surangular, and the anterior surangular foramen and its groove were envisioned as a fissure separating a pair of processes with “unique” articular relations with the dentary (Norman et al. 2011: 187, 210). The ventral of the two processes was shown articulating laterally with an expanded dentary coronoid process (Fig. 58). The form and articular relations of the coronoid process of the dentary, surangular and angular, however, are much more conventional (Figs 40, 41, 59). The external mandibular fossa and the grooves emanating from the surangular foramina, however, may be related to more elaborate insertion of jaw musculature on the lateral aspect of the lower jaw in heterodontosaurids as discussed below.

The articulation for the quadrate is cup-shaped and fitted tightly to the lateral condyle of the quadrate. The external rim of the articular socket is everted and curled dorsally, rising to a low process immediately posterior to the posterior surangular foramen, as in *Lesothosaurus* and *Hypsilophodon*. There is one additional oval depression, here termed the *lateral retroarticular fossa*, which is located distal to the jaw articulation on the lateral aspect of the retroarticular process (Fig. 59). A fossa of similar form is not known elsewhere among basal ornithischians, although the condition in other heterodontosaurids remains unknown.

##### Premaxillary teeth.

The first two premaxillary teeth (pm1, 2) are preserved in the holotypic skull (Norman et al. 2011: fig. 20). The second premaxillary tooth (pm2) also was originally preserved in the referred adult skull (Figs 90A, 91) but subsequently has been lost (Norman et al. 2011). The gentle swelling of the base of the crown, limited recurvature, and reduced ornamentation in pm 1 and 2 closely resemble the mesial premaxillary teeth in other basal ornithischians such as *Lesothosaurus* (Sereno 1991). The mesial and distal carinae in these premaxillary teeth are not well exposed but appear to lack serrations as reported by Norman et al. (2011). Butler et al. (2008: 19) suggested that there is no swelling of the crown above the root in the premaxillary teeth of heterodontosaurids as occurs in other ornithischians. Although subtle, such swelling of the crown above the root is present in the mesial two premaxillary teeth in *Heterodontosaurus*, *Echinodon*, *Lycorhinus* and the Kayenta heterodontosaurid. Some authors (Crompton and Charig 1974; Weishampel 1984; Crompton and Attridge 1986; Weishampel and Witmer 1990) have figured or described a distinct heel or step on the distal or lingual side of the crown (Fig. 57), but none is present (Fig. 91).

The third premaxillary crown (pm3), in contrast to the mesial two, is caniniform with markedly greater size, more pronounced recurvature, and more lateral compression of the upper crown (Figs 59, 90, 91). Basal crown width (mesiodistal) and height, however, are slightly less than the crown of the dentary caniniform tooth. Skull reconstructions showing these opposing caniniform teeth in labial view as equal in width or height are erroneous (Fig. 57). The central axis of the caniniform crown is vertical. The mesial carina is convex, whereas the distal carina is nearly straight (Figs 90A, 91). At least the distal carina is serrate, with approximately six serrations per millimeter (Norman et al. 2011). The tip of the crown of pm3 is broken on both sides of the holotypic skull, with some abrasion rounding the edges of the broken surface.

##### Maxillary teeth.

There are 11 or 12 maxillary teeth in the best-preserved adult skulls. The small broken crown base of a twelfth tooth was shown on the right side of the holotypic specimen (Crompton and Charig 1962: fig. 1A), although there appears to be evidence in this specimen for only 11 maxillary teeth. Subadult skull AMNH 24000 shows that there is only one small maxillary crown at the distal end of the tooth row, as preserved on both sides (Fig. 40). On the right side of skull SAM-PK-K1332, however, it is not clear whether there are two small distal maxillary crowns or one (Fig. 55; labeled m11). A slightly larger adult skull with 12 dentary teeth does not preserve enough of the maxillary tooth row to provide a reliable count (NM QR 1788; Porro et al. 2011). Variation in adult cheek tooth count seems to have been minimal in advanced heterodontosaurids such as *Heterodontosaurus*.

The maxillary tooth row is not straight as reconstructed in ventral view by Norman et al. (2011: fig. 13). As best preserved in the adult cranium SAM-PK-K1332 (Norman et al. 2011: fig. 6B), the anterior two and posteriormost tooth in the maxillary tooth row diverge labially (Fig. 60A). The ends of the maxillary tooth row, thus, curve laterally and are matched by a similar more subtle curvature of the dentary tooth row (Fig. 60B). The curvature of the cheek tooth rows has important ramifications when considering possible occlusal mechanics.

Mesiodistal crown width increases gradually from m1 to m8 and then decreases slightly in m9 and m10 and then more so in m11 (Figs 55, 59). Tooth size, thus, precisely speaking, does not increase toward the “middle of the tooth row” in *Heterodontosaurus* but rather distal to the center of the tooth row (*contra* Weishampel 1984: 53), as reported by Norman et al. (2011). This pattern in crown size differential is consistent with that in other ornithischians such as *Lesothosaurus*, which typically have significantly smaller crowns at each end of the tooth row (Sereno 1991). By comparison, crown size differential in heterodontosaurids, is enhanced by the relatively low number of crowns in the cheek dentition (Fig. 59).

The maxillary and dentary crowns in *Heterodontosaurus*, unlike those of other heterodontosaurids, have flattened mesial and distal surfaces that butt against one another along the tooth row, as shown in horizontal computed tomographic cross sections (Fig. 48–52). Unworn maxillary and dentary crowns are fan-shaped, gently expanding mesiodistally towards a denticulate apical margin that slopes at a low angle in mesial and distal directions away from the apical denticle (Fig. 41; crown d11). A gentle mesiodistal constriction is present between the crown and its slightly swollen, hollow root (Fig. 42). *Heterodontosaurus*, however, lacks the discrete cingulum or swollen shoulder present in other heterodontosaurids such as *Echinodon*, *Lycorhinus* and *Abrictosaurus*.

The labial surface is dominated by a strong primary ridge that is flanked by mesial and distal paracingular fossae, the former extending slightly farther toward the crown base than the latter (Fig. 41). The primary ridge, which tends to be offset toward the mesial edge of the crown face, and mesial and distal marginal ridges create a W-shaped leading edge to the wear facets (Norman et al. 2011; Fig. 51). Secondary ridges extend from denticles to either side of the apical denticle. They taper to an end around mid height on the crown and are lost in heavily worn crowns. The distalmost maxillary crown appears to have more squat proportions, the secondary ridges almost reaching the base of the paracingular fossae (Fig. 41).

The lingual surface of mesial maxillary crowns is best exposed in SAM-PK-K1332, which have short primary and secondary ridges (Norman et al. 2011: fig. 24C). In the largest crowns toward the distal end of the tooth row, the primary ridge is flanked by paracingular fossae as on the labial side of the crown (Fig. 50C, m10). When a wear facet obliterates most or all of the lingual side of the crown, the central fossa on the basal portion of the root creates a concave margin in cross section (Fig. 49C, m10). Maxillary crowns are also canted lingually relative to the vertical axis of their roots (Figs 43D, 44B). The enamel thins across the flat mesial and distal surfaces of the crown. Computed tomographic sections suggest that a thin layer of enamel may be present on the lingual side of the maxillary crowns, which is often obliterated by tooth wear (Figs 48–52).

In occlusal view the maxillary crowns are butted against each other, the mesial margin often slightly concave and the distal margin flat or slightly convex in cross section (Figs 51B, 52B). Although generally aligned, the wear facets in adult cheek teeth in *Heterodontosaurus* do not form a seamless wear surface as noted by Norman et al. (2011). They do not form a “tooth battery” (*contra* Norman et al. 2011), the latter term reserved for tooth-supported dentitions, the roots of which are pressed against other crowns in an open alveolar trough. In *Heterodontosaurus* the roots of all cheek teeth are anchored within individual alveoli.

**Figure 53. F53:**
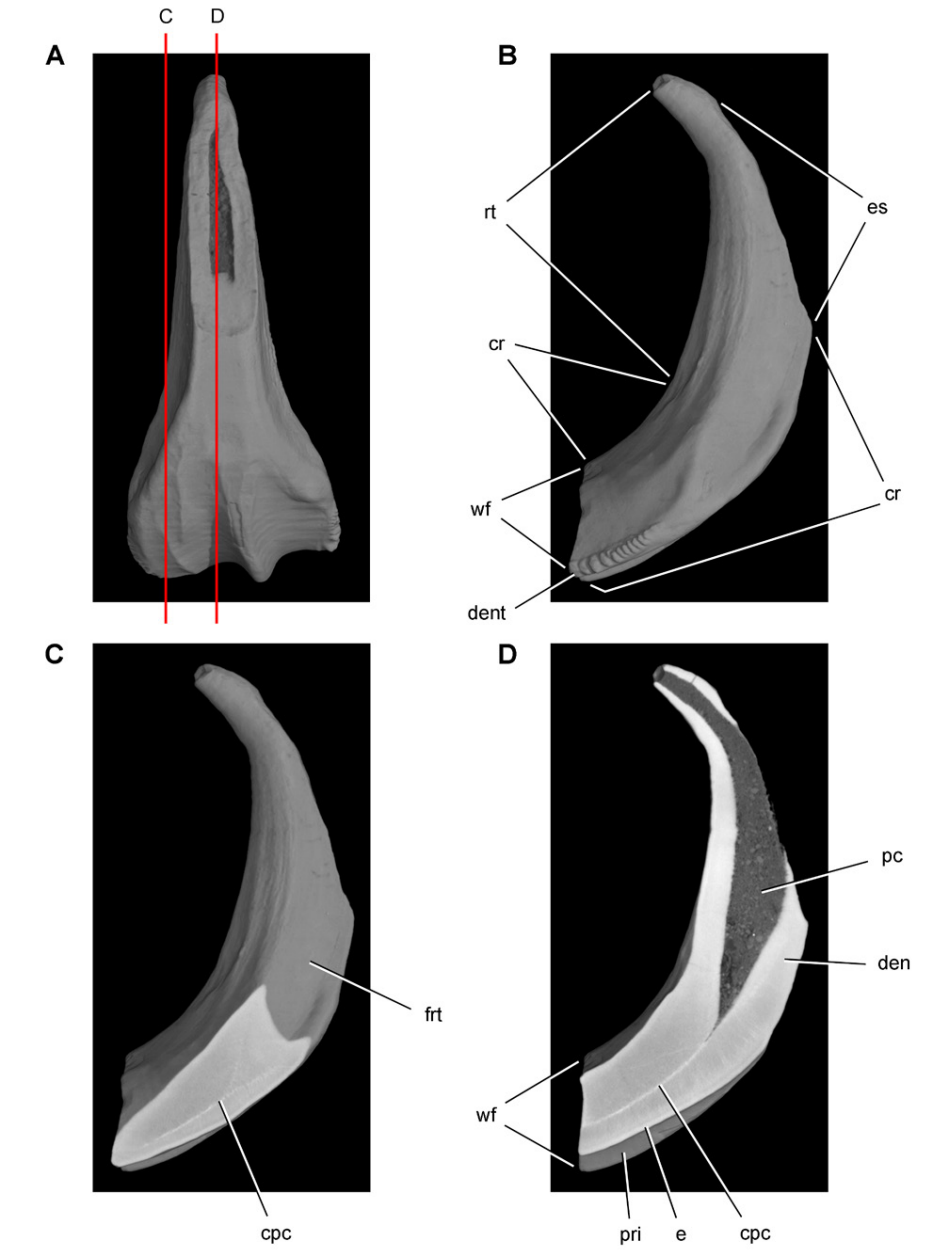
Tooth structure in *Ouranosaurus nigeriensis* from the mid-Cretaceous Elrhaz Formation of Niger. Successive coronal computed-tomographic sections in cutaway view of a worn maxillary tooth showing internal structure (MNBH GAD28) **A** Maxillary tooth in labial view showing the location of coronal cross-sections **B** Maxillary tooth in distal view showing the division between crown and root and the fossa for an adjacent replacement tooth **C** Section through mesial portion of crown **D** Section through mid section of crown. Abbreviations: ***cpc*** collapsed pulp cavity ***cr*** crown ***den*** dentine ***dent*** denticle ***e*** enamel ***es*** erosional surface ***frt*** fossa for replacement tooth ***pc*** pulp cavity ***pri*** primary ridge ***rt*** root ***wf*** wear facet.

**Figure 54. F54:**
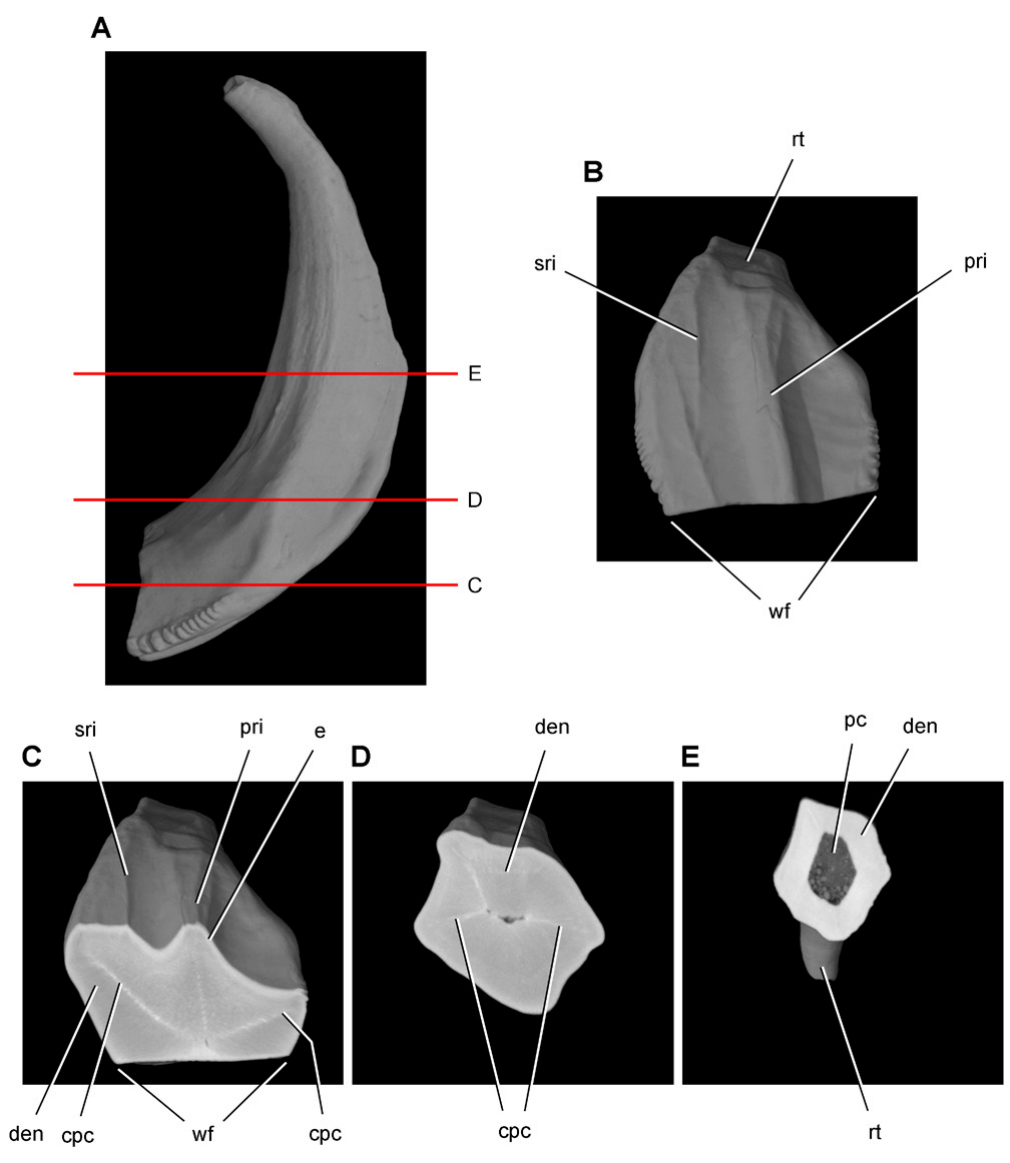
Tooth structure in *Ouranosaurus nigeriensis* from the mid-Cretaceous Elrhaz Formation of Niger. Successive horizontal computed-tomographic sections in cutaway view of a worn maxillary tooth showing internal structure (MNBH GAD28) **A** Maxillary tooth in distal view showing the location of horizontal cross-sections **B** Maxillary tooth in apical view (labial toward top of page) **C** Cross-section (labial toward top of page) through mid crown **D** Cross-section (labial toward top of page) through base of the crown **E** Cross-section (labial toward top of page) through proximal portion of the root. Abbreviations: ***cpc*** collapsed pulp cavity ***den*** dentine ***e*** enamel ***pc*** pulp cavity ***pri*** primary ridge ***rt*** root ***sri*** secondary ridge ***wf*** wear facet.

**Figure 55. F55:**
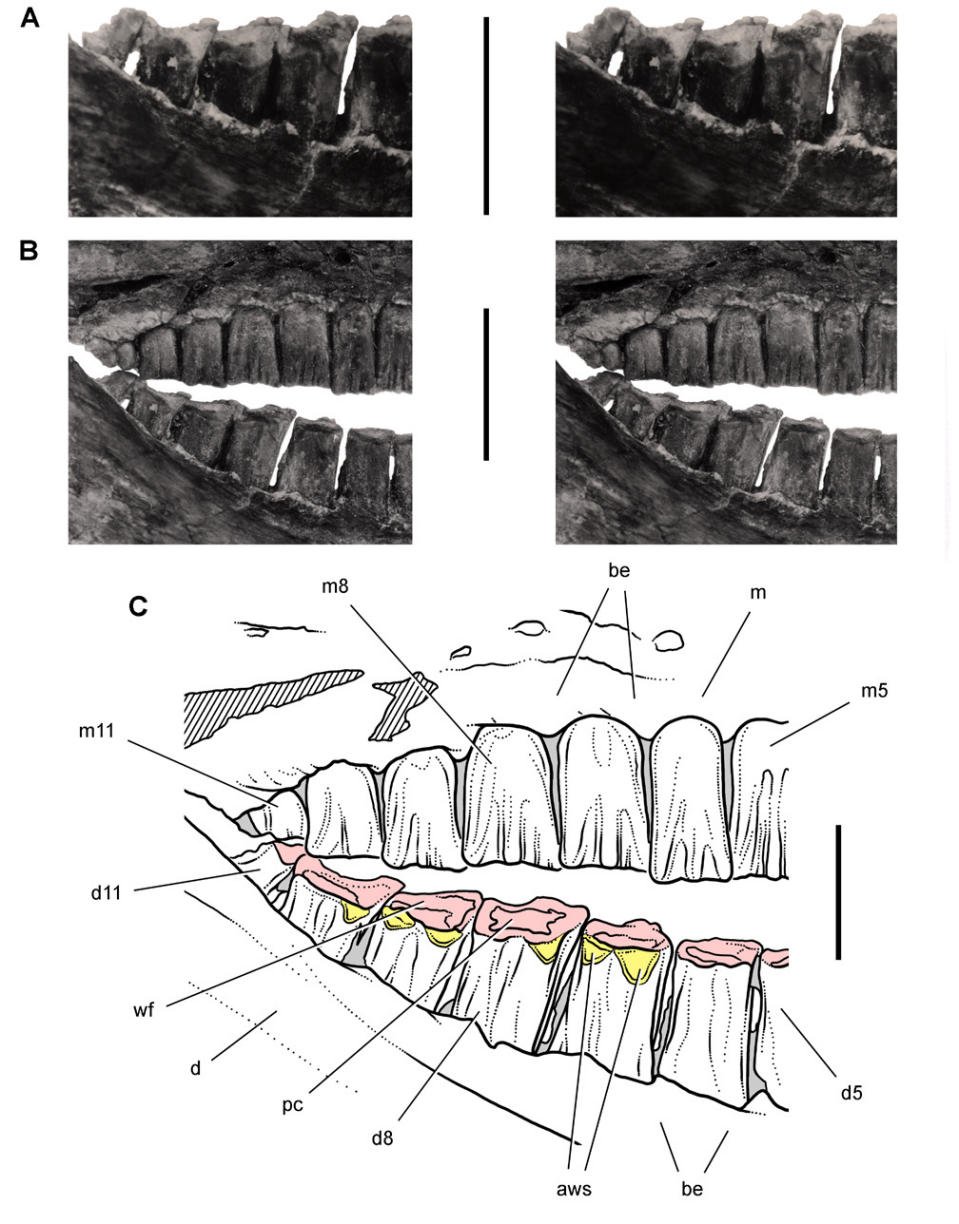
Posterior dentition of *Heterodontosaurus tucki* from the Lower Jurassic Upper Elliot and Clarens formations of South Africa. Posterior half of worn maxillary and dentary tooth row in an adult skull in right lateral view (SAM-PK-K1332). Stereopair (**A**) of right posterior dentary tooth row tipped labially (laterally) exposing the wear facets in dorsolateral view. Stereopair (**B**) and line drawing (**C**) of the posterior half of the tooth rows in natural articulation in lateral view. Hatching indicates broken bone; grey tone indicates matrix; pink tone indicates wear facets; yellow tone indicates accessory wear surfaces. Scale bars equal 1 cm in A and B, 5 mm in C. Abbreviations: ***aws*** accessory wear surface ***be*** buccal emargination ***d*** dentary ***d5***, ***8***, ***11*** dentary tooth 5, 8, 11 ***m*** maxilla ***m5***, ***8***, ***11*** maxillary tooth 5, 8, 11 ***pc*** pulp cavity ***wf*** wear facet.

The cheek teeth in *Heterodontosaurus* are unusual in two other regards—their internal structure and the form of their roots. The pulp cavity of cheek teeth is spacious and extends up into the crown as is well exposed by erosion (Fig. 42) and seen in cross section (Figs 45C, 50C). In the dentition of an adult skull, the pulp cavity is exposed on wear surfaces (Fig. 55B, C). The common ornithischian condition, in contrast, is that the pulp cavity closes during maturation of the tooth, such that the pulp cavity is limited to the root (Figs 53D, 54D). The second unusual feature of the cheek teeth is the bluntly rounded distal end to the roots (Figs 42, 44B). The roots of most ornithischian teeth are more tapered toward their tips, which may retain a small distal opening (Fig. 53B, D). The functional significance of blunt-rooted cheek teeth is not known.

**Figure 56. F56:**
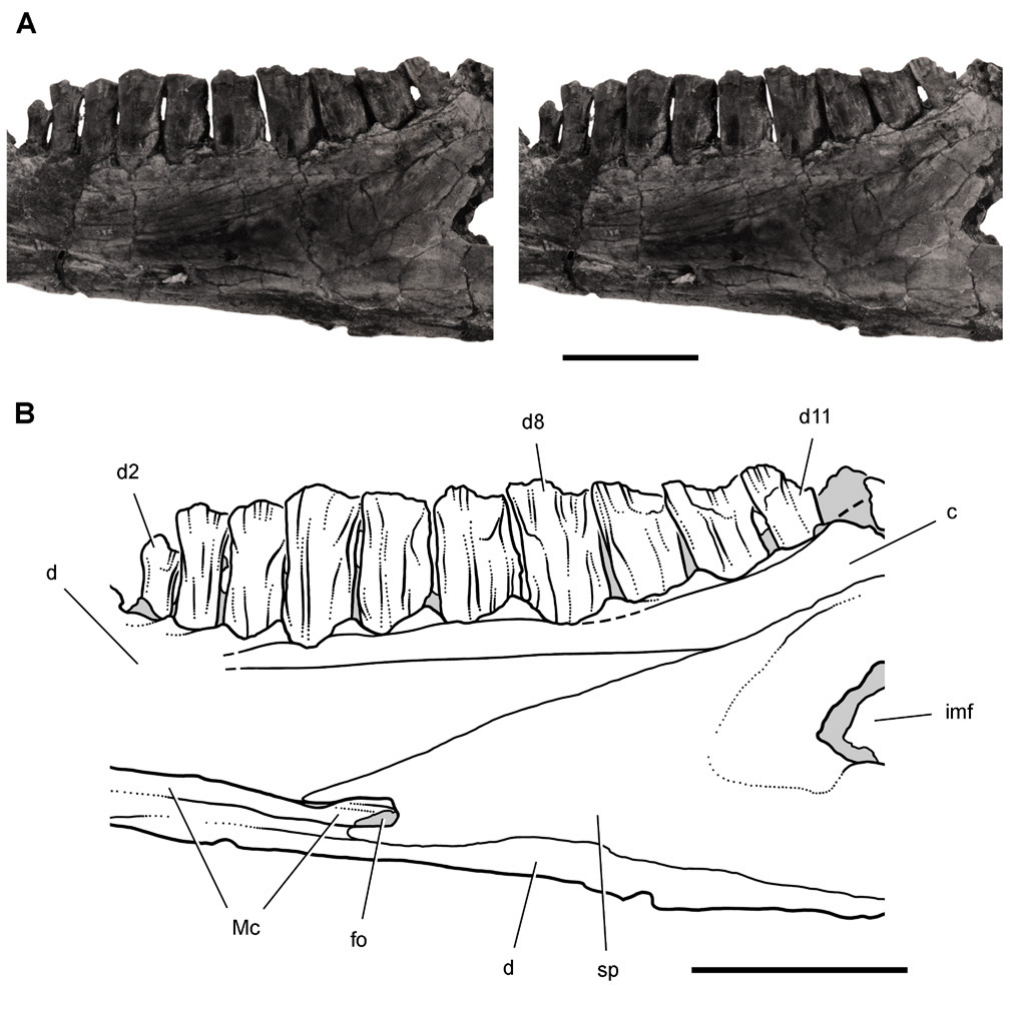
Postcaniniform dentary tooth row of *Heterodontosaurus tucki* from the Lower Jurassic Upper Elliot and Clarens formations of South Africa. Worn right dentary tooth row in an adult skull (SAM-PK-K1332). Stereopair (**A**) and line drawing (**B**) in medial view. Dashed lines indicate estimated edges; tone indicates matrix. Scale bars equal 1 cm in A and B. Abbreviations: ***c*** coronoid ***d*** dentary ***d2***, ***8***, ***11*** dentary tooth 2, 8, 11 ***fo*** foramen ***imf*** internal mandibular fenestra; ***Mc*** Meckel’s canal ***sp*** splenial.

##### Dentary teeth.

**Figure 57. F57:**
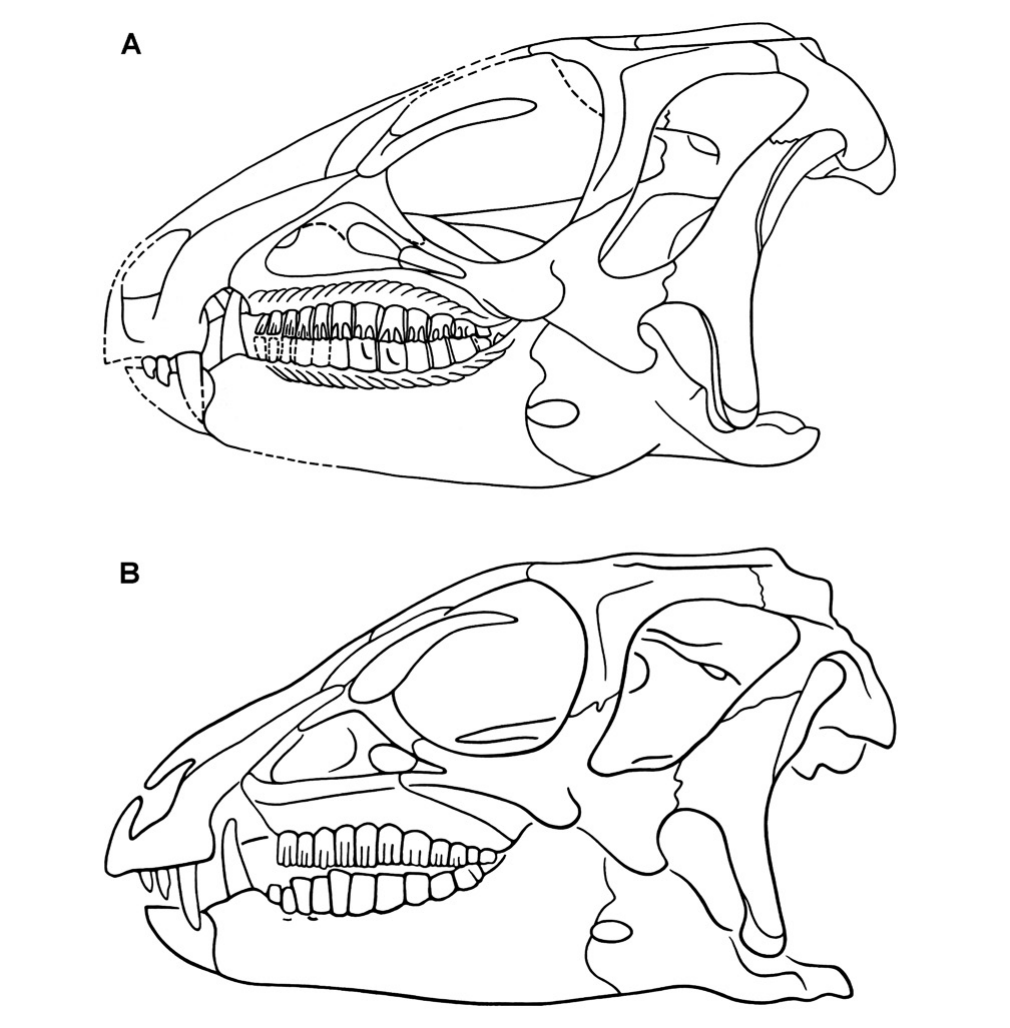
Skull of *Heterodontosaurus tucki* from the Lower Jurassic Upper Elliot and Clarens formations of South Africa. Previous skull reconstructions in left lateral view **A** From Charig and Crompton (1974) **B** Reversed from Weishampel (1984).

Unlike *Echinodon* and *Lycorhinus*, thereis no rudimentary dentary tooth preceding the caniniform tooth in *Heterodontosaurus*. The central axis of the dentary caniniform tooth, the largest tooth in the dentition, is tilted mesially toward the premaxillary caniniform tooth. Its root curves posteroventrally into the body of the dentary as in other heterodontosaurids (Figs 38, 59). When the lower tooth row is held horizontal, the tip of the caniniform crown is positioned over the mesial rather than distal edge of the crown base, as preserved on both sides of SAM-PK-K1332 (Norman et al. 2011: Figs 16, 17). The caniniform tooth is slightly more vertically oriented in the holotypic skull, although still mesially canted (Norman et al. 2011: fig. 1). In some skull reconstructions, the caniniform has been incorrectly shown with a vertical orientation (Fig. 57A). Mesial and distal carinae of the caniniform tooth are serrate. The serrations are limited to the apical one-half of the mesial margin.

There are 10 postcaniniform dentary crowns (d2-11) opposing 11 maxillary crowns. The dentary tooth row, nonetheless, terminates distally even with (SAM-PK-K1332), or distal to (AMNH 24000), the last maxillary tooth, because d1-4 crowns are relatively broader than their counterparts in the maxilla (Figs 41, 55, 59). Mesiodistal crown width increases from d2 to d8 or d9 and then decreases slightly in d10 and more dramatically in d11. Postcaniniform tooth count was initially reported as 12 or 13 (Crompton and Charig 1962; Charig and Crompton 1974), although only 10 postcaniniform alveoli are present in right and left dentaries in SAM-PK-K1332 (Hopson 1980; Norman et al. 2011; Fig. 59).

**Figure 58. F58:**
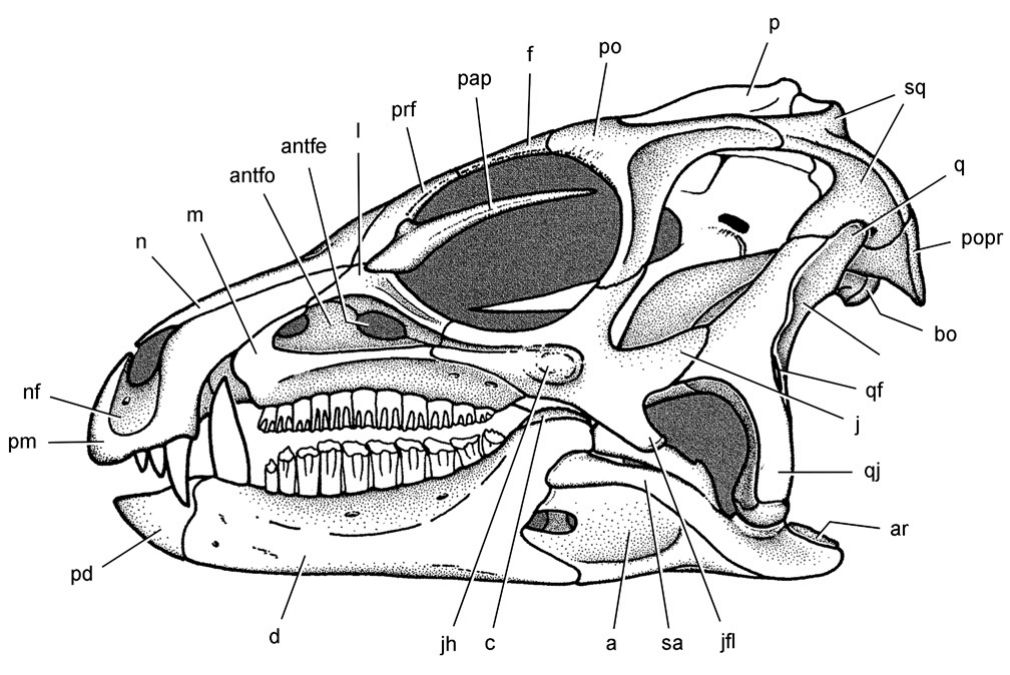
Skull of *Heterodontosaurus tucki* from the Lower Jurassic Upper Elliot and Clarens formations of South Africa. Skull reconstruction in left lateral view from Norman et al. (2011). Abbreviations: ***a*** angular ***antfe*** antorbital fenestra ***antfo*** antorbital fossa ***ar*** articular ***bo*** basioccipital ***c*** coronoid ***d*** dentary ***f*** frontal ***j*** jugal ***jfl*** jugal flange ***jh*** jugal horn ***l*** lacrimal ***m*** maxilla ***n*** nasal ***nf*** narial fossa ***p*** parietal ***pap***  palpebral ***pd*** predentary ***pm*** premaxilla ***po*** postorbital ***popr*** paroccipital process ***prf*** prefrontal ***q*** quadrate ***qf***  quadrate foramen ***qj*** quadratojugal ***sa*** surangular ***sq*** squamosal.

**Figure 59. F59:**
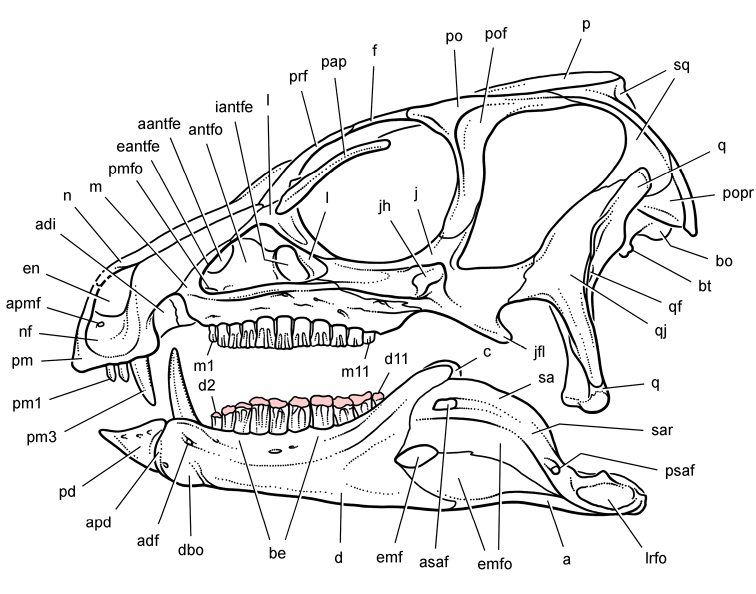
Skull of *Heterodontosaurus tucki* from the Lower Jurassic Upper Elliot and Clarens formations of South Africa. New skull reconstruction in lateral view showing the dentition with intermediate wear (scleral ring not shown). Pink tone indicates wear facets. Abbreviations: ***a*** angular ***aantfe*** accessory antorbital fenestra ***adf*** anterior dentary foramen ***adi*** arched diastema ***antfo*** antorbital fossa ***apd*** articular surface for the predentary ***apmf*** anterior premaxillary foramen ***asaf*** anterior surangular foramen ***be*** buccal emargination ***bo*** basioccipital ***bt*** basal tubera ***c*** coronoid ***d*** dentary ***d2***, ***d11*** dentary tooth 2, 11 ***dbo*** dentary boss ***eantfe*** external antorbital fenestra ***emf*** external mandibular fenestra ***emfo*** external mandibular fossa ***en*** external naris ***f*** frontal ***iantfe*** internal antorbital fenestra ***j*** jugal ***jfl*** jugal flange ***jh*** jugal horn ***l*** lacrimal ***lrfo*** lateral retroarticular fossa ***m*** maxilla ***m1***, ***11*** maxillary tooth 1, 11 ***n*** nasal ***nf*** narial fossa ***p*** parietal ***pap*** palpebral ***pd*** predentary ***pm*** premaxilla ***pm1***, ***3*** premaxillary tooth 1, 3 ***pmfo*** promaxillary fossa ***po*** postorbital ***pof*** postorbital fossa ***popr*** paroccipital process ***prf*** prefrontal ***psaf*** posterior surangular foramen ***q*** quadrate ***qf*** quadrate foramen ***qj*** quadratojugal ***sa*** surangular ***sar*** surangular ridge ***sq*** squamosal.

##### Cervical vertebrae.

The vertebral column includes a well exposed cervical series starting anteriorly with the axis (Figs 62, 63). As in other vertebrae, the axial centrum and neural arch are completely coossified, suggesting that the referred skeleton (SAM-PK-K1332) pertains to a mature individual. Anteriorly, the axial intercentrum is cup-shaped and fused to the axial centrum. The wedge-shaped odontoid, likewise, is fully coossified and is beveled for articulation with the occipital condyle (Fig. 63A). The posterior face of the axial centrum is gently concave. The relatively low-angled neural spine is proportionately long, projecting farther over C3 than in most other basal ornithischians such as *Lesothosaurus* and *Hypsilophodon* (Galton 1974a; Sereno 1991). Unlike other ornithischians, the axial neural spine is relatively narrow, tapering in transverse width from the postzygapophyses to the posterior termination of the spine.

The parapophysis and diapophysis of cervical vertebra 3 and successive cervical vertebrae are more prominently developed than in other basal ornithischians such as *Lesothosaurus* (Sereno 1991). The diapophysis is already developed as a subcylindrical process in C3 and the parapophysis likewise by C4 (Figs 62, 63B). The neural spine of C3 is unusually elongate for an ornithischian (Fig. 62). A hypertrophied epipophysis extends posterodorsally over the proximal one-half of C4. Joined medially for much of its length by the neural spine, this composite process resembles the axial spine in shape and orientation. Unlike the condition in the axis, however, a ridge from the prezygapophysis joins the epipophysis over the postzygapophysis (Figs 62, 63B). The basal ornithischian *Lesothosaurus* also has an epipophysis on C3, but it is developed only as a blunt process that does not extend beyond the postzygapophysis (Sereno 1991).

The postzygapophysis of C4 bears an epipophysis that is joined by a ridge from the prezygapophysis. Although developed as a prominent process, the epipophysis does not extend beyond the postzygapophysis. The neural spine also has a broad base that may be incomplete distally (Figs 62, 63B).

The postzygapophyses of C5 and C6 are joined to the prezygapophyses by a low ridge but no epipophyseal processes are present. A marked change in the shape of the prezygapophysis occurs between C5 and C6, the latter more erect and supporting a broad hook-shaped articular surface (Figs 62, 63B). As noted by Santa Luca, the neural spines of C5 and C6 project anterodorsally between the postzygapophyses of the preceding vertebra, another condition unique among ornithischians. No articulation occurs between these spines and the postzygapophyses of the preceding vertebrae.

The length of the centra varies along the cervical column with C6-C9 noticeably shorter in length, as observed by Santa Luca (Figs 62, 63C). In this connection, the centrum of C3 is also measurably shorter than that of C2, C4 and C5. Thus, there exists the unusual condition in *Heterodontosaurus* in which the centra of C4 and C5 rival the axial centrum in length (Table 7). Variation in centra shape, from a parallelogram to a trapezoid, imparts an S-shaped curve to the cervical series in natural articulation (Fig. 62). The cervical centra are slightly amphicoelous with no development of opisthocoelous articulations as occurs in derived euornithopods with a highly flexed cervical series.

Santa Luca tentatively identified the transition between cervical and dorsal vertebrae, and further information supports his conclusions. The best indicator of the transition from cervical to dorsal vertebrae in ornithischians is the abrupt dorsal shift of the parapophysis from the centrum to the base of the transverse process on the neural arch. The position of the parapophysis on the anterodorsal corner of the centrum in C9 is similar to that in preceding cervical vertebrae (Figs 62, 63C). Most of this portion of D1 is exposed with no sign of a prominent parapophysis. The rib of C9, in addition, has a significantly longer tuberculum than the rib of D1, both of which are near their natural articulation. The proportions of the rib of D1 suggest that the parapophysis was located at the base of the transverse process, rather than below the neurocentral suture. Thus the evidence suggests that there are nine cervical vertebrae.

**Figure 60. F60:**
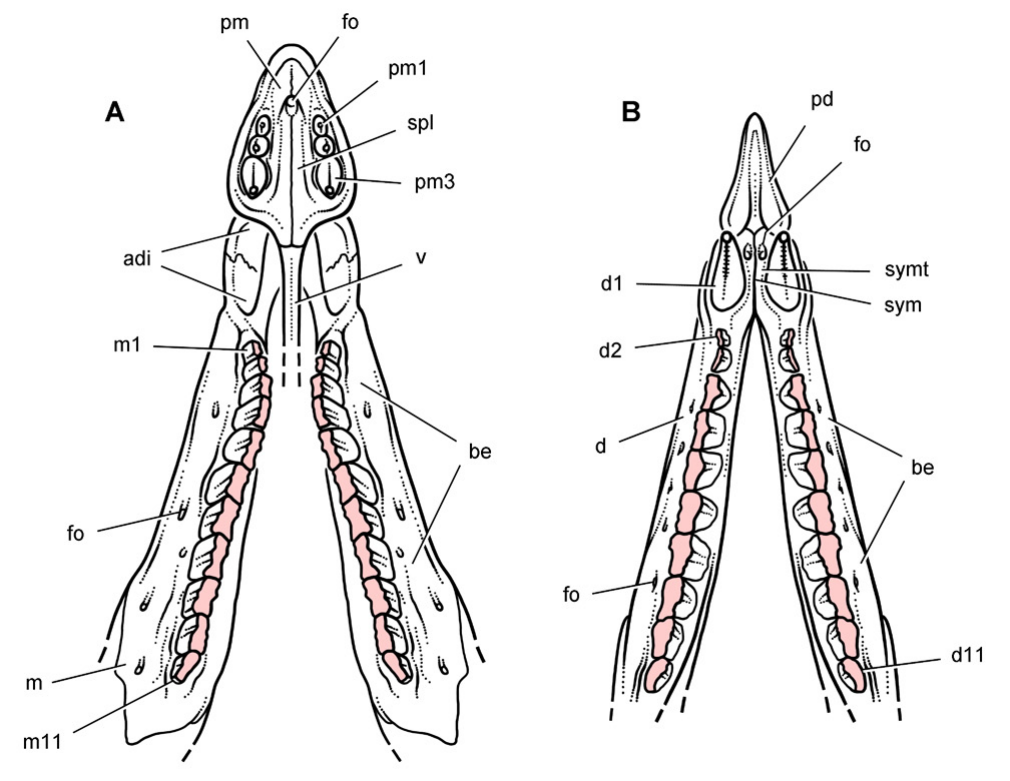
Jaws of *Heterodontosaurus tucki* from the Lower Jurassic Upper Elliot and Clarens formations of South Africa. Reconstruction of upper and lower jaws showing the dentition with heavy wear (based on SAM-PK-K1332). Dashes indicate estimated edges; pink tone indicates wear facets **A** Upper dentition and anterior palate in ventral view **B** Lower dentition, dentary symphysis and predentary in dorsal view. Abbreviations: ***adi*** arched diastema ***be*** buccal emargination ***d*** dentary ***d1***, ***2***, ***11*** dentary tooth 1, 2, 11 ***fo*** foramen ***m*** maxilla ***m1***, ***11*** maxillary tooth 1, 11 ***pd*** predentary ***pm*** premaxilla ***pm1***, ***3*** premaxillary tooth 1, 3 ***spl*** secondary palate ***sym*** symphysis ***symt*** symphyseal trough ***v*** vomer.

**Figure 61. F61:**
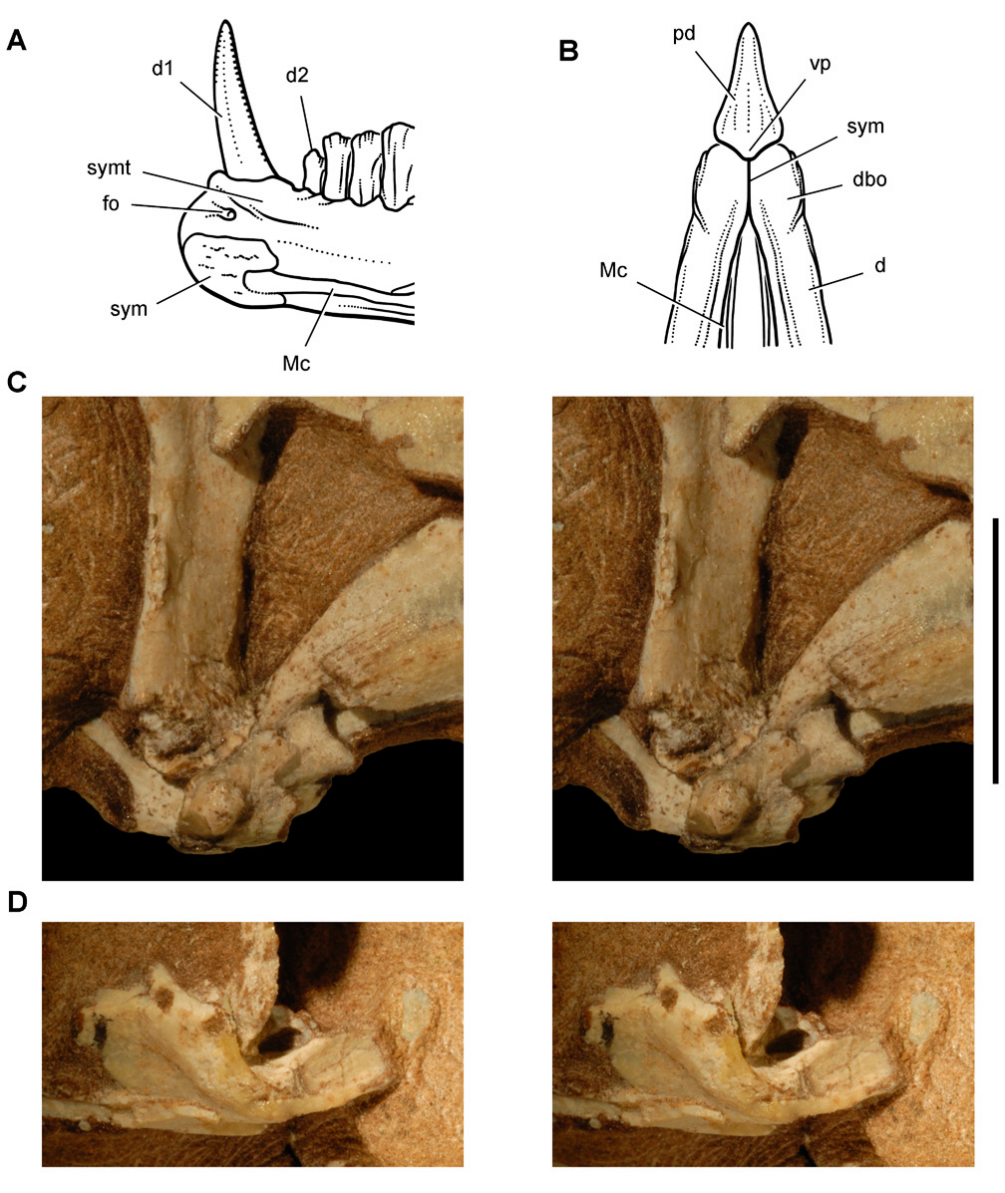
Jaw joints of *Heterodontosaurus tucki* from the Lower Jurassic Upper Elliot and Clarens formations of South Africa. Dentary symphysis and quadrate-articular jaw joint **A** Reconstruction of the anterior portion of the right dentary in medial view (based on SAM-PK-K1332) **B** Reconstruction of the anterior end of the lower jaws in ventral view (based on SAM-PK-K1332) **C** Stereopair of the right lower jaw joint in lateral view (the lateral edge of the quadrate condyle and articular are broken away exposing the tight jaw articulation) in a subadult skull (AMNH 24000) **D** Stereopair of the left lower jaw joint in lateral view (the lateral edge of the quadrate condyle is broken away exposing the inclined trough of the jaw articulation) in a subadult skull (AMNH 24000). Scale bar equals 1 cm in **C** and **D**. Abbreviations: ***d***  dentary ***d1***, ***2*** dentary tooth 1, 2 ***dbo*** dentary boss ***fo*** foramen ***Mc*** Meckel’s canal ***pd*** predentary ***sym*** symphysis ***symt*** symphyseal trough ***vp*** ventral process.

**Figure 62. F62:**
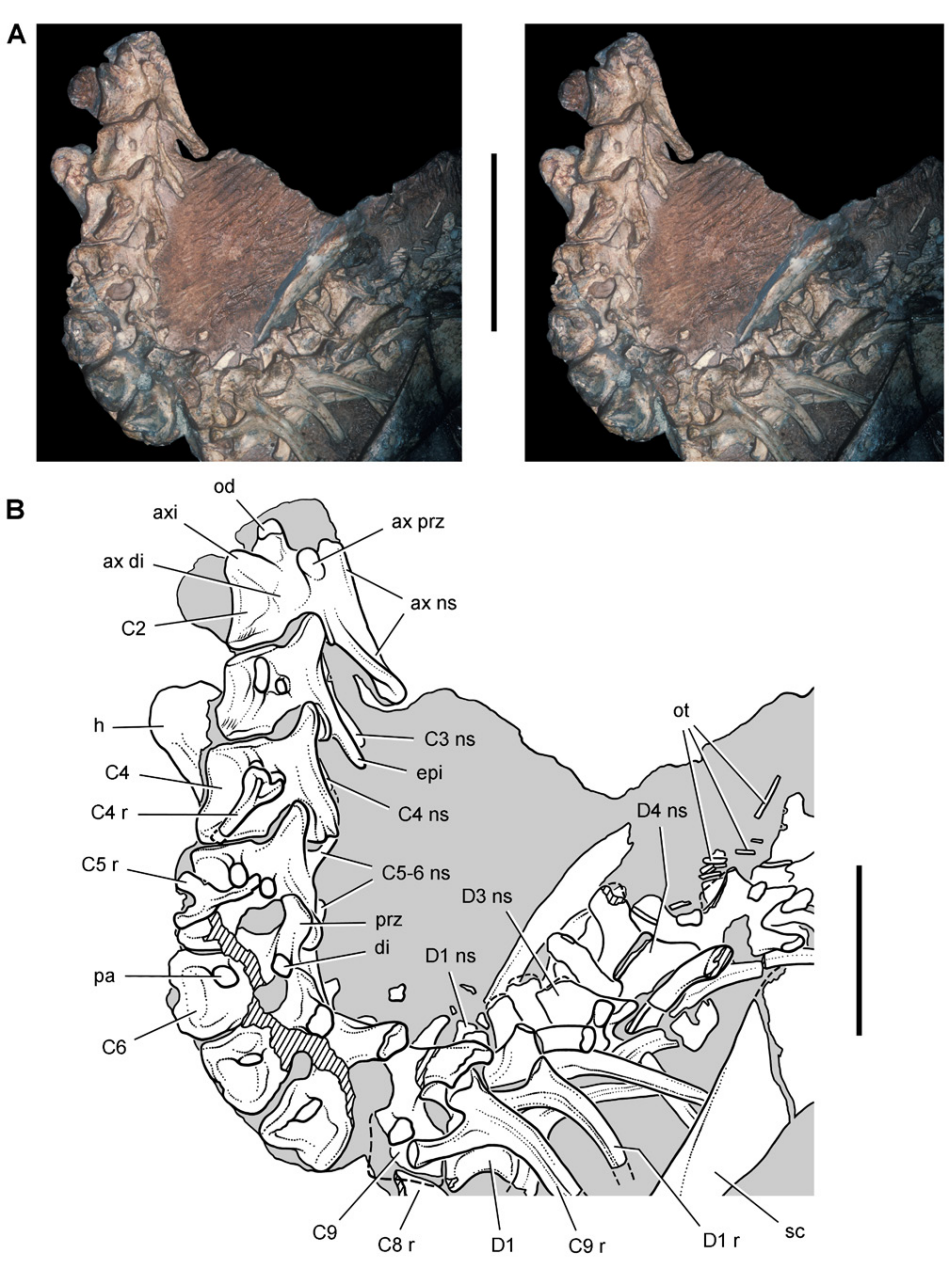
Presacral vertebrae of *Heterodontosaurus tucki* from the Lower Jurassic Upper Elliot and Clarens formations of South Africa. Cervical and anterior dorsal vertebrae and ribs of an adult skeleton (SAM-PK-K1332). Stereopair (**A**) and line drawing (**B**) in left lateral view. Hatching indicates broken bone; dashed lines indicate estimated edges; tone indicates matrix. Scale bars equal 5 cm in **A** and 3 cm in **B**. Abbreviations: ***ax*** axial ***axi*** axial intercentrum ***C2-6***, ***8***, ***9*** cervical vertebra 2–6, 8, 9 ***D1***, ***3***, ***4*** dorsal vertebra 1, 3, 4 ***di*** diapophysis ***epi*** epipophysis ***h*** humerus ***ns*** neural spine ***od*** odontoid ***ot*** ossified tendons ***pa*** parapophysis ***prz*** prezygapohpysis ***r*** rib ***sc*** scapula.

**Figure 63. F63:**
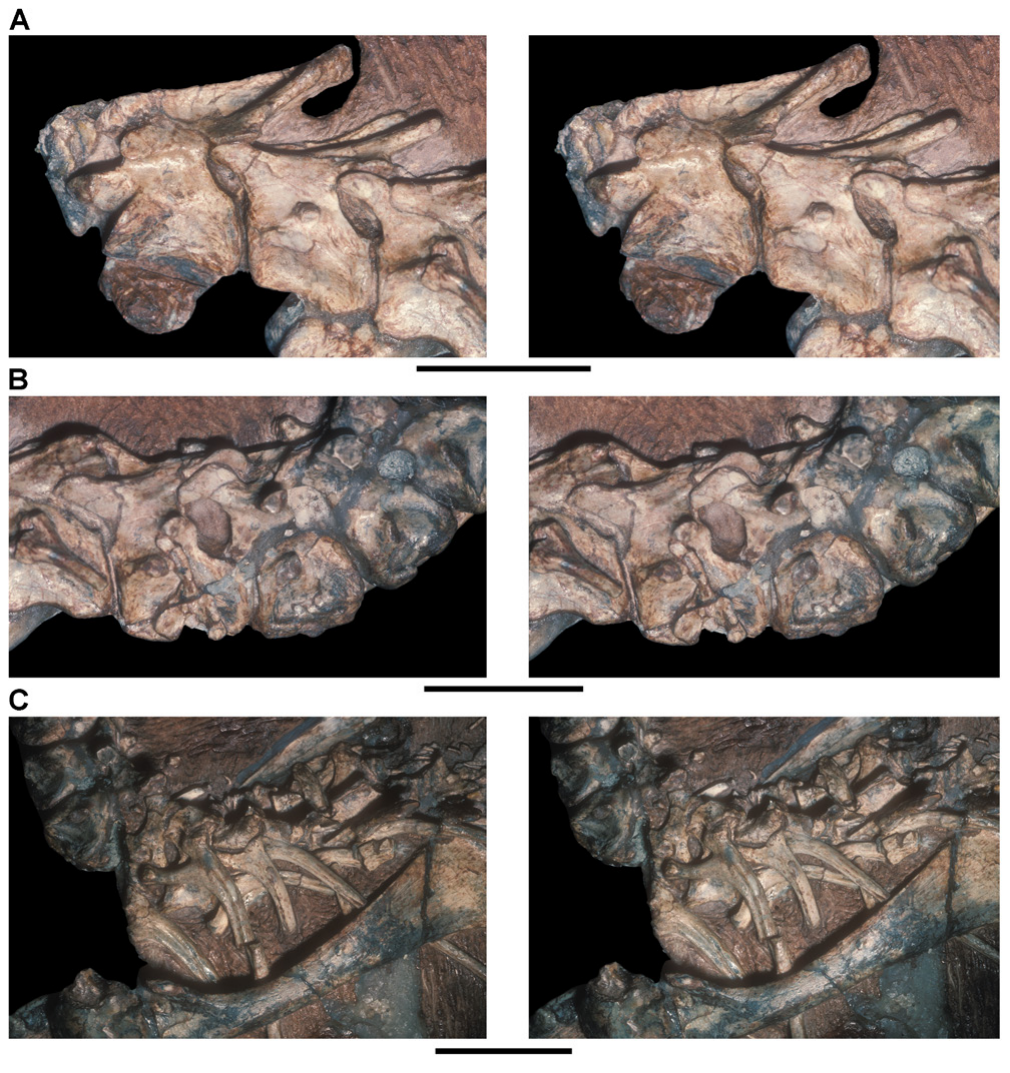
Presacral vertebrae of *Heterodontosaurus tucki* from the Lower Jurassic Upper Elliot and Clarens formations of South Africa. Cervical and anterior dorsal vertebrae and ribs of an adult skeleton (SAM-PK-K1332). Stereopairs of C1-3 (**A**), C4-6 (**B**), and C7-D4 (**C**) in left lateral view. Scale bars equal 2 cm in **A** and **B**, 3 cm in **C**.

##### Sacral vertebrae.

Santa Luca (1980) identified 12 dorsal and 6 sacral vertebrae, describing S1 and S2 with coossified centra and ribs that articulate with the preacetabular process of the ilium. These centra are clearly coossified, as is exposed ventral to the left preacetabular process (Fig. 68). The vertebra he identified as S2, however, is also clearly homologous with S1 in basal dinosaurs such as *Lesothosaurus*, which have only five sacral vertebrae (Sereno 1991; Butler 2005). In *Heterodontosaurus* the rib of this vertebra is exposed in dorsal view as a flat strut articulating at mid-length along the preacetabular process (Fig. 68). S3, according to Santa Luca (1980), has a stout sacral rib articulating with the pubic peduncle, the typical attachment for S2. The vertebrae he identified as S4-S6 have ribs or transverse processes that articulate with the iliac blade. The vertebra he identified as S6 contacts the brevis shelf at mid-length along the postacetabular process, as is typical of S5 in many other basal ornithischians (Fig. 68).

Dorsal and sacral counts hinge on the interpretation of the transverse process and rib in presacral vertebra 21 (girdle contact, not central fusion, is the arbiter regarding status as a sacral vertebra). Although the transverse process is broken at its tip, a portion of its rib is preserved projecting under the preacetabular process of the ilium (Fig. 68). An articulation with the pelvic girdle, if it existed, cannot be observed. The pelvis has undergone some transverse crushing, during which the left ilium has shifted posteroventrally. The sacral attachments that extend laterally on the right side, for example, angle posterolaterally on the left side. When restored to its original more elevated position, the left preacetabular process may well have arched over the rib of presacral vertebra 21, which is here interpreted as D13 (Fig. 68).

In most ornithischians, the end of the preacetabular process is free of sacral rib articulations. In the euornithopod *Thescelosaurus neglectus* (Galton 1974b), however, a dorsal rib contacts with the distal end of the preacetabular process, although neither the intercentral suture nor rib-ilium articulation are coossified. Given the position and apparent brevity of the rib of presacral vertebra 21 in *Heterodontosaurus*, this vertebra is tentatively regarded as the last dorsal (D13). This is a low number, as most basal ornithischians have 14-16 dorsal vertebrae. The position of the rib on the right side of presacral vertebra 21 could be determined by computed tomographic imaging.

##### Sternum, ribs and ossified tendons.

Santa Luca (1980: 173, fig. 23) mentioned the possible presence of a sternal plate, but he did not figure this bone and omitted it from his skeletal reconstruction. Subsequent skeletal reconstructions show ossified sternal plates (Bakker 1986: 453, 455) or possibly ossified plates and sternal ribs (Brett-Surman 1997: fig. 24.1B).

**Figure 64. F64:**
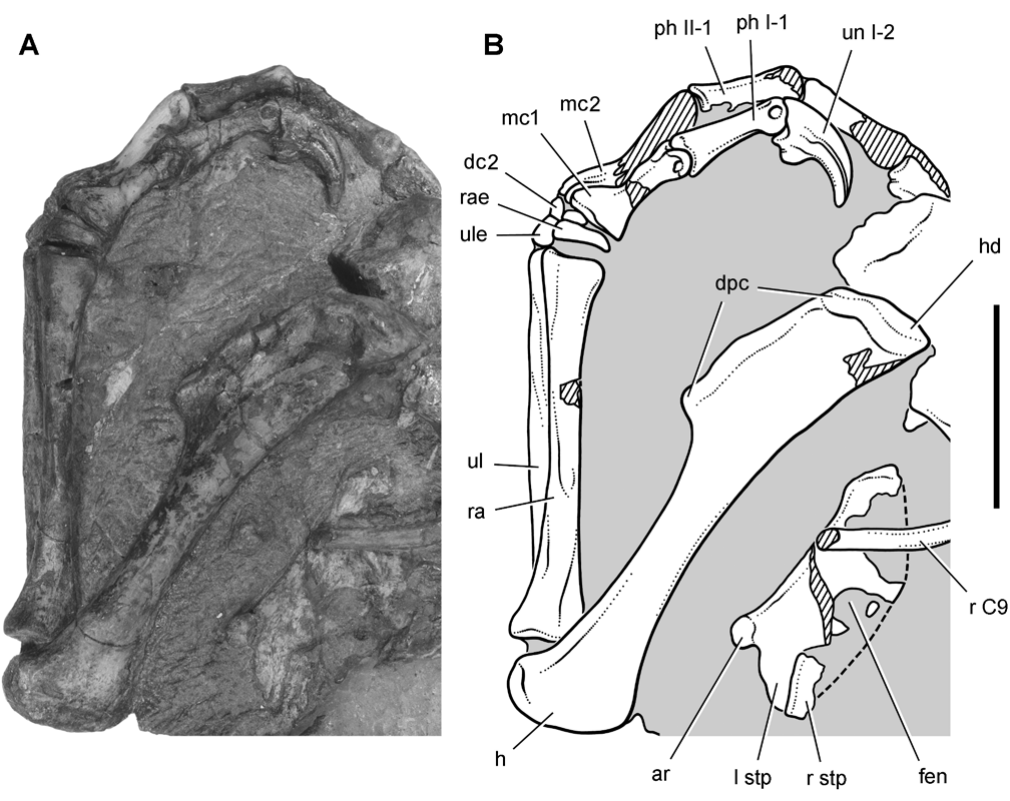
Pectoral girdle and forelimb of *Heterodontosaurus tucki* from the Lower Jurassic Upper Elliot and Clarens formations of South Africa. Pectoral girdle and forelimb of an adult skeleton (SAM-PK-K1332). Photograph (**A**) and line drawing (**B**) of the sternal plates and left forelimb in dorsal and medial view, respectively. Hatching indicates broken bone; dashed lines indicate estimated edges; tone indicates matrix. Scale bar equals 3 cm. Abbreviations: ***I***, ***II*** digit I, II ***ar*** articular surface for a sternal rib ***C9*** cervical 9 ***dc2*** distal carpal 2 ***dpc*** deltopectoral crest ***fen*** fenestra ***h*** humerus ***hd*** head ***l*** left ***mc1***, ***2*** metacarpal 1, 2 ***ph*** phalanx ***r*** rib or right ***ra*** radius ***rae*** radiale ***stp*** sternal plate ***ul*** ulna ***ule*** ulnare ***un*** ungual.

A pair of crescentic ossified sternal plates is present in SAM-PK-K1332, resembling in shape those in basal neornithischians such as *Hypsilophodon* (Galton 1974a) or *Psittacosaurus* (Sereno et al. 2009). Most of the left sternal plate is exposed in dorsal view posterior to the left forelimb (Fig. 64). Its lateral margin is rounded and gently concave, and its proximal end is narrow and thickened. A rounded process and notch are present along its distal edge, presumably for cartilaginous rib attachment. The medial margin, some of which is obscured by matrix, appears to be convex and longer than the lateral margin. The arcuate margin of an unossified fenestra appears to be present in the center of the left sternal plate (Fig. 64). Unlike *Hypsilophodon*, there are no ossified sternal ribs.

Although the axial rib is not preserved, a prominence straddling the neurocentral suture probably represents the diapophysis for a small two-headed axial rib (Fig. 62). The rib of C4 is preserved near its natural articulation and closely resembles that in *Hypsilophodon* (Galton (1974a). The capitulum is shorter and stouter than the tuberculum and more closely associated with a low spinous process. The rib shaft would have extended posteriorly just beyond the posterior edge of the centrum (Fig. 62). The rib of C5 is similar, the proximal processes Y-shaped and more divergent. Most of the ribs of C8 and 9 are exposed near the left scapulocoracoid. These are robust, long ribs with a longer capitulum and tuberculum then in *Hypsilophodon* (Galton 1974a).

Ossified tendons are present from the anterior dorsal through the sacral vertebrae (Figs 62, 68), a distribution that may be primitive for ornithischians. The first trace of ossified tendons occurs just posterior to the neural spine of D4, and the last trace is present on S4. All are preserved as slender, non-bifurcating, floating epaxial rods that are located between the transverse process and neural spine.

##### Forelimb.

**Figure 65. F65:**
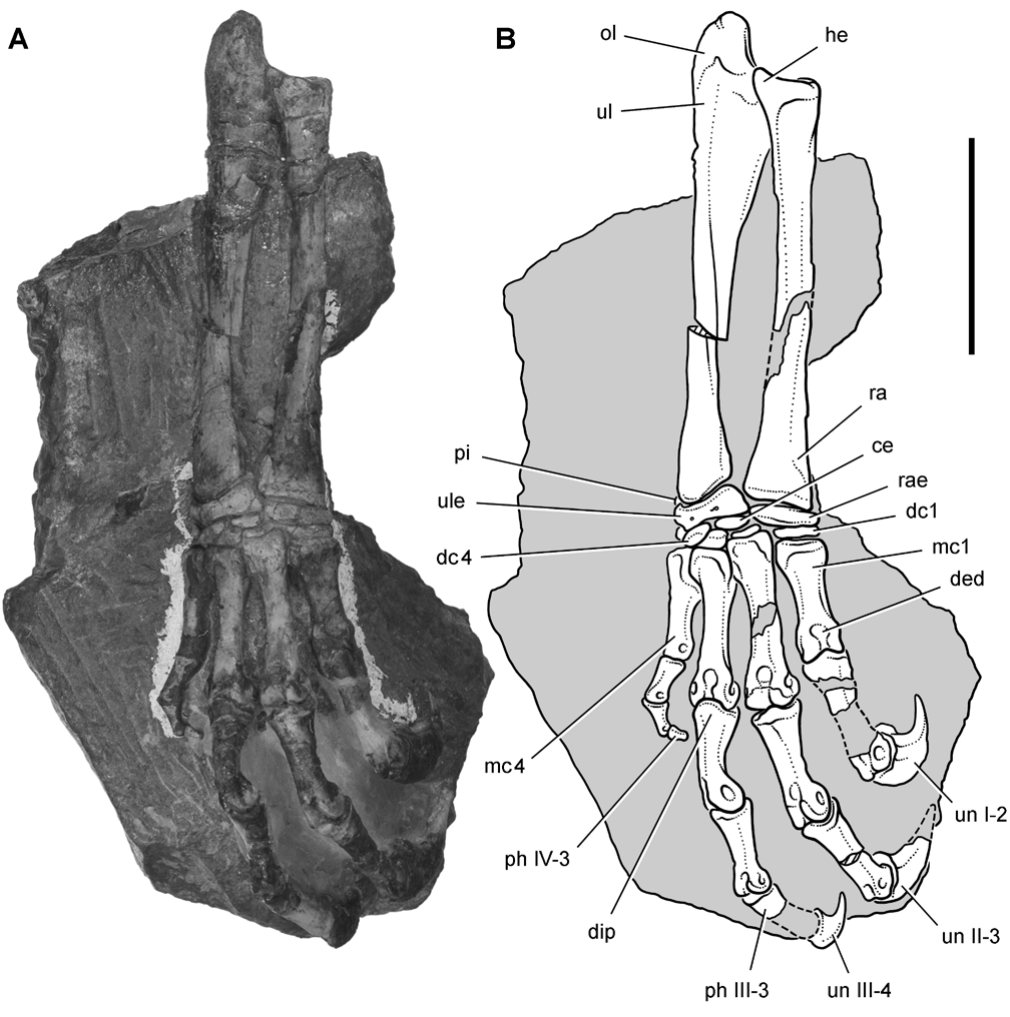
Forelimb of *Heterodontosaurus tucki* from the Lower Jurassic Upper Elliot and Clarens formations of South Africa. Antebrachium, carpus and manus of an adult skeleton (SAM-PK-K1332). Photograph (**A**) and line drawing (**B**) of the right radius, ulna, carpus and manus in dorsal view. Hatching indicates broken bone; dashed lines indicate estimated edges; tone indicates matrix. Scale bar equals 3 cm. Abbreviations: ***I-IV*** digits I-IV ***ce*** centrale ***dc1***, ***4*** distal carpal 1, 4 ***ded*** dorsal extensor depression ***dip*** dorsal intercondylar process ***he*** heel ***mc1***, ***4*** metacarpal 1, 4 ***ol*** olecranon ***ph***, phalanx ***pi*** pisiform ***ra*** radius ***rae*** radiale ***ul*** ulna ***ule*** ulnare ***un*** ungual.

The reconstruction of the forearm presented by Santa Luca (1980: Figs 13, 15) did not remove postmortem fracturing and movement that is visible in the right forearm. A fracture crosses the mid-shafts of the right radius and ulna (Fig. 65). The portion of these bones proximal to the fracture has rotated about 30°, so that the olecranon process is exposed in profile and the flattened end of the ulna lies parallel to a plane through the carpus and metacarpus (Fig. 65). The left ulna, in contrast, shows an uncrushed condition, in which a plane through the coronoid and olecranon processes of the ulna is canted at about 45° to the plane of the carpus and metacarpus. In the reconstruction presented here, the proximal portions of the radius and ulna are restored to their natural orientation (Fig. 67A).

Santa Luca reconstructed the metacarpus with the base of metacarpal 2 inset into the carpus relative to metacarpal 1, such that its base articulates medially with distal carpal 1 (Santa Luca 1980: fig. 13; Langer and Benton 2006: fig. 8A). This unusual condition, which is unknown elsewhere in dinosaurs, should not be confused with the basal sauropodomorph condition, in which metacarpal 1, rather than metacarpal 2, is inset into the carpus and articulates laterally with distal carpal 2 (Sereno 2007). Santa Luca’s interpretation was based on the right carpus and manus, which shows this configuration (Fig. 65). In the left forelimb, however, the bases of metacarpals 1 and 2 are aligned (Fig. 64), their squared bases in mutual and matching contact. The apparent inset of metacarpal 2 in *Heterodontosaurus*, thus, appears to be an artifact of preservation in the right forelimb. The bases of metacarpals 1 and 2 were probably in alignment (Fig. 67C, as also appears to be the case in the largely articulated manus of *Abrictosaurus* (Fig. 36). The base of metacarpal 3, in contrast, may have been positioned slightly distal to metacarpal 2, as this is preserved on both right and left sides in *Heterodontosaurus* (Fig. 65) and in *Abrictosaurus* (Fig. 36).

**Figure 66. F66:**
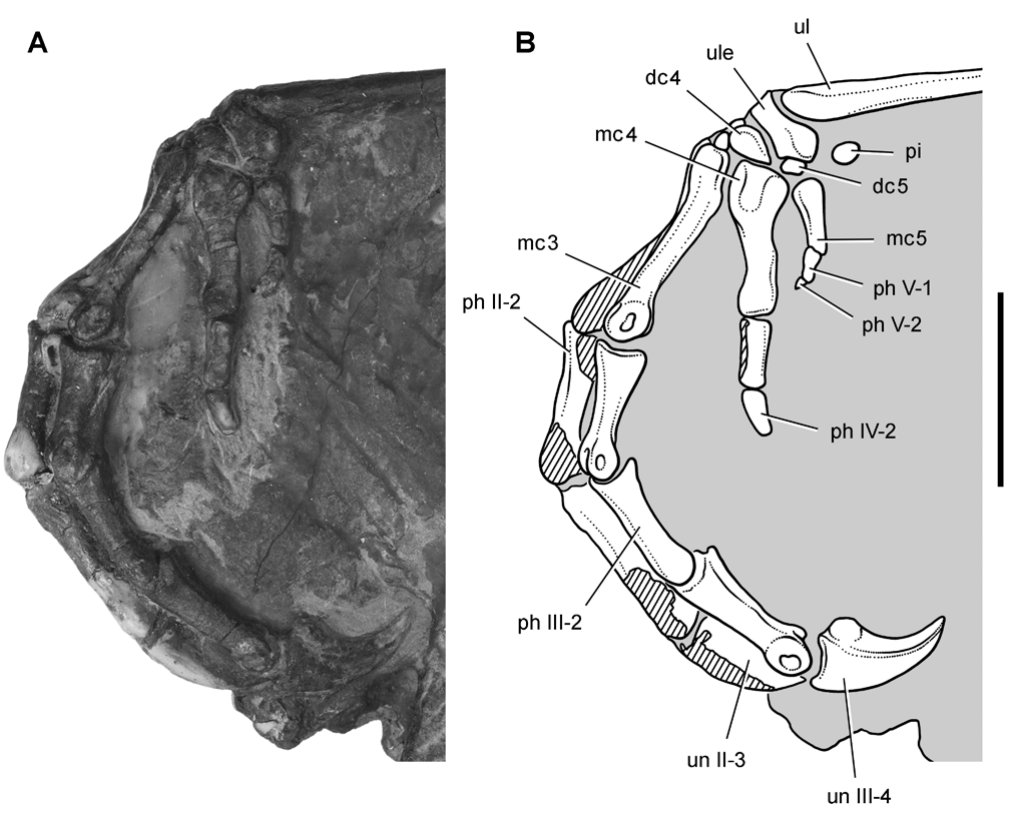
Carpus and manus of *Heterodontosaurus tucki* from the Lower Jurassic Upper Elliot and Clarens formations of South Africa. Carpus and manus of an adult skeleton (SAM-PK-K1332). Photograph (**A**) and line drawing (**B**) of the left carpus and manus in lateral view. Hatching indicates broken bone; tone indicates matrix. Scale bar equals 2 cm. Abbreviations: ***II-V*** digits II-V ***dc4***, ***5*** distal carpal 4, 5 ***mc3-5*** metacarpals 3–5 ***ph*** phalanx ***pi*** pisiform ***ul*** ulna ***ule*** ulnare ***un*** ungual.

**Figure 67. F67:**
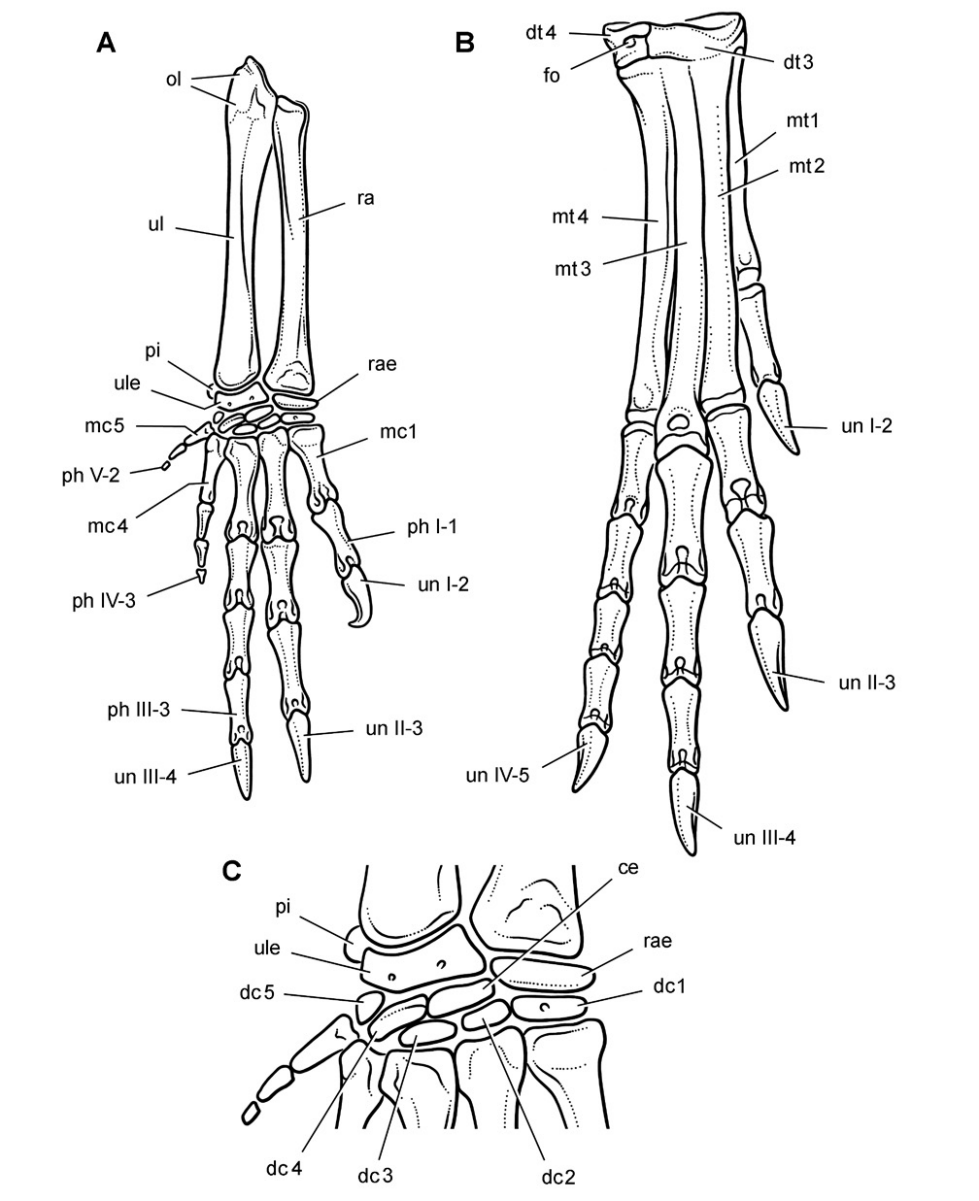
Limbs of *Heterodontosaurus tucki* from the Lower Jurassic Upper Elliot and Clarens formations of South Africa. Reconstructions based on an adult skeleton (SAM-PK-K1332). Right forearm, carpus and manus (**A**), right distal tarsals and pes (**B**), and right carpus (**C**) in dorsal view. Abbreviations: ***I-V*** digits I-V ***ce*** centrale ***dc1-5*** distal carpals 1–5 ***dt3***, ***4*** distal tarsal 3, 4 ***fo*** foramen ***mc1***, ***4***, ***5*** metacarpal 1, 4, 5 ***mt1-4*** metatarsals 1–4 ***ol*** olecranon ***ph*** phalanx ***pi*** pisiform ***ra*** radius ***rae*** radiale ***ul*** ulna ***ule*** ulnare ***un*** ungual.

Metacarpals 1-4 have squared bases that abut one another (Figs 65, 66, 67C), a striking similarity to the condition seen in primitive theropods such as *Herrerasaurus* (Sereno 1993) and *Eodromaeus* (Martinez et al. 2011). *Abrictosaurus* (Fig. 36) and the Kayenta heterodontosaurid appear to show a similar metacarpal condition, which may characterize heterodontosaurids in general.

Bakker and Galton (1974: fig. 1H) figured the manus of *Heterodontosaurus* with strong rotation in digit I. They showed the ungual of digit I directed medially in an extended manus, the medial aspect of the ungual fully exposed in dorsal view of the manus. These authors and Bakker (1986: 453) claimed that a medially twisted thumb characterizes both *Heterodontosaurus* and basal sauropodomorphs such as *Ammosaurus* (= *Anchisaurus*), which has strong medial rotation in the first phalanx of digit I (Sereno 2007).

The right manus as preserved in *Heterodontosaurus*, however, shows only very slight deflection of the distal end of digit I toward the medial side (Fig. 65). This is the original preserved orientation of digit I of the right manus. Despite some damage and loss of the mid section of the first phalanx, metacarpal 1, the base of phalanx I-1 and a portion of the ungual are preserved *in situ* embedded in matrix (Fig. 65). The left manus, in contrast, is transversely compressed with the digits lying subparallel to one another (Figs 64, 66). Although less informative with regard to digital orientation, left phalanx I-1 is compete and preserves a nearly straight shaft (Fig. 64). That matches the preserved position of the proximal and distal ends of right phalanx I-1 (Fig. 65). Thus there is no evidence for medial rotation of the pollex in *Heterodontosaurus* akin to that present in *Eoraptor* (Martinez et al. 2011) and other basal sauropodomorphs (Sereno 2007).

The distal end of metacarpal 1, which is best preserved on the right side, shows asymmetric distal condyles very similar to the condition present in many theropods. The lateral condyle is larger and raised above the medial condyle (Figs 64, 67A), which as noted by Santa Luca would cause the pollex to converge toward the palm during flexion, opposite the direction shown by Galton and Bakker (1974). It is common in the pollex of theropods such as *Eodromaeus* and *Herrerasaurus* (Sereno 1993; Martinez et al. 2011) that the first phalanx shows a slight twist in the opposite direction. A subtle opposite twist is apparent in the first phalanx on both right and left sides in *Heterodontosaurus*, when comparing vertical planes through the proximal and distal ends (Fig. 64). As preserved, all of the principal inner digits in the partially flexed right manus are canted slightly medially toward their distal ends. This is probably the result of subtle postmortem compression of the manus. Given the typical form of the distal condyles of metacarpal 1, the pollex is reconstructed in neutral pose in a manner that would result in its palmar deflection upon flexion (Fig. 67A).

**Figure 68. F68:**
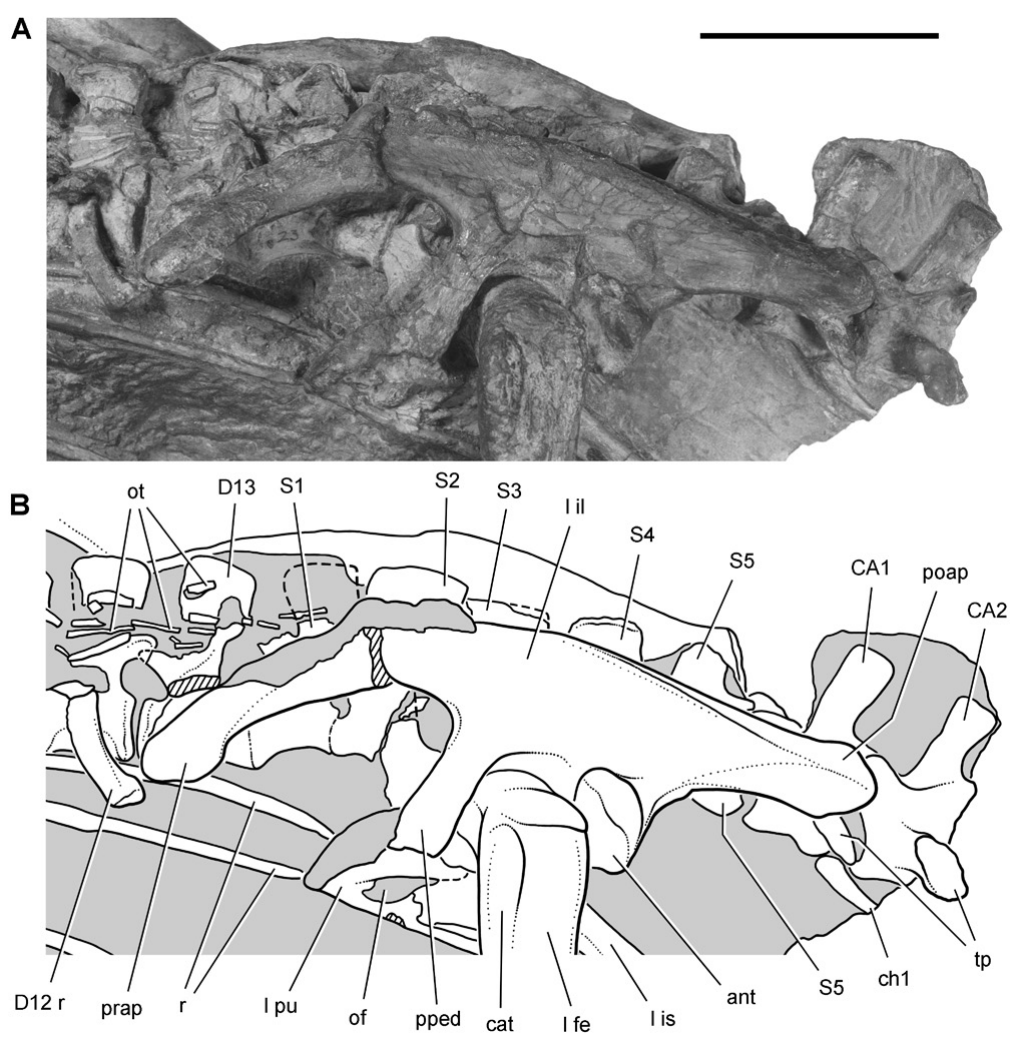
Hip and posterior trunk of *Heterodontosaurus tucki* from the Lower Jurassic Upper Elliot and Clarens formations of South Africa. Hip and posterior trunk in an adult skeleton (SAM-PK-K1332). Photograph (**A**) and line drawing (**B**) of the left ilium and pubis and sacral vertebrae in left lateral view. Hatching indicates broken bone; dashed lines indicate estimated edges; tone indicates matrix. Scale bar equals 3 cm. Abbreviations: ***ant*** antitrochanter ***CA*** caudal vertebra ***cat*** coossified anterior trochanter ***ch*** chevron ***D*** dorsal vertebra ***fe*** femur ***il*** ilium ***is*** ischium ***l*** left ***of*** obturator foramen ***ot*** ossified tendon ***poap*** postacetabular process ***pped*** pubic peduncle ***prap*** preacetabular process ***pu*** pubis ***r*** rib ***S*** sacral vertebra ***tp*** transverse process.

**Figure 69. F69:**
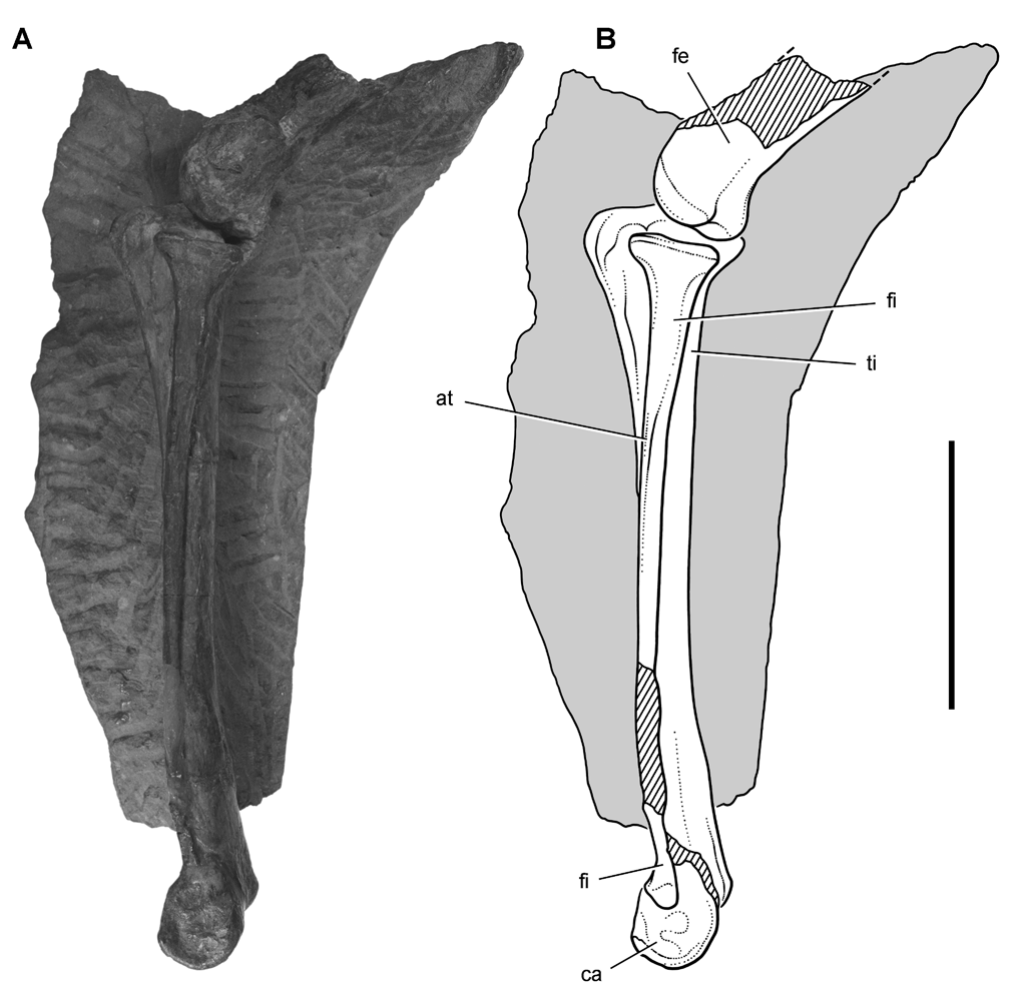
Hindlimb of *Heterodontosaurus tucki* from from the Lower Jurassic Upper Elliot and Clarens formations of South Africa. Tibiotarsus in an adult skeleton (SAM-PK-K1332). Photograph (**A**) and line drawing (**B**) of the left distal femur and tibiotarsus in lateral view. Hatching indicates broken bone; dashed lines indicate estimated edges; tone indicates matrix. Scale bar equals 5 cm. Abbreviations: ***at*** anterior trochanter ***ca*** calcaneum ***fe*** femur ***fi*** fibula ***ti*** tibia.

##### Hindlimb.

**Figure 70. F70:**
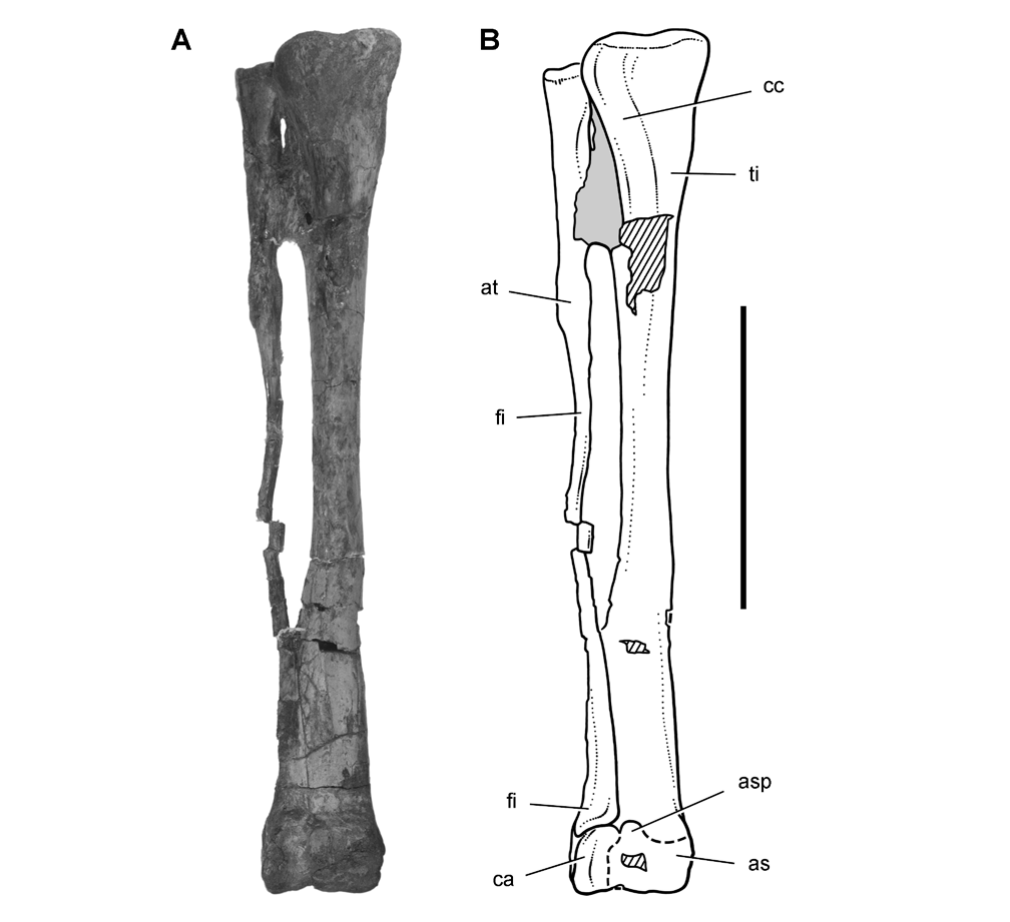
Hindlimb of *Heterodontosaurus tucki* from the Lower Jurassic Upper Elliot and Clarens formations of South Africa. Tibiotarsus in an adult skeleton (SAM-PK-K1328). Photograph (**A**) and line drawing (**B**) of the right tibiotarsus in anterior view. Hatching indicates broken bone; dashed lines indicate estimated edges; tone indicates matrix. Scale bar equals 5 cm. Abbreviations: ***as*** astragalus ***asp*** ascending process ***at*** anterior trochanter ***ca*** calcaneum ***cc*** cnemial crest ***fi*** fibula ***ti*** tibia.

In SAM-PK-K1332 the tibia, fibula, astragalus and calcaneum are coossified as a tibiotarsus as noted by Santa Luca. That coossification, however, is not as complete and homogeneous as in avians. The distal suture between the fibula and calcaneum, for example, is visible, and the distal end of the fibula is expanded anteroposteriorly and transversely (Fig. 69). In a second individual of similar size collected from the same formation (SAM-PK-K1328), partial sutures between the fibula, calcaneum and astragalus are visible (Fig. 70). The astragalus has a short ascending process that may establish a contact with the distal end of the fibula, and the calcaneum has a transverse width approximately 25% of the width of the proximal tarsus (Fig. 70).

**Figure 71. F71:**
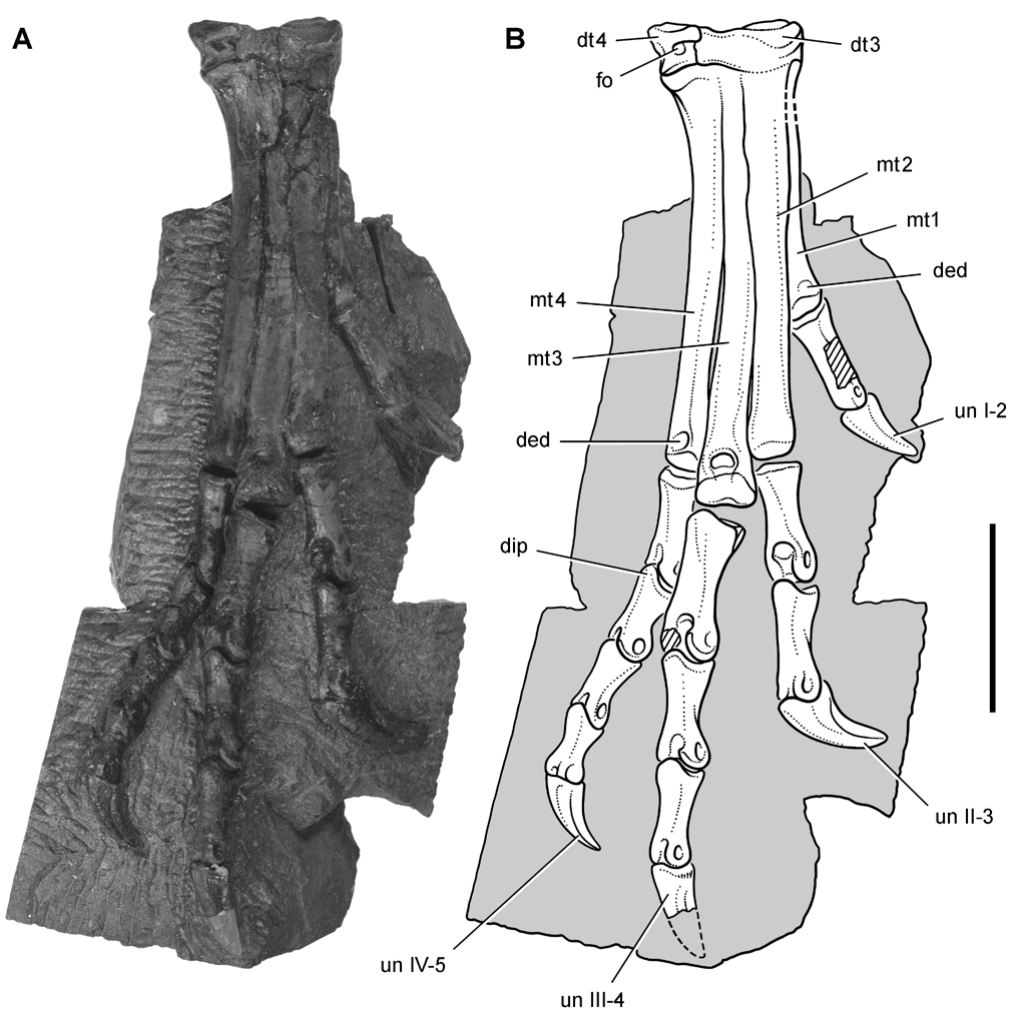
Pes of *Heterodontosaurus tucki* from the Lower Jurassic Upper Elliot and Clarens formations of South Africa. Tarsometatarsus and pes of an adult skeleton (SAM-PK-K1328). Photograph (**A**) and line drawing (**B**) of the right tarsometatarsus and pes in anterior view. Hatching indicates broken bone; dashed lines indicate estimated edges; tone indicates matrix. Scale bar equals 3 cm. Abbreviations: ***I-IV*** digits I-IV ***ded*** dorsal extensor depression ***dip*** dorsal intercondylar process ***dt3***, ***4*** distal tarsals 3, 4 ***fo*** foramen ***mt1-4*** metatarsals 1-4 ***un*** ungual.

Santa Luca described three distal tarsals. This would be an unusual condition for an ornithischian, which usually retain two distal tarsals interpreted as distal tarsals 3 and 4. The usual pair of distal tarsals, however, are present, the flatter broader medial distal tarsal split by a crack in the block containing the right distal tarsus and pes (Figs 67B, 71). The left tarsometatarsus confirms the presence of only two distal tarsals.

Distal tarsal 3 is partially coossified with the metatarsus, the suture between its thinner medial portion and metatarsal 1 obliterated medially and posteriorly. The cuboid distal tarsal 4 is partially coossified with the medial distal tarsal and the proximal end of metatarsal 4. It is slightly wasted with an everted dorsal rim and a foramen on its anterior face (Figs 67B, 71).

The proximal end of metatarsal 1 is reduced proximally to a thin narrow splint (Figs 67B, 71). Compared to the proximal end of metatarsal 2, metatarsal 1 has approximately 20% of its transverse width and 30% of its anteroposterior depth. Santa Luca erroneously figured the proximal end of metatarsal 1 as roughly as broad as metatarsal 2. Metatarsal 1 extends proximally to contact the tarsus, where it fuses with the medial distal tarsal. Metatarsal 5, a dorsoventrally flattened sinuous splint, is preserved in articulation on the posterior aspect of the right tarsometatarsus (Table 9). Although Santa Luca described metatarsal 5 as positioned ventral to the proximal end of metatarsal 4, it preserves articular relations seen in other ornithischians and appears to be in natural articulation, along with all other elements of the right tarsometatarsus. Its proximal end articulates with the beveled posterior face of distal tarsal 4, and its shaft angles at about 45° mediodistally across metatarsal 4 to the ventral aspect of metatarsal 3.

**Figure 72.**
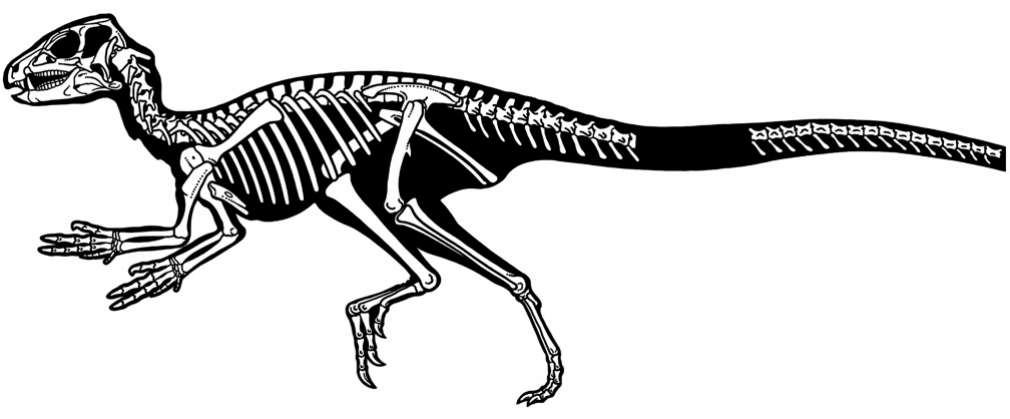
Skeleton of *Heterodontosaurus tucki* from the Lower Jurassic Upper Elliot and Clarens formations of South Africa. Silhouette skeletal reconstruction in lateral view showing preserved bones (based on SAM-PK-K1332). Distal most caudal vertebrae unknown.

**Table 7.** Measurements (mm) of the skull and axial column of the holotypic skeleton of the South African heterodontosaurid *Heterodontosaurus tucki* (SAM-PK-K1332). Except for the ninth cervical centrum and the chevrons, measurements are from Santa Luca (1984). Parentheses indicate estimated measurement.

**Region**	**Measurement**	
Cranium	Length, anterior tip to occipital condyle	115
	Height, parietal to quadrate condyle	66
Cervical vertebrae	C2 centrum length	16
	C3 centrum length	14
	C4 centrum length	16
	C5 centrum length	16
	C6 centrum length	13
	C7 centrum length	13
	C8 centrum length	13
	C9 centrum length	(13)
Dorsal vertebrae	D1 centrum length	(13)
	D2 centrum length	13
	D3 centrum length	14
	D4 centrum length	(13)
	D5 centrum length	15
	D6 centrum length	15
	D7 centrum length	15
	D8 centrum length	15
	D9 centrum length	15
	D10 centrum length	—
	D11 centrum length	(15)
	D12 centrum length	15
Sacral vertebrae	S1 centrum length	14
	S2 centrum length	(13)
	S3 centrum length	—
	S4 centrum length	—
	S5 centrum length	—
	S6 centrum length	14
Caudal vertebrae	CA1 centrum length	14
	CA2 centrum length	—
	CA3 centrum length	15
	CA4 centrum length	(15)
	CA5 centrum length	16
	CA6 centrum length	16
	CA7 centrum length	16
	CA8 centrum length	17
	CA9 centrum length	18
	CA10 centrum length	18
	CA11 centrum length	18
	CA12 centrum length	—
	?CA19 centrum length	16
	?CA20 centrum length	16
	?CA21 centrum length	16
	?CA22 centrum length	17
	?CA23 centrum length	16
	?CA24 centrum length	17
	?CA25 centrum length	16
	?CA26 centrum length	16
	?CA27 centrum length	16
	?CA28 centrum length	17
	?CA29 centrum length	16
	?CA30 centrum length	16
	?CA31 centrum length	—
	?CA32 centrum length	16
	?CA33 centrum length	16
	?CA34 centrum length	—
Chevrons	Ch1 length	23
	Ch2 length	23
	Ch3 length	22
	Ch4 length	23
	Ch5 length	23
	Ch6 length	24
	Ch7 length	24
	Ch8 length	24
	Ch9 length	22
	Ch10 length	17
	Ch11 length	15
	?Ch20 length	19
	?Ch21 length	20
	?Ch22 length	19
	?Ch23 length	17
	?Ch24 length	18
	?Ch25 length	15
	?Ch26 length	15
	?Ch27 length	17
	?Ch28 length	16
	?Ch29 length	15
	?Ch30 length	15
	?Ch31 length	16

**Table 8. T8:** Measurements (mm) of the pectoral girdle and forelimb bones of the holotypic skeleton of the South African heterodontosaurid *Heterodontosaurus tucki* (SAM-PK-K1332). Except for the bones of manual digit V, measurements are from Santa Luca (1984), with right and left sides averaged when both are available. Parentheses indicate estimated measurement.

**Structure**	**Measurement**	
Scapula	Blade length	86
	Blade, minimum width of neck	8
	Blade, maximum width at distal end	22
Humerus	Length	83
	Proximal end, maximum width	21
	Deltopectoral crest length	35
	Shaft, minimum diameter	7
	Distal end, maximum width	19
Ulna	Length	68
	Shaft, minimum diameter	4
Radius	Length	58
	Shaft, minimum diameter	4
Manual digit I	Metacarpal 1 length	18
	Phalanx I-1 length	17
	Ungual I-2 length	17
Manual digit II	Metacarpal 2 length	23
	Phalanx II-1 length	15
	Phalanx II-2 length	17
	Ungual II-3 length	18
Manual digit III	Metacarpal 3 length	22
	Phalanx III-1 length	14
	Phalanx III-2 length	12
	Phalanx III-3 length	15
	Ungual III-4 length	17
Manualdigit IV	Metacarpal 4 length	15
	Phalanx IV-1 length	7
	Phalanx IV-2 length	5
Manual digit V	Metacarpal 5 length	7
	Phalanx V-1 length	6
	Phalanx V-2 length	4

**Table 9.** Measurements (mm) of the pelvic girdle and hind limb bones of the holotypic skeleton of the South African heterodontosaurid *Heterodontosaurus tucki* (SAM-PK-K1332). Except for metatarsal 5, measurements are from Santa Luca (1984), with right and left sides averaged when both are available.

**Structure**	**Measurement**	
Ilium	Blade length	97
	Blade, height dorsal to acetabular rim	15
	Preacetabular process length	46
	Postacetabular process length	24
Femur	Length	112
	Minimum shaft diameter	9
	Head to distal end of fourth trochanter	46
Tibiotarsus	Length	145
	Minimum shaft diameter	9
Pedal digit I	Metatarsal 1 length	38
	Phalanx I-1 length	17
	Ungual I-2 length	18
Pedal digit II	Metatarsal 2 length	59
	Phalanx II-1 length	19
	Phalanx II-2 length	16
	Ungual II-3 length	21
Pedal digit III	Metatarsal 3 length	68
	Phalanx III-1 length	22
	Phalanx III-2 length	16
	Phalanx III-3 length	14
	Ungual III-4 length	18
Pedal digit IV	Metatarsal 4 length	61
	Phalanx IV-1 length	17
	Phalanx IV-2 length	12
	Phalanx IV-3 length	11
	Phalanx IV-4 length	10
	Ungual IV-5 length	16
Pedal digit V	Metatarsal 5	15

#### Lycorhinus angustidens

Haughton, 1924

http://species-id.net/wiki/Lycorhinus_angustidens

Figs 3 73 [Fig F74] [Fig F75] [Fig F76] [Fig F77] [Fig F78] [Fig F79] 80 Tables 1 [Table T2] 3

Lycorhinus angustidens Haughton, 1924 – Thulborn (1970, Figs 1–5); Gow (1975, 336, Figs 1, 2, pl. 1); Hopson (1975, Figs 1, 2, 3a, b, e); Hopson (1980, Figs 2, 3); Weishampel (1984, fig. 12a, b); Gow (1990, Figs 1–7); Porro et al. (2011, Figs 4-6); Norman et al. (2011, Figs 37B, 38, 39C)
= Lanasaurus scalpridens Gow 1975

##### Holotype.

SAM-PK-K3606, left dentary with 11 teeth (preserved mostly as a natural mold; UCRC PVC10 was cast from the natural mold; Fig. 3D)

##### Referred material.

NHMUK RU A100 (formerly BMNH A100), partial skull preserving the right premaxilla, right nasal, right maxilla, the posterior two-thirds of the right lower jaw, and the anterior third of the left lower jaw; BP/1/4244, left maxilla with 12 teeth (holotype *Lanasaurus scalpridens*); BP/1/5253, left maxilla with 15 teeth.

##### Type locality.

Paballong, near Mount Fletcher, Transkei (Herschel) District, Cape Province, South Africa; S30°26', E28°31' (Kitching and Raath 1984) (Fig. 1B).

##### Horizon.

Upper Elliot Formation; Lower Jurassic, Hettangian, ca. 200-196 Ma (Thulborn 1970; Gow 1990; Knoll 2005; Gradstein and Ogg 2009).

##### Revised diagnosis.

Heterodontosaurid ornithischian characterized by the following two autapomorphies: (1) prominent ridge on the distal crown margin running from the first denticle to the cingulum (labial aspect of maxillary crowns, lingual aspect of dentary crowns); (2) lingually curved maxillary and dentary tooth rows.

##### Description.

The validity of *Lycorhinus angustidens* and referral of an important partial skull (NHMUK RU A100; Thulborn 1970) have been at the center of debate between several authors (Thulborn 1974, Charig and Crompton 1974; Hopson 1975, Gow 1975, 1990). In the course of this argument, the holotype of *Lycorhinus angustidens* has been described in detail (Hopson 1975; Gow 1990). The same cannot be said for the partially disarticulated skull of NHMUK RU A100. Several misidentifications in Thulborn’s original description have gone unnoticed. Proper referral of this specimen is critical, as the holotype for *Lycorhinus angustidens* is limited to a single dentary that now is preserved largely as an impression (Hopson 1975; Gow 1990; Fig 3). Here I describe the partial skull NHMUK RU A100 and later discuss the history and resolution of the taxonomy of *Lycorhinus* (see Discussion, Taxonomy of *Lycorhinus*).

##### Specimen map.

**Figure 73.**
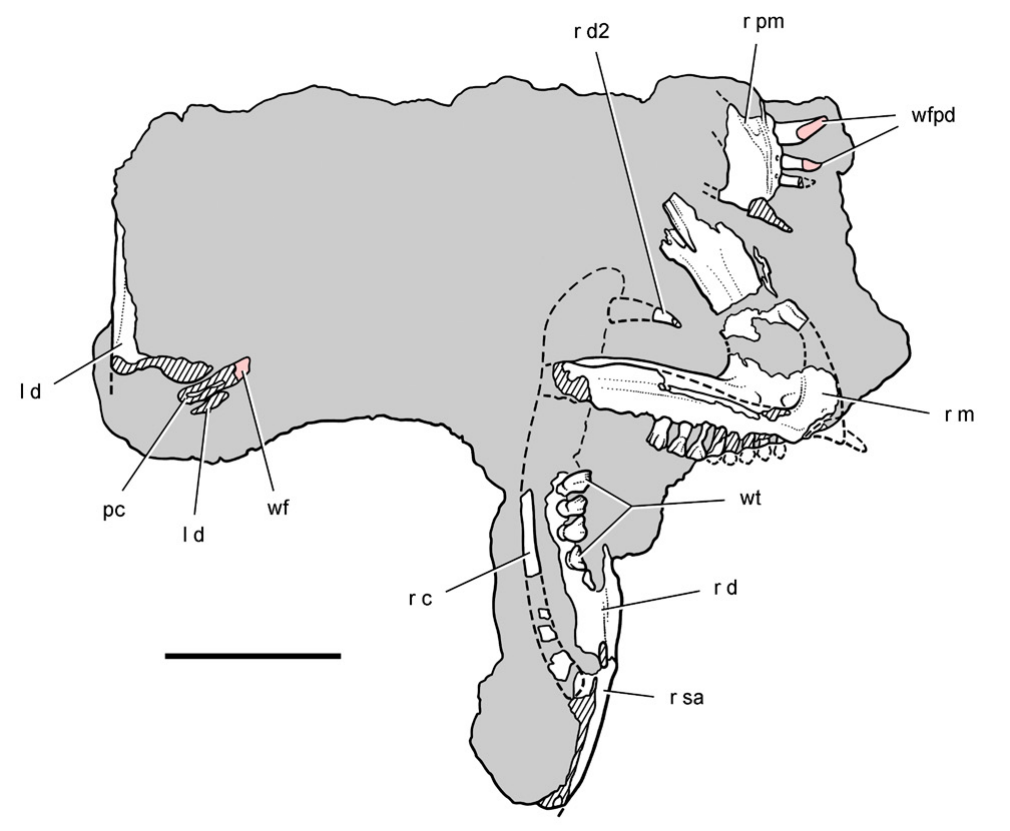
Block with remains of the heterodontosaurid *Lycorhinus angustidens* from the Lower Jurassic Upper Elliot Formation of South Africa. Top view of block showing the right premaxilla and maxilla and portions of the right and left lower jaws (NHMUK RU A100). Hatching indicates broken bone; dashed lines indicate estimated edges; grey tone indicates matrix; pink tone indicates wear facets. Scale bar equals 3 cm. Abbreviations: ***c*** coronoid ***d*** dentary ***d2*** dentary tooth 2 ***l*** left ***m*** maxilla ***pc*** pulp cavity ***pm*** premaxilla ***r*** right ***sa*** surangular ***wf*** wear facet ***wfpd*** wear facet from predentary bill ***wt*** worn teeth.

**Figure 74. F74:**
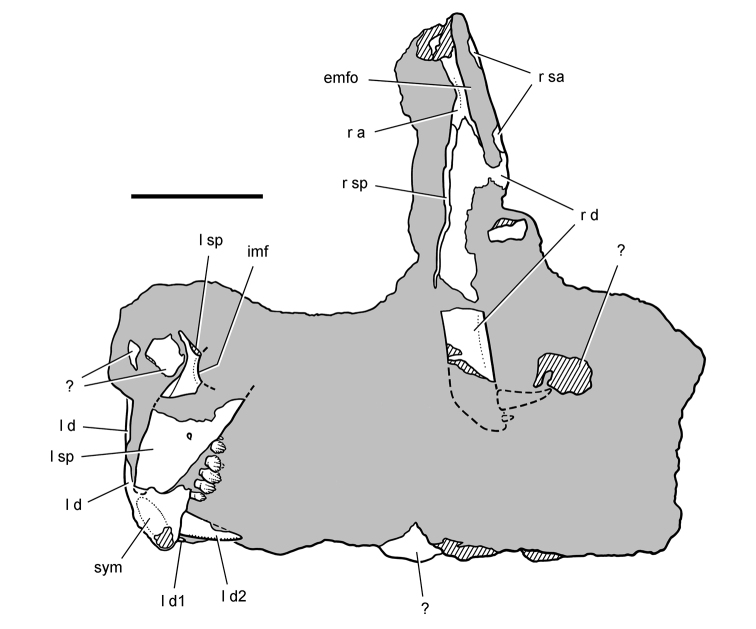
Block with remains of the heterodontosaurid *Lycorhinus angustidens* from the Lower Jurassic Upper Elliot Formation of South Africa. Bottom view of block (NHMUK RU A100) showing portions of the right and left lower jaws. Hatching indicates broken bone; dashed lines indicate estimated edges; tone indicates matrix. Scale bar equals 3 cm. Abbreviations: ***a*** angular ***d*** dentary ***d1***, ***2*** dentary tooth 1, 2 ***emfo*** external mandibular fossa ***imf*** internal mandibular fenestra ***l*** left ***r*** right ***sa*** surangular ***sp*** splenial ***sym*** symphysis.

The NHMUK RU A100 is preserved on a single block of matrix with bone exposed on both sides. In its current state of preparation, the block preserves the right premaxilla (medial view), right maxilla (lateral view), anterior end of the left lower jaw (medial view), and mid and posterior parts of the right lower jaw (partial medial and lateral views) (Figs 73, 74). Thulborn (1970) misidentified a portion of the right maxilla as part of a nasal, the right lower jaw as a partial cranium, and the anterior end of the left lower jaw (medial view) as the end of the right lower jaw (lateral view) (Fig. 4). First I provide evidence for identification of these bones as shown here (Figs 73, 74).

**Figure 75. F75:**
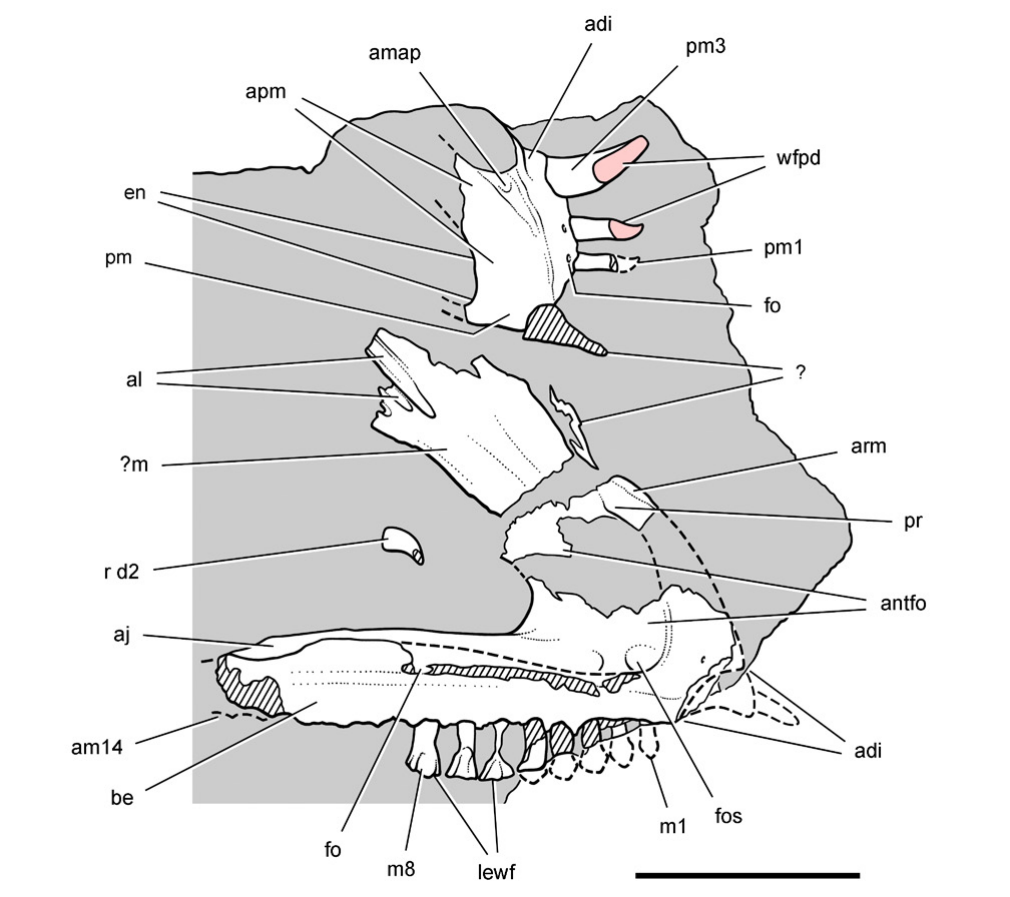
Upper jaw of the heterodontosaurid *Lycorhinus angustidens* from the Lower Jurassic Upper Elliot Formation of South Africa. Premaxilla and maxilla of an adult individual with worn teeth (NHMUK RU A100). Right premaxilla and maxilla in medial and lateral views, respectively. Hatching indicates broken bone; dashed lines indicate estimated edges; grey tone indicates matrix; pink tone indicates wear facets. Scale bar equals 2 cm. Abbreviations: ***adi*** arched diastema ***aj*** articular surface for the jugal ***al*** articular surface for the lacrimal ***am14*** alveolus for maxillary tooth 14 ***amap*** articular surface for the maxillary anterior process ***antfo*** antorbital fossa ***apm*** articular surface for the opposing premaxilla ***arm*** ascending ramus of the maxilla ***be*** buccal emargination ***d2*** dentary tooth 2 ***en*** external nares ***fo*** foramen ***fos*** fossa ***lewf*** leading edge of the wear facet ***m*** maxilla ***m1***, ***8*** maxillary tooth 1, 8 ***pm*** premaxilla ***pm1***, ***3*** premaxilla tooth 1, 3 ***pr*** process ***r*** right ***wfpd*** wear facet from predentary bill.

A flat broad bone next the right maxilla is here tentatively identified as the posterodorsal ramus of the maxilla, preserved near its natural position relative to the remainder of the bone (Figs 75, 77B). It is difficult to envision this platelike bone as the nasal as identified by Thulborn (Fig. 4), as it is very broad (especially for a heterodontosaurid), lacks a median suture, and shows a fluted surface posteriorly that is difficult to understand as an articular surface for the frontal. The posterodorsal ramus of the maxilla is very broad as preserved in NHMUK RU A100 and BP/1/4244 (Gow 1975). Most of the surface is devoted to the antorbital fossa, which in heterodontosaurids approaches the nasal suture. The relatively flat bone piece in question, however, does not have any external rim between the surface of the fossa and the edge in contact with the nasal. As a result I tentatively identify the bone as a continuation of the maxilla, although it was not included in the skull reconstruction (Fig. 80). The identity of the bone could be determined with more confidence after further preparation of the specimen.

The left lower jaw (Figs 73, 74) preserves the anterior portion of the splenial broadly overlapping the medial aspect of the dentary, a raised dentary symphysis, and anteromedially canted crowns that are exposed in medial view. Despite the assertion that no crowns are worn (Thulborn 1970: 240), almost all of the preserved left dentary crowns are truncated by prolonged tooth-to-tooth abrasion. Only the truncated apical edge on some of the anterior dentary crowns is visible, however, because the left dentary teeth are exposed in medial view (Fig. 74). The axis of each exposed left dentary tooth is medially convex with thicker, orange-tinted enamel as is typical of the medial side of dentary crowns. The last preserved left dentary tooth, which is exposed on the other side of the block, preserves a broad, low-angle, wear facet on the lateral side of the crown (Fig. 73).

The more complete right lower jaw is also exposed on both sides of the block (Figs 73, 74). On one side of the block, the right lower jaw is broadly exposed in lateral view (Fig. 74). Many observations support the identification of these bones as the right lower jaw rather than a partial cranium in dorsal view as suggested by Thulborn (Fig. 4). The buccal emargination is on the lateral aspect of the dentary (previously identified as a left frontal), there are sutures typical of that between the surangular, angular and dentary, a strong surangular ridge is present (previously considered part of the left jugal), and an external mandibular fossa is present as in *Heterodontosaurus* (previously identified as the laterotemporal fenestra). Finally, although the anterior end of the right lower jaw is embedded in matrix, the enlarged dentary caniniform tooth passes through to the opposite side of the block, its tip emerging near the right maxilla (Figs 73, 74). The description of the frontal, postorbital and jugal by Thulborn (1970) and remarks concerning the postorbital-jugal suture by Weishampel (1984: 44), thus, were based on the right lower jaw of NHMUK RU A100.

With the identifications proposed here, there is no reason to doubt that NHMUK RU A100 represents a single, probably adult individual with worn upper and lower cheek of similar form, worn premaxillary teeth, and large upper and lower caniniform teeth. The specimen preserves the right side of the snout and lower jaw and a matching anterior portion of the left lower jaw. This important specimen, the salient features of which are outlined below, should be fully prepared and described in more detail.

##### Premaxilla.

**Figure 76. F76:**
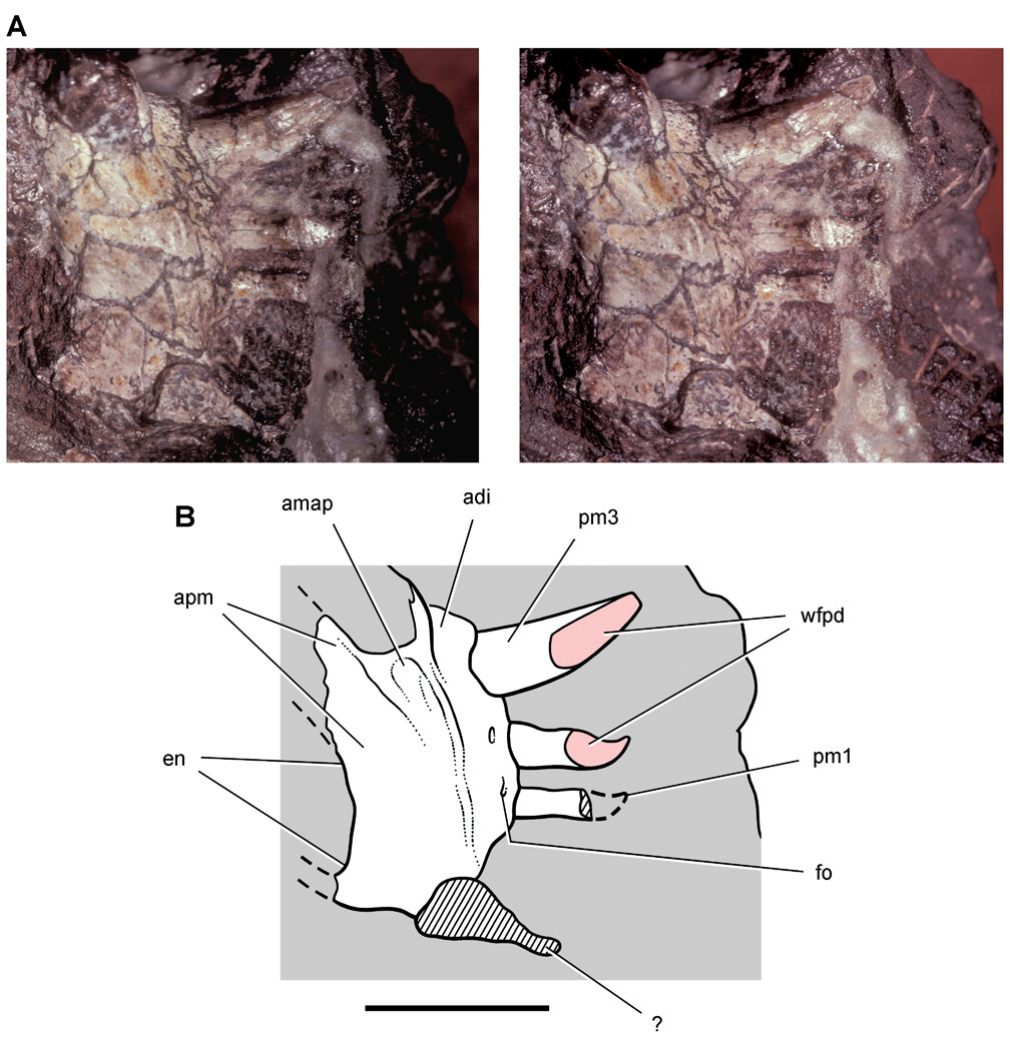
Premaxilla of the heterodontosaurid *Lycorhinus angustidens* from the Lower Jurassic Upper Elliot Formation of South Africa. Premaxilla of an adult individual with worn teeth (NHMUK RU A100). Stereopair (**A**) and line drawing (**B**) of the right premaxilla in medial view. Hatching indicates broken bone; dashed lines indicate estimated edges; grey tone indicates matrix; pink tone indicates wear facets. Scale bar equals 1 cm. Abbreviations: ***adi*** arched diastema ***amap*** articular surface for the maxillary anterior process ***apm*** articular surface for the opposing premaxilla ***en*** external nares ***fo*** foramen ***pm1***, ***3*** premaxilla tooth 1, 3 ***wfpd*** wear facet from predentary bill.

The right premaxilla is preserved in ventromedial view, exposing the median articular surface for the left premaxilla and the palatal shelf medial to the premaxillary teeth (Figs 73, 75, 76). The internarial and posterolateral processes are broken, the former very narrow and the latter quite broad as in *Abrictosaurus* and *Heterodontosaurus*. The only lateral exposure of the premaxilla occurs along its posterior margin, where the anterior portion of an arched, inset upper diastema is preserved (Fig. 76). The arched diastema for the dentary caniniform tooth is broadly open laterally. A secondary lateral wall enclosing a space within the diastema is initiated in the upper half of the premaxillary margin of the diastema and continues onto the maxilla as in *Heterodontosaurus* (Fig. 75).

The palatal surface is flat with a near horizontal orientation as in *Heterodontosaurus* and most ornithischians. Judging from the location of the anterior portion of the arched diastema, the palate and premaxillary tooth row would have been set below the maxillary tooth row. Just above the posterior end of the palate, an anteriorly tapering slot accommodates the anteromedial process of the maxilla (Fig. 76). This maxillary process, therefore, inserts above the palate as in *Heterodontosaurus* (Norman et al. 2011), *Hypsilophodon* (Galton 1974a), and many other ornithischians, rather than against the ventral surface of the premaxillary palate as previously suggested (*contra* Weishampel 1984: 34). The posterior end of the palatal surface turns sharply upward to join the anterior portion of the arched diastema. Two small foramina are present near the bases of the first and second premaxillary teeth. Although it is possible that they represent replacement foramina, only two are present (rather than three). Unlike replacement foramina, in addition, they are not positioned immediately adjacent to premaxillary alveoli and lack a connecting groove for the dental lamina. It is probable, therefore, that they represent neurovascular foramina associated with the palate, an interpretation that could be verified with computed-tomographic imaging of the bone.

##### Maxilla.

**Figure 77. F77:**
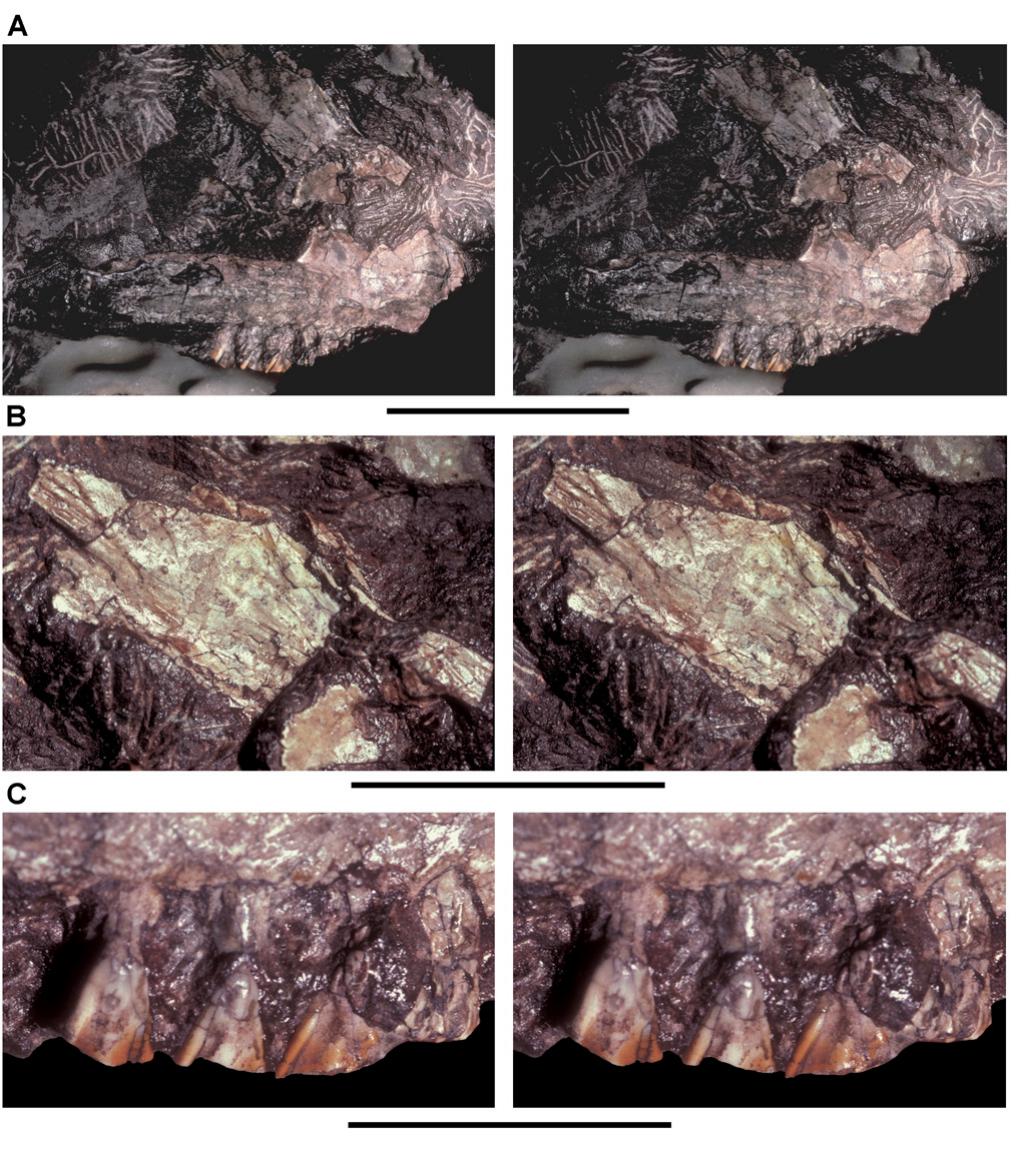
Maxilla of the heterodontosaurid *Lycorhinus angustidens* from the Lower Jurassic Upper Elliot Formation of South Africa. Maxilla of an adult individual with worn teeth (NHMUK RU A100). **A** Stereopair of the right maxilla in lateral view **B** Close-up view of the ascending ramus **C** Close-up view of the worn crowns of maxillary teeth 6–8. Scale bars equal 2 cm in **A**, 3 cm in **B**, and 1 cm in **C**.

The right maxilla, which is broadly exposed in lateral view (Figs 73, 75, 77A), has sustained some fracturing and loss during the time of burial as well as some breakage after exposure of the fossil. Fracturing and loss during burial appears to have isolated the base of the posterodorsal ramus of the maxilla, leaving it near its natural position relative to the main body of the maxilla (Fig. 75). The distal portion of this ramus may also be preserved near its natural position, although the identification of this plate-shaped bone remains tentative (Figs 75, 77B).

The body of the maxilla thickens transversely from the alveolar margin to the ventral rim of the antorbital fenestra, which protrudes laterally as in other heterodontosaurids (Figs 75, 77A). Several neurovascular foramina lie just under this rim, which forms the upper margin of a deeply inset buccal emargination (Fig. 80). Dorsal to the rim, the smooth medial wall of the antorbital fossa is exposed, which increases in depth anteriorly. Near the anteroventral corner of the antorbital fossa are located two oval depressions (Figs 77A, 80). The broadly arched anteroventral corner of the antorbital fossa is not invaginated as occurs in *Abrictosaurus* and *Heterodontosaurus*.

The edges of the arched diastema and the anterior end of the anteromedial process of the maxilla are broken away (Figs 75, 77A). This portion of the maxilla is best preserved in an isolated referred left maxilla (Gow 1975, 1990). In NHMUK RU A100, some of the inset medial wall of an arched diastema is preserved. The posterodorsal ramus is divided between a raised external margin and the recessed medial wall of the antorbital fossa (Figs 75, 77A). Unlike *Abrictosaurus* and *Heterodontosaurus*, the fossa is not deeply invaginated, and the raised external margin separates the antorbital fossa from the premaxilla by a greater distance. A low prominence is present about halfway along the anterior margin of the antorbital fossa (Fig. 80).

**Figure 78. F78:**
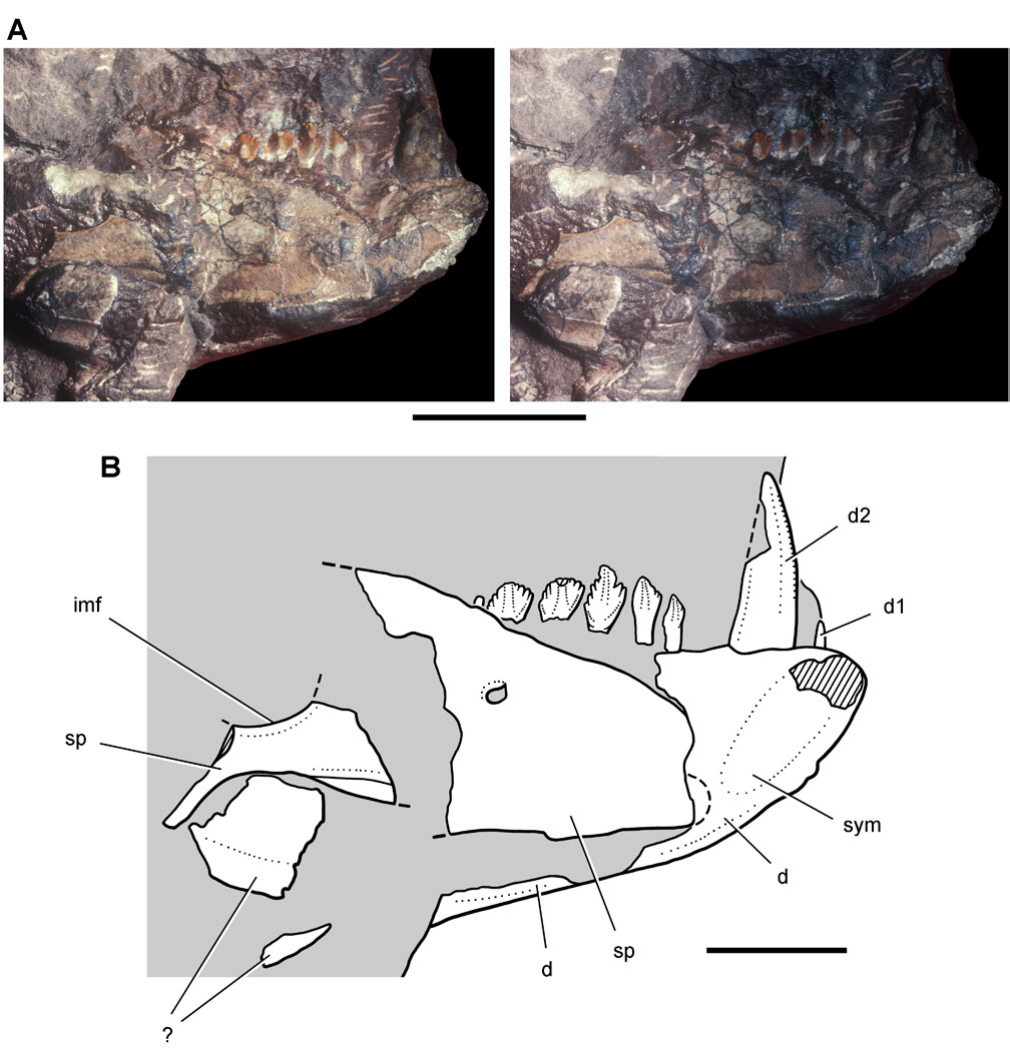
Lower jaw of the heterodontosaurid *Lycorhinus angustidens* from the Lower Jurassic Upper Elliot Formation of South Africa. Anterior end of the lower jaw of an adult individual (NHMUK RU A100). Stereopair (**A**) and line drawing (**B**) of the anterior end of the left lower jaw in medial view. Hatching indicates broken bone; dashed lines indicate estimated edges; tone indicates matrix. Scale bars equal 2 cm in A and 1 cm in B. Abbreviations: ***d*** dentary ***d1***, ***2*** dentary tooth 1, 2 ***imf*** internal mandibular fenestra ***sp*** splenial ***sym*** symphysis.

##### Lower jaw.

**Figure 79. F79:**
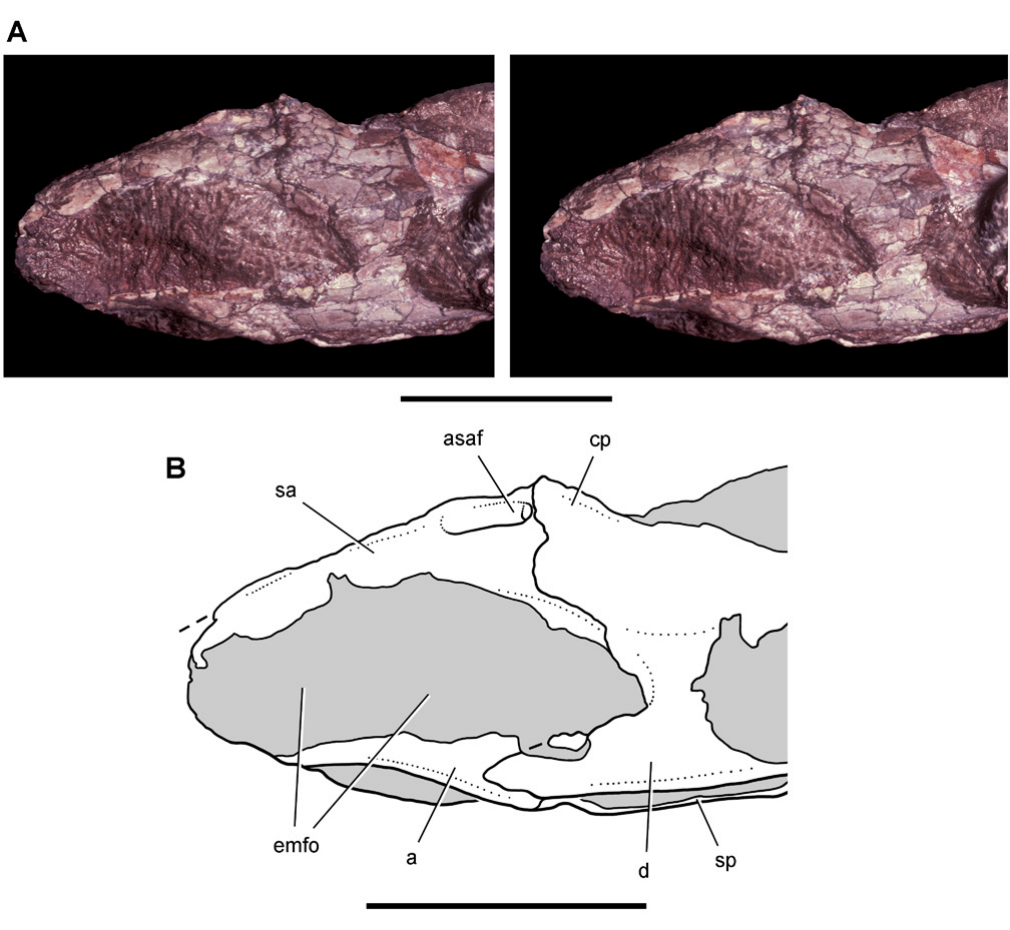
Lower jaw of the heterodontosaurid *Lycorhinus angustidens* from the Lower Jurassic Upper Elliot Formation of South Africa. Posterior end of the lower jaw of an adult individual (NHMUK RU A100). Stereopair (**A**) and line drawing (**B**) of the posterior end of the right lower jaw in lateral view. Dashed lines indicate estimated edges; tone indicates matrix. Scale bars equal 2 cm. Abbreviations: ***a*** angular ***asaf*** anterior surangular foramen ***cp*** coronoid process ***d*** dentary ***emfo*** external mandibular fossa ***sa*** surangular ***sp*** splenial.

The right lower jaw is exposed mainly in lateral view and is complete except for the retroarticular region and small areas of breakage (Figs 74, 79). The anterior end, however, remains embedded in the matrix, and information on this end of the lower jaw comes from the left side, which is exposed in medial view (Figs 74, 78). There is some chance that the predentary is hidden within the matrix of the block, given that both dentary rami are present.

**Figure 80.**
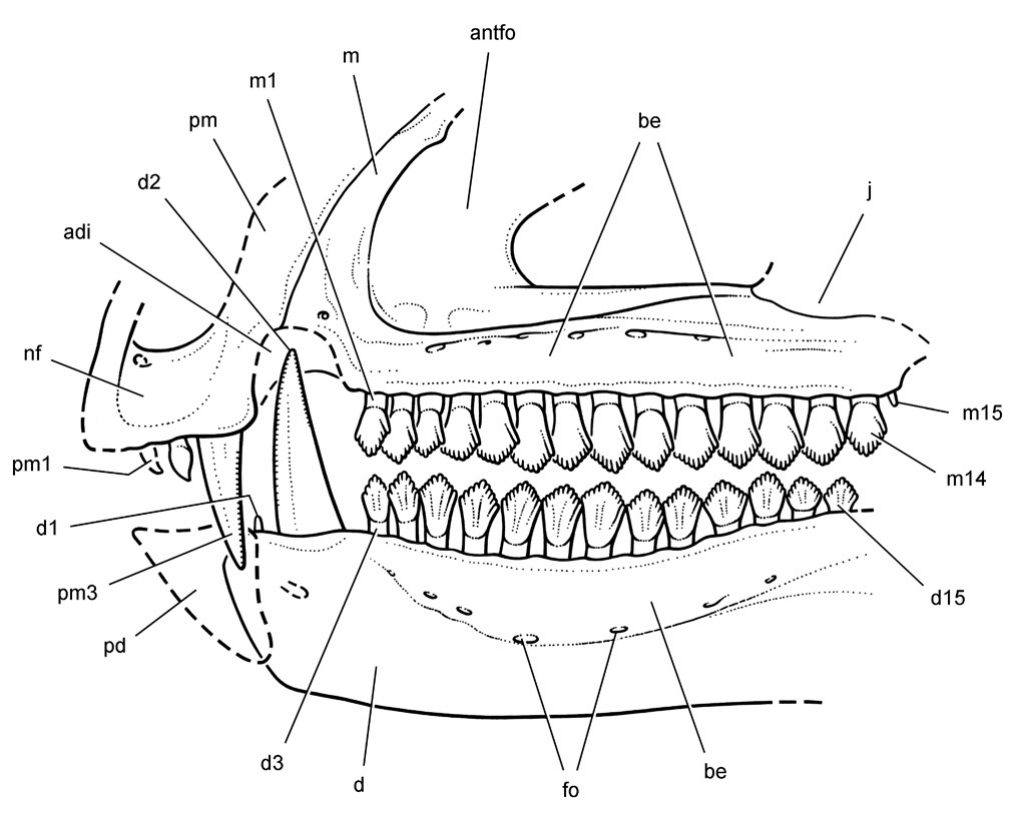
Skull of the heterodontosaurid *Lycorhinus angustidens* from the Lower Jurassic Upper Elliot Formation of South Africa. Skull reconstruction based on the holotypic dentary (SAM-PK-K3606) and referred specimens (NHMUK RU A100, BP/1/4244). Dashed lines indicate estimated edges. Abbreviations: ***adi*** arched diastema ***antfo*** antorbital fossa ***be*** buccal emargination ***d*** dentary ***d1-3***, ***15*** dentary teeth 1–3, 15 ***fo*** foramen ***j*** jugal ***m*** maxilla ***m1***, ***14***, ***15*** maxillary tooth 1, 14, 15 ***nf*** narial fossa ***pd*** predentary ***pm*** premaxilla ***pm1***, ***3*** premaxilla tooth 1, 3.

The dentary is a robust bone approximately 17 mm in depth at mid-length and some 70 mm in length. The ventral margin of the ramus is thickened, and the tooth row is inset by a ventrally arched, deep buccal emargination (Fig. 80). Although partially visible in NHMUK RU A100, the lateral aspect of the dentary is best exposed in the holotype (Fig. 3D). Anteriorly, the dentary curves toward an oval symphysis (Fig. 78) that is deeper than that in *Heterodontosaurus* (Fig. 61A). In medial view the end of the dentary is beveled anteroventrally (Fig. 78), unlike the rounded and expanded end of the dentary in *Abrictosaurus* and *Heterodontosaurus* (Figs 35, 59).

Medially the dentary is overlapped by a broad, tongue-shaped splenial, which appears to have been displaced anteriorly toward the symphysis on the right side (Fig. 78), and a long strap-shaped coronoid (Fig. 73) similar to that in *Heterodontosaurus* (Fig. 56). The coronoid curves posterodorsally to overlap the junction between the dentary and surangular on the coronoid process. An external mandibular fossa occupies the central portion of the surangular and angular below the coronoid process and is filled with matrix (Fig. 79). Additional preparation is needed to determine if a mandibular fenestra is present at the anterior end of this fossa, as occurs in *Heterodontosaurus*.

The retroarticular region is broken away, so the exact location of the jaw articulation is unknown. Judging from the preserved margin of the surangular and the location of the dentary tooth row, the jaw articulation was probably offset ventrally at least as much as preserved in *Abrictosaurus* (Fig. 35).

##### Premaxillary teeth.

The three premaxillary teeth (pm1-3) are preceded by an edentulous margin approximately one-third of the length of the ventral margin of the premaxilla (Figs 76, 80). The first two premaxillary crowns may have slid partially out of their sockets. A gentle constriction is present on these teeth where the crown and root meet, and the second more complete crown is clearly recurved. A broad wear facet on pm2 may have obliterated the midline of the crown. No ornamentation is visible as preserved. The basal swelling of the crown of pm2 and posterior deflection of its tip resembles the form of pm2 in *Heterodontosaurus* (Fig. 91) and pm1 in *Lesothosaurus* (Sereno 1991: fig. 6C). Thulborn (1970: 239) described the crowns of pm1 and 2 as “conical pegs”.

The large, caniniform third premaxillary tooth lacks any constriction between the crown and root (Fig. 76). The crown base is cylindrical but flattens distally with development of anterior and posterior carinae. The smooth anterior carina is convex in lateral view, whereas the posterior carina is nearly straight. The crown apex thus is only slightly recurved (Fig. 80). The straight posterior carina has about five serrations per millimeter as noted by Thulborn (1970).

The wear facets on pm2 and 3 are planar but are not “flat” as described by Thulborn (1970: 239). The facets are mesiodistally flat but concave apicobasally (Fig. 76). These facets are the result of shearing wear against the straight keratinous edge of the predentary bill. The tip of the pm3, in addition, is broken along a horizontal fracture as preserved in matrix. The crown tip thus either was broken in life or during transport after death. Wear alone cannot account for the straight edge that truncates the tip of this caniniform tooth. Breakage and subsequent apical abrasion characterizes the teeth of adult *Heterodontosaurus*, as described in more detail below.

##### Maxillary teeth.

The right maxilla of NHMUK RU A100 has alveoli for 14 fully developed teeth (Fig. 75) rather than 13 as described by Thulborn (1970: 239). A count of 14 fully developed maxillary teeth matches that in another complete maxillary tooth row, which has one peglike distalmost crown (Gow 1990). Judging from the empty first alveolus in NHMUK RU A100, m1 is smaller than more posterior maxillary teeth as preserved in a referred maxilla (Gow 1975). An edentulous margin about the width of one tooth is present between the arched diastema and first maxillary tooth in NHMUK RU A100 (Fig. 75). The edentulous margin is much shorter in referred maxillae (Gow 1975, 1990). Maxillary crowns are set at an angle to the root and are subject to considerable wear against the dentary crowns (Fig. 77C). Wear facets truncate the distal portion of all of the preserved maxillary crowns. Unlike *Abrictosaurus* and *Heterodontosaurus*, all but the most mesial and distal maxillary crowns are fairly similar in size and shape (Fig. 80).

The distinguishing feature of the lateral aspect of the maxillary crowns is the accentuated distal edge, which is raised as a distal marginal ridge (Figs 10, 77C). The dentary crowns, in contrast, are more symmetrical as seen in lingual view (Fig. 78). A rounded median eminence is present but not developed into a distinct primary ridge, and no secondary ridges are present on either maxillary or dentary crowns. The medial aspect of the maxillary crowns is not exposed; the description of the medial side of the maxillary crowns by Thulborn (1970: 240) is based on misidentification of the dentary as a maxilla (Fig. 4B). Besides the distal marginal ridge, the only other distinguishing feature in the maxillary dentition is the medial curvature of the tooth row (Gow 1990). In *Echinodon*, *Abrictosaurus* and *Heterodontosaurus*, in contrast, the maxillary tooth row is nearly straight.

##### Dentary teeth.

The posterior portion of a small dentary tooth (d1) is preserved anterior to the caniniform tooth (Fig. 78). Its crown appears to have been subconical or peglike with a rounded distal margin. Although figured by Thulborn (1970: fig. 5), this tooth was not described. The second dentary tooth (d2) is caniniform, with anterior and posterior carinae. The crown is more than twice the height of dentary crowns in the center of the tooth row (Fig. 80). The premaxillary caniniform tooth (pm 3) is approximately 70% the height and basal width of d2. Unlike pm3, d2 is noticeably recurved with convex and concave carinae anteriorly and posteriorly, respectively (Fig. 78). The mesial carina of d2 is serrate, whereas the distal carina is not fully exposed. The supposed absence of serrations on the distal carina (Thulborn 1970) thus cannot be used to differentiate NHMUK RU A100 from the caniniform in the holotype, which has a serrate distal carina (Hopson 1975; Gow 1990). An edentulous margin the width of one tooth is situated between the dentary caniniform tooth and d3 in NHMUK RU A100 (Fig. 78), which closely matches the condition in the holotypic dentary of *Lycorhinus angustidens* (Fig. 3).

Eleven postcaniniform teeth (d3-13) are present in the holotypic dentary, which lacks the distalmost teeth. In NHMUK RU A100, the first and second postcaniniform crowns (d3, 4) are smaller and proportionately taller than more distal dentary crowns (Fig. 78). The tip of the crown of d5 is truncated by shearing wear on the unexposed labial side of the crown. More substantial wear is present on d6 and 7 that truncates more of the crown tip. Thinking they were maxillary teeth, Thulborn (1970: 240) reported that all of the exposed dentary crowns are unworn. His lower count of only two or three denticles on mesial and distal crown margins, rather than four or five, is based on dentary crowns truncated by wear facets.

The lingual side of the dentary crowns has a prominent, slightly mesially offset, vertical eminence that is stronger than that on the labial aspect of the maxillary crowns (Figs 77C, 78). Unlike the labial surface of the maxillary crowns, neither margin is raised as a ridge nor are the crowns tilted relative to the axis of the root. The labial side of the dentary crowns is exposed in the holotype but all are heavily worn (Fig. 3). The fourth postcaniniform tooth in the holotype dentary (Fig. 3C; labeled “4”)—the only one preserving some of the crown edge—has a prominent distal marginal ridge comparable to that on the labial aspect of the maxillary crowns in NHMUK RU A100 (Fig. 77C). A rounded median eminence is present which joins a labial prominent, swollen cingulum, which is well-demarcated form the root. Thus the labial aspect of the dentary crowns in the holotype of *Lycorhinus angustidens* bears a striking resemblance to the labial aspect of the maxillary crowns in NHMUK RU A100. In both the cingulum is swollen and there is a prominent distal marginal ridge.

The roots of the dentary teeth in NHMUK RU A100 appear to be swollen. The left dentary and one of its posterior teeth are exposed in cross-section (Fig. 73). Although there is a gentle constriction below the crown, the hollow root expands to a width equal to that of the crown, resembling the condition in *Abrictosaurus* and *Heterodontosaurus*. Hopson (1975: 304) also noted swelling of the roots of the dentary teeth in the holotype of *Lycorhinus angustidens*.

Maxillary and dentary crowns have an imbricate arrangement with the mesial edges of the crowns angled mesiolingually. Although some adjacent crowns are in contact, there is little overlap of crown edges in either the holotypic dentition, the tooth rows of NHMUK RU A100, or the two referred maxillae. As described by Gow (1990) in the worn dentition of the holotype, wear facets in *Lycorhinus* do not join to form a single surface nor are they oriented in a single occlusal plane (*contra* Weishampel 1984: 54). The enamel on the dentary crowns is distributed asymmetrically with a thicker layer on the lingual side in NHMUK RU A100. A similar asymmetrical distribution of enamel (reversed) is probably present in maxillary crowns but cannot be unequivocally demonstrated.

##### Skull reconstruction.

The partial skull reconstruction (Fig. 80) is the first for *Lycorhinus angustidens*. Previously Gow (1990: fig. 3) had juxtaposed the maxillary and dentary tooth rows of BP/1/5253 and SAM-PK-K3606. The reconstruction presented here is based on the holotypic dentary (UCRC PVC10; silicone cast from the natural mold of (SAM-PK-K3606; Hopson 1975), two referred maxillae (BP/1/4244, BP/1/5253; Gow 1975, 1990), and specimen NHMUK RU A100. The premaxilla and maxilla of the new reconstruction are based primarily on NHMUK RU A100. The anterior end of the dentary in NHMUK RU A100 is exposed only in medial view, and so the lateral aspect of the dentary is based on the holotypic natural mold (Hopson 1975; Gow 1990). The dentary of NHMUK RU A100, however, shows that its anterior end is less expanded and lacks the rounded profile of that in *Heterodontosaurus* and *Abrictosaurus* (Figs 35, 59). The predentary is not preserved in available material of *Lycorhinus*.

The premaxillary teeth, which are based on NHMUK RU A100, are inset from the anterior margin of the premaxilla with the first two premaxillary teeth set back into their sockets. The premaxillary tooth row is positioned below the level of the maxillary tooth row (Fig. 80), judging from the low position of the maxillary anteromedial process (BP/1/4244; Gow 1975: fig. 1) and the slot on the premaxilla for that process (Fig. 76). Maxillary and dentary crown shape is best preserved in BP/1/4244 and NHMUK RU A100, respectively (Figs 77C, 78). The maxillary tooth count of 15 (the last a rudimentary crown) is based the nearly complete tooth rows of NHMUK RU A100 and BP/1/1523 (Gow 1990). The dentary tooth count of 15 is an estimate. The holotype preserves 11 dentary teeth posterior to the caniniform tooth for a total of 13 teeth (d3-13), but the posterior end of the row is probably lacking one or two teeth. The right dentary in NHMUK RU A100 may well preserve a complete tooth row but is not fully exposed. Dentary crown shape is based on the exposed and unworn anterior dentary crowns in NHMUK RU A100 (Fig. 78).

#### Manidens condorensis

http://species-id.net/wiki/Manidens_condorensis

Figs 8 81 Tables 1 3

Manidens condorensis Pol et al. (2011; Figs 1, 2)

##### Holotype.

**Figure 81.**
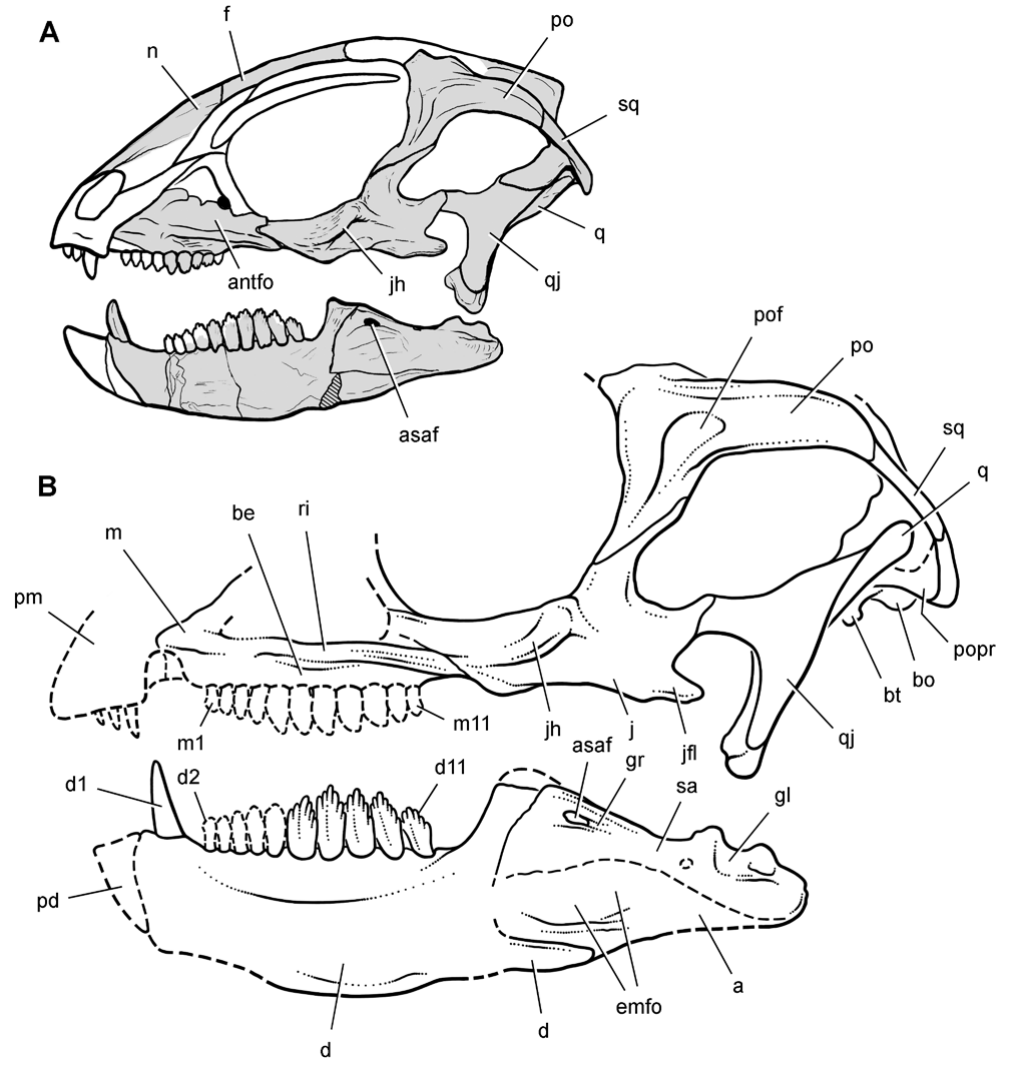
Partial skull of *Manidens condorensis* from the Middle Jurassic Cañadón Asfalto Formation of Argentina. Skull reconstructions in lateral view **A** Reversed from Pol et al. (2011) **B** This study. Dashed lines indicate estimated edges. Abbreviations: ***a*** angular ***antfo*** antorbital fossa ***asaf*** anterior surangular foramen ***be*** buccal emargination ***bo*** basioccipital ***bt*** basal tubera ***d*** dentary ***d1***, ***2***, ***11*** dentary tooth 1, 2, 11 ***emfo*** external mandibular fossa ***f*** frontal ***gl*** glenoid ***gr*** groove ***j*** jugal ***jfl*** jugal flange ***jh*** jugal horn ***m*** maxilla ***m1***, ***11*** maxillary tooth 1, 11 ***n*** nasal ***pd*** predentary ***pm*** premaxilla ***po*** postorbital ***pof*** postorbital fossa ***popr*** paroccipital process ***q*** quadrate ***qj*** quadratojugal ***ri*** ridge ***sa*** surangular ***sq*** squamosal.

MPEF-PV 3211, partial skull and postcranial skeleton lacking the forelimbs, hindlimbs, and caudal vertebrae (Figs 8, 81B).

##### Referred material.

MPEF-PV 1718, 1719, 1786, 3810, and 3811, isolated teeth.

##### Type locality.

Queso Rallado, 2.3 km west of Cerro Cóndor, Chubut Province, Argentina (Pol et al. 2011).

##### Horizon.

Cañadón Asfalto Formation (Stipanicic et al. 1968; Rougier et al. 2007); Middle Jurassic, Aalenian-Bathonian, ca. 176-165 Ma (Gradstein and Ogg 2009; Cabaleri et al. 2010).

##### Revised diagnosis.

Heterodontosaurid ornithischian characterized by the following four autapomorphies: (1) external mandibular fenestra absent; (2) denticules on the margins of individual denticles; (3) mesially divergent basal denticle on mesial margin in some dentary crowns; (4) mesial denticulate margin approximately 60% the length of the distal margin.

##### Description.

The revised diagnosis above restricts cited features to those interpreted as potential autapomorphies for *Manidens condorensis*. Several features listed in the initial diagnosis (Pol et al. 2011: 370) have broader distributions and therefore were omitted. Besides the unusual features of the dentition, closure of the external mandibular fenestra is the only nondental autapomorphy listed, although more will surely be identified with additional preparation and description. The following descriptive comments are based on Pol et al. (2011) as well as stereophotographs of the holotypic specimen (courtesy of D. Pol).

##### Cranium.

Only a portion of theskull roof and braincase are preserved and figured (Pol et al. 2011). Judging from the length of the dentary, the snout is likely to be proportionately shorter than in *Heterodontosaurus* (Figs 8A, 59, 81B). An inset, arched diastema was probably present between the premaxilla and maxilla, given the presence of a dentary caniniform tooth (Figs 8A, 81B). The form of the premaxilla and predentary are currently unknown.

As noted by Pol et al. (2011), aspects of the cranium are reminiscent of derived features in *Tianyulong* and *Heterodontosaurus* such as the arched profile of the upper temporal bar and the spacious laterotemporal fossa (Fig. 81B). The form of the postorbital, jugal, quadratojugal and quadrate exhibit features present in *Heterodontosaurus*, most of which are poorly known in other heterodontosaurids. The lateral aspect of the postorbital is excavated by a fossa, a laterally projecting crest and posteroventrally directed flange are present on the jugal, an embayment ventral to the lower temporal bar results in a T-shaped quadratojugal, and the ventral articular surface of the quadrate condyles angles ventrolaterally. The shaft of the quadrate dorsal to the condyles appears to be considerably more robust than in *Heterodontosaurus* (Pol et al. 2011: fig. 1e).

The maxilla has a laterally protruding rim along the ventral margin of the antorbital fossa as in other heterodontosaurids. Pol et al. (2011: 371) suggested that an arched diastema may not have been present. The anterior margin of the maxilla, however, is not well preserved (Fig. 81B). The presence of a caniniform tooth in the dentary (Fig. 8A) strongly suggests that an arched diastema would have been present as in other heterodontosaurids.

The prominent laterally projecting jugal horn is very similar in form and location to that in *Heterodontosaurus* (Figs 59, 81B). The dorsoventrally compressed horn, which is located just ventral to the orbital margin, is connected by a ridge to the everted ventral rim of the antorbital fenestra. The jugal flange differs in its position from that in *Heterodontosaurus* (Figs 59, 81B). It projects posteriorly rather than posteroventrally. As a result, the flange has a more elevated position relative to the lower jaw than in *Heterodontosaurus*.

The postorbital has a particularly deep posterior ramus compared to *Heterodontosaurus* as seen in lateral view (Figs 59, 81B). In this regard, the postorbital is most similar to that in *Pegomastax africanus* gen. n. sp. n.asdescribed below. The postorbital fossa is well developed as in *Heterodontosaurus*.

##### Lower jaw.

The lower jaw is proportionately short with a deep dentary similar to that in *Pegomastax africanus* gen. n. sp. n.asdescribed below. A number of features in the lower jaw are present in several other heterodontosaurids including a deep buccal emargination, prominent coronoid process, an external mandibular fossa, and an enlarged anterior surangular foramen and associated neurovascular groove (Fig. 81B). The retroarticular process appears to be proportionately shorter than in *Heterodontosaurus*, and the external mandibular fenestra is closed (Figs 8A, 81B).

Pol et al. (2011) have depicted the proportions of the angular and surangular in lateral view in two ways, the former deeper as well as shallower than the latter (Figs 8A, 81A). The deeper angular (Fig. 8A) is their intended interpretation (D. Pol pers. comm.), which was recorded as a derived character in their matrix for *Manidens* and *Heterodontosaurus*. As noted above, however, the deep proportions of the angular in *Heterodontosaurus* (Fig. 58) are regarded here as erroneous (Fig. 59). In the reconstruction given here of *Manidens*, we have followed their drawing of the lower jaw, in which the angular is somewhat deeper than the surangular (Figs 8A, 81B).

The jaw joint is offset ventral to the maxillary tooth row as in *Heterodontosaurus* and probably also *Lycorhinus* (Figs 8A, 81B). Pol et al. (2011: 373) suggested that the articular cup for the quadrate condyles is “considerably longer anteroposteriorly”, allowing some fore-aft movement of the quadrate. This cannot be verified in currently available images. If that is an accurate assessment of the jaw joint, then it would differ from the tight fit of the quadrate condyles to the articular cotylus in *Heterodontosaurus* (Fig. 61C).

##### Dentition.

There appears to be approximately 11 teeth in maxillary and dentary tooth rows and a diastema between the caniniform and postcaniniform teeth, judging from the preserved portions of the tooth row in the right dentary (Fig. 81B). The relatively low tooth count, marked disparity in crown size along the tooth row, straight maxillary and dentary alveolar margins, and robustly proportioned dentary are comparable to the condition in *Pegomastax africanus* gen. n. sp. n.as described below.

The dentary crowns also resemble the new African species in the mesial bowing of the central axis of the crown. The mesial carina, in addition, is shorter than the distal carina in both species, although this asymmetry is more strongly expressed in *Manidens* (Pol et al. 2011).

##### Skull reconstruction.

The skull reconstruction presented here (Fig. 81B) differs in several regards from the preliminary reconstruction presented in Pol et al. (2011) (Fig. 81A). The most important differences involve the addition of an arched diastema to accommodate the dentary caniniform tooth, the alignment of maxillary teeth over the dentary cheek tooth row (the former was displaced anteriorly), the addition of braincase elements posteroventral to the quadrate head, and the lowering of the jaw joint relative to the tooth rows to match the geometry preserved in the right dentary (Fig. 8A).

##### Postcranial skeleton.

Portions of the axial column and pelvic girdle are preserved (Fig. 8B). The short trapezoidal centra and epipophyseal processes in mid cervical vertebrae (Pol et al. 2011: fig. 1d) are similar to that in *Heterodontosaurus* (Fig. 62) and suggest that the cervical series was arched as reconstructed (Pol et al. 2011: fig. 1). Mid and posterior dorsal vertebrae have neural spines that are longer anteroposteriorly than deep, which more closely resembles *Tianyulong* (Fig. 20) than *Heterodontosaurus* (Fig. 68).

The pelvic girdle exhibits a fully open acetabulum and a laterally prominent ischial peduncle on the ilium (Fig. 8B) similar to that in *Tianyulong*, the Kayenta heterodontosaurid, *Abrictosaurus*, *Heterodontosaurus*, and more advanced ornithischians. The pelvis is more primitive in two regards than some other heterodontosaurids. The iliac postacetabular process is deeper dorsoventrally than in *Abrictosaurus* and *Heterodontosaurus* (Figs 37, 68), and the postpubic process does not appear to be shortened as in *Tianyulong* (Fig. 30).

#### Genus Pegomastax gen. n.

urn:lsid:zoobank.org:act:239D2A52-1C29-4AD6-811D-3CCB7D81FEE2

http://species-id.net/wiki/Pegomastax

##### Derivation of name.

From the Greek *pegos* and *mastax*, meaning “strong jaw”.

##### Diagnosis.

Same as for only known species.

#### Pegomastax africanus sp. n.

urn:lsid:zoobank.org:act:11E67968-C4E9-40EA-B7F5-D05D6023DF5C

http://species-id.net/wiki/Pegomastax_africanus

Figs 5B 82 [Fig F83] [Fig F84] [Fig F85] [Fig F86] 87 Table 1 [Table T2] 3

##### Holotype.

SAM-PK-K10488, fragmentary skull preserving right and left dentaries and the predentary.

##### Type locality.

Voyizane (= Voisana), Transkei (Herschel) District, Cape Province, South Africa; S30°34', E27°25' (Crompton and Charig 1962; Kitching and Raath 1984) (Fig. 1B).

**Horizon**.Upper section of the Elliot Formation (= Red Beds); Lower Jurassic, Hettangian to Sinemurian, ca. 200-190 Ma (Crompton and Charig 1962; Santa Luca et al. 1976; Knoll 2005; Gradstein and Ogg 2009).

##### Derivation of name.

From “Latin *africanus*, meaning “pertaining to Africa”.

##### Diagnosis.

Heterodontosaurid ornithischian characterized by the following four autapomorphies: (1) proportionately deep predentary with a dorsal margin about 70% the anteroventral margin; (2) predentary dorsal margin angled anteroventrally at approximately 45°; (3) postcaniniform dentary crowns with an mesially-bowed primary ridge that angles from the apical denticle toward the mesial side of the crown base; (4) slightly concave denticulate crown margins to either side of a prominent apical denticle.

##### Description.

**Figure 82.**
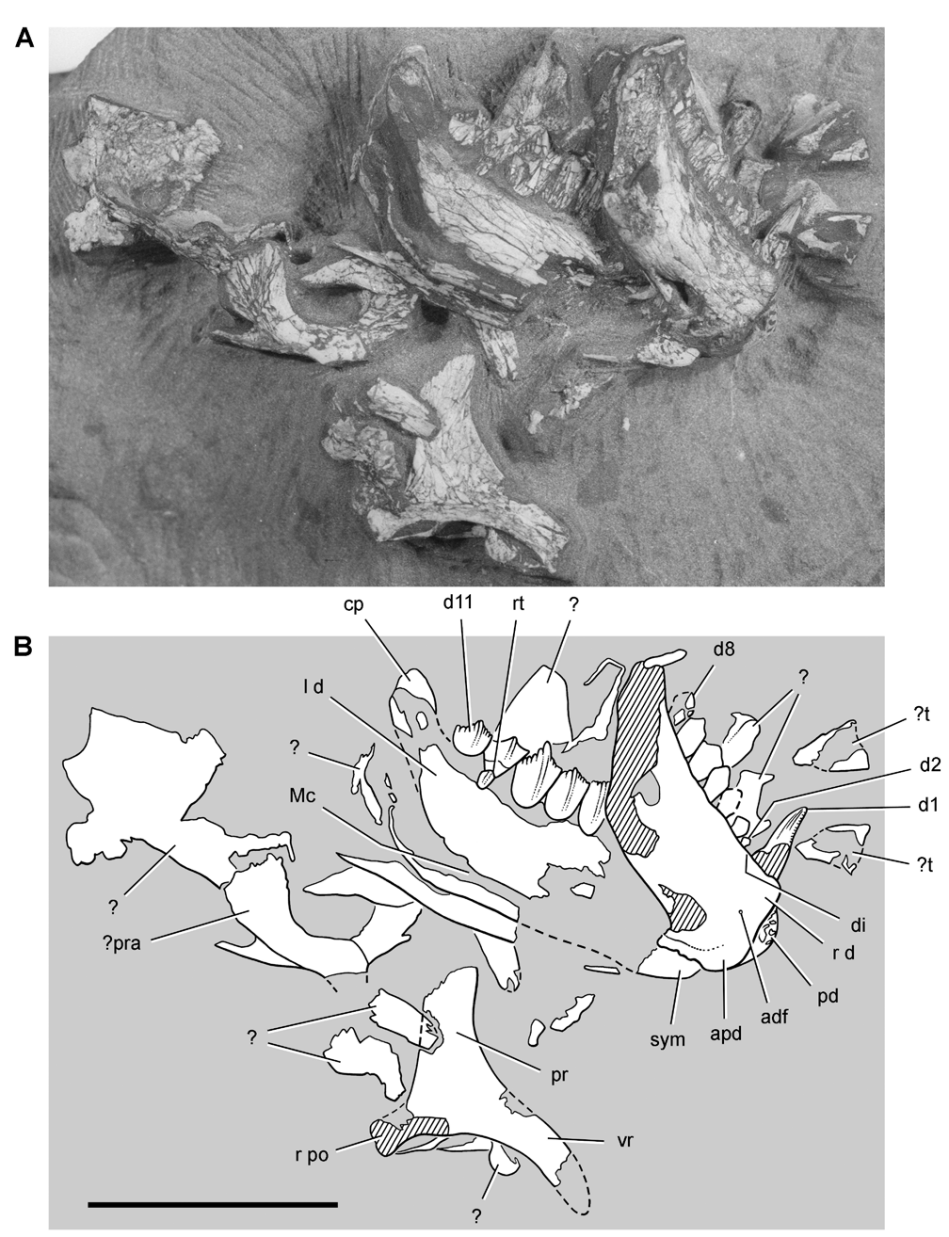
Partial skull of the heterodontosaurid *Pegomastax africanus* gen. n. sp. n. from the Lower Jurassic Upper Elliot Formation of South Africa. Partial skull (SAM-PK-K10488). Photograph (**A**) and line drawing (**B**) of the partial skull preserving the postorbital and anterior portion lower jaws. Hatching indicates broken bone; dashed lines indicate estimated edges; tone indicates matrix. Scale bar equals 2 cm. Abbreviations: ***adf*** anterior dentary foramen ***apd*** articular surface for the predentary ***cp*** coronoid process ***d*** dentary ***d1***, ***2***, ***8***, ***11*** dentary tooth 1, 2, 8, 11 ***di*** diastema ***l*** left ***Mc*** Meckel’s canal ***pd*** predentary ***po*** postorbital ***pr*** posterior ramus ***pra*** prearticular ***r*** right ***rt*** replacement tooth ***sym*** symphysis ***t*** tooth ***vr*** ventral ramus.

**Figure 83. F83:**
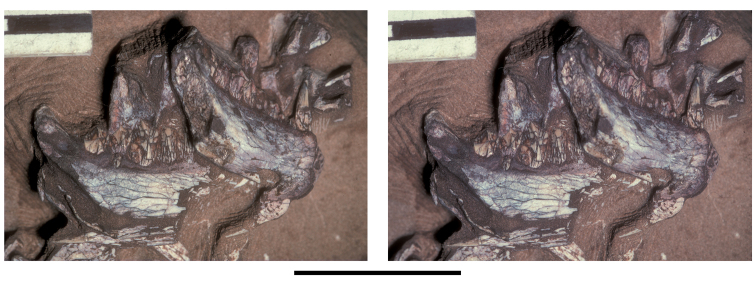
Lower jaws of the heterodontosaurid *Pegomastax africanus* gen. n. sp. n. from the Lower Jurassic Upper Elliot Formation of South Africa. Anterior portion of the lower jaws (SAM-PK-K10488). Stereopair of dentaries and predentary. Scale bar equals 2 cm.

**Figure 84. F84:**
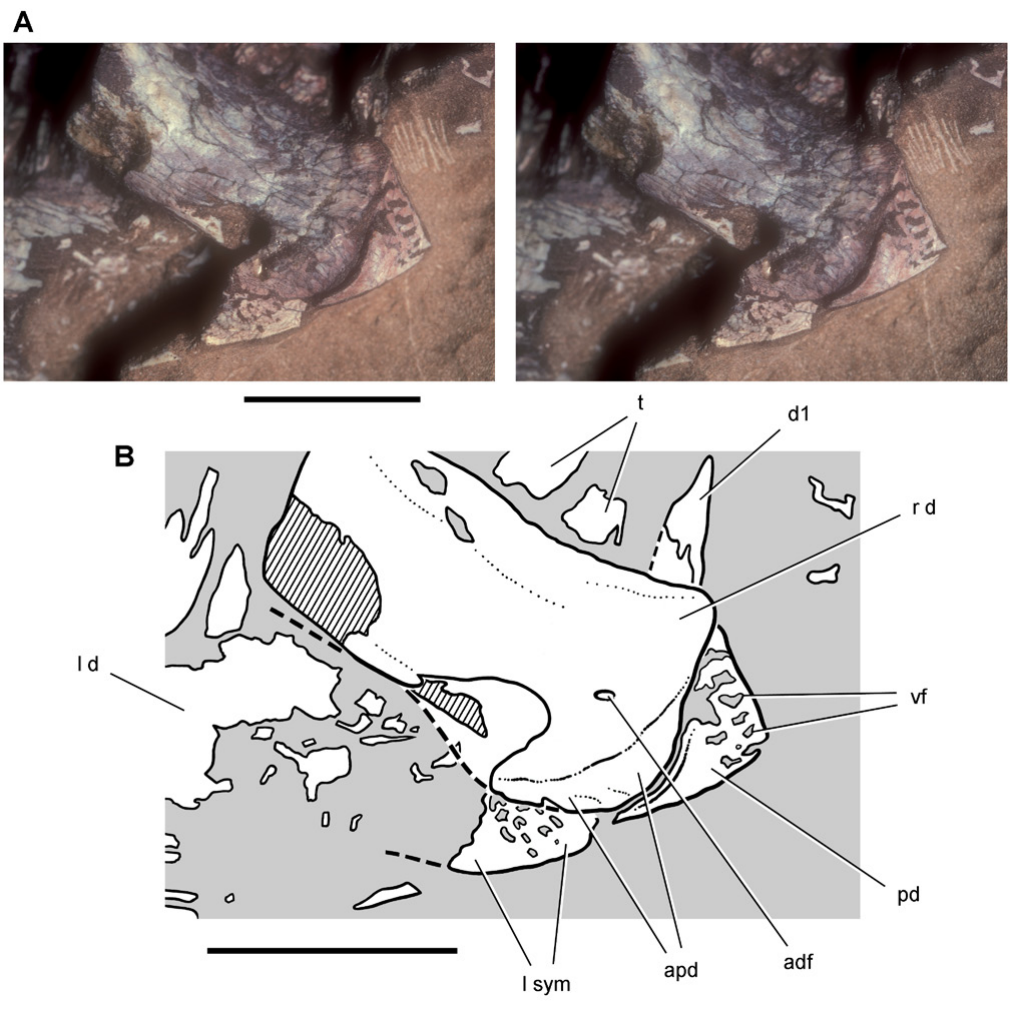
Lower jaw of the heterodontosaurid *Pegomastax africanus* gen. n. sp. n. from the Lower Jurassic Upper Elliot Formation of South Africa. Predentary and dentaries (SAM-PK-K10488). Stereopair (**A**) and line drawing (**B**) of the predentary and anterior portion of the dentaries in ventrolateral view. Hatching indicates broken bone; dashed lines indicate estimated edges; grey tone indicates matrix. Scale bars equal 1 cm. Abbreviations: ***adf*** anterior dentary foramen ***apd*** articular surface for the predentary ***d*** dentary ***d1*** dentary tooth 1 ***l*** left ***pd*** predentary ***r*** right ***sym*** symphysis ***t*** tooth ***vf*** vascular foramen.

The holotypic represents a small heterodontosaurid that is preserved on a small block of sandstone matrix (Figs 5B, 82) collected at Voyizane (Fig. 1B) near sites that yielded the best-preserved remains of *Heterodontosaurus tucki* (SAM-PK-K337, -K1332). Only portions of the skull are preserved, which includes the right postorbital, both dentaries, and the predentary. Further preparation or computed-tomographic imaging may allow identification of other elements. The anterior end of the lower jaws is preserved close to their natural articulation. The left dentary has slid slightly ventral to the right dentary, exposing a portion of its symphyseal articular surface (Figs 82–84). Bones that should be present medial to the dentaries, such as the coronoid and splenial, have moved from their natural articulation if they are preserved.

##### Predentary.

The predentary is a wedge-shaped bone that is very short anteroposteriorly (Figs 84, 87). Its dorsal margin is only about 70% of the dorsoventral depth of the bone. In *Heterodontosaurus*, in contrast, the dorsal margin of the predentary slightly exceeds its dorsoventral depth (Figs 59-61). Matrix currently fills several vascular foramina and associated grooves below the sharp dorsal margin of the predentary, which would have supported a keratinous bill (Fig. 84). The row of foramina parallels the sharp dorsal edge of the bone, which shows no evidence of breakage despite its strong anteroventral inclination at about 45°. The inclination of the dorsal margin of the predentary appears natural, as the bone is preserved in articulation with its dorsal edge butted against the right dentary. The posterior margin of the predentary is sinuous, tapering ventrally to a point among the midline (Fig. 84). As in *Heterodontosaurus*, there is no development of a discrete lateral predentary process, and the posterior margin is positioned against the upper part of a saddle-shaped articular surface along the anterior end of the dentary.

##### Dentary.

The robustly proportioned right and left dentaries are exposed in lateral and medial views, respectively (Figs 83, 84, 87). The dentary tooth row has a length of approximately 27 mm, as measured from the anterior end of the left dentary to the posterior edge of its posteriormost dentary crown (Fig. 82). The depth of the dentary ramus at mid-length is approximately 9 mm, or about one-third of the total length of the tooth row. In *Heterodontosaurus*, in contrast, the dentary tooth row has a length of 42 mm and a depth at mid-length of 10 mm (SAM-PK-K1332), for relative depth less than one-quarter the length of the toothed portion of the bone. The dentary in *Manidens* (Fig. 81B) appears to be slightly more robust than that in *Pegomastax* by this proportion.

The lateral aspect of the right dentary preserves a deep buccal emargination including several matrix-filled foramina. A small anterior dentary foramen is present near the predentary and is not associated with an impressed vessel tract as in *Echinodon* and *Fruitadens* (Figs 9A, 16). The blunt anterior end of the dentary has a smooth, saddle-shaped articular surface for the predentary, which is narrower dorsally and broadest ventral to the midline. Although fitted to the sinuous posterior margin of the predentary, the articular surface is significantly deeper than the predentary, similar to the condition in *Heterodontosaurus* (Fig. 39). The dorsal end of the saddle-shaped articular surface extends under the predentary near the prominent anterodorsal corner of the dentary. The ventral end of the trough-shaped articulation is situated below the ventral margin of the dentary ramus. The anterior end of the dentary is expanded dorsoventrally relative to the main body of the dentary ramus (Fig. 87), as in *Abrictosaurus* and *Heterodontosaurus*.

The flat ventral portion of the dentary symphysis is exposed in medial view of the left dentary (Fig. 84). Meckel’s canal is exposed as a narrow trough located just above the ventral margin of the left dentary ramus as in other heterodontosaurids (Fig. 82). A robust tongue-shaped coronoid process rises at about 45° from the posterior end of the tooth row. Replacement foramina do not appear to be present near the alveolar margin of the left dentary, which is well exposed in medial view.

##### Postorbital.

The right postorbital is preserved in medial view (Fig. 82). The most notable feature is the deep proportions of the posterior ramus, which resembles the condition in *Manidens* (Fig. 81) in contrast to the more slender ramus in *Heterodontosaurus* (Fig. 59). The arched ventral ramus forms the posterior portion of the orbital margin.

##### Dentary teeth.

There are probably 11 dentary teeth. Most of the right tooth row is preserved in lateral view from the caniniform tooth (d1) to d8 (Fig. 82). The left dentary preserves the coronoid process and five relatively large crowns at the posterior end of the tooth row (d7-11). The longer length of the dentigerous portion of the left dentary, from its anterior end to the posteriormost crown, suggests that the posterior portion of the right dentary and several posterior teeth have been sheared away. This is confirmed on the breakage surface of the right dentary, where the roots of three posterior teeth are seen in the cross-section (Figs 82, 83). The right dentary, thus, suggests there are at least 11 teeth in the dentary, and that the left dentary crowns represent d7-11 (Fig. 86). The anterior portion of the left dentary tooth row may be preserved but obscured by the right dentary.

**Figure 85. F85:**
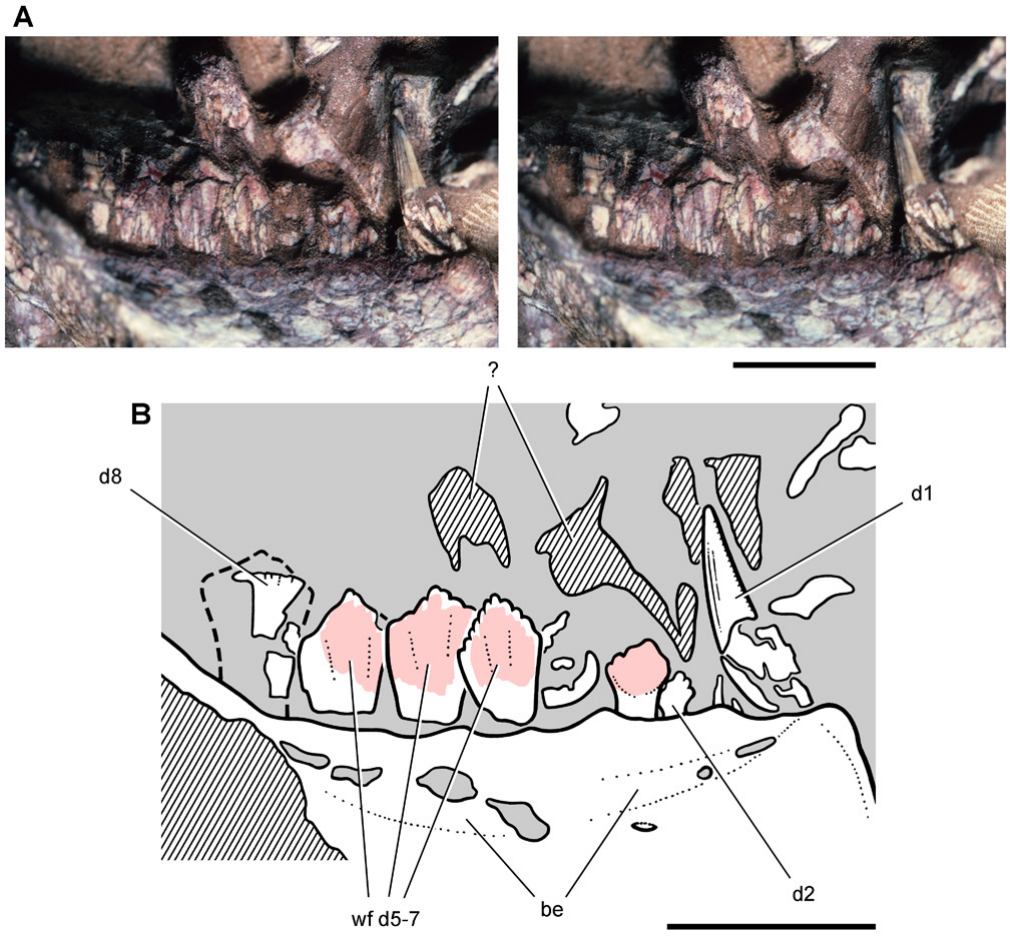
Dentary teeth of the heterodontosaurid *Pegomastax africanus* gen. n. sp. n. from the Lower Jurassic Upper Elliot Formation of South Africa. Dentary tooth row (SAM-PK-K10488). Stereopair (**A**) and line drawing (**B**) of left dentary teeth 1-8 in lateral view. Hatching indicates broken bone; dashed lines indicate estimated edges; grey tone indicates matrix; pink tone indicates wear facets. Scale bars equal 5 mm. Abbreviations: ***be*** buccal emargination ***d1***, ***2***, ***5–8*** dentary teeth 1, 2 5–8 ***wf*** wear facets.

The caniniform tooth (d1) has straight mesial and distal carinae, the former with serrations (Fig. 85). The base of the crown is crushed and the upper one-half slightly twisted. The distal carina was probably straight like the mesial carina. Unlike the mesial carina, the distal carina does not appear to have had serrations. The lack of any distal recurvature in the crown of the dentary caniniform tooth contrasts with the condition in most heterodontosaurids including *Lycorhinus, Abrictosaurus* and *Heterodontosaurus*.

There is a significant diastema in the right dentary tooth row between the caniniform tooth and d2, the relatively small first postcaniniform crown (Fig. 85, 87). Right d2-4 show an increase in crown size, and crown size seems to culminate in d6 (Fig. 85). The crowns of d2 and d3 are diamond shaped with a basal constriction under the crown. Like all dentary crowns, they are asymmetrical, with the distal apical margin slightly longer and extending farther down the crown than the mesial apical margin. A few denticles are preserved on their margins but these are not well preserved. The crown of d5 is diamond-shaped with six or seven denticles preserved distal to the apical denticle. Like more distal crowns, most of its lingual face is worn away by a planar wear facet, which has yet to obliterate the distal denticles. Small areas of the crown surface of d3 and d4 have a glassy appearance, suggesting that a thin layer of enamel may have been retained on the labial side of the dentary crowns.

**Figure 86. F86:**
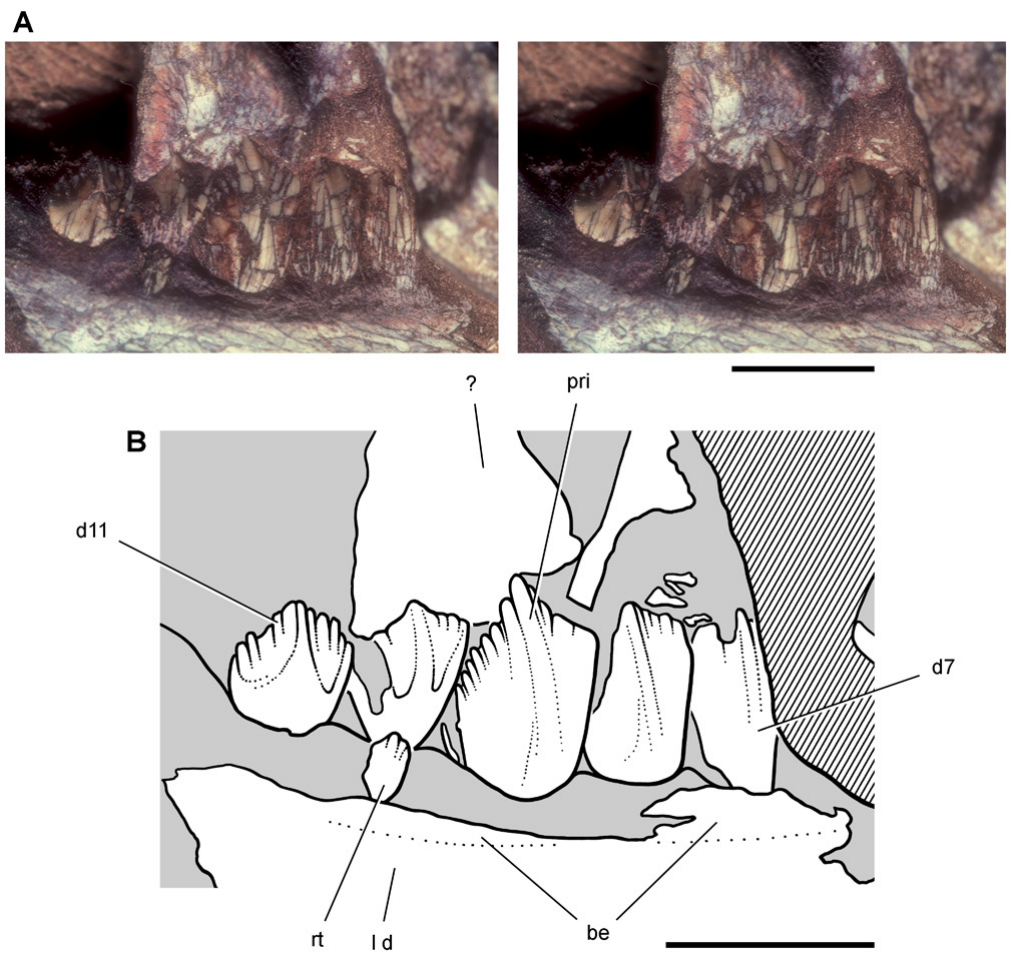
Dentary teeth of the heterodontosaurid *Pegomastax africanus* gen. n. sp. n. from the Lower Jurassic Upper Elliot Formation of South Africa. Posterior dentary teeth (SAM-PK-K10488). Stereopair (**A**) and line drawing (**B**) of left dentary teeth 7-11 in medial view. Hatching indicates broken bone; tone indicates matrix. Scale bars equal 5 mm. Abbreviations: ***be*** buccal emargination ***d*** dentary ***d7***, ***11*** dentary tooth 7, 11 ***l*** left ***pri*** primary ridge ***rt*** replacement tooth.

Right d6 and d7 have a dorsal crown profile like all more distal crowns. Concave apical margins are present on either side of the apical denticle, the mesial apical margin of which is shorter and more apically positioned than the distal apical margin (Fig. 86). Adjacent worn crowns create a scalloped leading edge, as preserved in the middle of the right tooth row (Fig. 85).

The medial crowns surface is most completely preserved in distal teeth (d7-11) on the left side (Fig. 86). All but the distalmost corner of the crown of d9 is exposed, which has a height 150% of its maximum width. The crown expands above its root, although the crown-root junction in this tooth is not exposed. The margins of the basal portion of the crown are not raised, and the first denticle on the apical margin on either side is not enlarged or divergent as in *Manidens*. *Pegomastax* has a well-developed primary ridge that originates just above the crown base and gains prominence as it arches from the mesial to the center of the crown before joining the apical denticle distal to the central crown axis (Fig. 86). The terminal end of the ridge is wide enough that it incorporates small accessory denticles, one to each side of the apical denticle, an unusual feature that may only be expressed in the largest crowns. There are about eight denticles to each side of the apical triumvirate. The denticles appear to be slightly larger on the distal apical margin, which is slightly longer and more steeply inclined from the horizontal than the mesial apical margin. The distalmost dentary tooth (d11) has lower proportions, as is typical for the last crown of the tooth row in heterodontosaurids. Its crown is only slightly taller than it is wide (Fig. 86).

The roots are only partially exposed in cross-section of the right lower jaw. The most complete of these, which probably belongs to d10, has a relatively large diameter and central lumen. The roots in *Pegomastax* thus may have been swollen as in many other heterodontosaurids.

**Figure 87.**
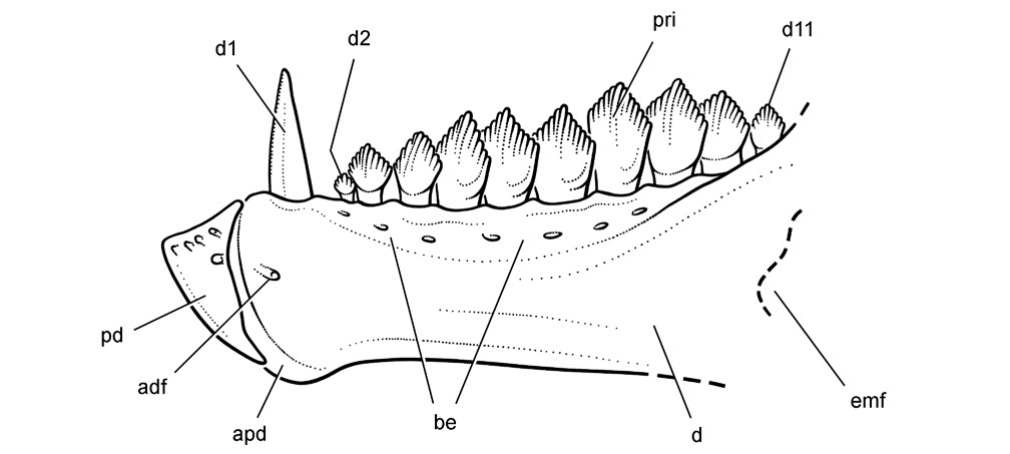
Lower jaw of the heterodontosaurid *Pegomastax africanus* gen. n. sp. n. from the Lower Jurassic Upper Elliot Formation of South Africa. Reconstruction of the predentary and dentary in lateral view (based on SAM-PK-K10488). Dashed lines indicate estimated edges. Abbreviations: ***adf*** anterior dentary foramen ***apd*** articular surface for the predentary ***be*** buccal emargination ***d*** dentary ***d1***, ***2***, ***11*** dentary teeth 1, 2, 11 ***emf*** external mandibular fenestra ***pd*** predentary ***pri*** primary ridge.

The crowns of right d5-8 and left d6-9 have an imbricate arrangement relative to one another (Figs 85-87). This feature is common in ornithischian dentitions and positions the mesial edge of each crown lingual to the distal edge of the next most mesial crown. The distal two-thirds of the dentary tooth row in *Pegomastax*, thus, exhibits this minor overlap between adjacent crowns edges. There does not appear to be a mesial fossa at the base of the crown to accommodate the edge of an adjacent crown as in *Manidens* (Pol et al. 2011). This fossa in *Manidens*, however, is only well exposed in mesial view, a view not yet available in *Pegomastax*.

All postcaniniform teeth in the right dentary are worn to varying degrees except perhaps the small crowns of d2-4 (Fig. 85). Sustained wear has obliterated all denticles on d6 and d7 in the right dentary. On the left side, d10 has reached this stage of wear and has no denticles; right d7 and d8 show less wear, and d9 and d11 show little or no wear. Their broad, nearly planar wear surfaces join to form a nearly continuous shearing surface, which is set at a very low-angle to the vertical crown axis.

Differential wear along the tooth row strongly suggests cyclic tooth replacement, despite the general alignment of wear surfaces and the absence of replacement foramina. Direct evidence of replacement is present in the left dentary tooth row. A small replacement crown is emerging at the base of the heavily worn crown of d10 (Fig. 86).

##### Jaw reconstruction.

The reconstruction of the dentary of *Pegomastax* is based on the holotype and only known specimen (Fig. 87). The labial surfaces of available dentary crowns are worn or broken, obscuring details of ornamentation. In the skull reconstruction, the ornamentation of labial crown surfaces was tentatively based on the exposed lingual crown surfaces in the left dentary (Fig. 86). Computed tomographic imaging might reveal evidence of the form of the lateral crown surface in a relatively unworn crown such as left d9 (Fig. 86).

The most unusual feature of the preserved portion of the skull is the shape of the predentary. Despite many similarities to *Heterodontosaurus* in the differentiated dentition and the saddle-shaped form of the predentary-dentary articular surface, the unusually deep, parrot-like shape of the predentary suggests that its function in cropping vegetation may have become specialized in various heterodontosaurid species, similar to the way bill shape varies among birds. *Pegomastax*, furthermore, provides additional evidence in favor of a mobile predentary-dentary joint, given the disparity in size between the predentary and the longer articular surface on the dentaries.

## Discussion

### Taxonomic resolution

Two major concerns in heterodontosaurid systematics include the tentative heterodontosaurid status of *Echinodon becklesii* (Sereno 1997; Norman and Barrett 2002) and unresolved issues surrounding the validity of *Lycorhinus angustidens* and allied specimens (Charig and Crompton 1974; Thulborn 1974; Hopson 1975). Both of these issues are resolved below with greater confidence in light of the additional anatomical information presented above.

***Echinodon*, a heterodontosaurid.**In traditional classifications of the early and mid twentieth century, *Echinodon* was referred provisionally to the Fabrosauridae, Hypsilophodontidae, or Stegosauria (Norman and Barrett 2002). Galton (1978) was first to mention similarities between *Echinodon* and heterodontosaurids, citing as many as 11 characters (Galton 1978: 154). The list was not compiled under a cladistic paradigm, and so many of the features are symplesiomorphies shared with many other ornithischians as noted by Norman and Barrett (2002). In the context of other basal ornithischians, the only feature that can be construed as a shared derived heterodontosaurid character is the “reduced number of premaxillary teeth”. In the absence of obvious synapomorphies, Galton (1986) and others (Coombs et al. 1990) subsequently allied *Echinodon* with armored (thyreophoran) ornithischians based on an erroneous association between *Echinodon* and small osteoderms from the same formation (Norman and Barrett 2002).

Sereno (1991, 1997) cited three synapomorphies linking *Echinodon* and heterodontosaurids, namely an arched premaxilla-maxilla diastema, a wedge-shaped predentary, and a dentary caniniform tooth. In their review of *Echinodon*, Norman and Barrett (2002) cast doubt on two of these synapomorphies, claiming that only the wedge-shaped predentary could be verified among preserved specimens. They noted two additional features in support a heterodontosaurid interpretation, namely the restriction of the denticles to the distal portion of the crown and the absence of replacement foramina.

It is possible that (1) reduction of the premaxillary tooth row to three teeth (Galton 1978) is a derived heterodontosaurid feature present in *Echinodon*, despite parallel reduction in several ornithischian clades. Most potential saurischian outgroups have at least four premaxillary teeth, and many other basal ornithischians have six or more. Three other synapomorphies visible in the available material of *Echinodon* include (2) an arched premaxilla-maxilla diastema, (3) a wedge-shaped predentary, and (4) a dentary caniniform tooth as suggested by Sereno (1997) and depicted in reconstruction (Fig. 19B). The latter two are based on evidence from the dentaries, namely the presence of an enlarged alveolus for a caniniform tooth at the mesial end of the dentary tooth row and the presence of a rounded articular surface, rather than inset median and parasagittal facets, for contact with the predentary. Finally (5) the absence of replacement foramina in either the maxilla or dentary is a potential synapomorphy of heterodontosaurids (Norman and Barrett 2002; *contra* Galton 1978), although the plesiomorphic condition among potential saurischian outgroups is ambiguous (Martinez et al. 2011).

Other heterodontosaurid synapomorphies now apparent in the original material of *Echinodon* include (6) a proportionately short dentary (height at mid-length 25% or more of length from anterior end to the notch of the external mandibular fenestra), (7) Meckel’s canal developed as a narrow trough immediately adjacent to the ventral margin of the dentary, (8) premaxillary teeth that increase in height posteriorly, and (9) a rudimentary precaniniform dentary tooth that is lost in some more advanced heterodontosaurids. The last synapomorphy in *Echinodon* is based on the presence of a tiny alveolus (Figs 16C, 19) that matches the very small first dentary tooth in *Abrictosaurus* and *Lycorhinus* (Figs 35, 78, 80). The lobe-shaped, strongly inclined coronoid process on the dentary in *Echinodon* is also similar to other heterodontosaurids and better developed than in basal ornithischians such as *Lesothosaurus* and *Scutellosaurus*. Enhancement of this process, however, also occurs in neornithischians in general, and so it is not included here as a heterodontosaurid synapomorphy.

In sum, there is substantial character evidence, that *Echinodon* is a late surviving basal heterodontosaurid (*contra* Norman and Benton 2002). The recent phylogenetic analysis of Butler et al. (2008: 18) was unable to link *Echinodon* with other heterodontosaurids, because little of the character information cited above was included in the analysis.

***Lycorhinus*, a valid taxo*n*.** The genus *Lycorhinus* and its type species *Lycorhinus angustidens* have been subject to conflicting taxonomic opinions ever since the description of NHMUK RU A100, a disarticulated partial skull (Thulborn 1970) (Table 2). The holotypic left dentary of *Lycorhinus angustidens*, as recounted in the introduction of this study, is now preserved for the most part as a natural impression, from which was derived a silicone cast (Fig. 3). The more complete specimen NHMUK RU A100, here referred to *Lycorhinus angustidens*, was found in the vicinity of the type locality. Thulborn (1970) regarded the genus *Heterodontosaurus* as a junior synonym of *Lycorhinus*, a generic synonomy without obvious justification that has not been accepted by other taxonomists.

Galton (1973a: 71) stated that there is no basis for Thulborn’s reference of NHMUK RU A100 to *Lycorhinus angustidens*, and he placed the new material in *Heterodontosaurus* sp. Charig and Crompton (1974), on the other hand, argued that *Lycorhinus angustidens*, specimen NHMUK RU A100, and *Heterodontosaurus tucki* are all distinct taxa probably at the generic level, although they also stated that *Lycorhinus* and *Lycorhinus angustidens* should be regarded as *nomina dubia* (Table 2). They suggested specimen NHMUK RU A100 might represent two individuals, because the crowns in the maxilla were worn and could not be reasonably paired with unworn crowns in a dentary.

Thulborn (1974) described a new species, *Lycorhinus consors*, based on a partial skeleton (NHMUK RU B54), and Gow (1975) established the new genus and species *Lanasaurus scalpridens* based on an isolated left maxilla. Gow (1975) regarded *Lanasaurus scalpridens* as distinct from NHMUK RU A100 but possibly conspecific with *Lycorhinus consors* (Table 2).

Hopson (1975) entered the fray as arbiter, reexamining the holotypic dentary of *Lycorhinus angustidens* andconcludingthat both *Lycorhinus angustidens* and *Heterodontosaurus tucki* are generically distinct. He also argued that these two heterodontosaurids are generically distinct from Thulborn’s species *Lycorhinus consors* (NHMUK RU B54) and specimen NHMUK RU A100, placing the latter two specimens in a new genus as *Abrictosaurus consors* (Table 2). Weishampel (1984) and subsequent taxonomic compilations (Norman et al. 2004) accepted Hopson’s assignment of NHMUK RU A100 to *Abrictosaurus consors *), whereas Gow (1990) and this review suggest that NHMUK RU A100 should be assigned to *Lycorhinus angustidens* (Table 2). Gow (1990) also described an additional isolated maxilla, referring it and the holotypic maxilla of *Lanasaurus scalpridens* to *Lycorhinus angustidens*. The status of *Lanasaurus scalpridens* as a junior synonym of *Lycorhinus angustidens* has been accepted by later authors (Norman et al. 2004; this study), although recently Porro et al. (2010) and Norman et al. (2011) provisionally retained *Lanasaurus scalpridens* as a distinct genus and species.

Taxonomic controversy, in sum, involves the validity of the genus and species *Lycorhinus angustidens*, the status of NHMUK RU A100 as a single individual, and the proper referral of specimen NHMUK RU A100 and specimens referred to *Lanasaurus scalpridens* (Table 2). *Lycorhinus angustidens*, as outlined above, has two diagnostic features, a prominent distal marginal ridge and medial curvature of maxillary and dentary tooth rows (Fig. 3). Yet, this same pair of features was used by Norman et al. (2011: 239) to justify provisional retention of *Lanasaurus scalpridens*.Following Gow (1990), *Lanasaurus scalpridens* is here regarded as a subjective junior synonym of *Lycorhinus angustidens* (Table 2).

The reidentification of several of the bones of specimen NHMUK RU A100 (Figs 4, 73, 74) has removed reasonable doubt that it represents a single individual. The paired dentaries are of similar size; the premaxilla and maxilla are compatible; upper and lower cheek teeth are similar in shape and ornamentation; and both upper and lower cheek teeth exhibit high-angle wear facets.

Maxillary and dentary crowns inNHMUK RU A100 and *Abrictosaurus consors* (NHMUK RU B54) differ markedly in crown height and shape, the former diamond-shaped with a prominent cingulum (Figs 77C, 78) and the latter with taller proportions (Figs 32, 33). Hopson’s (1975) referral of NHMUK RU A100 to *Abrictosaurus consors* did not cite derived similarities, and none is listed in his diagnosis of *Abrictosaurus consors*. Comparisons between NHMUK RU A100 and the holotype of *Lycorhinus angustidens* (SAM-PK-K3606) are also limited. The dentary and its teeth provide the only overlap between these specimens, which are currently available only in medial view in the former and lateral view in the latter (as a silicone cast; Fig. 3D). The initial illustration of the medial aspect of the holotype of *Lycorhinus angustidens* by Haughton (1924), nonetheless, clearly shows several crowns with a prominent distal marginal ridge (Fig. 3B), and the natural cast exhibits some medial curvature of the dentary tooth row (Fig. 3D). The specimens are also similar in the relatively large size of the caniniform tooth and the relatively uniform size of the postcaniniform teeth. This suite of features warrants referral of NHMUK RU A100 to *Lycorhinus angustidens* (Tables 1, 2).

Several misleading statements have been made regarding the teeth of NHMUK RU A100. Thulborn (1970) stated that serrations occur only on the anterior margin of the caniniform tooth in NHMUK RU A100 and *Lycorhinus angustidens*, whereas Hopson (1975) reported the presence of serrations on both margins in the latter. The posterior margin of the caniniform tooth in NHMUK RU A100, however, is poorly exposed and could well have been serrated. Hopson (1975) cited other differences between NHMUK RU A100 and *Lycorhinus angustidens* (a more compact arrangement of cheek teeth, shorter crown proportions, a narrower basal cingulum, and more slender roots), but these are difficult to discern.

### Body size and proportions

**Body size.** *Echinodon* is one of the smallest non-avian dinosaurs on record. Although known almost entirely from isolated jaw bones, these can be used to estimate skull length, assuming the available dentaries are near adult size and the dentary composes about 45% of skull length as in *Heterodontosaurus* (Table 3). Besides isolated teeth, the hypodigm of *Echinodon* consists of eight specimens that were given nine specimen numbers; the lectotypic specimens (NHMUK 48209, 48210) belong to a partial skull (Fig. 13). All of these jaws are very similar in size, suggesting that they may pertain to adults or at least subadults. The length of the dentary in *Echinodon* is approximately 28 mm, as measured from the anterior tip of the dentary to the end of the coronoid process (NHMUK 48215). A skull length estimate based on this dentary is 62 mm, which is subequal to the estimated skull length for *Tianyulong* (average 66 mm) and juvenile individuals of *Fruitadens* (60 mm) and shorter than adult individuals of *Fruitadens* (75 mm) (Table 3). The Kayenta heterodontosaurid is smaller, but the sole specimen currently known is immature (MCZ 9092).

Two of the four specimens of *Fruitadens* are regarded as adult (LACM 115727, 115747) and two as subadults or juveniles (LACM 120478, 128258) based on their relative size and analysis of histological sections of large and small individuals (Butler et al. 2010). Four lines of arrested growth were identified in a section of the femoral shaft of the holotype (LACM 115747), although only three were labeled in the figure provided (Butler et al. 2010: fig. 3f). The holotype of *Fruitadens* was argued to be a young adult in its fifth year of growth (Butler et al. 2010), a reasonable conclusion based on the histological evidence. A subadult specimen (LACM 120478) is approximately 80% the size of the holotype, as best as can be estimated from overlapping tibial measurements (Table 3). The length of the dentary in another subadult specimen (LACM 128258) provides the basis for its skull length estimate of approximately 60 mm. That estimate yields an adult skull length estimate of approximately 75 mm, which places *Fruitadens* in the subgroup of small-bodied heterodontosaurids, although possibly somewhat larger than *Echinodon* and *Tianyulong* (Table 3).

*Manidens*, *Pegomastax*, and *Abrictosaurus* are known principally from individual specimens estimated to have comparable skull length (~ 70-80 mm). This overlaps the skull length range estimated for *Tianyulong* and *Fruitadens* (~ 65-75) and cannot be considered significant with such low specimen numbers (Table 3). *Heterodontosaurus* and *Lycorhinus* seem to be distinctly larger than other heterodontosaurids, their skull length estimates ranging from approximately 115 to 200 mm. The best known skulls of *Heterodontosaurus* are at the low end of the range with a large referred skull at the high end (NM QR 1788; Porro et al. 2010). *Lycorhinus* lies between these endpoints with a skull length estimate of approximately 145 mm (Table 3).

**Skeletal proportions.** *Tianyulong* has unusual skeletal proportions compared to *Heterodontosaurus* (Figs 30, 72; Tables 3-9). The skull is relatively larger and the hindlimbs are relatively longer, whereas the neck, trunk and forelimbs are relatively shorter. Compared to the Eichstätt specimen of *Archaeopteryx*, an individual with an identical femoral length (Wellnhofer 1993), the skull of *Tianyulong* is 25% longer (Table 5). The tibiofemoral ratio of the Eichstätt specimen of *Archaeopteryx* is high (140%), although that in *Tianyulong* is slightly higher (143%). The humerus in *Tianyulong*, in contrast, is only approximately 48% the length of that in *Archaeopteryx*.

These unusual proportions give *Tianyulong* the appearance of a large-headed, short-armed, long-legged dwarf (Fig. 30). This has not been noticed previously, because the initial report did not include a skeletal reconstruction (Zheng et al. 2009). *Tianyulong* had relatively short trunk proportions, judging from the distance between the skull and the pelvic girdle in the holotype (Zheng et al. 2009: fig. 1a). The anterior cervical vertebrae were lost on an adjacent slab that was not recovered. Centrum length in the posterior cervical vertebrae (probably C5-9) is uniform (5 mm), suggesting that *Tianyulong* does not have longer mid cervical centra as are present in *Heterodontosaurus* (Fig. 72). *Tianyulong* is reconstructed here with 9 cervical and 12 dorsal vertebrae, the precise count subject to confirmation when available specimens are more completely exposed.

Compared to *Heterodontosaurus*, the skull of *Tianyulong* is proportionately long compared to femoral length (Table 3). The forelimb and manual digit III, in contrast, are reduced relative to the hindlimb and manual digit II, respectively, compared to similar measures in *Heterodontosaurus*. Although relatively long in *Heterodontosaurus*, the tail appears to be relatively even longer in *Tianyulong*, as mid caudal centra are longer relative to proximal caudal centra than in *Heterodontosaurus*.

*Fruitadens* provides some evidence to estimate relative lengths of skull, femur and tibia, and the ratios involving these three measurements are closer to those in *Heterodontosaurus* than *Tianyulong* (Table 3). Likewise, skeletal proportions for *Abrictosaurus* closely match those in *Heterodontosaurus*, the tibiofemoral ratio, for example, very close to 130% (Table 3). The available skeletal evidence thus suggests that *Tianyulong* has unusual skeletal proportions compared to several other heterodontosaurids.

### Tooth replacement

**Tooth eruption.** Thulborn’s (1978) “aestivation” hypothesis outlined a life history scenario for heterodontosaurids in which tooth replacement occurred during a seasonal dry season when vegetation was scarce. During the remainder of the year, in this view, tooth eruption was suppressed, allowing newly erupted crowns in cheek tooth rows to develop contiguous wear facets as an integral functional unit. Thulborn forwarded the hypothesis that most heterodontosaurids had a nearly continuous wear surface along each cheek tooth row, which slid fore and aft in a propalinal masticatory cycle.

There is extensive evidence, however, for tooth replacement in all known heterodontosaurid species. The evidence consists of direct observation of erupting crowns visible near the alveolar margin or within tooth crypts, as shown in computed tomographic scans (Figs 9B, 14, 23, 33, 48, 44C) and differential wear in fully erupted functional crowns of a single cheek tooth row (Figs 3C, 55). Secondly, heterodontosaurid wear facets exhibit a variety of orientations and only approximate a continuous wear surface in any given cheek tooth row, even in the most derived species (Figs 41, 42, 55). Finally, several lines of evidence discussed by Hopson (1980) and further elaborated below strongly suggest that jaw movement was not propalinal but rather arcilineal (vertical) with a small amount of transverse translation and long-axis rotation.

Active tooth replacement is readily observed in all heterodontosaurids with simple subtriangular crowns, including *Echinodon* (Figs 13, 14, 18), *Fruitadens* (Butler et al. 2010), *Tianyulong* (Figs 22, 23), and the Kayenta heterodontosaurid (Fig. 9B). Erupting crowns in these taxa are intermingled with more mature crowns, a pattern of tooth eruption grossly similar to that in other basal ornithischians such as *Lesothosaurus* (Sereno 1991).

Tooth replacement also occurs in heterodontosaurids with deeper crown proportions, such as *Lycorhinus* (Gow 1990), *Abrictosaurus* (Fig. 33), and *Pegomastax*. (Fig. 86). At present we have no evidence of active tooth replacement in the holotypic and only known specimen of *Manidens* (Pol et al. 2011), although a worn crown pertaining to *Manidens* has been recovered from the formation (Pol pers. comm.). A juvenile skull of *Heterodontosaurus* (e.g., AMNH 24000) measuring approximately 50% of adult length shows active, staggered tooth replacement in upper and lower cheek tooth rows (Figs 41, 45–52). Tooth replacement in a second similar-sized subadult skull of *Heterodontosaurus* (SAM-PK-K10487) is not apparent in computed tomographic scans, although image resolution may not be sufficient in this case (Butler et al. 2008: fig. 5).

Tooth eruption may have decreased or ceased entirely in mature specimens of *Heterodontosaurus* (SAM-PK-K337, -K1332), a diminution in replacement also recorded with age among extant reptiles (Robinson 1976; Erickson 1996). Although there is no evidence of erupting crowns in these specimens, high resolution computed tomographic scans have yet to be published to be confident tooth replacement is truly suppressed. The teeth composing each functional tooth row in SAM-PK-K1332, nevertheless, erupted at different times, as suggested by differential wear (Hopson 1975, 1980; Gow 1990; Fig. 55). Another maxillary fragment pertaining to an adult *Heterodontosaurus* subequal in size to the aforementioned specimens (SAM-PK-K1334) captures active tooth replacement at several positions (Norman et al. 2011: Figs 30-33).

Heterodontosaurids may have suppressed replacement of caniniform teeth in the premaxilla, maxilla and dentary, as shown by currently available specimens. The only specimens showing active eruption in these tooth positions are clearly subadult individuals (e.g., Kayenta heterodontosaurid) (Fig. 9B). Adult specimens, in contrast, show fully erupted caniniform teeth (e.g., *Echinodon*, *Fruitadens*, *Tianyulong*, *Lycorhinus*) in many cases with broken tips with edges polished by abrasive wear (*Heterodontosaurus*).

In sum, there is no evidence in favor of periodic complete replacement of the cheek teeth in heterodontosaurids (*contra* Thulborn 1978). Neither is there solid evidence for “episodic” rather than continuous tooth replacement in *Heterodontosaurus* during growth (*contra* Norman et al. 2011: 182). Staggered tooth replacement in cheek tooth rows is commonplace in heterodontosaurids and occurs in subadult *Heterodontosaurus* as in other ornithischians without interruption of masticatory function. Adult specimens of *Heterodontosaurus* with dentitions truncated by high-angle wear facets show either active tooth replacement (SAM-PK-K1334) or suppressed replacement as mature adults (SAM-PK-K337, -K1332). These mature adult skulls, nonetheless, have tooth rows characterized by differential wear indicative of diachronous eruption of their functional, and possibly final, dentition.

**Replacement foramina.** Replacement foramina are only rarely present and possibly transitory in heterodontosaurids. Replacement foramina have been described in one adult maxilla of *Heterodontosaurus* (Norman et al. 2011: fig. 32). In that specimen, a groove for the dental lamina links replacement foramina, through which tooth buds could pass into alveolar crypts as is thought to occur in other ornithischians and basal sauropodomorphs. The absence of replacement foramina in heterodontosaurids has added to previous doubts regarding tooth replacement within the group. The dental lamina in heterodontosaurids more generally must have resided within alveolar bone, because the vast majority of specimens do not exhibit a groove for the dental lamina or replacement foramina, even with extensive evidence of active tooth replacement.

**Tooth roots.** The roots of the cheek teeth in heterodontosaurids have closed tips in the sharpest computed tomographic images and natural cross sections (Fig. 42). The roots are held snugly within the alveolar crypt. Heterodontosaurid roots thus do not resemble the open-ended, ever-growing condition of high-crowned, or hypsodont, teeth in mammals. Norman et al. (2011: 1, 44, 49, 71) used the term “hypsodont” to describe “high wear adapted” teeth in *Heterodontosaurus*. This term is misleading in connection with heterodontosaurid teeth, which do not have comparably extreme crown proportions, enamel sequestered within the alveolus or post-eruption (continuous) growth (von Koenigswald 2011).

The tapered roots of cheek teeth in heterodontosaurids with simple subtriangular crowns, such as *Echinodon* (Figs 15–19), *Tianyulong* (Fig. 24) or *Fruitadens* (Fig. 9A) resemble those in other basal ornithischians such as *Lesothosaurus* (Sereno 1991) and even larger ornithischians such as *Ouranosaurus* (Figs 53B, 54A). In a typical ornithischian tooth, the root tapers in width distal to the crown, often terminating as a slender tube (Fig. 53D). The roots of cheek teeth in advanced heterodontosaurids such as *Lycorhinus* and *Heterodontosaurus*, in contrast, appear swollen in diameter (subequal to maximum crown width) and have blunt, rounded root tips (Figs 3C, 42). The cingulum is reduced and so there is only a gentle constriction under the crown. The root then expands slightly in girth before tapering bluntly near its end.

The pulp cavity collapses during growth, and is usually restricted to the root in a mature ornithischian tooth (Figs 53D, 54). In *Heterodontosaurus* and possibly other advanced heterodontosaurids, in contrast, the pulp cavity appears to be relatively larger and extends into the base of the crown. In broken teeth (Fig. 42) and computed tomographic cross sections (Fig. 50C, m10), the basal portion of the crown in noncaniniform maxillary and dentary teeth is hollowed by the pulp cavity. The capacious pulp cavity is present in subadult specimens approximately 50% adult body size (AMNH 24000) as well as mature adults, in which the pulp cavity is exposed in cross section on the worn crowns (Fig. 55). In *Heterodontosaurus*, thus, the pulp cavity is not limited to the root in fully erupted functioning teeth (*contra* Norman et al. 2011: 213). The biological meaning of the unusual root form that characterizes *Heterodontosaurus*, and possibly other heterodontosaurids, is not apparent.

## Tooth wear

**Premaxillary teeth.** The premaxillary teeth occupy the posterior one-half of the premaxillary alveolar margin. The lingual face of premaxillary crowns opposes the edge of a keratinous beak covering the predentary (Fig. 95). This tooth-to-bill contact has generated large planar wear surfaces on pm2 and pm3 in *Lycorhinus* (Fig. 76). These shearing wear facets were figured but not identified when first described (Thulborn 1970: fig. 2; mentioned later in Thulborn 1974: 163). The crown of pm1 may have been similarly worn but is broken away.

Similar shearing wear facets are present in the premaxillary teeth of other heterodontosaurids. In *Heterodontosaurus* Crompton and Attridge (1986: 229) remarked there are “wear facets on the premaxillary teeth” but never supported the observation with reference to specific specimens. The left premaxillary teeth in adult skull (SAM-PK-K1332) are the most completely exposed and provide the best evidence for wear in the premaxillary series. Sometime after this specimen was initially prepared, however, the entire crown of pm2 was lost and the crown of pm3 has sustained some damage (Norman et al. 2011: append. 4). A cast from an early mold of the left side of the snout of this specimen (UCRC PVC11), nevertheless, preserves pm2 and pm3 crowns with excellent fidelity (Fig. 91).

The crown of pm2 provides evidence for three kinds of tooth wear. First, a large planar wear facet from abrasion against the bill of the predentary is present on the lingual side of the crown (Fig. 91B). Only the mesial and distal edges of the planar facet are exposed, with the remainder pressed against the matrix. Second, a tongue-shaped sliver of the crown is missing from the mesial aspect of the labial surface of the crown and appears to represent an axially-oriented fragment chipped from the crown by impact and concave in cross-section (Fig. 91B, safs). In its generally concoidal form and orientation, the missing fragment resembles “spalling”, as described recently in theropod teeth (Schubert and Unger 2005). The enamel edges of the spalled fragment are rounded from subsequent abrasion. Finally, the apical end of the crown is rounded from tooth-to-food apical abrasion, leaving a polished surface distinct from the remainder of the crown (Fig. 91B, aa).

The caniniform crown (pm3) preserves a large planar wear facet on its lingual side similar to that described above in *Lycorhinus*. This shearing wear facet also was drawn and identified in camera lucida tracings of the tooth as formerly preserved (A. W. Crompton, unpublished drawings). In the early cast of this tooth, the straight mesial and distal edges of the facet are visible (Fig. 91B, elwf). In addition to this tooth-to-bill wear facet, there are two breakage surfaces on the same crown that truncate its apical end. The first and larger of the two has an uneven surface that truncates the crown at a high angle. The edges of this breakage surface are rounded and polished (Fig. 91B, bsae). The second, smaller breakage surface is tongue-shaped and slightly mesiodistally concave. This missing fragment resembles the spalled fragment in premaxillary tooth 2 but appears to have occurred more recently, as its enamel edges are only slightly polished (Fig. 91B, sfs). The distal carina lacks serrations, which are present in the comparable carina of pm3 in *Lycorhinus*. The distal carina, however, appears slightly rounded and polished, suggesting that the original ornamentation may have been worn away in this mature individual of *Heterodontosaurus*. On the right side, pm3 does not have a comparable wear facet on the lingual face of the crown.

Premaxillary teeth are also preserved on both sides of the holotypic skull (SAM-PK-K337), although none is as complete and as well exposed as in SAM-PK-K1332 (Norman et al. 2011: fig. 20). The left pm2 and right pm3 show evidence of apical breakage with subsequent polishing by abrasion. In both cases, the crowns are preserved *in situ* in matrix with their apical ends broken away. In the crown of left pm2, a flat breakage surface is inclined mesially, its labial edge rounded and polished. In the crown of right pm3, an irregular breakage surface is lingually inclined and shows rounding or polishing of its edges (Norman et al. 2011: fig. 20).

None of these wear facets or the polished breakage surfaces have been described as such in *Heterodontosaurus*. Norman et al. (2011: 50), to the contrary, concluded “The absence of significant levels of wear and the lack of occlusal relationships between upper and lower caniniforms is not consistent with their use for cropping or rooting for vegetation.” The evidence of breakage and wear described above in *Heterodontosaurus* and *Lycorhinus*, nonetheless, clearly supports opposing conclusions. First, there is excellent evidence in *Lycorhinus* and *Heterodontosaurus* for broad, planar tooth-to-bill shearing wear facets on the lingual aspect of some premaxillary crowns. The lower bill, thus, likely functioned in cropping vegetation by shearing against the lingual aspect of at least some of the premaxillary crowns and upper bill. Second, loss from breakage and spalling appears to be commonplace at the apical end of premaxillary crowns in these genera. These apical breakage surfaces were then polished by subsequent abrasion.

The caniniform crowns thus were put to considerable use in cropping and possibly in rooting functions. Crown breakage in the premaxillary series suggests at least occasional contact with hard materials, as may occur in the course of agnostic or rooting behaviors. Loss of the crown tip or obliteration of marginal serrations, furthermore, does not appear to have curtailed their function, as might be the case if they played a significant role in carnivory.

**Dentary caniniform teeth.** In *Lycorhinus* a lingual “wear facet” was described near the tip of the dentary caniniform tooth in the holotypic dentary (Gow 1990: 372, fig. 2). Wear facets are generated either by tooth-to-tooth or by tooth-to-bill shearing contact. Inspection of the crown tip under magnification, however, casts doubt on this interpretation. First, the surface is mesiolingually inclined, which is difficult to explain as a product of either tooth or upper bill contact, as the available opposing dental or bill structures would contact the opposite side of the crown. Second, the surface of the supposed facet is slightly concave rather than flat, and the crown tip shows abrasive rounding. The structure, thus, is here interpreted as a spalled fragment of the tooth surface lost by impact near the tip of the crown and then rounded by subsequent abrasion.

In *Heterodontosaurus* a labial “wear facet” was described at the tip of the right dentary caniniform tooth (Norman et al. 2011: 215-216, fig. 18B), the presence of which the authors were pressed to explain:

“there is no obvious caniniform occlusion that might have generated such a facet. One possibility is that this facet reflects some malocclusion with the opposing caniniform. Other (much less likely) proposed of the uses of the caniniforms for digging and or some type of agonistic behavior might be considered as potential means for accounting for such a facet . . . but the absence of more general indications of wear and retention of marginal denticles [*sic* serrations] seems to obviate digging/rooting as the cause.”

Inspection of the crown tip under magnification, however, casts doubt on its identification as a wear facet. The tip of the crown is broken, leaving an irregular rather than planar surface, the edges of which show clear signs of abrasive polishing. The same pattern of breakage with subsequent abrasive polishing is described above in premaxillary crowns, although in dentary caniniform crowns there is no evidence of planar shearing wear facets.

When the jaws of SAM-PK-K1332 are drawn apart, the tips of the enlarged pm3 and d1 caniniform teeth are positioned near one another in effective opposition for a nipping bite (Fig. 93B). As the jaws close, in contrast, the dentary caniniform tooth is drawn posteriorly away from the premaxillary caniniform tooth and sequestered within an inset diastema (Fig. 93A). There is no opposing tooth that could generate tooth-to-tooth contact with the dentary caniniform tooth. The absence of evidence for tooth-to-tooth or tooth-to bill wear on dentary caniniform teeth, thus, is not surprising. The chipped or broken and subsequently polished ends of dentary caniniform crowns in *Lycorhinus* and *Heterodontosaurus*, nevertheless, suggest that loss and subsequent polishing of the crown tip was common in mature individuals. There is no evidence that these blunted caniniform teeth were replaced during adulthood, but rather they continued to function in feeding, and possibly agonistic or defensive, behaviors.

**Cheek teeth.** Hatchling and juvenile heterodontosaurids may have subsisted on a softer diet that did not generate significant tooth wear, as appears to be the case in the very small Kayenta heterodontosaurid and perhaps some of the smaller specimens of *Fruitadens* (Fig. 9A, B). Shearing wear facets, however, are present in the adult dentition of even the smallest heterodontosaurids with simple subtriangular crowns. In *Echinodon*, shearing wear facets are present on all fully erupted maxillary crowns that are well preserved and exposed in lingual view (Fig. 14B, C). Small low-angle wear facets are also present on the labial side of dentary crowns (NHMUK 48213), which resemble those on the lingual side of maxillary crowns. These facets truncate the crown at a low angle from the vertical axis of the tooth. They do not resemble the high-angle wear facets that dominate the dentition in adult specimens of *Lycorhinus* (Figs 3B, 77C) and *Heterodontosaurus* (Figs 55, 56).

In *Echinodon* the smooth, planar surface of wear facets can be readily distinguished under magnification from more irregular breakage surfaces, although these have been confused in previous accounts. High angle “apical wear facets” were described and figured in maxillary crowns of the lectotype (NHMUK 48209, 48210; Norman and Barrett 2002: 177, fig. 7B). These breakage surfaces, however, clearly represent damage generated after the specimens were collected and initially figured. The most mesial of these “facets”, for example, is on a crown that was complete when originally figured by Owen (NHMUK 48210; compare Owen 1861: pl. 8, fig. 2). “Small apical wear facets” were also reported in maxillary teeth of another specimen (NHMUK 48211; Norman and Barrett 2002: fig. 8A, C). These crowns, however, are exposed only in labial view, so the missing crown tips also are surely the result of postmortem damage.

Tooth wear in heterodontosaurine cheek teeth is usually very well developed. In some genera, such as *Heterodontosaurus* and *Pegomastax*, a nearly continuous wear surface is present in the larger cheek teeth (Figs 55, 85). The general continuity of the wear surface is well preserved in AMNH 24000, in which the crowns are butted against one another and the wear surfaces are aligned in a nearly a single plane (Figs 41, 42). This specimen also shows that this derived wear pattern is maintained despite active tooth replacement (Figs 43–52).

In *Lycorhinus* a near alternating alignment of upper and lower cheek teeth has generated a pair of wear facets per crown (Hopson 1975, 1980; Gow 1990; Fig. 3C). In *Heterodontosaurus* skulls with upper and lower jaws in articulation show variation in the relative position of upper and lower crowns, although none preserve one-to-one or alternating alignment. On both sides of the adult skulls (SAM-PK-K337, -K1332), the maxillary crowns are positioned slightly distal to opposing dentary crowns in the center of the tooth row (Fig. 55). In the subadult skull (AMNH 24000), the maxillary crowns are positioned slightly mesial to opposing dentary crowns (Fig. 41). The dentary in this specimen, however, is slightly disarticulated from the surangular, which appears to have misaligned the upper and lower tooth rows (Fig. 40). The wear facets on the dentary crowns are offset distal to the opposing maxillary crowns. With the tooth rows realigned, the relation would appear to be similar to that in the adult skulls, with maxillary crowns positioned slightly distal to opposing dentary crowns across most of the tooth row.

Hopson (1980) described how this overlapping alignment in *Heterodontosaurus* results in two adjacent wear facets on each dentary crown when they are heavily worn. A large facet is generated from the principal crown in opposition. A smaller triangular facet, here termed an “accessory facet”, is generated by the corner of a second maxillary crown. A number of accessory facets are present in dentary series in an adult skull of *Heterodontosaurus* (Fig. 55B, C). Their polished edges suggest that they were subject to abrasive wear from the processing of plant materials held within the cheek emargination after their initial formation.

In the worn dentition of SAM-PK-K1332, both maxillary and dentary facets are gently transversely concave, the dentary wear facets more strongly concave than maxillary wear facets (Fig. 89B). The distal three crowns in the dentary series are also concave mesiodistally (Fig. 55). Measured from a parasagittal reference plane through the tooth row, the occlusal plane is angled ventrolabially at approximately 55° at the mesial end of the tooth row and increases to approximately 65° at the distal end (Crompton and Attridge 1986; SAM-PK-K337, -K1332; Fig. 60B). Because the maxillary crowns are canted lingually at approximately 25° toward the occlusal plane, the angle of the facet relative to the crown axis is correspondingly greater, or about 80-90° (Fig. 89B). The facet, thus, truncates maxillary crowns at nearly a right angle to the crown axis. The dentary teeth are canted labially to a lesser degree, approximately 10°, and so the angle of the facet relative to the crown axis is lower, or about 65–75°. The angle of the facets relative to the crown is considerably higher in *Heterodontosaurus* and *Lycorhinus* than in *Echinodon* (Fig. 14A, B).

The tooth rows of SAM-PK-K1332 suggest that the inclination of wear facets increases from low- to high-angle at the mesial end of the dentary and maxillary tooth series (Fig. 60). The crowns of d2-3, for example, have low-angle wear facets that diverge less from the axis of the crown. In dorsal view, these small mesial dentary teeth curve labially from the axis of the remainder of the tooth row (Fig. 60B). Likewise, the most mesial maxillary teeth appear to have low-angle wear facets (Norman et al. 2011: fig. 24). The mesial pair of cheek teeth in both upper and lower tooth rows is displaced labially from the axis of the remainder of their respective cheek tooth row (Fig. 60A). Thus, the cheek tooth rows are not completely linear (*contra* Norman et al. 2011: fig. 13).

Hopson (1980) suggested that the inclination of wear facets increases with age and/or with stage of wear of a particular crown in *Lycorhinus*. The newly discovered subadult specimen of *Heterodontosaurus* (AMNH 24000) provides strong evidence in favor of this hypothesis, which may characterize other heterodontosaurids with deeper crowns proportions. A striking feature of the subadult specimen of *Heterodontosaurus* is the low-angle, near vertical occlusal plane preserved in the distal region of the tooth row (Figs 40–42). The wear facets are set at an angle to the crown axis of approximately 15° at most, which is approximately 50° less than angle of comparable wear facets in the distal dentary teeth of SAM-PK-K1332 (Fig. 55). The differential in occlusal angle is not an artifact of preservation, as the posterior end of the subadult skull is only slightly transversely compressed, as is also the case in the adult skull SAM-PK-K1332. The low angle of incidence of the wear facets in the subadult skull nearly obliterates the entire labial surface of the dentary crowns (Figs 41, 42). These facets are slightly mesiodistally concave due to a raised edge along the distal margin of the crown.

Sustained wear is the most likely explanation for the stark increase in the angle of incidence of the wear facets between subadult AMNH 24000 and the two adult skulls (SAM-PK-K3606, -K1332). The crowns in AMNH 24000 preserve secondary ridges in maxillary crowns, apical portions of the dentary crowns, and active tooth eruption. With more sustained wear in SAM-PK-K1332, few secondary ridges remain on maxillary crowns, most of the posterior dentary crowns are obliterated, and there are no erupting crowns in either tooth row (Fig. 55).

### Masticatory function

**Previous hypotheses.** Hypotheses regarding heterodontosaurid masticatory function have been proposed thus far on the basis of *Lycorhinus* and *Heterodontosaurus*. Wear facets in cheek teeth are also known in *Pisanosaurus* (Fig. 6) and are described in this study in *Echinodon* (Fig. 14), *Abrictosaurus* and *Pegomastax* (Fig. 85). The discussion of masticatory function, nevertheless, focuses on *Heterodontosaurus tucki*, because it is the only heterodontosaurid represented by reasonably complete skull material with well preserved and exposed tooth rows. Future work on the skull and dentition in *Tianyulong* may add another perspective on masticatory function in a less modified heterodontosaurid.

**Figure 88.**
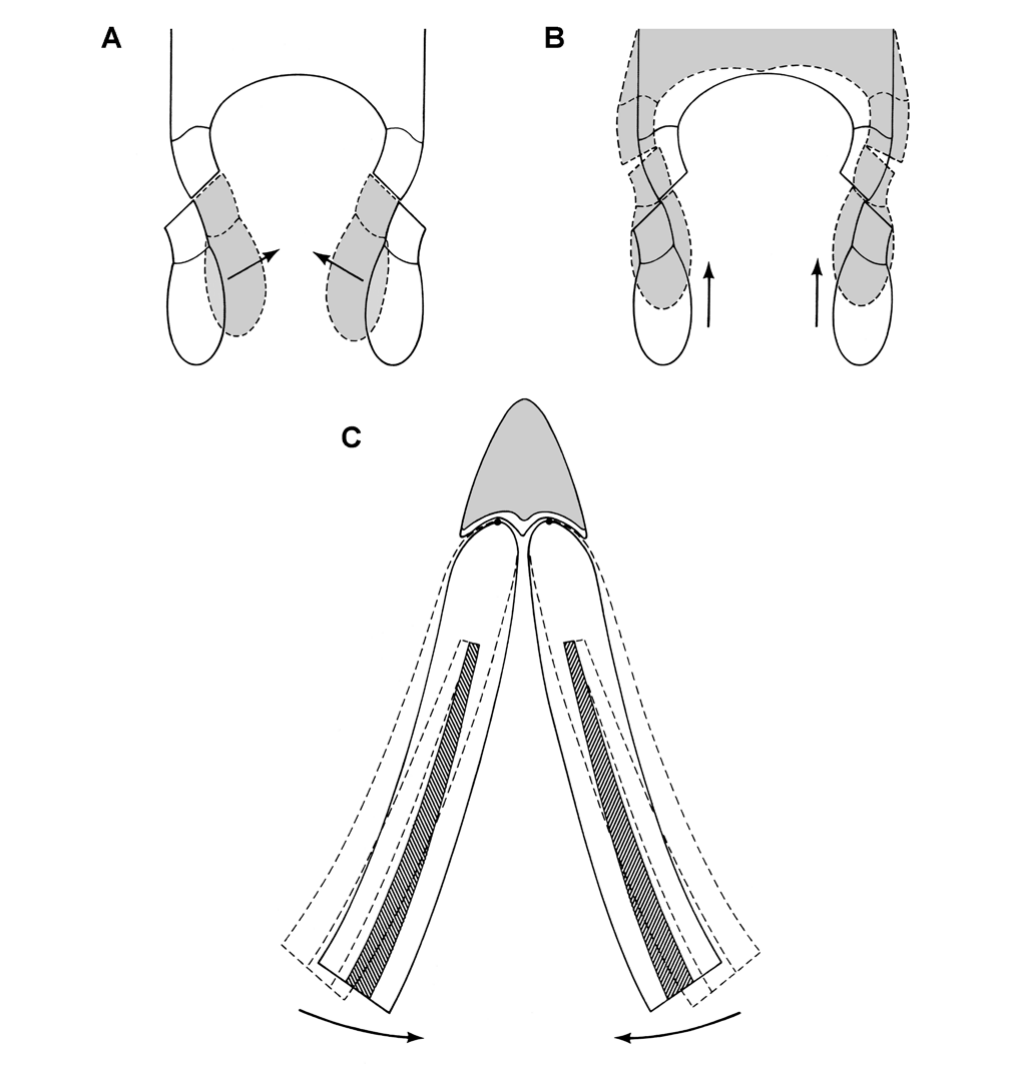
Hypotheses for jaw movement during tooth-to-tooth occlusion in *Heterodontosaurus tucki*. Jaw movements (arrows) to account for shearing wear facets during isognathous occlusion (from Crompton and Attridge 1986) **A** Dorsomedial movement of the lower jaws against stable maxillary tooth rows **B** Dorsal movement of the lower jaws with lateral flaring of the maxillary tooth rows **C** Medial rotation of the dentaries pivoting anteriorly at the predentary-dentary joint.

Two general kinds of isognathous jaw mechanisms have been proposed to account for the truncating, high-angle wear facets observed in the teeth of *Lycorhinus* and *Heterodontosaurus*—*propalinal* (fore-aft) and *arcilineal* (vertical or near vertical) (Reilly at al. 2001). Within the arcilineal paradigm, several hypotheses have been proposed to account for the transverse jaw movement required to generate high-angle (non-vertical) wear facets in upper and lower cheek tooth rows. These jaw mechanisms, which are not necessarily mutually exclusive, include:

(1) *medial rotation* about the long axis of the lower jaw (Weishampel 1984: fig. 16j; Norman and Weishampel 1985);

(2) *medial flexion* (“wishboning” or “scissoring”) of the dentaries at the predentary-dentary joints (Crompton and Attridge 1986) (Fig. 88C);

(3) *medial flexion* within the lower jaws at mid-length (Porro 2007);

(4) *lateral excursion* of the quadrate (Porro 2007).

Crompton and Attridge (1986) presented diagrammatic versions of alternative jaw mechanisms, although the match to the four listed above is not clear in some cases (Fig. 88). The first is a cross-section showing *dorsomedial displacement* (Fig. 88A), in which each dentary tooth row moves “inward relative to stationary uppers” (Crompton and Attridge 1986: 230). Linear movement of the lower jaws as depicted, nonetheless, is not possible without some medial rotation or flexion, because the tooth rows converge to a median dentary symphysis that is constrained at the midline. This first diagram, thus, is incomplete at best.

The second mechanism is a cross-section showing *lateral rotation* of the maxillary tooth rows with vertical movement of stationary dentary tooth rows (Fig. 88B). No one has supported this mechanism, because there is no evidence of kinetic articulations within the sidewall of the snout to accommodate transverse movement. The first two diagrams, in fact, do not correspond to any of the four jaw mechanisms listed above.

The third jaw mechanism, identified here and listed above as *medial flexion* of the dentaries, shows medial rotation of each dentary about a vertical axis through the predentary-dentary suture (Fig. 88C). Several aspects of this diagram, however, depart from the known structure of the lower jaw in *Heterodontosaurus*. First, there is a substantial median symphysis between the anterior ends of the dentaries, which is nearly as long as the predentary (Fig. 61A). In ventral view of the lower jaws, only the distal half of the symphysis is exposed posterior to the predentary (Fig. 61B). Second, the predentary-dentary joint is not “spheroidal” or a “smooth ball-and-socket” as described and diagrammed by Weishampel (1984), Norman and Weishampel (1985) and Crompton and Attridge (1986). Rather this articulation is saddle-shaped, with a surface at the expanded anterior end of the dentary that is dorsoventrally convex and transversely concave (Fig. 39). In horizontal cross-section, the predentary-dentary joint would have the opposite polarity to that shown by Crompton and Attridge (Fig. 88C), with convex and concave predentary and dentary surfaces, respectively.

**Figure 89. F89:**
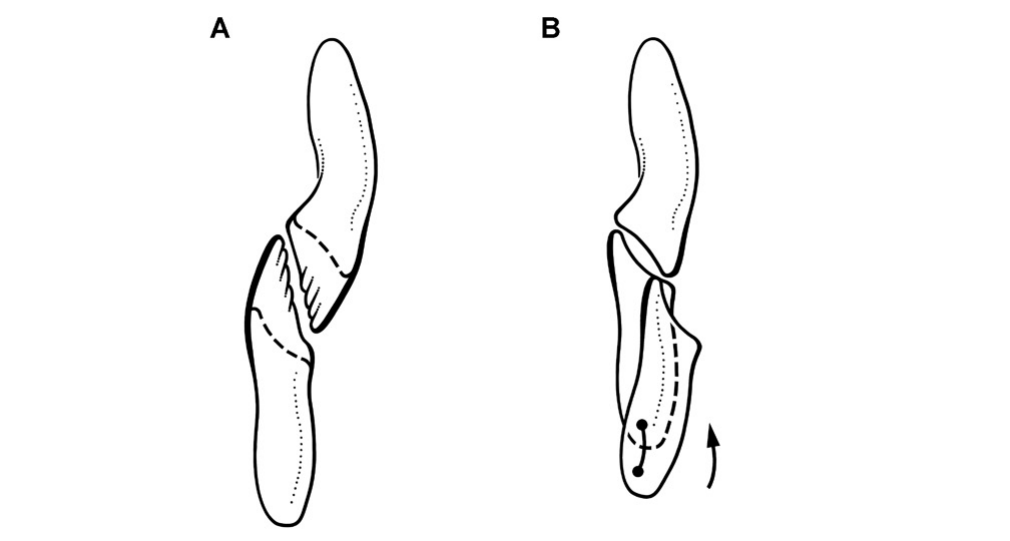
Occlusion and wear between maxillary and dentary teeth in *Heterodontosaurus tucki*. Reconstruction of left maxillary and dentary teeth in anterior view at different stages of wear **A** Dentition in a subadult with unworn crowns and near vertical occlusal plane with dashed lines indicating the occlusal surface of a worn crown (based on AMNH 24000) **B** Worn crowns from the middle of the tooth rows showing labiolingually concave wear facets, which are more concave in dentary than maxillary crowns (based on SAM-PK-K1332). The dentary tooth is shown at the initiation and conclusion of a masticatory stroke, with a dashed line showing the tooth margin hidden from view and registration dots and an arrow indicating tooth movement during occlusion.

The saddle-shaped predentary-dentary joint, which is also present in *Pegomastax* and *Abrictosaurus*, appears to be designed to enhance dorsoventral movement of the predentary (see below). The transverse width of the dentary articular surface, in addition, appears to be larger than the width of the predentary, which would have allowed medial flexion of the dentaries against the predentary (“wishboning”) as diagrammed by Crompton and Attridge (Fig. 88C). The hypothesis of medial flexion of the dentaries at the predentary-dentary joints, nonetheless, entails motion elsewhere in the skull, given that the dentary is an integral part of the lower jaw connected posteriorly at the jaw joint to the cranium. The mechanism of medial flexion of the dentaries, thus, requires compensatory medial flexion at mid-length along the lower jaw (hypothesis 3) and/or medial movement of the quadrate or jaw joint. It is difficult to understand how lateral displacement, or splaying, of the jaw joint (hypothesis 4) would facilitate medial flexion of the dentaries (hypothesis 3). The well-formed socket in the squamosal for the head of the quadrate and the vertically elongate quadrate-quadratojugal articulation do not support lateral movement of the quadrate shaft (hypothesis 4).

Norman et al. (2011: 253) did not endorse a particular jaw mechanism but seemed to support flexion within the lower jaw (hypothesis 3) by stating a “remnant of the archosaurian mid-mandibular joint permitted flexure within the dorsal part of the lower jaw that was linked to a highly unusual jaw action”. Elsewhere they stated “unusual sutural relationships between the bones along the dorsal edge of the lower jaw” contributed to a “novel form of jaw action” in *Heterodontosaurus* (Norman et al. 2011: 182). As discussed above, the form and sutural relations of the postdentary bones in *Heterodontosaurus* are not unusual. Damaged bone in the coronoid region of SAM-PK-K1332 led to an erroneous reconstruction of this region of the lower jaw (Fig. 58). Thus far, little evidence has been published to support intramandibular flexion (hypothesis 3) or transverse splaying of the quadrate (hypothesis 4) in *Heterodontosaurus*.

**Caution and constraints on inferred movements and muscles.** Caution is warranted when extrapolating from bony or dental landmarks to *jaw muscles* and *hypothetical jaw movements* in a specialized ornithischian dinosaur with a differentiated dentition, because there is no close modern analog. Recourse to extant archosaurs (Crocodylia, Aves) provides phylogenetic context for the reconstruction of jaw muscles (Witmer 1995; Holliday 2009), although avians specialized for durophagy present a range of possible muscle reconstructions (Sereno et al. 2010: Fig. 97A). Hypothetical muscle groups in heterodontosaurids, in addition, may have a closer functional analog among mammalian herbivores, given the parallel evolution of adductor insertion on the dentary and enhanced buccal processing of foodstuffs.

In *Heterodontosaurus* wear facets with radically different orientations are generated in the cheek teeth of skulls that differ only in size. Key structural areas of subadult and adult skulls appear identical, such as jaw joint structure and tooth orientation and form. Yet, the near vertical wear facets in the subadult skull AMNH 24000, for example, differ by as much as 50° from high-angle wear facets in the adult skull SAM-PK-K1332. This observation alone is grounds for caution when settling on a functional mechanism.

In the opinion of this author, the skull of *Heterodontosaurus* alone is not “sufficient to determine the distribution and orientation of the principal jaw-closing muscles” nor is reconstruction of the adductor musculature “required in order to understand the general function of the jaws and teeth during feeding” (*contra* Norman et al. 2011: 225). Two previous attempts to reconstruct the main adductor musculature in *Heterodontosaurus*, for example, have generated very different solutions for the distribution and differentiation of muscle groups (Figs 94A, 94B-E). The configuration of adductor musculature presented below (Figs 95, 96) is limited to the jaw-closing muscles of the adductor mandibulae muscle group and differs substantially from that proposed recently by Norman et al. (2011) (Fig. 94B-E). Finite-element modeling of bone and/or muscle configurations is an heuristic exercise (Porro 2007), but it involves a suite of assumptions regarding joints, muscle mass and muscle distribution that are particularly difficult to estimate in extinct forms without close modern analogs (Rybczynski et al. 2008).

Norman et al. (2011: 230-231) listed nine observations or constraints for potential jaw mechanisms in adult individuals of *Heterodontosaurus*. The following five, which are rephrased and in some cases combined, are regarded here as strongly supported by available fossil material:

1) Jaw movement is isognathous and fundamentally arcilineal rather than propalinal, based on mesowear (i.e., macroscopic evidence of wear direction) and the form of the jaw joint;

2) Shearing between upper and lower premaxillary teeth and the edges of keratinous upper and lower bill sheaths results in near vertical wear facets on premaxillary teeth and would have limited transverse movement at the anterior end of the lower jaws;

3) Wear facets are high-angle (~ 45-65°) in the central portion of upper and lower cheek tooth rows;

4) Wear facets are gently transversely concave in the central portion of upper and lower cheek tooth rows, a feature more pronounced in the lower than the upper tooth row.

5) Wear facets tend to be lower-angle in the premaxillary teeth and the mesial end of upper and lower cheek tooth rows.

Norman et al. (2011) regarded the form of the arched diastema, dentary symphysis, and pterygoid and jugal flanges as additional constraints, eliminating the possibility of any long-axis rotation of the lower jaw. Given the small amount of rotation needed to accommodate shearing occlusion in *Heterodontosaurus*, however, these features may not have functioned as effective constraints in the manner proposed. The lower jaw must be displaced transversely by at least 2 mm to have generated the broadest wear facets. The width of the arched diastema in SAM-PK-K1332 is approximately 3 mm. For long-axis rotation to account for all of the transverse motion of the dentary crowns against stationary maxillary crowns, the tip of the dentary caniniform tooth would be expected to swing across a somewhat broader transverse arc, as it is positioned about 4 mm farther from the axis of rotation than the occlusal surface of the dentary teeth. An arc of perhaps 2.5 or 3 mm could be accommodated within the diastema. At least some long-axis rotation of the dentary, thus, cannot be excluded as impossible on the basis of the invaginated pocket of the diastema. Likewise, pterygoid and jugal flanges cannot be shown to articulate so closely with the lower jaw that they restrict movement within a narrow slot. Neither the pterygoid nor jugal contacts the lower jaw or has a fortified margin that could function as a guide during jaw movements, as occurs in some crocodylomorphs (Iordansky 1964).

If the prevention of long-axis rotation was important, furthermore, a deeper more vertical symphysis would provide more effective resistance. In *Heterodontosaurus* the dentary symphysis is a ligament-bound syndesmosis. The surface devoted to the symphysis (Fig. 61A) is slightly less extensive than figured by Norman et al. (2011: fig. 19B). The symphyseal surface is limited to the ventral one-half of the end of the dentary. The dorsal one-half of the end of the dentary forms a trough-shaped surface that separates the lower caniniform teeth to each side of the midline (Figs 60B, 61A).

**Propalinal versus arcilineal jaw movement.** Thulborn (1974, 1978) argued for propalinal jaw movement, basing this argument loosely on the perceived need to maintain “continuous” wear surfaces in cheek tooth rows. Hopson (1980), to the contrary, argued that wear facets in *Lycorhinus* and *Heterodontosaurus* are not continuous and that the disposition of the wear facets is incompatible with propalinal movement. Thulborn’s proposal of propalinal jaw movement in heterodontosaurids has been defended only in unpublished research as the principal (Barrett 1998) or optional (Porro 2009) jaw mechanism. Two lines of evidence, namely the structure of the jaw joint and mesowear, eliminate propalinal jaw movement as a viable hypothesis to explain tooth wear in *Heterodontosaurus*.

A partial subadult skull of *Heterodontosaurus* (AMNH 24000) preserves articulated right and left jaw joints, showing the fit between the rounded quadrate condyles and concave articular socket on the lower jaw (Fig. 61C, D). On the right side, the lateral extremity of the larger posteroventrally offset lateral condyle and the adjacent edge of the surangular are eroded, exposing in cross-section the articular contact between this condyle and the articular socket of the lower jaw (Fig. 61C). The articular socket is particularly deep anteriorly, where it rounds dorsally to a near vertical orientation—an immobile stop for the quadrate condyle, which measures 4.2 mm in anteroposterior diameter. On the left side, the edge of the surangular is completely preserved and has a very low projection, which is medially concave and seems to wrap around the lateral side of the quadrate condyle (Fig. 61D). On this side, the posterior portion of the quadrate condyle is eroded away, exposing the posterior portion of the articular cup, which measures 4.2 mm in anteroposterior length. Thus the fully preserved condyle would fit snugly into the concave articular socket on the lower jaw. On this side, the medial edge or lip of the articular cup curves dorsomedially, shaped to fit and partially contain the medial condyle of the quadrate. The shape of the articular cup, the small lateral surangular process, and the medial lip of the articular cup are also preserved on both sides in SAM-PK-K1332 (Fig. 59).

Thus the jaw articulation is a well-fitted rotary joint, unsuitable for significant anteroposterior motion of the quadrate condyle. The deep anterior wall of the socket, in particular, would prevent any significant posterior motion of the lower jaw relative to the quadrate. The anteroposterior width of the socket equals the width of the quadrate condyle in the subadult skull (AMNH 24000); socket width may be slightly greater than the width of the quadrate condyle in SAM-PK-K1332 (Norman et al. 2011: 211). Both skulls suggest that significant anteroposterior displacement of the lower jaw relative to the quadrate was not possible. Propalinal movement at the jaw articulation is extremely rare among archosaurs. In *Psittacosaurus*, the best-documented case for propaliny among non-avian dinosaurs, the quadrate condyle is free to move fore and aft, articulating against the flat articular surface of the glenoid (Sereno et al. 2010; Fig. 97B). The condition in *Psittacosaurus* bears no resmblance to the snug concavoconvex glenoid articulation in *Heterodontosaurus*.

Several aspects of the lower jaw joint in *Heterodontosaurus* have not been depicted accurately (Fig. 58). The articular socket in the lower jaw was shown as relatively shallow and separated from the rising margin of the surangular (Norman et al. 2011: Figs 8, 11, 16, 19). The lateral quadrate condyle is the larger of the two (maximum anteroposterior diameter 5.3 mm versus 4.9 mm in SAM-PK-K1332) but was shown as smaller (Norman et al. 2011: fig. 13). Finally, the quadrate condyles are not fully exposed in lateral view (Norman et al. 2011: fig. 8) but rather are contained in part by the lateral lip of the surangular (Norman et al. 2011: fig. 4) (Fig. 61D). The jaw articulation in *Heterodontosaurus* appears to be a strictly rotary, rather than sliding, joint.

Mesowear also strongly contradicts propalinal jaw movement as a viable hypothesis to explain tooth wear in *Heterodontosaurus*, as noted by Norman et al. (2011: 231). Arcilineal jaw movement, on the other hand, can account for wear facets in adult (SAM-PK-K1332) and subadult skulls (AMNH 24000) that preserve opposing upper and lower tooth rows. Wear facets can be aligned between upper and lower tooth rows (Hopson 1980), and sometimes these wear facets preserve low marginal edges that indicate the occlusal passage of one crown across an opposing crown. These macroscopic ridges are preserved between high-angle wear facets on adult cheek teeth (Norman et al. 2011: fig. 27) and between or within low-angle wear facets on subadult cheek teeth (Fig. 42). Finally, transverse cupping of both upper and lower wear facets in the cheek teeth of adult dentitions (Fig. 89) is impossible to generate with propalinal occlusion alone.

**Transverse jaw movement.** Tooth wear in adult individuals of *Heterodontosaurus* requires transverse translation of the dentary crowns across opposing maxillary crowns during occlusion. Judging from the maximum width of the wear facets in SAM-PK-K1332, this would involve transverse displacement of at least 2 mm. How was this accomplished?

Almost nothing has been said about the jaw joint in discussions about masticatory movement in heterodontosaurids. In *Heterodontosaurus* the quadrate condyles are not a simple cylinder positioned orthogonal to the long-axis of the lower jaw. In both subadult and adult skulls, the larger lateral quadrate condyle is offset ventral to the medial condyle, such that in posterior view the distal articular surface angles ventrolaterally at about 30° (Fig. 92A). In ventral view, the axis across the condyles is angled anteromedially at about 40° from a transverse axis (Fig. 92B). Finally, in palatal view, the long axis of the tooth rows diverge approximately 10° from the midline for an interdental angle of 20° (Fig. 92B). In the best preserved adult skull of *Heterodontosaurus* (SAM-PK-K1332), the cheek tooth rows are transversely compressed (Norman et al. 2011: fig. 6) and have been reconstructed here independently with the same interdental angle of approximately 20° (Norman et al. 2011: fig. 13) (Fig. 58). The articular socket of the lower jaw, as described above, is fitted to the quadrate condyles, and thus the joint would have been constrained for rotation. *Manidens* seems to have a similar jaw joint, as much as can be determined from its present state of preparation (Pol et al. 2011; Fig. 81B).

It is important to determine the effect of a canted jaw joint on the arc of the lower jaw, as it nears occlusion with the maxillary tooth row. To better understand this, a physical model of upper and lower jaws was created at 10 times natural size to exaggerate subtle transverse displacement. In the model as in *Heterodontosaurus* (Fig. 92), the jaw joint was set below the tooth row and canted ventrolaterally and anteromedially. In anterior view, the canted jaw joint rotates the lower jaw slightly about its long-axis during jaw closing, such that the dentary tooth row rotates counterclockwise (Fig. 89B). This gentle arc, which appears to be built into the jaw joint, provides an explanation for the transverse dishing of the wear facets in the central portion of the cheek tooth rows in SAM-PK-K1332. The thickened enamel on the leading edge of maxillary and dentary crowns would scour the central part of the opposing crown, as the dentary tooth changes its angle of incidence during occlusion (Fig. 89B). Although no such long-axis rotation need be invoked to explain the near vertical wear facets in the subadult dentition (Fig. 89A), some long-axis rotation is required to generate the opposing, transversely concave wear facets in the adult dentition (Fig. 89B).

Some medial flexion (“wishboning” or “scissoring”) of the dentaries at the predentary-dentary joints may also have occurred (Fig. 88C). Such movement would generate more translational movement in distal than mesial portions of the tooth rows. The vertical premaxillary and low-angle wear facets in the mesial crowns of the cheek teeth suggest that some medial flexion did occur at predentary-dentary joint in adult *Heterodontosaurus*.

**Caniniform function.** The premaxillary series appears to function foremost in the puncturing and cropping of plant material. Mature specimens of *Heterodontosaurus* and *Lycorhinus* provide the best evidence. The premaxillary canine (pm3) and at least the smaller pm2, as detailed above, have large planar wear facets on their lingual sides generated by abrasion with the cutting edge of the predentary bill (Figs 76, 91). In several specimens, the tip of the premaxillary caniniform tooth is broken away *in situ*, the breakage surface polished by subsequent abrasion. The broken crown tip, thus, was probably used in the processing of foodstuffs. Because pm3 is not enlarged or caniniform in several heterodontosaurids, such as *Echinodon*, *Fruitadens* and *Abrictosaurus* (Figs 9A, 19, 35), it seems unlikely that it was used for capturing or subduing prey.

The relation between upper and lower caniniform teeth in *Heterodontosaurus* suggests that the enlarged pm3 and d1 may have served a useful nipping function in agonistic behavior among peers or defense but may not have functioned well in prey capture or processing. Because the jaw joint is depressed below the occlusal plane of the cheek teeth, the dentary caniniform tooth swings anteroventrally as it moves from its protected position within the arched diastema (Fig. 91A) to a slightly prognathous position with a reasonable gape (Fig. 91B). In this open pose, the tips of the premaxillary and dentary caniniform teeth approximate one another and might well have provided a potent nipping or slashing function. As the jaws close, however, the pointed tips of dentary and premaxillary caniniform crowns and their serrate carinae move apart. The opposing caniniform premaxillary and dentary caniniform teeth thus would not appear to function particularly well in slicing muscle tissue. The puncturing and slicing of prey would be further hindered by entry of the tip of the dentary caniniform tooth into the invaginated diastema.

**Predentary mobility.** The anterior end of the dentary in some advanced heterodontosaurids is dorsoventrally expanded with a semicircular contour in lateral view, as preserved in *Pegomastax*, *Abrictosaurus* and *Heterodontosaurus* (Figs 31, 35, 39, 59, 87). The predentary articulates against a smooth saddle-shaped articular surface, which appears dorsoventrally longer and transversely broader than needed to accommodate the predentary. The oversized, smooth articular surface may have allowed the predentary to slide a short distance dorsoventrally, guided by the shallow trough of the articular surface. If the predentary was mobile to some degree, then the keratin sheath it supported would not have extended across the joint and onto the adjacent surface of the dentary (Fig. 95) as it does in some ornithischians (Fig. 97B).

In heterodontosaurids with simple subtriangular tooth crowns, the variable form of the predentary-dentary joint does not suggest similar predentary mobility. In *Echinodon* the articular surface for the predentary is a smooth gently convex surface positioned on the ventral aspect of the end of the dentary (Figs 17, 19B). In *Tianyulong* impressed vascular channels on the dentary ramus suggests that the keratin sheath may have extended posteriorly from the predentary along the ventral edge of the buccal emargination on the dentary (Figs 22, 23). In the Kayenta heterodontosaurid, the articular surface for the predentary is a well-defined subtriangular surface that faces anterolaterally (Fig. 9B).

**Inferred jaw musculature.** The adductor chamber in *Heterodontosaurus* is relatively large. In dorsal view, the supratemporal fossa is equal in length to the orbit, and in lateral view the large volume of the chamber is visible through the laterotemporal fenestra. Depressions for muscle attachment are present along the perimeter of both supra- and laterotemporal fenestrae. The anterior margin of the supratemporal fossa is deeply inset, its medial margin bounded by a sharp sagittal crest. A deep-rimmed fossa is present on the body of the postorbital. This fossa narrows as it passes down the ventral ramus of the postorbital. Similarly, a narrow-rimmed fossa arches from the body of the postorbital across the temporal bar along the edge of the paroccipital process (Fig. 59). These features suggest that the adductor musculature had a large volume and surface of origination for powerful operation of a stoutly proportioned lower jaw with a prominent coronoid process.

Although many ornithischians show similar attachment fossae for the adductor musculature along the margins of the laterotemporal fenestra (Fig. 97B), only heterodontosaurids incorporate the head of the quadrate within the fossa. The quadrate head is inset and lies ventral to an everted, arcuate edge formed by the squamosal and distal margin of the paroccipital process (Fig. 59). A similar everted arcuate margin, although damaged, appears to be present in *Tianyulong* and *Manidens* (Figs 21, 81B).

Other notable structures of potential relevance for reconstructing the adductor musculature include the jugal flange, the robust dorsal margin of the surangular, the thin-walled external mandibular fossa, and the everted, thickened ventral margin of angular. The jugal flange is a stout, transversely compressed pendant process that is found only in heterodontosaurids. It does not resemble the pyramidal jugal horn in *Psittacosaurus* (Sereno 2010). Low rugosities are present on the flange, which is now known in a second heterodontosaurid, *Manidens* (Fig. 81B). The presence of the jugal flange in two heterodontosaurids suggests that it may play more than a decorative role.

The robust dorsal margin of the surangular is notable for the large anterior surangular foramen and the associated impressed vessel tract, which arches posteriorly toward the posterior surangular foramen (Fig. 59). The impressed vessel tract and thickened dorsal margin of the bone suggest that the entire margin served for the insertion of a powerful jaw adductor muscle (M. adductor mandibulae externus superficialis), which is the case in extant archosaurs (Iordansky 1964; Holliday 2009).

The enlarged surangular foramen, impressed vessel tract, external mandibular fossa, and everted ventral margin of angular in *Heterodontosaurus* are also present in *Lycorhinus* (Fig. 79) and *Manidens* (Fig. 81B) and may eventually be shown to characterize other heterodontosaurids. The depression on the lower jaw here termed the external mandibular fossa is formed equally by rami of the surangular and angular posterior to the external mandibular fenestra (Fig. 59). Unlike other depressions thought to be suitable for muscle attachment, this thin-walled fossa may facilitate muscle attachment to its prominent ventral margin.

Crompton and Attridge (1986) first noticed the relatively large volume of the adductor chamber in *Heterodontosaurus* and sketched the attachment of adductor musculature to the parietal on the medial side of the supratemporal fossa (= M. adductor mandibulae externus profundus) and to the postorbital, squamosal and quadratojugal along the margins of the laterotemporal fenestra (= M. adductor mandibulae externus superficialis) (Fig. 94A).

Norman et al. (2011: 253) have reconstructed the M. adductor mandibulae externus superficialis in *Heterodontosaurus* as a “remarkable curtain of superficial adductor muscles that drape the lateral surface of the skull from the postorbital-squamosal bar to the rim of the angular on the lower jaw” (Fig. 94B). Several aspects of their reconstruction of this muscle are problematical. First, the position of this muscle sheet *lateral to the lower temporal bar* is unlike the condition in any extant archosaur; this muscle always passes medial to the lower temporal bar (when that bar is maintained) (Iordansky 1964; Holliday 2009). Second, the muscle as reconstructed courses over a sharp, everted edge along the anterior margin of the quadrate shaft (Fig. 59). Third, the lower temporal bar and thickened jugal flange are positioned between two moving muscle sheets—the medial and superficial divisions of the adductor mandibulae (Fig. 94B, C). Fourth, the usual insertion site for the adductor mandibulae externus superficialis in both crocodilians and birds on the dorsal margin of the surangular (Holliday 2009) is now replaced by the M. adductor mandibulae externus medialis (Fig. 94C).

**Figure 90. F90:**
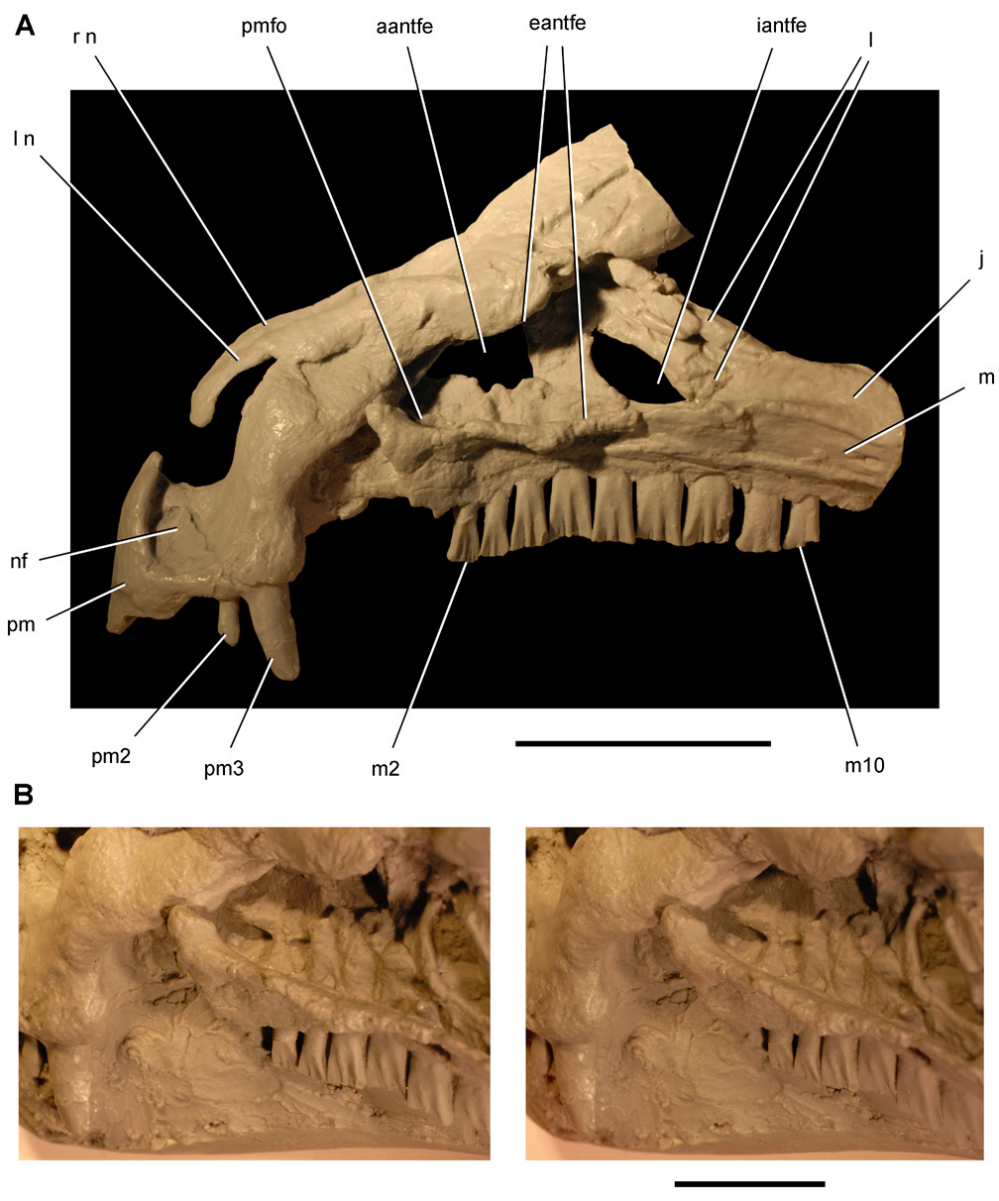
Snout and upper dentition in *Heterodontosaurus tucki*. Cast of snout and upper dentition (UCRC PVC11) from an early mold of adult skull SAM-PK-K1332 **A** Cast of snout and upper dentition in left lateral view **B** Stereopair of anterior portion of the left antorbital fossa in posterolateral view. Scale bar equals 2 cm in **A** and 1 cm in **B**. Abbreviations: ***aantfe*** accessory antorbital fenestra ***eantfe*** external antorbital fenestra ***iantfe*** internal antorbital fenestra ***j*** jugal ***l*** lacrimal or left ***m*** maxilla ***m2***, ***10***, maxillary tooth 2, 10 ***n*** nasal ***nf*** narial fossa ***pm*** premaxilla ***pm2***, ***3*** premaxillary tooth 2, 3 ***pmfo*** promaxillary fossa ***r*** right.

**Figure 91. F91:**
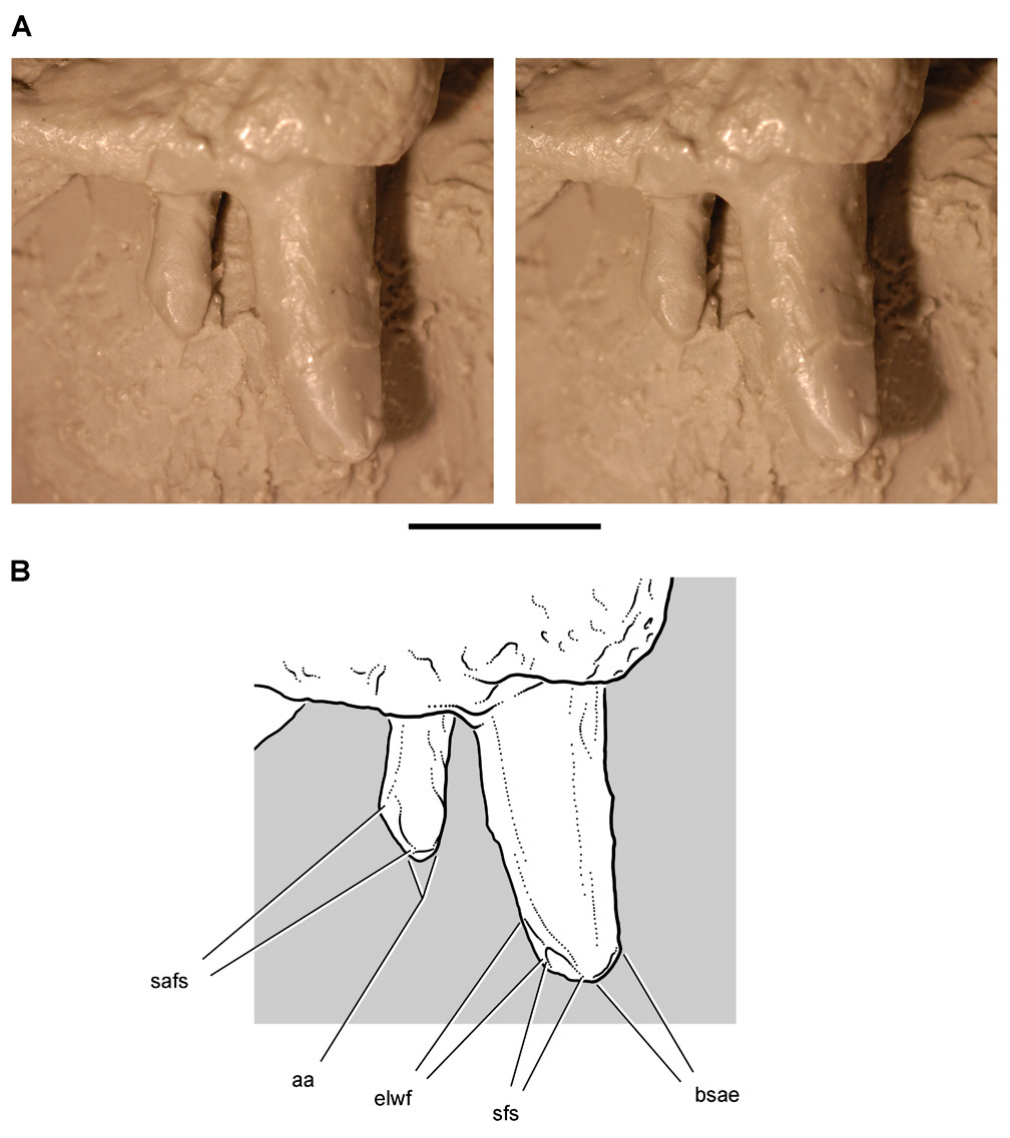
Premaxillary dentition in *Heterodontosaurus tucki*. Cast of premaxillary tooth 2 and 3 in left lateral view (UCRC PVC11) from an early mold of adult skull SAM-PK-K1332. Stereopair (**A**) and line drawing (**B**) showing spalling, abrasion, and the mesial and distal edges of ligual tooth-to-bill wear facets. Scale bar equals 5 mm. Abbreviations: ***aa*** apical abrasion ***bsae*** breakage surface with abraded edges ***elwf*** edge of lingual wear facet ***sfs*** spalled fracture surface ***safs*** spalled abraded fracture surface.

**Figure 92. F92:**
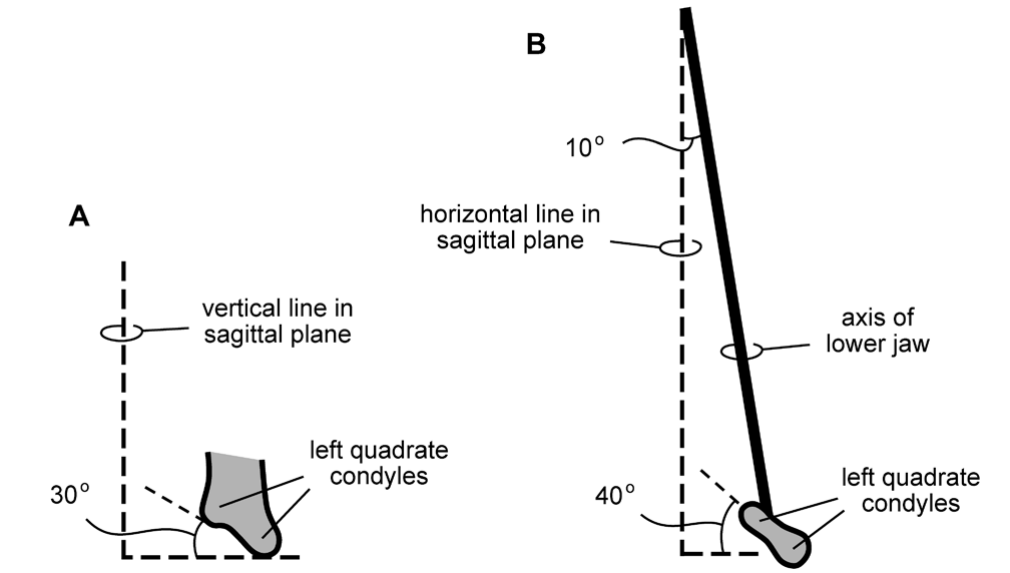
Configuration of jaw articulation and long axis of the lower jaw in *Heterodontosaurus tucki* from the Lower Jurassic Upper Elliot and Clarens formations of South Africa. Diagrammatic depiction of jaw configuration based on subadult skull AMNH 24000 and adult skull SAM-PK-K1332 **A** Left quadrate condyles in anterior view showing 30° angle from the horizontal **B** Left quadrate condyles and long axis of the lower jaw in ventral view showing 40° angle from a transverse axis and 10° divergence from the midline, respectively.

**Figure 93. F93:**
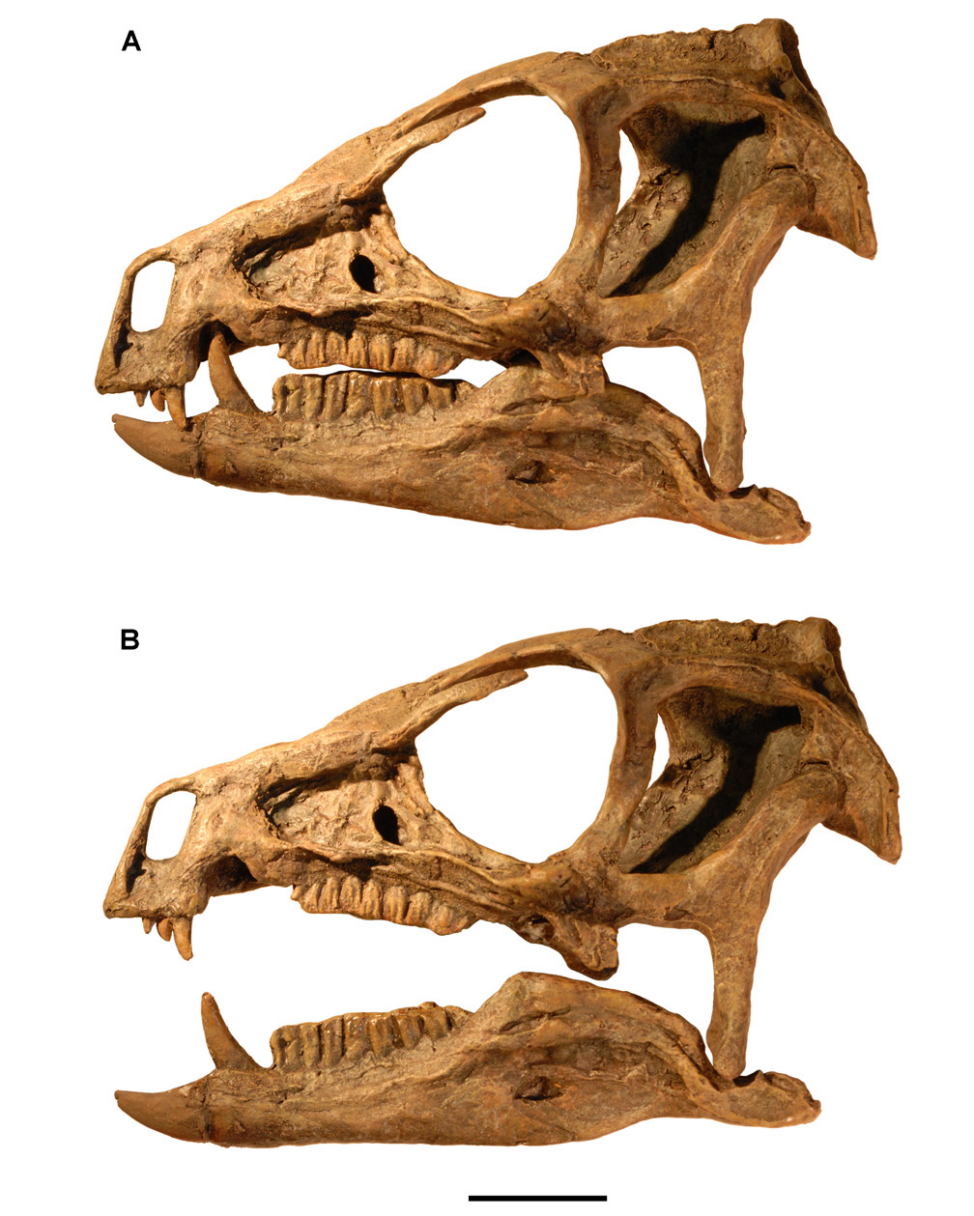
Relative position of the lower jaw in *Heterodontosaurus tucki* from the Lower Jurassic Upper Elliot and Clarens formations of South Africa. Position of the lower jaw during occlusion (**A**) and moderate gape (**B**) as seen in left lateral view of a cast of the cranium and lower jaws of adult specimen SAM-PK-K1332. Scale bar equals 2 cm.

**Figure 94. F94:**
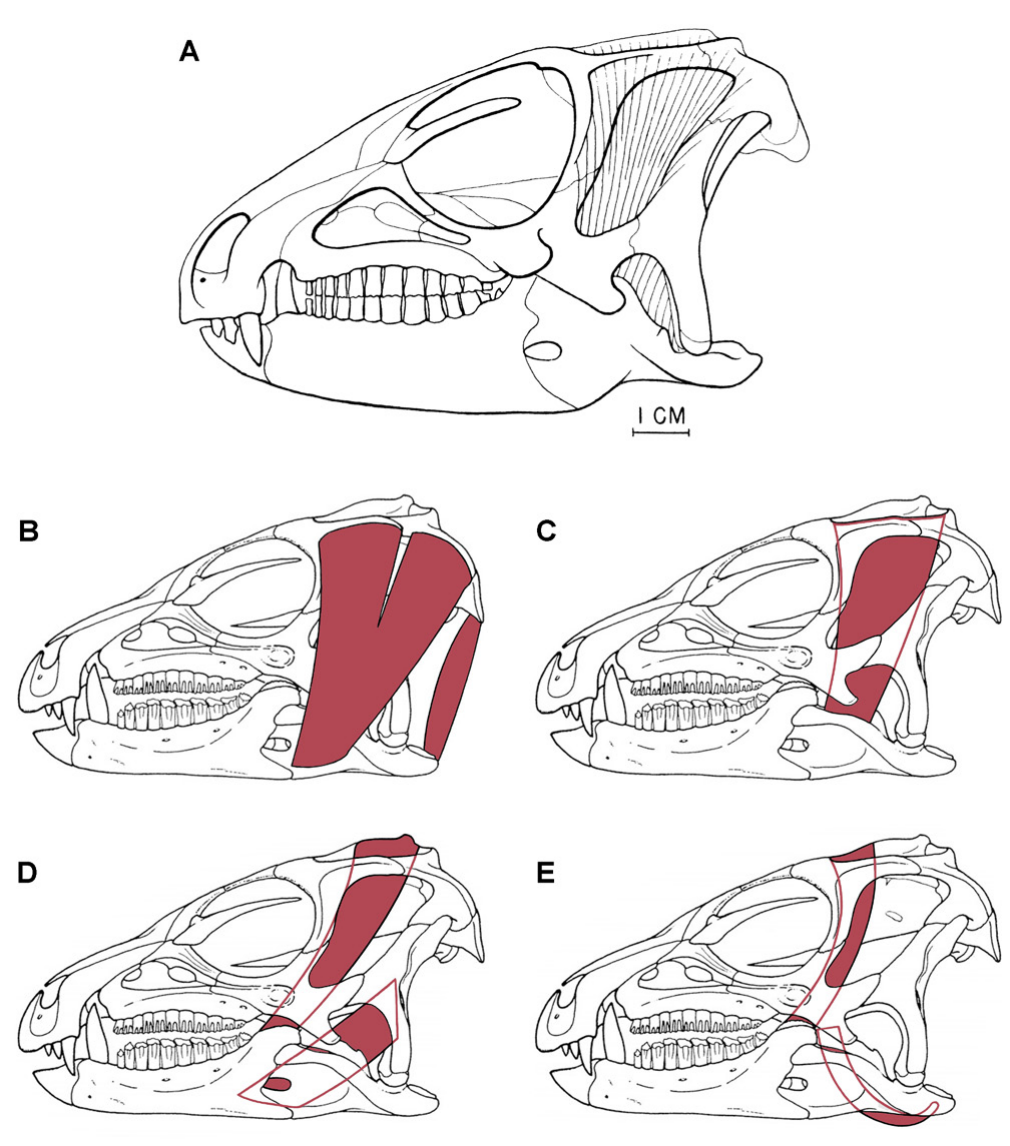
Previous jaw muscle reconstructions in *Heterodontosaurus tucki* from the Lower Jurassic Upper Elliot and Clarens formations of South Africa **A** Initial adductor muscle reconstruction (from Crompton and Attridge 1986) **B** Reconstruction of the adductor mandibulae externus superficialis (left) and the depressor mandibulae (right) (adapted from figures in Norman et al. 2011) **C** Reconstruction of the adductor mandibulae externus medialis (adapted from figures in Norman et al. 2011) **D** Reconstruction of the adductor mandibulae externus profundus (left) (misidentified as “adductor mandibulae externus posterior” in legend for Fig. 35, Norman et al. 2011) and the adductor mandibulae posterior (right) (adapted from figures in Norman et al. 2011) **E** Reconstruction of the pseudotemporalis (left) and the pterygoideus posterior (right) (adapted from figures in Norman et al. 2011).

**Figure 95. F95:**
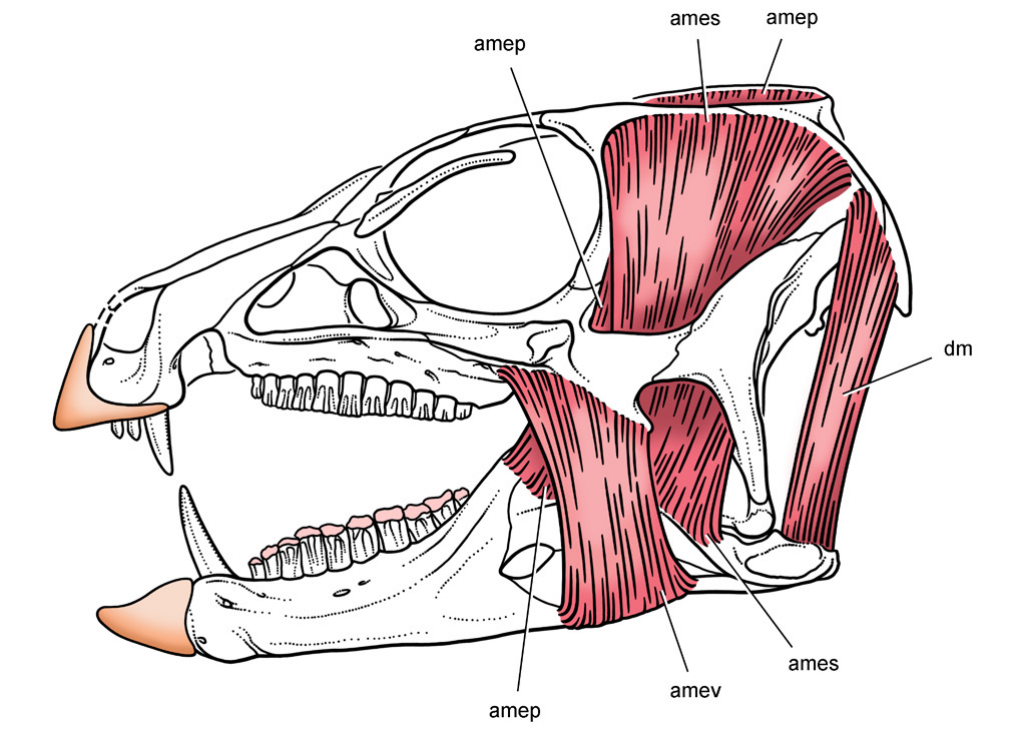
Jaw musculature and keratin sheathing in *Heterodontosaurus* from the Lower Jurassic Upper Elliot and Clarens formations of South Africa. Reconstruction of select jaw muscles and keratin sheathing of upper and lower bills supported in this study. Pink tone indicates wear facets. Abbreviations: ***amep*** adductor mandibulae externus profundus ***ames*** adductor mandibulae externus superficialis ***amev*** adductor mandibulae externus ventralis ***dm*** depressor mandibulae.

**Figure 96.**
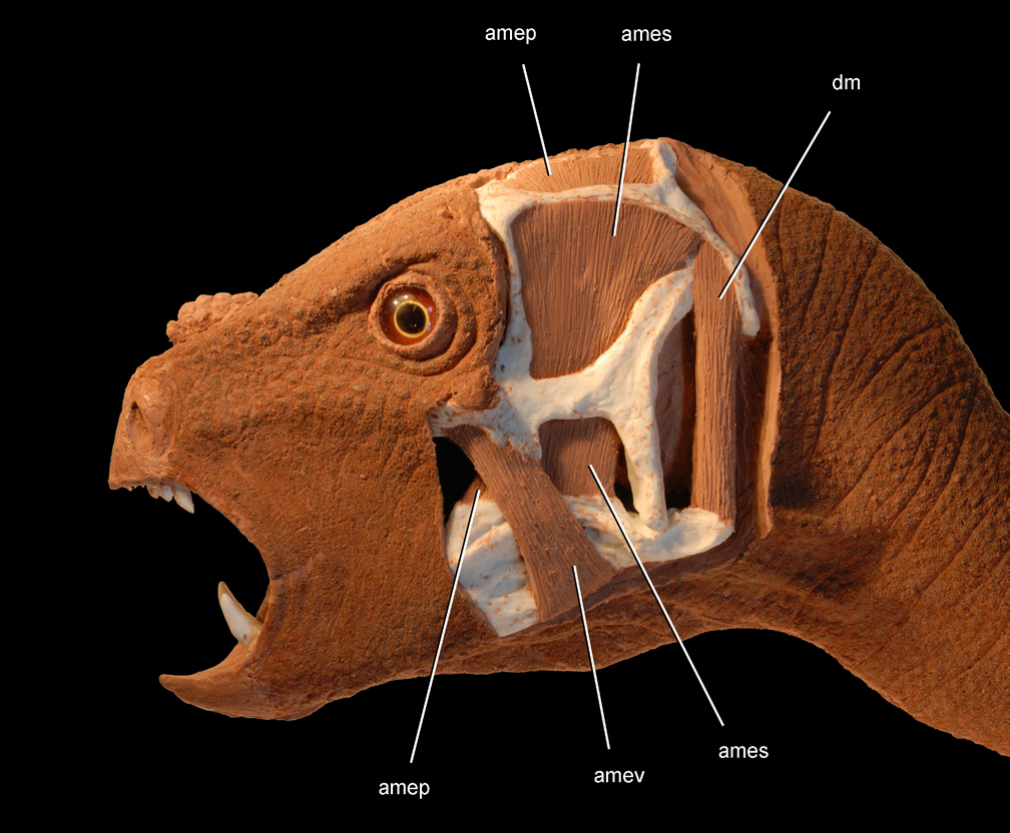
Jaw adductor musculature in *Heterodontosaurus tucki* from the Lower Jurassic Upper Elliot and Clarens formations of South Africa. Flesh head sculpting over a cast of an adult skull (SAM-PK-K1332) showing surface tissues (anterior half) and select jaw muscles (posterior half). Abbreviations: ***amep*** adductor mandibulae externus profundus ***ames*** adductor mandibulae externus superficialis ***amev*** adductor mandibulae externus ventralis ***dm*** depressor mandibulae.

**Figure 97.**
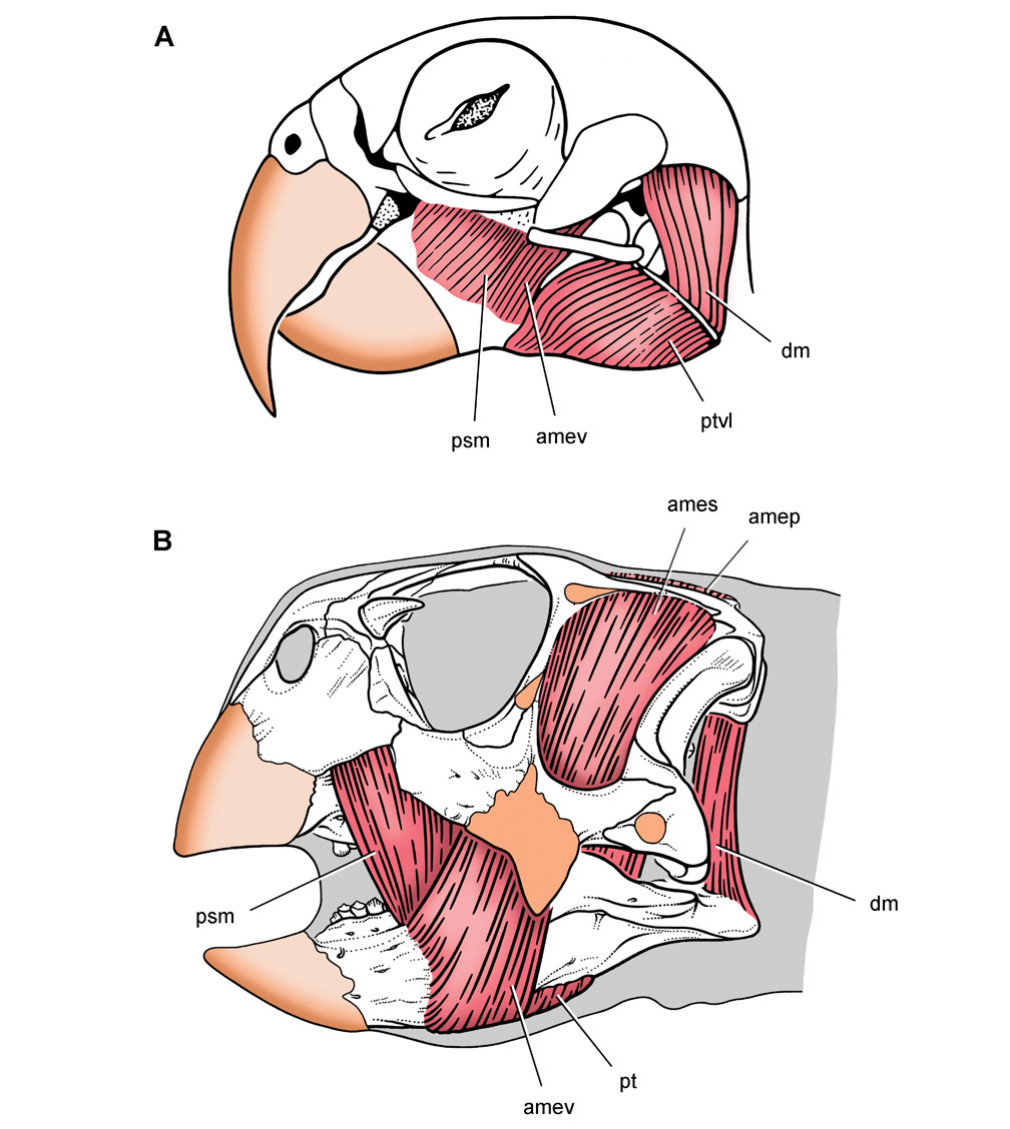
Jaw adductor musculature and keratin sheathing in a psittaciform bird and parrot-beaked dinosaur. Differentiation of facial components of the adductor muscle mass to increase bite force **A** *Pionites leucogaster* (white-bellied parrot) (from Sereno et al. 2010) **B** *Psittacosaurus gobiensis* with inferred jaw muscles and keratin sheathing (from Sereno et al. 2010). Abbreviations: ***amep*** adductor mandibulae externus profundus ***ames*** adductor mandibulae externus superficialis ***amev*** adductor mandibulae externus ventralis ***dm*** depressor mandibulae ***psm*** pseudomasseter ***pt*** pterygoideus ***ptvl*** pterygoideus ventralis lateralis.

An alternative muscle reconstruction for *Heterodontosaurus* (Figs 95, 96):

(1) Maintains the usual archosaurian insertion for the M. adductor mandibulae externus superficialis on the dorsal margin of the surangular;

(2) Positions the insertions for both the M. adductor mandibulae externus profundus and medialis as in archosaurs on the prominent coronoid process formed by the dentary and coronoid;

(3) Avoids the everted edge of the quadrate shaft by filling the posterior portion of the squamosal-paroccipital fossa with the origin of the M. depressor mandibulae;

(4) Reconstructs a separate muscle mass, the M. adductor mandibulae externus ventralis, originating on the ventral margin of the jugal flange and inserting on the everted ventral margin of the angular.

The differentiation of a M. adductor mandibulae externus ventralis, which is depicted originating on a prominent lateral crest on the maxilla and jugal flange and inserting on the raised ventral margin of the angular (Figs 95, 96, amev), is the most speculative suggestion in the reconstruction of jaw musculature proposed in this study. This masseter-like muscle is differentiated among archosaurs only in birds such as psittaciforms with enhanced musculature for powerful jaw adduction (Fig. 97A). Not only would a muscle in this position enhance jaw adduction in *Heterodontosaurus* in a similar manner, but it would also facilitate long-axis rotation of the lower jaw. A muscle in this position could rotate the lower jaw to generate the transversely concave wear facets observed in worn adult dentitions (Fig. 89B).

The other muscle in archosaurs that might insert on the swollen ventral margin of the angular is the M. pterygoideus ventralis, which originates on the posterior palate and wraps around the ventral margin of the lower jaw (Iordansky 1964; Holliday 2009). The M. pterygoideus ventralis in *Heterodontosaurus*, likewise, would extend posteroventrally from the palate to insert farther posteriorly on the angular ventral to the jaw joint (Fig. 94E). The everted margin of the angular, however, is located ventral to the coronoid process, anterior to the usual insertion of the M. pterygoideus ventralis in crocodilians and birds (Holliday 2009). Thus there is no obvious muscle to insert on the swollen ventral margin of the angular, which would be a suitable site for a M. adductor mandibulae externus ventralis.

The other proposed function of the jugal flange is that it formed the later wall of a bony slot, into which slid the surangular when the jaws are closed (Norman et al. 2011). This configuration is well illustrated in the holotypic skull of *Heterodontosaurus* when the jaws are closed (SAM-PK-K337) (Fig. 91A). The jugal flange is positioned lateral to the dorsal margin of the surangular; and the mandibular flange is positioned more anteriorly in the center of the adductor fossa of the lower jaw (Norman et al. 2011: Figs 1, 2). The surangular is free of both flanges when the jaws are opened (Fig. 93B). In *Manidens*, however, the jugal and pterygoid are not positioned as a slot for the surangular even when the jaws are closed, because of the elevated position of the jugal flange and lower profile of the surangular (Fig. 81B). This suggests that the jugal flange did not function as a bony guide for the lower jaw in heterodontosaurids. Because jaw function in adult *Heterodontosaurus* clearly requires significant (2 mm) transverse displacement of the lower tooth row during occlusion, the functional purpose of a rigid vertical guide for the lower jaw is not clear.

### Inferred diet

**Previous hypotheses.** Heterodontosaurids, like other ornithischians, long have been assumed to be herbivores. As a result, few remarks were made regarding the diet of these early ornithischians when excellent cranial and postcranial material of *Heterodontosaurus tucki* came to light (Crompton and Charig 1962; Santa Luca et al. 1976). The reduced caniniform teeth of *Abrictosaurus consors*, another early and important heterodontosaurid discovery (Thulborn (1974), drew attention to the possibility of sexual dimorphism in the caniniform dentition and possible use of these teeth in agonistic and defensive behavior (Thulborn 1974; Molnar 1977). The aberrant style of tooth replacement in heterodontosaurids (no replacement foramina, possible cessation of replacement with age) and evidence of extensive tooth-to-tooth wear, however, served to underscore the interpretation of heterodontosaurids as specialized herbivores (Thulborn 1974, 1978).

More recently, Barrett (2000: 64) suggested that in heterodontosaurids “the shapes of the premaxillary teeth and the dentary caniniform tooth suggest that facultative omnivory may have been a possibility”. He inferred from the serrate carinae of the caniniform teeth that “they were used during feeding”. Butler et al. (2008) suggested that the early appearance of caniniform teeth during growth in heterodontosaurids and the lack of evidence for sexual dimorphism among heterodontosaurid specimens favored “alternative functions for the caniniforms, such as defense and occasional omnivory”. Porro et al. (2011: 365) speculated “*Heterodontosaurus* may have fed upon tough, fibrous vegetation, while *Abrictosaurus* and ‘fabrosaurids’ selected more nutritious, less abrasive plants or engaged more frequently in omnivory”.

Norman et al. (2011: 231) suggested that the caniniform teeth in heterodontosaurids were unlikely to have been used for “cropping or rooting for vegetation” or “intraspecific display”, because of the absence of wear and sexual dimorphism, respectively. However, as presented above, there is evidence for sustained tooth-to-bill shearing in the premaxillary caniniform teeth and evidence for breakage and apical abrasion in both premaxillary and dentary caniniform teeth. Intraspecific display structures in non-avian dinosaurs, in addition, only rarely show sexual dimorphism comparable to that in mammals. The horns and crests in neoceratopsians and hadrosaurids, for example, usually show little evidence of sexual dimorphism, and so it is potentially presumptive to assume that heterodontosaurid caniniform teeth had no intraspecific agonistic function.

Norman et al. (2011: 231) highlighted the relatively long forelimb and large, grasping manus with elongate penultimate phalanges and trenchant claws in *Heterodontosaurus*, remarking “The combination of cursorial hindlimbs, raptorial forelimbs, and puncturing caniniforms is suggestive of an ability to catch and consume small prey”, raid nests or feed on “carrion”. Yet, why would presumptive “opportunistic predators” greatly reduce one of these features? *Abrictosaurus*, for example, lacks enlarged caniniform teeth (Fig. 35), and *Tianyulong* has diminutive forelimbs (Fig. 30). Many herbivores have evolved cursorial proportions for escape or energetically efficient locomotion. Large, sharp-clawed, grasping hands, in addition, would also be useful for rooting behaviors or for selective herbivory, as in the case in coelurosaurian theropods that have shifted in diet from carnivory to herbivory (ornithomimosaurs, oviraptorosaurs, therizinosauroids, basal avians; Zanno et al. 2009). In ornithomimids, the ungual of manual digit I remains trenchant, even when some manual unguals are straightened for more effective scratch-digging. Sharp, recurved unguals, in these cases, represent a retained ancestral condition. Likewise, if heterodontosaurids are basal ornithischians, the similarity in the form of their forelimbs to that of basal saurischians, such as *Eoraptor* and *Eodromaeus* (Martinez et al. 2011), may be interpreted similarly—as a retained primitive condition.

**Figure 98.**
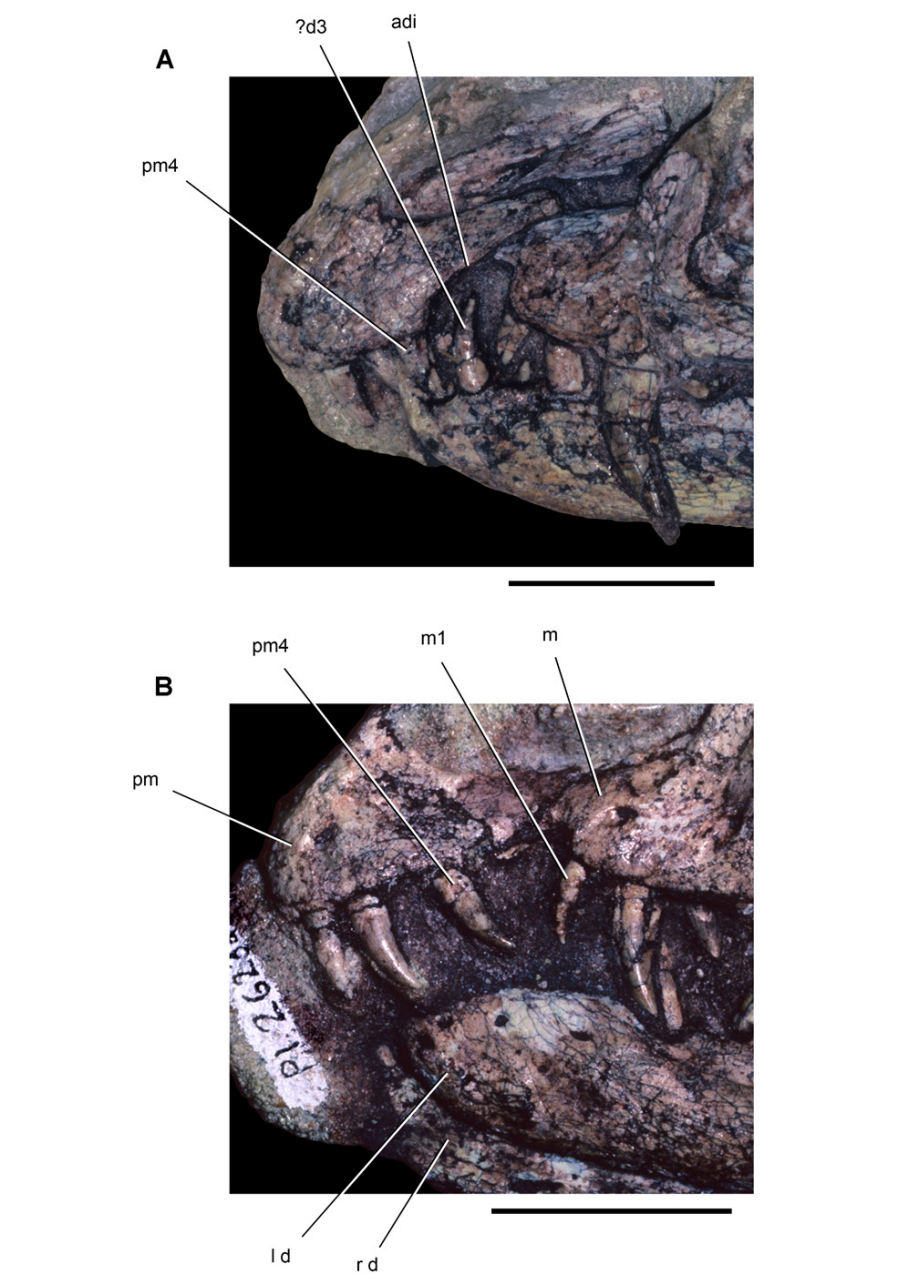
Snout and anterior dentition of dinosaurian carnivores. Snout and anterior dentition in left lateral view of coelophysoid theropods **A** *Coelophysis bauri* (MCZ 4327) from the Late Triassic **B** “*Syntarsus*” *kayentakatae* (MNA V2623) from the Early Jurassic. Scale bars equal 2 cm. Abbreviations: ***adi*** arched diastema ***d*** dentary ***d3*** dentary tooth 3 ***l*** left ***m*** maxilla ***m1*** maxillary tooth 1 ***pm*** premaxilla ***pm4*** maxillary tooth 4 ***r*** right.

Thulborn (1974) and others (Norman et al. 2011) have drawn an apt parallel between the caniniform teeth in heterodontosaurids and tayassuid suiforms (peccaries), which comprise a closer analog than the hinged upper canine or tusk in the chevrotain and various deer (Aitchison 1946). In heterodontosaurids and peccaries, the caniniform teeth are vertical rather than splayed as in suid suiforms, their edges are sharp, and the tip of the dentary caniniform tooth is sequestered in a pocket when the jaws are closed (Herring 1972). Peccary canines are used in intraspecific biting and defense. In addition, they are not sexually dimorphic, because they function less in visual display in contrast to suid suiforms. Peccaries are herbivores that root for food and consume a wide variety of foodstuffs (fruit, roots, bulbs, grass, acorns, pine nuts, and thistles; Corn and Warren 1985) and may represent the closest living analogs to heterodontosaurids.

**Evidence from dental structure and function.** Three lines of evidence in dental structure and function favor a predominantly, or exclusively, herbivorous diet.

**Figure 99.**
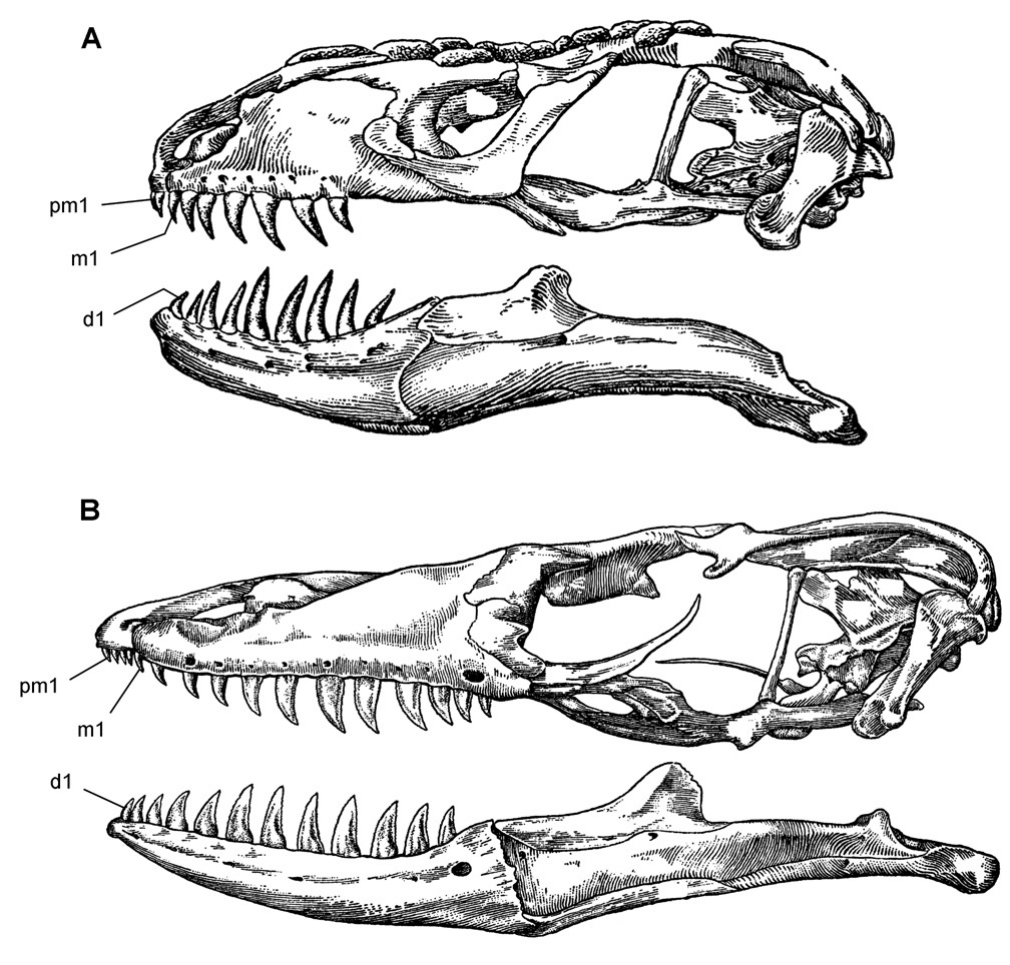
Skulls of extant lepidosaurian carnivores. Skulls in left lateral view (from McDowell and Bogert 1954) **A** *Heloderma horridum* (beaded lizard) **B** *Varanus varius* (lace monitor). Abbreviations: ***d1*** dentary tooth 1 ***m1*** maxillary tooth 1 ***pm1*** premaxillary tooth 1.

1. *Tooth-to-tooth shearing wear facets in the cheek dentition*. Wear facets from tooth-to-tooth occlusion are present and often very well developed in heterodontosaurids, as preserved in *Pisanosaurus*, *Echinodon*, *Lycorhinus*, *Pegomastax*, *Abrictosaurus*, and *Heterodontosaurus*. Tooth-to-tooth oblique shearing occlusion is a sophisticated ornithischian adaptation for processing plant material. Only a few specialized crocodylomorphs and squamates with propalinal jaw movement have evolved tooth-to-tooth shear for processing plant matter.

2. *Extent of tooth wear unrelated to presence/size of caniniform teeth*. Increase in the extent of wear or the presence of asymmetrical enamel as in *Heterodontosaurus* does not entail a decrease in the size of caniniform teeth. Caniniform teeth of equal size are present in *Tianyulong*, which has relatively simple cheek tooth crowns. Dietary function thus may have been a secondary role for the caniniform teeth.

3. *Edentulous anterior end of upper and lower jaws*. The ends of both upper and lower jaws lack teeth and in life were covered by a keratinous bill. By contrast, the teeth at the anterior end of the jaws that are critical for prey capture are never lost in insectivorous or carnivorous reptiles (Figs 98, 99).

4. *Variable caniniform size and position*. Pm3 has a relatively small bulbous crown in *Echinodon*, a small more columnar crown in *Abrictosaurus*, and a considerably larger, laterally compressed and probably serrate crown in *Tianyulong*, *Lycorhinus* and *Heterodontosaurus* (Figs 19B, 23, 35, 59, 81B). The dentary caniniform is large, laterally compressed and probably serrate in all heterodontosaurids except *Abrictosaurus* (Fig. 35), where the rounded crown is even smaller than the premaxillary crowns (Fig. 35). All heterodontosaurids except *Echinodon* have the upper caniniform anterior in position to the lower caniniform (Fig. 59), opposite the relation of the caniniform in most mammals; *Echinodon*, in contrast, has a moderate-sized, smooth edged caniniform crown at the anterior end of the maxilla (Fig. 19B). Thus, there is considerable variation in the size and position of the caniniform teeth in heterodontosaurids, which does not lend support to a single, specific dietary function. Parallel development and variability in pachycephalosaurid premaxillary and dentary caniniform teeth, likewise, is more easily explained by hypotheses invoking the primacy of agonistic functions.

5. *Caniniform occlusion and sequestering*. The caniniform teeth in heterodontosaurids do not occlude or slice in close coordination, and the tip of the dentary caniniform is sequestered within an invaginated pocket in the upper jaw when the jaws are closed (Fig. 93A). In *Heterodontosaurus* upper and lower caniniform teeth are positioned in opposition with a substantial gape but move apart with jaw closure. The lack of aligned movement and the sequestered tip of the largest caniniform tooth may not be the most effective configuration for prey capture or puncturing. Theropods with an arched diastema for a caniniform dentary tooth, in contrast, usually have opposing premaxillary and maxillary crowns that project toward the caniniform tooth, which is not sequestered upon jaw closing (Fig. 98).

**Evidence from jaw structure and function.** Four lines of evidence in jaw structure and function favor a predominantly, or exclusively, herbivorous diet.

**Figure 100:**
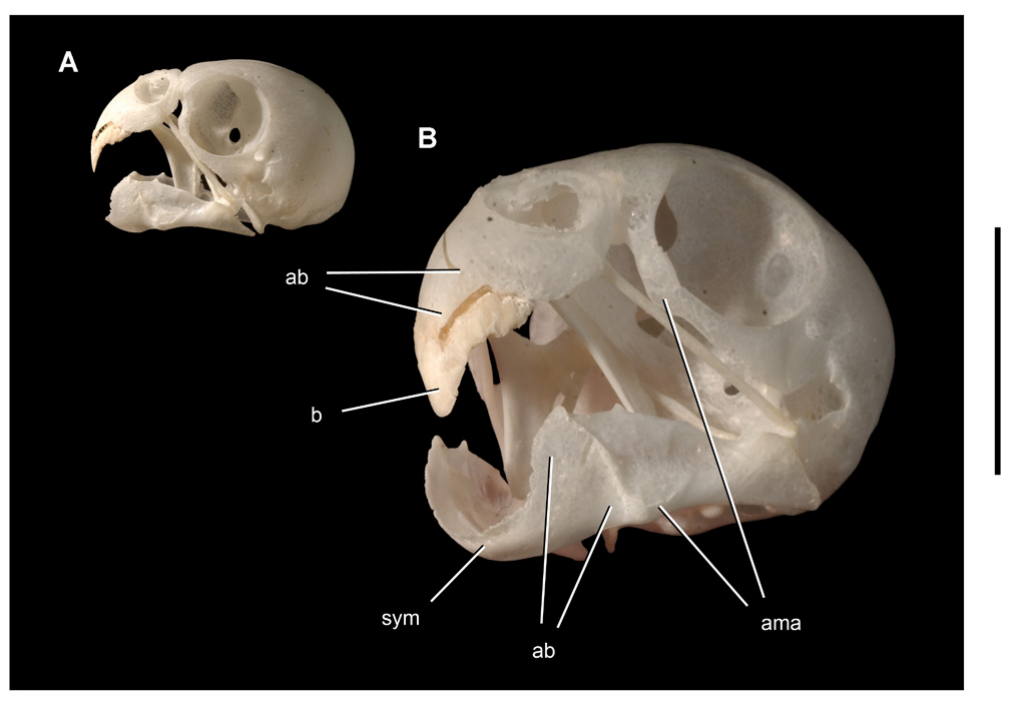
**.** Bone and keratinous sheath in the upper bill of a parrot. Skull of the budgerigar *Melopsittacus undulatus* (UCRC R1) with keratinous sheath of upper bill **A** Lateral view **B** Magnified anterolateral view. Scale bar equals 1cm in **B**. Abbreviations: ***ab*** attachment platform for bill ***ama*** area of muscle attachment ***b*** bill ***sym*** symphysis.

1. *Bills cropping in form*. Upper and lower bills in heterodontosaurids appear to be cropping, rather than puncturing or raptorial, in form. The premaxillary and dentary platforms usually adumbrate the form of the keratinous bill (Fig. 100). Carnivorous turtles and birds typically have recurved bills for prey capture and/or the puncturing and tearing of flesh. Upper and lower bill platforms in heterodontosaurids, in contrast, have linear cropping margins that generate planar wear facets on premaxillary crowns (Fig. 76).

2. *Jaw articulation ventrally offset*. Advanced heterodontosaurids have lowered the jaw articulation relative to the occlusal axis. As well documented among mammalian herbivores, displacement of the jaw joint relative to the occlusal axis equalizes pressure along the tooth row and enhances the mechanical advantage of masseter-like muscles (Greaves 1980).

3. *Cheeks well developed*. Herbivores, not insectivores or carnivores in general, are known for extensive oral processing of foodstuffs, and this has been the long favored hypothesis for the vascularized buccal emargination in ornithischian dinosaurs (Galton 1973). The buccal emargination is deep and well vascularized in all heterodontosaurids.

4. *Enhanced adductor musculature*. In *Heterodontosaurus* and other heterodontosaurids (when preserved), the adductor chamber is spacious, the peripheral attachment sites for adductor musculature are enhanced (inset supratemporal fossa, sagittal crest, extensive fossae on upper border of the laterotemporal fenestra), the mandibular ramus that anchors the pterygoideus musculature is elongate, and the coronoid process on the dentary and dentary ramus are robust. These features, particularly when expressed in dinosaurs of small body size, favor herbivory with significant oral processing of plant matter rather than omnivory or carnivory.

**Figure 101.**
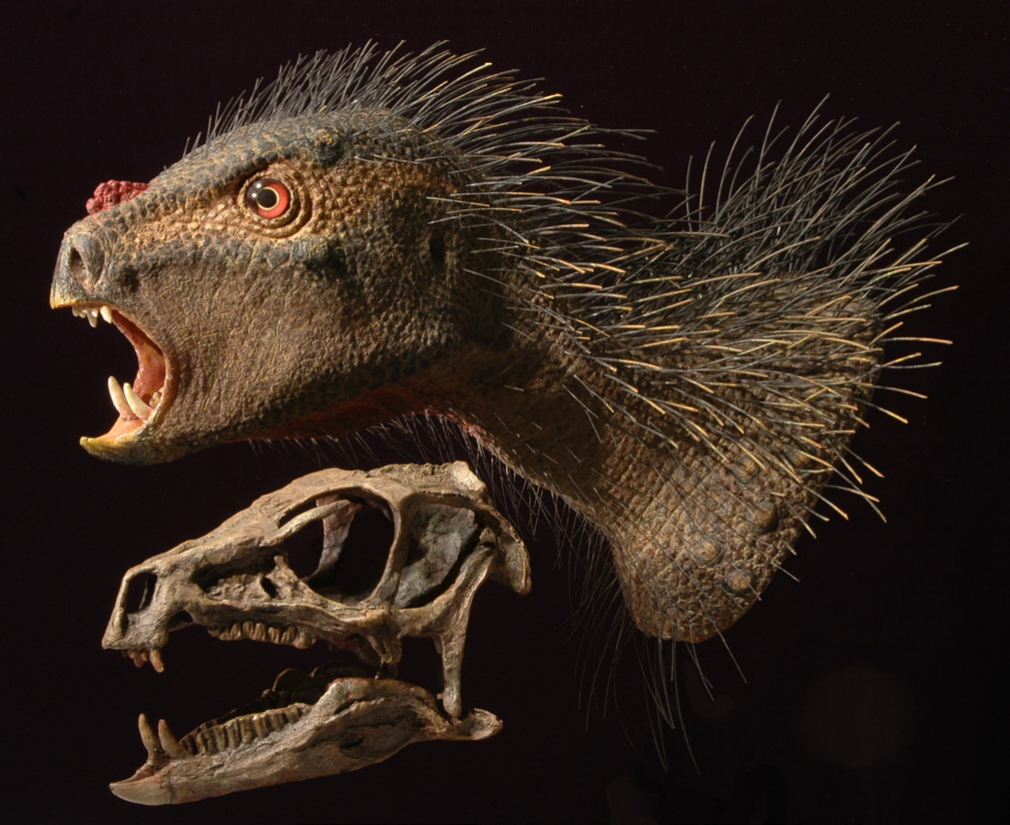
Flesh reconstruction of the head and neck of *Heterodontosaurus tucki* from the Lower Jurassic Upper Elliot and Clarens formations of South Africa. Flesh head and neck and skull reconstructions with similar gape. The flesh reconstruction was modeled over a skull cast with skin texture based on bone surface details in the skull of *Heterodontosaurus tucki* (SAM-PK-K1332) and bristles based on those preserved in *Tianyulong confuciusi* (STMN 26-3). Reconstructions by Tyler Keillor.

### Phylogenetic inferences

**Figure 102.**
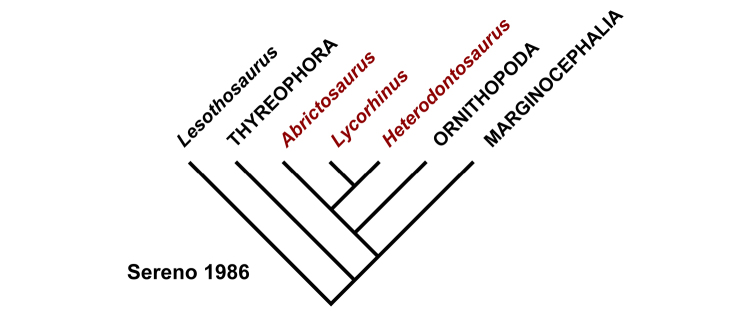
Initial phylogenetic hypothesis for heterodontosaurid relationships. Qualitative parsimony analysis by Sereno (1986) with Heterodontosauridae as the sister taxon to Euornithopoda (= Ornithopoda) and with *Abrictosaurus* basal to *Lycorhinus* and *Heterodontosaurus* among heterodontosaurids. Some terminal taxa in the original analysis are collapsed into suprageneric taxa; red text indicates heterodontosaurid taxa.

**Figure 103.**
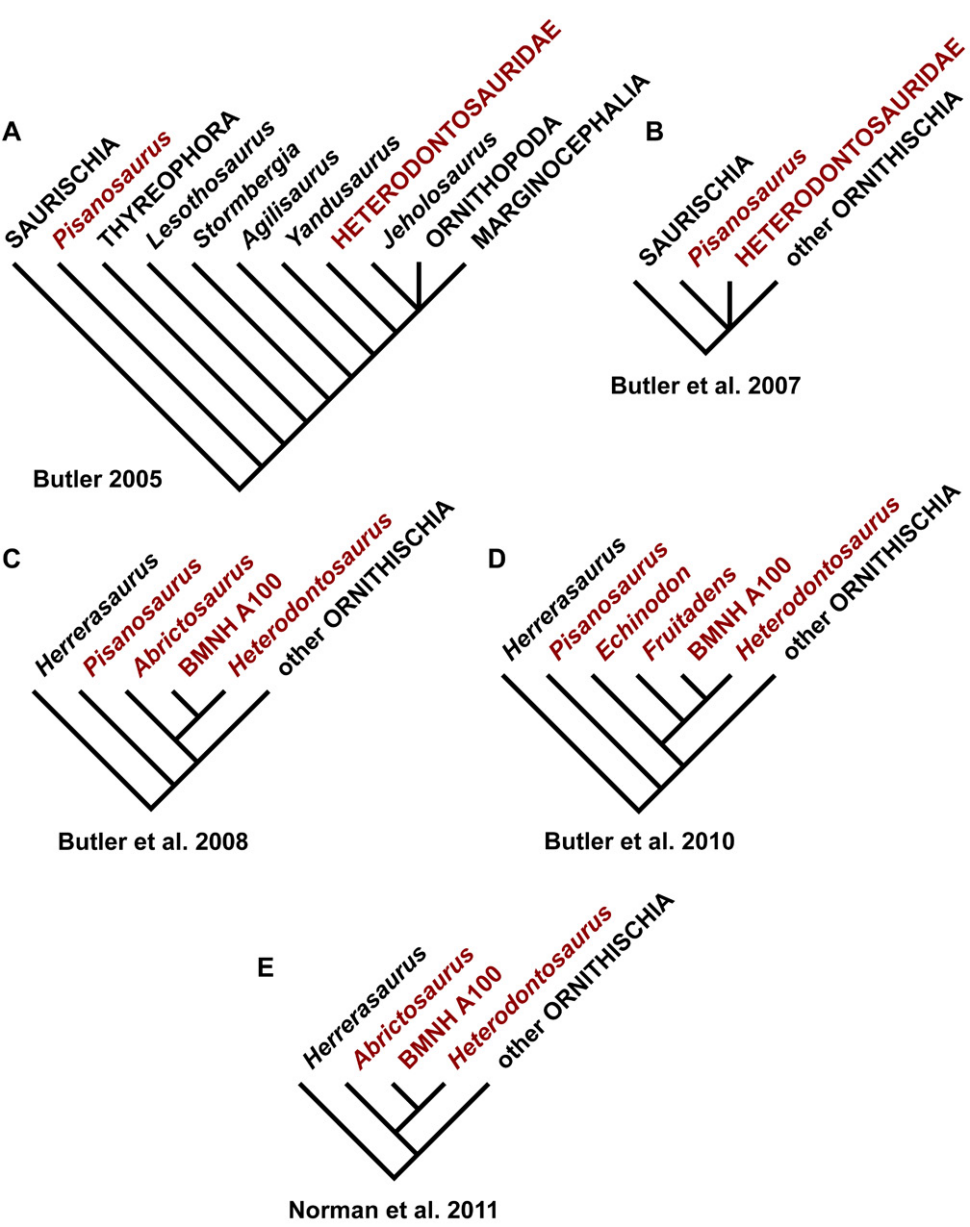
Phylogenetic hypotheses for heterodontosaurids by Butler and colleagues. **A** Heterodontosaurids with the exception of *Pisanosaurus* allied with euornithopods (after Butler 2005) **B** Heterodontosaurids and *Pisanosaurus* as basal ornithischians (after Butler et al. 2007) **C** Heterodontosaurids and *Pisanosaurus* as basal ornithischians with *Lycorhinus* (NHMUK RU A100, formerly BMNH A100) closer to *Heterodontosaurus* than *Abrictosaurus* (after Butler et al. 2008) **D** Heterodontosaurid relationships including *Echinodon* and *Fruitadens* as basal taxa (after Butler et al. 2009) **E** Heterodontosaurid relationships as in Butler et al. (2008) (after Norman et al. 2011). Some terminal taxa in the original analyses are collapsed into suprageneric taxa; red text indicates heterodontosaurid taxa; “BMNH A100” is now catalogued as NHMUK RU A100.

**The phylogenetic position of heterodontosaurids among ornithischians.** The phylogenetic position of heterodontosaurids among ornithischian dinosaurs is an important but, as yet, poorly resolved question. Until recently, cladistic analysis placed heterodontosaurids within Ornithischia in a position more advanced than *Lesothosaurus* and the armored thyreophorans, as the sister group to either Euornithopoda (*sensu* Sereno 2005b; Sereno 1986), Marginocephalia (You et al. 2003; Xu et al. 2006), or Euornithopoda + Marginocephalia (Butler 2005) (Figs 102, 103A). These assessments are based almost entirely on *Heterodontosaurus tucki*, which still dominates information on heterodontosaurids (Santa Luca et al. 1976; Santa Luca 1980; Norman et al. 2011). None of these hypotheses offers substantial character support for the preferred sister group relationship, although the union of heterodontosaurids with Marginocephalia is perhaps the weakest (based on a few homoplastic, poorly defined characters).

Heterodontosaurids with primitive subtriangular crowns were known to exist on northern landmasses, but so little was known about their morphology that they did not impact the aforementioned phylogenetic hypotheses. The heterodontosaurid status of *Echinodon* from southern England, for example, was proposed (Sereno 1997), but that re-identification was questioned (Norman and Barrett 2002). A small heterodontosaurid also was discovered in the Kayenta Formation of western North America with postcranial bones resembling those of *Heterodontosaurus* (Attridge et al. 1985; Sereno 1986), but that specimen awaits description Fig. 9B).

**Figure 104.**
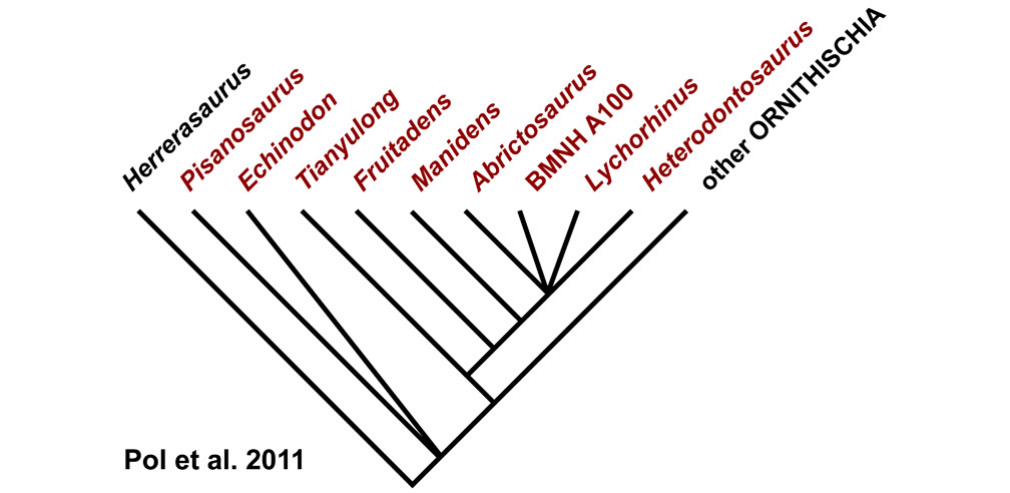
More inclusive phylogenetic hypothesis for heterodontosaurids. Parsimony analysis of Pol et al. (2011) based on character data modified from Butler et al. (2009). The analysis includes the South American genus *Manidens* and the Asian genus *Tianyulong*. Some terminal taxa in the original analysis are collapsed into suprageneric taxa; red text indicates heterodontosaurid taxa; “BMNH A100” is now catalogued as NHMUK RU A100.

More recently, heterodontosaurids have been reinterpreted as basalmost ornithischians (Butler et al. 2007, 2008) (Figs 103B-E, 104). Basal saurischian outgroups (*Saturnalia*, *Eoraptor*, *Herrerasaurus*, *Eodromaeus*) have grasping, sharp-clawed hands similar to those in heterodontosaurids (*Tianyulong*, *Abrictosaurus*, *Heterodontosaurus*). When these outgroups were incorporated in the most recent analysis of basal dinosaurs (Martinez et al. 2011), however, heterodontosaurids were nested above *Lesothosaurus* within Ornithischia, albeit by only a slim margin of character evidence.

Recent discovery of several skeletons of the Chinese heterodontosaurid *Tianyulong* (Zheng et al. 2009), which has simple subtriangular crowns, has added an important new taxon to the debate (Fig. 30). Some neornithischian features cited previously to position *Heterodontosaurus* within Neornithischia are present in *Tianyulong*, such as the open acetabulum and laterally prominent ischial peduncle on the ilium (Sereno 1986, 1999), whereas others are clearly absent, such the ventral displacement of the jaw joint relative to the tooth rows (Fig. 9C).

Comparison of character data is key to resolving the position of heterodontosaurids among ornithischians—or at least isolating the characters and/or character state scores that support alternative hypotheses (Sereno 2009). Although Norman et al. (2011) initiated qualitative comparison of select characters, quantitative comparison is needed to reveal the percentage of shared character data and character state scores between analyses. Although support for alternative positions of heterodontosaurids within Ornithischia is uniformly weak (basal nodes rarely have a decay index greater than two), there are stark differences in character data between analyses. Butler’s initial analysis involved 73 characters derived from a literature survey scored in 23 ornithischian ingroups (Butler 2005). A later analysis (Butler et al. 2007) involved twice as many characters (150 characters) in roughly the same ingroups. The most recent analysis involved 220 characters in roughly twice as many ingroups (43 ingroups) (Butler et al. 2008) and was used with slight modification elsewhere (Butler et al. 2010; Zheng et al. 2009; Norman et al. 2011; Pol et al. 2011). The analysis of Martinez et al. (2011), by contrast, involved 139 characters in 16 basal dinosaurian taxa and included *Eoraptor* and *Eodromaeus* as basal saurischian outgroups. These datasets require quantitative comparison, which is beyond the scope of the present study.

**Heterodontosaurid monophyly.** The 13 features listed below are present in *Heterodontosaurus tucki* and at least one other heterodontosaurid species and have the potential to stand as synapomorphies in phylogenetic context, using either euornithopods or basal saurischians as immediate outgroups (Sereno 1999; Butler et al. 2007; Martinez et al. 2011; Appendix I). Several of these synapomorphies are known only in *Heterodontosaurus* and *Manidens*, the skull of which is better known than other heterodontosaurids. Additional cranial information on *Tianyulong* and the Kayenta heterodontosaurid will likely clarify the status of these characters as pertaining to all heterodontosaurids or only a subset.

Several features previously considered to characterize heterodontosaurids have been omitted, because they also occur in basal saurischians and thus may constitute dinosaurian symplesiomorphies. These include the absence of replacement foramina and features of manus, such as metacarpals 1-3 with block-like bases and dorsal extensor pits for hyperextension and trenchant manual unguals with prominent flexor processes (Sereno, 1986; Martinez et al. 2011). Other features listed previously as heterodontosaurid synapomorphies (Sereno 1986; Butler et al. 2010, 2012) are omitted, because new evidence shows a more complicated distribution among heterodontosaurids. The ventrally placed jaw joint and proportionately long manus in *Heterodontosaurus* and *Abrictosaurus* (Sereno 1986), for example, do not characterize *Tianyulong* (Figs 23, 30, Table 3).

Butler et al. (2010: 6; 2012: 3) recently listed eight features in a diagnosis of the family, four of which overlap those listed below (apomorphies i, iii, xi, xiii). The other four (constriction between humeral head and medial tubercle, rod-shaped fourth trochanter, astragalocalcaneal fusion, proximal pedal phalanges with distal extensor pit) are problematic as heterodontosaurid autapomorphies, because of variation among included taxa (astragalocalcaneal fusion), presence in other basal dinosaurs (proximal pedal phalanges with distal extensor pit, as in *Eoraptor* and *Eodromaeus*), or validity on morphologicalal grounds (rod-shaped fourth trochanter). The rodlike shape of the fourth trochanter may have been based largely on the condition in *Heterodontosaurus* as figured by Santa Luca (1980: fig. 18B). In this case, the lower edge of the trochanter is covered by matrix. A fully exposed heterodontosaurid fourth trochanter probably does not differ in form from that seen in other small ornithischians (Fig. 37C).

At present heterodontosaurid synapomorphies reside almost entirely in the skull and most of these are concerned with the number and form of the anterior teeth, the form of the loosely attached predentary, and the stout proportions of the dentary. There is only one postcranial synapomorphy listed below, the reduced shaft and distal end of the fibula. This synapomorphy is questionable if *Pisanosaurus* is properly regarded as a heterodontosaurid. In *Pisanosaurus*, the shaft and distal end of the fibula are not reduced (Fig. 7A). In other heterodontosaurids, in contrast, the shaft and distal end are very slender (less than 25% of the transverse width of adjacent parts of the tibia), as preserved in *Fruitadens*, *Tianyulong*, *Abrictosaurus*, *Heterodontosaurus*, and the Kayenta heterodontosaurid.

The present study makes a much stronger case that *Echinodon* as a heterodontosaurid, as the limited material available for this genus exhibits 5 of the 12 dental and cranial synapomorphies listed below (i-iii, xi, xii). In *Echinodon*, the dentary caniniform tooth and wedge-shaped form of the predentary are inferred from the large second alveolus and the rounded anterior end of the dentary, respectively. The following list of potential heterodontosaurid synapomorphies are discussed in more detail in Appendix I where their distribution among heterodontosaurids and outgroups is given (characters 1, 2, 4, 13–16, 18–21, 24, 30, respectively):

(i) Three or fewer premaxillary teeth (Figs 19B, 23, 35, 59).

(ii) Premaxillary teeth increase in size distally (Figs 19B, 35, 59, 80).

(iii) Dentary caniniform tooth associated with an arched premaxilla-maxilla diastema Figs 19B, 35, 59, 80).

(iv) Nasal fossa, dorsomedian with rounded lateral margins (Figs 90, 101).

(v) Jugal flange, ventral embayment of jugal-quadratojugal embayment. (Figs 59, 81B).

(vi) Jugal horn below orbit, laterally directed and dorsoventrally compressed (Figs 59, 81B).

(vii) Postorbital body, arcuate fossa with raised anterior rim (Figs 59, 81B).

(viii) Quadrate head included within laterotemporal fossa (Figs 59, 81B).

(ix) Quadrate condyle, articular surface ventrolaterally inclined at approximately 30° (Figs 59, 81B).

(x) Quadratojugal T-shaped (Figs 59, 81B).

(xi) Predentary processes (lateral, ventral) rudimentary (Figs 59, 60B, 61B).

(xii) Dentary ramus stoutly proportioned, substantial depth at mid ramus compared to length (Figs 35, 59, 81B, 87).

(xiii) Fibular mid-shaft and distal end reduced (Figs 30, 37C, 69, 70).

**Heterodontosaurid phylogeny, previous work.** The first cladistic interpretation of heterodontosaurid interrelationships positioned *Abrictosaurus* basal to *Lycorhinus* + *Heterodontosaurus*, the latter united on the presence of two characters, the presence of premaxillary and dentary caniniform teeth (Sereno 1986; Fig. 102). Subsequent work on heterodontosaurid materials pertaining to these and other taxa does not confirm this weakly supported arrangement.

Butler et al. (2008) added *Echinodon* and “BMNH A100” (now NHMUK RU A100) to the three aforementioned heterodontosaurids (*Lycorhinus*, *Abrictosaurus*, *Heterodontosaurus*) and 37 other ornithischian ingroups in an analysis of 221 characters. Reanalysis of 218 of these characters (3 are uninformative) confirms their result; heterodontosaurids join a basal polytomy at the base of Ornithischia in a strict consensus of the many minimum length trees. Resolution of heterodontosaurid monophyly or relationships among heterodontosaurids was possible only when *Echinodon*, *Lycorhinus* and three other poorly known ornithischians (*Zephyrosaurus*, *Talenkauen*, *Yandusaurus*) were removed from the analysis (Fig. 103C). With these poorly known taxa removed, there are 27 minimum length trees (468 steps) with heterodontosaurid interrelationships resembling that in Sereno (1986): “BMNH A100” is more closely related to *Heterodontosaurus* than *Abrictosaurus* (Butler et al. 2007: fig. 4, node 4; Fig. 103C). In Sereno (1986) and the present study, “BMNH A100” is referred to *Lycorhinus angustidens*, and so the proposed relationships are the same (Figs 102, 103C). *Pisanosaurus* was positioned outside all other ornithischians.

The link between “BMNH A100” and *Heterodontosaurus*, however, is supported by two synapomorphies that have broader distribution among heterodontosaurids. Increase in size of distal premaxillary teeth (Butler et al. 2008: character 114), is present to some degree in both *Echinodon* (Fig. 19B) and *Abrictosaurus* (Figs 31, 35). “Heterodont dentary dentition” (Butler et al. 2008: character 124) is a poorly constructed character that refers in part to the presence of a dentary caniniform tooth in “BMNH A100” and *Heterodontosaurus*. *Echinodon*, *Lycorhinus* and *Abrictosaurus* were scored as lacking such “heterodonty”, although from the information in this study it is clear that *Echinodon* (Figs 16B, C, 19B) and *Lycorhinus angustidens* (Fig. 3) clearly have a dentary caniniform tooth and the corresponding dentary crown in *Abrictosaurus* (Figs 31, 32) is not truly primitive in form (i.e., labiolingually compressed, subtriangular).

Zheng et al. (2009) added *Tianyulong* to the dataset in Butler et al. (2008), which added one more genus to the heterodontosaurid polytomy. Norman et al. (2011: 59) also used the dataset in Butler et al. (2008), restricting their reanalysis to “diagnosable heterodontosaurids” and a total of 14 ingroups. Thus they excluded *Fruitadens*, *Lycorhinus*, and *Tianyulong*, leaving three heterodontosaurids in the analysis (*Abrictosaurus*, “BMNH A100”, *Heterodontosaurus*). Like previous hypotheses (Sereno 1986; Butler et al. 2008), their resolution positioned “BMNH A100” closer to *Heterodontosaurus* than *Abrictosaurus* (Norman et al. 2011: fig. 41; Fig. 103E) on the basis of the weak evidence cited above.

Butler et al. (2010) added six characters (for a total of 227) and two heterodontosaurids (*Tianyulong*, *Fruitadens*) to the previous dataset (Butler et al. (2008). They corrected character state scores for *Echinodon* and ordered a subset of the multistate characters (112, 135, 137, 138, 174). Their reanalysis yielded an unresolved basal polytomy of heterodontosaurid genera including *Echinodon*. The more resolved cladogram in the main text of the paper (Butler et al. 2010: fig. 4) was obtained by removing three of the seven heterodontosaurids in the data matrix (*Abrictosaurus, Lycorhinus*, *Tianyulong*). This more resolved tree unites “BMNH A100” and *Heterodontosaurus*, with *Fruitadens* and *Echinodon* positioned as more remote sister taxa and *Pisanosaurus* positioned outside all other ornithischians (Fig. 103D).

Reanalysis of 224 of these characters (3 are uninformative) confirms their result in a strict consensus tree (summarizing 2246 minimum-length trees of 520 steps), although the supporting character evidence among heterodontosaurids was not discussed and is problematic. Ordering of one character (premaxillary tooth count; Butler et al. 2010: character 112) is responsible for maintaining *Echinodon* among heterodontosaurids. One unambiguous synapomorphy (“heterodont dentary dentition”; Butler et al. 2010: character 124) unites other heterodontosaurids to the exclusion of *Echinodon*, but this character remains incorrectly scored in *Echinodon*. *Fruitadens* is positioned outside two other heterodontosaurids (“BMNH A100”, *Heterodontosaurus*) on the basis of three problematic synapomorphies (Butler et al. 2010: characters 114, 126, 222). The first involves increasing crown size within the premaxillary tooth row, but this synapomorphy depends on the identification of a fragmentary bone in *Fruitadens* as a premaxilla, which is questioned in this study. The second concerns the absence of replacement foramina, which are not present in either *Echinodon* or *Fruitadens* (Butler et al. 2010) and are transiently present in some specimens of *Heterodontosaurus* (Norman et al. 2011). The third involves the “systematic development of wear facets”, which is subject to a number of factors (growth, replacement) and here is considered problematic as character. The evidence, in sum, for heterodontosaurid interrelationships is extremely weak and problematic.

Pol et al. (2011) added the newly discovered heterodontosaurid *Manidens* to the dataset of Butler et al. (2010). They also added three characters (characters 228-230), adjusted several character descriptions, and ordered two multistate characters (107, 118; these were altered from binary to three-state characters). Unlike Butler et al. (2010), however, they did not order five of the multistate characters, and as a result *Echinodon* was excluded from the heterodontosaurid clade (Figs 103D, 104). Using an alternative parsimony analysis package (TNT), the reported 216 minimum-length trees of 551 steps, the consensus of which positions *Manidens*, *Fruitadens*, and *Tianyulong* as successive outgroups to southern African heterodontosaurids (Fig. 104).

Reanalysis of 227 of these characters (3 are uninformative) confirms their result (1629 minimum-length trees of 551 steps), although the issues cited above regarding character evidence among heterodontosaurids remain. The single unambiguous synapomorphy uniting African heterodontosaurids to the exclusion of *Manidens*, *Fruitadens* and *Tianyulong* is “systematic development of wear facets”. For this character, *Abrictosaurus* is scored as having such facets, although to my knowledge tooth wear of any kind in this taxon is described for the first time in this study.

**Heterodontosaurid phylogeny, present study.** Heterodontosaurid interrelationships are poorly resolved for two principal reasons: (1) only a few characters are informative for heterodontosaurid interrelationships in the large datasets discussed above and (2) only a subset of these can be scored in most heterodontosaurids. As a result, resolution among heterodontosaurids is weakly supported and possible only when eliminating poorly represented “wild card” species. In the present analysis of heterodontosaurid interrelationships, outgroup relationships are constrained so that character evidence is focused only on potential heterodontosaurid synapomorphies and features that vary within Heterodontosauridae. Of the 30 characters considered, 14 (47%) are new to an analysis of heterodontosaurids or basal ornithischians and 17 (57%) vary within Heterodontosauridae (Appendix I). Details regarding these characters and the distribution of character states are given in Appendix I.

**Figure 105.**
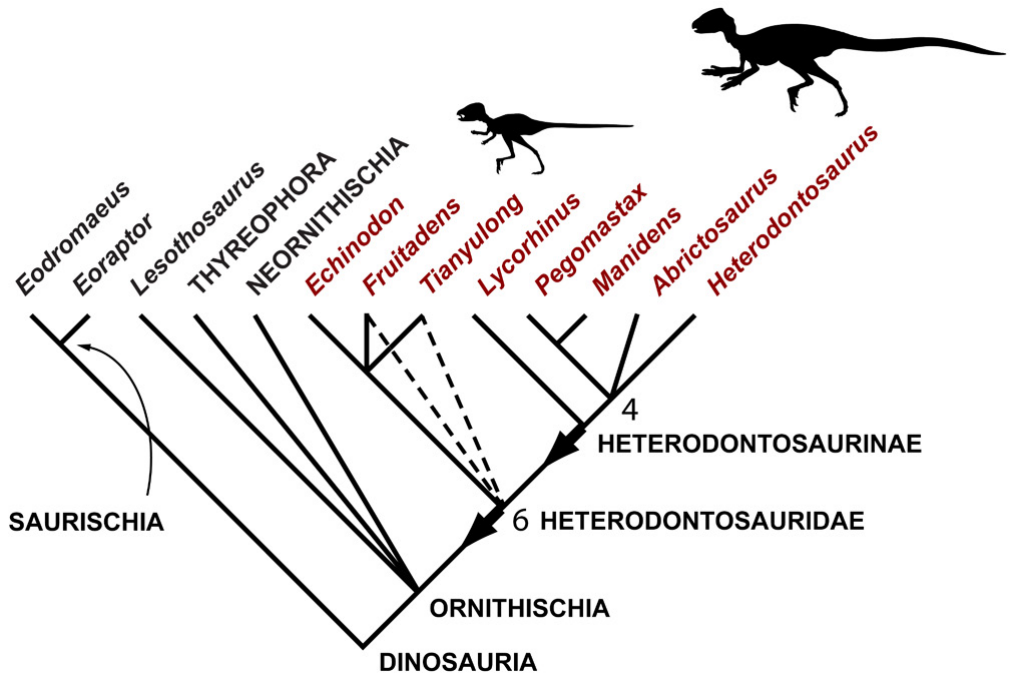
Phylogenetic hypothesis for heterodontosaurids based on character data assembled in this study. Consensus tree summarizing 6 minimum-length trees (38 steps) based on maximum parsimony analysis of 30 characters in 8 heterodontosaurid genera (consistency index = 0.79; retention index = 0.88; Appendix I). Outgroups (constrained as shown) include basal saurischians (*Eoraptor*, *Eodromaeus*) and representative ornithischians (*Lesothosaurus*, Thyreophora, Neornithischia). Dashed lines indicate loss of resolution with an increase of one step in tree length; numbers at nodes indicate the decay index when four poorly known heterodontosaurids (*Echinodon*, *Fruitadens*, *Lycorhinus*, *Pegomastax*) are removed from the analysis. Red text highlights heterodontosaurid genera; arrows indicate stem-based definitions for Heterodontosauridae and Heterodontosaurinae; body icons of *Tianyulong* and *Heterodontosaurus* are shown at appropriate relative size (based on Figures 30 and 72).

All heterodontosaurid genera (eight) are included except the problematic taxon *Pisanosaurus mertii*, inclusion of which results in loss of resolution. All characters are analyzed as unordered, except for the presence of a peg-shaped tooth at the anterior end of the dentary tooth row in some heterodontosaurids (character 3). This reduced tooth is ordered to allow loss only (“Dollo-up”; Swofford 1998), as it appears to represent a rudimentary nonfunctional tooth anterior to the caniniform tooth that is probably subject to repeated elimination. Character polarity is established by five proximate outgroups with a constrained outgroup configuration (Fig. 105; Appendix I). Two suprageneric outgroups are scored on representative basal species: Thyreophora is based on *Scutellosaurus lawleri* (Colbert 1981), *Emausaurus ernsti* (Haubold 1990) and *Scelidosaurus harrisonii* (Owen 1861; Norman 2001); Neornithischia is based on *Hypsilophodon foxii* (Galton 1974a) and *Yandusaurus hongheensis* (He and Cai 1984). *Lesothosaurus* and basal saurischian outgroups, *Eoraptor lunensis* and *Eodromaeus murphi*, are scored from study of original materials (Sereno 1991; Martinez et al. 2011).

Maximum parsimony analysis yields six minimum-length trees (38 steps) with relatively high consistency and retention indices (Fig. 105). The minimum-length trees vary in resolution of relationships among low-crowned Laurasian heterodontosaurids (*Echinodon*, *Fruitadens*, *Tianyulong*) and among advanced heterodontosaurines (*Manidens*, *Pegomastax*, *Abrictosaurus*, *Heterodontosaurus*). Heterodontosaurid monophyly inclusive of *Echinodon* is supported by six unambiguous synapomorphies (characters 1, 2, 4, 21, 24, 29). Other clades with moderately strong support (four unambiguous synapomorphies) include Gondwanan heterodontosaurids (here termed Heterodontosaurinae) and Gondwanan heterodontosaurids exclusive of *Lycorhinus* (Fig. 105).

Considering trees one step longer than minimum length (39 steps, 12 trees), resolution of a Laurasian heterodontosaurid clade breaks down (Fig. 105, dashed lines). When considering trees two steps longer than minimum length (40 steps), all resolution among heterodontosaurids breaks down. The heterodontosaurids responsible for complete loss of resolution include *Echinodon*, *Fruitadens*, *Lycorhinus* and *Pegomastax*, genera with positive scores for less than 60% of the characters. Inclusion of any combination of these genera results in loss of resolution among heterodontosaurids in trees two steps longer than minimum length. Removal of these four genera yields a single tree (34 steps) with *Tianyulong* as the sister group to Gondwanan heterodontosaurids, with *Manidens* as the sister group to the African heterodontosaurines *Abrictosaurus* + *Heterodontosaurus*. The outgroup position of *Tianyulong* relative to this subset of Gondwanan heterodontosaurids (*Manidens*, *Abrictosaurus*, *Heterodontosaurus*) is stable in trees three steps longer than minimum length (37 steps).

The present dataset, therefore, strongly supports (1) the monophyly of heterodontosaurids and (2) the basal position of *Tianyulong* outside Gondwanan heterodontosaurids (=Heterodontosaurinae). Weaker support is present for (3) a split between Laurasian and Gondwanan heterodontosaurids, (4) an outgroup position for *Lycorhinus* among Gondwanan heterodontosaurids, and (5) a close relationship between the South American and southern African heterodontosaurines *Manidens* and *Pegomastax*, respectively. Contrary to previous analyses (Butler et al. 2008, 2009; Norman et al. 2011; Fig. 103C-E), there is no support for positioning *Lycorhinus* (either the holotype [SAM-PK-K3606] or NHMUK RU A100 [formerly “BMNH A100”]) as the sister taxon to *Heterodontosaurus* among African heterodontosaurids.

**Figure 106.**
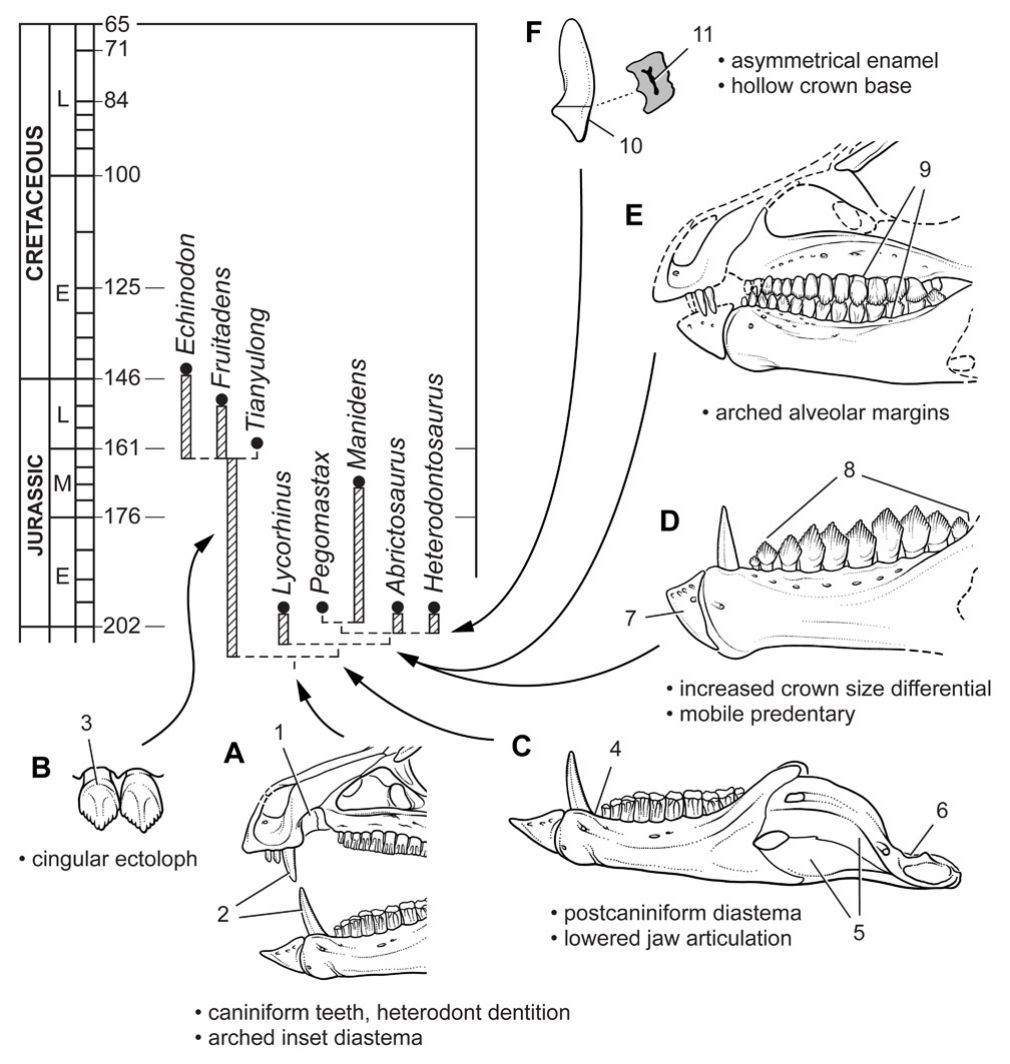
Evolution of key masticatory specializations among heterodontosaurids. **A** Heterodontosauridae **B** Simple-crowned heterodontosaurids **C** Heterodontosaurinae **D** Advanced heterodontosaurines **E** *Abrictosaurus* and *Heterodontosaurus* **F** *Heterodontosaurus*. Abbreviations: ***1*** arched inset diastema ***2*** heterodont dentition with caniniform teeth ***3*** cingular ectoloph ***4*** postcaniniform diastema ***5*** external mandibular fossa ***6*** lowered jaw articulation ***7*** mobile predentary articulating against saddle-shaped dentary articular surface ***8*** increased differential in crown size ***9*** arched alveolar margins in cheek dentition ***10*** asymmetrical enamel ***11*** hollow crown base.

The analysis allows a more detailed understanding of heterodontosaurid evolution (Fig. 106). All heterodontosaurids appear to share their most outstanding features—a heterodont dentition characterized by caniniform premaxillary and dentary teeth, the latter sequestered in an inset arched diastema formed by the premaxilla and maxilla (Fig. 106, features 1, 2). By the earliest Jurassic, heterodontosaurids appear to have split along two lines, one maintaining a low-crowned dentition and the other evolving crowns with deeper proportions. At present there is little information uniting the four known low-crowned heterodontosaurids (*Echinodon*, *Fruitadens*, *Tianyulong*, Kayenta heterodontosaurid). Their cheek teeth are characterized by a cingular ectoloph (Fig. 106, feature 4), but this feature is only weakly expressed in *Echinodon* (Appendix I: character 3). Three of these low-crowned heterodontosaurids (*Echinodon*, *Fruitadens*, Kayenta heterodontosaurid) share a deeply impressed vascular groove leading from the anterior dentary foramen (Appendix I: character 25), but the condition is unknown in *Tianyulong*. Three of the low-crowned heterodontosaurids (*Echinodon*, *Fruitadens*, *Tianyulong*) are younger in age than heterodontosaurines, implying one or more missing lineages as long as 50 million years in length (Fig. 106).

Heterodontosaurines are characterized by a postcaniniform diastema, lowered jaw articulation, an enlarged, inset surangular foramen, and an external mandibular fossa (Appendix I: characters 5, 26-28; Fig. 106, features 4-6). These features are not preserved in all heterodontosaurines. Better documentation of the relative position of the jaw articulation is needed in *Lycorhinus* and *Abrictosaurus*. Furthermore, in *Abrictosaurus* the postcaniniform diastema is not present, which may have been reversed as a part of the reduction of the caniniform complex.

*Lycorhinus* is positioned as the sister group to remaining heterodontosaurines, a subclade characterized by loss of the cingulum and increased crown size differential in the cheek teeth and a particularly mobile predentary articulating against the expanded end of the dentary (Appendix I: characters 5, 23, 26-28; Fig. 106, features 7, 8). Among these more advanced heterodontosaurines, *Manidens* and *Pegomastax* appear to be most closely related based on the asymmetrical shape of the crowns of their cheek teeth in lateral view (distally curved). *Abrictosaurus* and *Heterodontosaurus* have arched alveolar margins, their cheek teeth in occlusion exhibiting a characteristic oval shape in lateral view (Appendix I: character 12; Fig. 106, feature 9). Homoplasy in other characters, however, creates ambiguity over their relationship as sister taxa (Fig. 105). Finally, in several regards *Heterodontosaurus* remains the most derived heterodontosaurine. The crowns of the cheek teeth have particularly thin enamel on the side opposite the cutting edge (Fig. 106, feature 10), an asymmetrical enamel pattern that arose independently in euornithopod and neoceratopsian ornithischians (Sereno 1997). The cavity normally restricted to the root in ornithischians (Fig. 53, 54), furthermore, extends into the base of the crown in the cheek teeth (Fig. 106, feature 11). The presence or absence of these specialized dental features among other heterodontosaurines remains poorly known.

All of the derived features that evolved in heterodontosaurines are plausibly interpreted as trophic specializations for procuring and more efficiently processing plant materials (Fig. 106, features 4-11). The sophistication of these specializations, known in their entirety only in *Heterodontosaurus*, is remarkable for such an early ornithischian radiation. Together they imply that all heterodontosaurines had enhanced the size and/or differentiation of adductor musculature (Fig. 106, feature 5), dropped the jaw articulation to enhance simultaneous occlusion of the cheek teeth (Fig. 106, feature 6), and increased tooth wear to create broad, truncating wear facets on cheek tooth crowns.

### Biogeographic inferences

**Figure 107.**
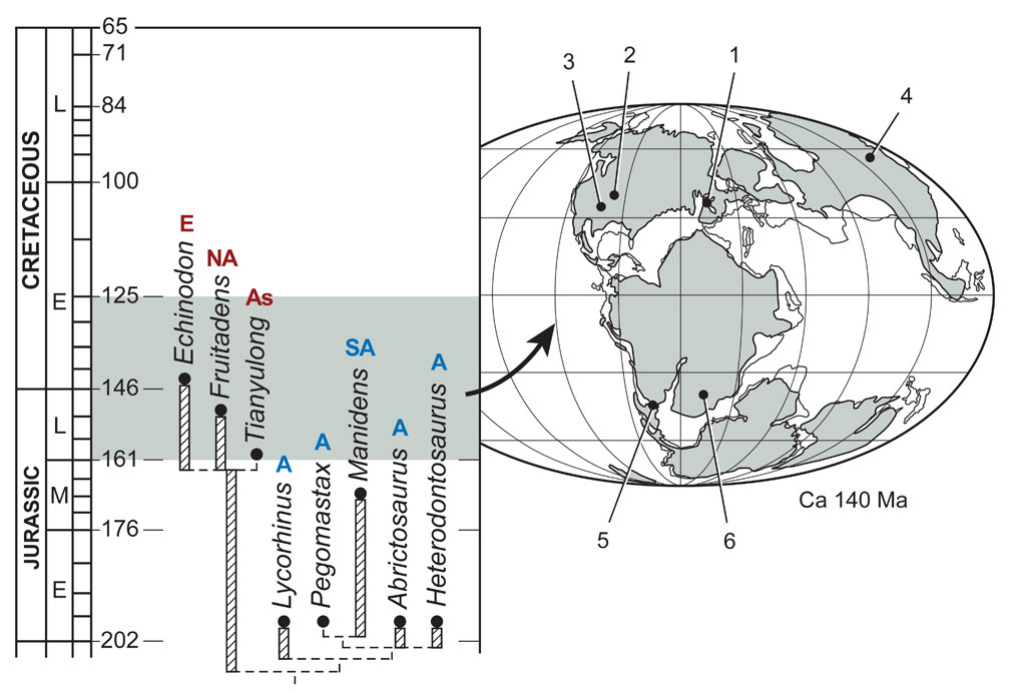
Biogeographic distribution in time for heterodontosaurids included in the phylogenetic analysis. Localities for included taxa are plotted on an Early Cretaceous (Berriasian-Valanginian) paleogeographic map (Smith et al. 1994) and recent time scale (Gradstein and Ogg 2009). Abbreviations for taxon localities and biogeographic areas: **1** *Echinodon* **2** *Fruitadens* **3** Kayenta heterodontosaurid **4** *Tianyulong* **5** *Manidens* **6** *Abrictosaurus*, *Lycorhinus*, *Heterodontosaurus*, *Pegomastax* ***A*** Africa ***As*** Asia ***E*** Europe ***NA*** North America ***SA*** South America.

Phylogenetic analysis provides weak support for an initial split among heterodontosaurids prior to the earliest Jurassic into low-crowned Laurasian and deeper-crowned Gondwanan subclades (Fig. 107). Even with *Tianyulong* positioned as Late Jurassic in age (Liu et al. 2012), all described heterodontosaurids from Laurasia are from horizons younger in age than those yielding heterodontosaurids on Gondwanan landmasses (Fig. 107, grey tone). The Early Jurassic heterodontosaurid from the Kayenta Formation of northern Arizona will begin fill in the earlier history of low-crowned heterodontosaurids (Attridge et al. 1985; Sereno 1986, 1997; MCZ 9092; Fig. 9B). As heterodontosaurids are among the smallest known ornithischians, their temporal and geographic ranges will likely always remain poorly established.

The Gondwanan subclade, Heterodontosaurinae, has better phylogenetic support (Figs 105, 107). This southern group was recognized previously when *Manidens* was described, the first undoubted South American heterodontosaurid (Pol et al. 2011). As these authors noted, the Middle Jurassic age of *Manidens* indicates that heterodontosaurines with deeper crown proportions survived as small-bodied herbivores through most of the Jurassic, or for a duration of at least 35 million years (Fig. 107).

## Conclusions

Review of available heterodontosaurid fossils unequivocally establishes *Echinodon becklesii* as a very small-bodied, late-surviving northern heterodontosaurid similar to the other northern heterodontosaurids with simple subtriangular cheek tooth crowns. One of these low-crowned species from northern China, *Tianyulong confuciusi*, is shown to have unusual skeletal proportions, including a relatively large skull, short forelimb and long manual digit II. The southern African heterodontosaurid *Lycorhinus angustidens* is established as a valid genus and species, to which several specimens are referred including a partial skull (NHMUK RU A100). A new taxon, *Pegomastax africanus*, is named and described from contemporary Early Jurassic horizons in southern Africa. *Manidens condorensis* from South America and all African heterodontosaurids are placed in the subclade Heterodontosaurinae.

Tooth replacement and tooth-to-tooth wear is more common than previously thought among heterodontosaurids. In *Heterodontosaurus tucki* the angle of tooth-to-tooth shear is shown to increase markedly during maturation. Based on evidence from mesowear and the angle and snug fit of the jaw articulation, long-axis rotation of the lower jaw during occlusion is identified in *Heterodontosaurus tucki* as the most likely primary functional mechanism underlying marked tooth wear in mature specimens. Predominant or exclusive herbivory is regarded as the most probable dietary inference for all heterodontosaurids, based primarily on the sequestering of the dentary caniniform tooth during occlusion, details of mesowear on the crowns of caniniform and cheek teeth, and the presence of many features that enhance the strength of the dentary and adductor musculature.

Heterodontosaurines evolved remarkably sophisticated masticatory adaptations comparable in complexity to those of euornithopod neornithischians of the Late Jurassic. Comprising the most diverse early radiation of ornithischians, heterodontosaurids ranged across both Laurasia and Gondwana, persisting on various landmasses into the Early Cretaceous for a total duration of at least 100 My.

## Supplementary Material

XML Treatment for Heterodontosauridae

XML Treatment for Echinodon becklesii

XML Treatment for Fruitadens haagarorum

XML Treatment for Tianyulong confuciusi

XML Treatment for Heterodontosaurinae

XML Treatment for Abrictosaurus consors

XML Treatment for Heterodontosaurus tucki

XML Treatment for Lycorhinus angustidens

XML Treatment for Manidens condorensis

XML Treatment for Pegomastax

XML Treatment for Pegomastax africanus

## Appendix I

### Character list

The character statements listed below vary among heterodontosaurids and are informative for relationships within Heterodontosauridae. All are unordered except character 1, which is constrained by Dollo parsimony as explained below. The dataset was analyzed using PAUP* 4.0 (beta v. 10; Swofford 1998) configured for maximum parsimony using a constraint tree for the configuration for outgroups. Authors are cited for first use of a character in analyses of basal ornithischian dinosaurs.

### Dental

**1. Premaxillary teeth, number (modified from Butler [2005: character 1])**

0 four or more

1 three or fewer

*Description*: Most heterodontosaurids in which the premaxillary series is known have three teeth inset distally from the anterior end of the premaxilla (Figs 19B, 35, 59). *Tianyulong* appears to have only two premaxillary teeth (Fig. 23). The bone identified as a premaxilla in *Fruitadens* may be correct but is unusual in several regards (Fig. 9A), so the number and size of the premaxillary crowns in this genus are regarded as unknown. At least four premaxillary teeth are present in other basal ornithischians and basal saurischian outgroups (Martinez et al. 2011).

**2. Premaxillary teeth, relative crown size (modified from Butler et al. [2008: character 114])**

0 subequal with largest within series

1 increase distally

*Description*: In all heterodontosaurids the premaxillary teeth increase in size distally. The last premaxillary tooth is much larger and caniniform in form in all heterodontosaurids except *Echinodon* and *Abrictosaurus* (Figs 19B, 35, 59, 80). The premaxilla may be misidentified in *Fruitadens* (Fig. 9A), and so the number and size of the premaxillary crowns in this genus are regarded as unknown pending further information. In ornithischians retaining the premaxillary series, the crowns are usually subequal in size (e.g., *Lesothosaurus*; Sereno 1991). In some outgroups such as the basal sauropodomorph *Eoraptor*, the largest crown is in a penultimate position. In the basal theropod *Eodromaeus*, the relative sizes of the crowns are not well known (Martinez et al. 2011).

**3. First (precaniniform) dentary tooth small, peg-shaped (modified from Butler et al. [2008: character 125])**

0 present

1 absent

*Description*: In some heterodontosaurids, a small peg-shaped tooth is present at the mesial end of the dentary tooth row (*Echinodon*, *Fruitadens*, *Lycorhinus*, *Abrictosaurus*). In *Echinodon*, only the small alveolus is preserved (Figs 16B, C, 19B). In *Abrictosaurus*, the two teeth at the anterior end of the dentary are here identified as the small peg-shaped tooth and an enlarged second dentary tooth (albeit relatively small compared to other heterodontosaurids) (Figs 32, 35). Based on these heterodontosaurids, it seems that the second dentary tooth was enlarged as a caniniform at the expense of the first dentary tooth, which was subsequently reduced and eliminated. In this interpretation, the caniniform dentary tooth is considered homologous, whether or not it is preceded by a peg-shaped tooth, and the peg-shaped tooth is thought to have been lost independently in heterodontosaurids with primitive (e.g., *Tianyulong*, Kayenta heterodontosaurid) and more derived dentitions (e.g., *Pegomastax*, *Heterodontosaurus*). Among heterodontosaurid outgroups and especially basal saurischians, comparison to anterior dentary teeth is ambiguous, especially for basal saurischians. This character, thus, is constrained by “Dollo-up” character state order in the analysis, which only allows multiple losses.

**4. Dentary caniniform tooth and arched premaxilla-maxilla diastema (modified from Butler [2005: character 5] and Butler et al. [2008: characters 15, 124])**

0 present

1 absent

*Description*: Heterodontosaurids have a caniniform tooth in the first or second tooth position at the anterior end of the dentary. In occlusion the tip of the caniniform tooth rests within an inset, arched diastema formed by the premaxilla and maxilla (Figs 9, 19B, 23, 59, 80, 81B, 93A). In *Abrictosaurus* the second dentary tooth is small but has a subconical crown that projects above adjacent crowns and may represent a reduced caniniform tooth (Figs 31, 35). If an arched diastema were present in *Abrictosaurus*, it could not have been as large as that in other heterodontosaurids. The dentary caniniform tooth and accommodating arched diastema are regarded here as correlated within heterodontosaurids, as they are functionally interrelated and have not been shown to differ in distribution.

**5. Diastema distal to dentary caniniform tooth (new)**

0 absent

1 present

*Description*: In some heterodontosaurids, a space approximately the width of one crown is present between the caniniform tooth and the successive tooth in the dentary tooth row (*Lycorhinus*, *Pegomastax*, *Heterodontosaurus*). *Abrictosaurus* does not exhibit this diastema, although in this case the caniniform tooth is very reduced in size (Fig. 35).

**6. Cheek teeth, number (modified from Butler [2005: character 8])**

0 14 or more

1 12 or less

*Description*: Some heterodontosaurids appear to have limited the number of teeth in the tooth row to 12 or less (*Echinodon*, *Fruitadens*, *Manidens*, *Pegomastax*, *Heterodontosaurus*). Tooth number does not appear to be strictly size related, as *Abrictosaurus* and other small bodied ornithischians (e.g., *Lesothosaurus*) have more teeth in either the dentary or maxillary tooth rows. Lycorhinus is somewhat larger and also has more than 12 cheek teeth (Figs 35, 80).

**7. Cheek teeth, differential crown size along tooth row (new)**

0 subequal

1 central crowns more than twice as large as mesial and distal crowns

*Description*: Some heterodontosaurids have substantially larger crowns in the center of both maxillary and dentary tooth rows (*Manidens*, *Pegomastax*, *Abrictosaurus*, *Heterodontosaurus*). In other heterodontosaurids, crown size is more uniform along the tooth row, diminishing only in teeth at the extreme end of the tooth row (e.g., *Echinodon*, *Tianyulong*, *Fruitadens*).

**8. Cheek teeth, crown height relative to mesiodistal crown width (modified from Pol et al. [2011: character 228])**

0 less than 1.5

1 greater than or equal to 1.5

*Description*: In some heterodontosaurids, crown proportions are substantially taller (*Lycorhinus*, *Manidens*, *Pegomastax*, *Abrictosaurus*, *Heterodontosaurus*). Crown proportions, in this case, are based on the largest teeth near the center of maxillary and dentary tooth rows, as seen in labial view.

**9. Cheek teeth, curvature of crown axis (labial view) (new)**

0 absent

1 mesially bowed

*Description*: In *Manidens* and *Pegomastax*,the central axis of crowns of the cheek teeth appears to curve distally, as seen in labial view. In other heterodontosaurids, no such curvature is apparent. The crown as a whole, however, is not recurved as in some basal dinosaurs that probably have an omnivorous or herbivorous diet such as *Eoraptor* (Martinez et al. 2011).

**10. Cheek teeth, cingulum separating crown from root, development (modified from Butler et al. [2007: characters 78, 83])**

0 marked expansion of crown base

1 reduced, weak expansion of crown base

*Description*: The crown is poorly differentiated from the root transversely and mesiodistally in several heterodontosaurids (*Manidens*, *Pegomastax*, *Abrictosaurus*, *Heterodontosaurus*), which differs from the primitive condition exhibited in other heterodontosaurids (e.g., *Lycorhinus*, *Echinodon*, *Tianyulong*, *Fruitadens*).

**11. Cheek teeth, cingular ectoloph separating cingulum from the remainder of the labial crown surface (new)**

0 absent

1 present

*Description*: In some heterodontosaurids, a distinctive ridge (= loph) is present labially near the base of crown base. If there is a cingulum, it forms a discrete raised belt around the crown and has a well-defined dorsal edge differentiated from the remainder of the labial crown face. This cingular ectoloph is well developed in *Tianyulong* and *Fruitadens* and poorly developed in *Echinodon* (scored as polymorphic). This structure is not present in other heterodontosaurids.

**12. Alveolar margin in cheek, dorsoventral curvature (new)**

0 straight

1 bowed dorsally (maxilla) and ventrally (dentary)

*Description*: In *Abrictosaurus* and *Heterodontosaurus*, the alveolar margin of the maxilla and dentary is gently arched, as seen in lateral view. The opposing dorsal and ventral arching of the alveolar margin seems to accommodate larger crowns in the center of each tooth row, which meet along a nearly straight line of occlusion similar to adjacent smaller crowns. In other heterodontosaurids, the alveolar margin appears to be relatively straight.

### Cranial

**13. Nasal fossa, dorsomedian, subtriangular with rounded lateral rims (****)**

0 absent

1 present

*Description*: A median fossa is present on the dorsal aspect of the nasals posterior to the internarial bar. Rounded raised edges of the nasals bound the fossa to each side. The fossa comes to a median pit anteriorly and dissipates on to the dorsal surface of the snout distally. The function, if any, of this fossa remains unknown; that it may have housed a display structure is entirely hypothetical (Fig. 101). Among heterodontosaurids, the fossa is currently known in *Heterodontosaurus*, *Tianyulong*, and the Kayenta heterodontosaurid. A nasal fossa of similar form and location is present in the basal ornithischian *Agilisaurus* (Peng 1992) and to a lesser extent in the basal sauropodomorph *Eoraptor* (Martinez et al. 2011), although most basal dinosaurs do not have a marked depression in this location.

**14. Jugal flange (projecting from posterior ramus) (new)**

0 absent

1 present

*Description*: In *Manidens* and *Heterodontosaurus*, a tongue-shaped, transversely compressed flange projects posteriorly or posteroventrally from the ventral margin of the posterior ramus of the jugal. The flange is present and fully developed in subadult *Heterodontosaurus* (Fig. 40), and thus does not appear to be a display feature associated with sexual maturation. Its presence in more than a single heterodontosaurid suggests a common function. The location of the flange near an embayment of the infratemporal bar and immediately lateral to the capacious adductor chamber suggests it may have served as the origin of specialized jaw-closing musculature (Figs 95, 96). Unfortunately, this portion of the jugal is not known in other heterodontosaurids.

**15. Jugal horn (laterally directed, dorsoventrally compressed) ventral to orbit (modified from Butler [2005: character 20] and Butler et al. [2007: character 24])**

0 absent

1 present

*Description*: In *Manidens* and *Heterodontosaurus*, a wedge-shaped, dorsoventrally compressed rugose horn projects laterally from just below the orbital margin of the jugal (Figs 59, 81B). The anterior margin of the horn joins the everted edge of the maxilla dorsal to the buccal emargination. Unfortunately, this portion of the jugal is not known in other heterodontosaurids.

**16. Postorbital central body, form (lateral view) (modified from Pol et al. [2011: character 229])**

0 flat

1 arcuate fossa with raised anterior rim

*Description*: In *Manidens* and *Heterodontosaurus*, an arcuate depression is present on the lateral aspect of the central body of the postorbital, bounded anteriorly by a well-defined edge. The resulting hollow borders the laterotemporal fossa and presumably served as a site for jaw-closing musculature (Figs 95, 96). Unfortunately, the postorbital is exposed only in medial view in *Pegomastax* and is not known in other heterodontosaurids.

**17. Postorbital posterior ramus, dorsoventral width (lateral view) (new)**

0 narrow, basal width approximately 1/3 length

1 broad, basal width approximately 1/2 length

*Description*: In *Manidens* and *Pegomastax*, the posterior ramus of the postorbital is very broad and tongue-shaped rather than prong-shaped as in *Heterodontosaurus* (Figs 59, 81B, 82). Unfortunately, the postorbital is not known in other heterodontosaurids.

**18. Quadrate head, relation to fossa in dorsolateral corner of laterotemporal fenestra (new)**

0 excluded

1 included

*Description*: In *Manidens* and *Heterodontosaurus*, the head of the quadrate is inset into the laterotemporal fenestra and presumably contributes to the attachment area for jaw-closing musculature (Figs 95, 96). Unfortunately, this portion of the jugal is not known in other heterodontosaurids.

**19. Quadrate condyle, inclination of ventral articular surface (anterior view) (new)**

0 horizontal

1 ventrolaterally inclined at approximately 30°

*Description*: Some heterodontosaurids appear to have limited the number of teeth in the tooth row to 12 or less (*Echinodon*, *Fruitadens*, *Manidens*, *Pegomastax*, *Heterodontosaurus*), a feature that does not appear to be size related.

**20. Quadratojugal, shape (lateral view) (new)**

0 L-shaped

1 T-shaped

*Description*: In *Manidens* and *Heterodontosaurus*, the T-shaped quadratojugal contributes to an embayment ventral to the infratemporal bar Figs 59, 81B). Unfortunately, the shape of the quadratojugal is poorly known in *Abrictosaurus* (Fig. 35), and the bone has not been described in other heterodontosaurids.

**21. Predentary processes (modified from Butler et al. [2007: character 55])**

0 present

1 rudimentary or absent

*Description*: The wedge-shaped predentary, which is known in most heterodontosaurids, has rudimentary lateral and ventral processes. In *Echinodon* a predentary of similar form is inferred from the anterior end of the dentary.

**22. Dentary articular surface for predentary, form (new)**

1 flat

2 saddle-shaped

*Description*: In *Heterodontosaurus*, *Abrictosaurus* and *Pegomastax*, the saddle-shaped form of the articular surface for the predentary is distinctive (Figs 35, 59, 87). The anterior end of the dentary has a planar or gently convex surface in *Echinodon*, *Fruitadens* and the Kayenta heterodontosaurid. A similar condition is probably present in *Lycorhinus*, but the anterior end of the dentary is currently visible only in medial view (Fig. 78).

**23. Dentary anterior end, form (lateral view) (new)**

0 tapered, subtriangular

1 dorsoventrally expanded, rounded

*Description*: In *Heterodontosaurus*, *Abrictosaurus* and *Pegomastax*, the dorsoventrally expanded shape of the anterior end of the dentary is distinctive (Figs 35, 59, 87). The anterior end of the dentary is not expanded in *Echinodon*, *Tianyulong*, *Fruitadens*, *Lycorhinus* and the Kayenta heterodontosaurid.

**24. Dentary depth at mid-length relative to length (new)**

0 less than 25%

1 25% or more

*Description*: The height of the dentary at mid-length is 25% or more of its length in heterodontosaurids. Dentary length in this case is measured from the anterior extremity to the notch formed by the external mandibular fenestra or an equivalent sutural junction (Figs 19B, 35, 59, 80, 81B). The dentary has more slender proportions in other ornithischians, including those of comparable body size such as *Lesothosaurus* (Sereno 1991).

**25. Vascular tract from anterior dentary foramen, form (new)**

1 shallow groove

2 impressed canal

*Description*: In *Echinodon* and *Fruitadens*, the vascular tract extending from the anterior dentary foramen toward the predentary is sharply inset into the dentary (Figs 9A, 17). In several other heterodontosaurids, only a shallow groove is present anterior to the foramen.

**26. Anterior surangular foramen, size/form (new)**

0 small or absent, shallow groove on anterior side

1 large, impressed channel on posterior side

*Description*: The anterior surangular foramen in some heterodontosaurids is enlarged with an impressed groove on its posterior side (*Lycorhinus*, *Manidens*, *Heterodontosaurus*) (Figs 59, 79, 81B), which appears to be absent in *Tianyulong* (Fig. 9C). Among outgroups, the anterior surangular foramen, when present, is not enlarged and typically has a groove on its anterior side (Sereno, 1991; Martinez et al. 2011).

**27. External mandibular fossa (new)**

0 absent

1 present

*Description*: There is a distinctive depression, or fossa, centered around the external mandibular foramen in several heterodontosaurids (*Lycorhinus*, *Manidens*, *Heterodontosaurus*) (Figs 59, 79, 81B), which appears to be absent in *Tianyulong* (Fig. 9C). This fossa is not known among outgroups and may be related to the elaboration of jaw muscle attachments in derived heterodontosaurids (Figs 95, 96).

**28. Jaw articulation, percent drop (measured from axis at mid-crown height) compared to the length from the jaw joint to the midpoint of the cheek tooth row (modified from Butler et al. [2008: character 109])**

0 less than 10%

1 more than 10%

*Description*: The jaw articulation is noticeably dropped below the level of occlusion between the cheek teeth in *Manidens* and *Heterodontosaurus* (Figs 59, 81B) as occurs in euornithopod ornithischians (Galton 1974a). There is some evidence for a lowered jaw articulation in both *Lycorhinus* (NHMUK RU A100) and *Abrictosaurus*, although this region of the skull is not as well preserved and exposed in available specimens. In *Tianyulong*, in contrast, the jaw joint is not lowered (Fig. 9C), and so this feature probably arose independently within Heterodontosauridae.

**29. Iliac postacetabular process, proportions relative to length and to minimum depth of preacetabular process (new)**

0 minimum depth 40% or more of length, greater than minimum depth of preacetabular process

1 minimum depth 30% of length, subequal to minimum depth of preacetabular process

*Description*: The slender proportions of the postacetabular process of the ilium are unusual in *Abrictosaurus* and *Heterodontosaurus* (Figs 37A, 68) as compared to *Manidens* (Fig. 8B) and the Kayenta heterodontosaurid. In the former two genera, the postacetabular process is very narrow and subequal in minimum depth to the preacetabular process. In most ornithischians including *Lesothosaurus* (Sereno 1991) and *Hypsilophodon* (Galton 1974a), the postacetabular process is proportionately deeper relative to its length and to the depth of the preacetabular process.

**30. Fibular mid-shaft and distal end, transverse diameter relative to comparable tibial diameter (new)**

0 25% or more

1 less than 25%

*Description*: The distal shaft and end of the fibular are reduced in width in many heterodontosaurids (*Fruitadens*, *Tianyulong*, *Abrictosaurus*, *Heterodontosaurus*, Kayenta heterodontosaurid) (Figs 30, 37C, 69, 70). In *Pisanosaurus*, in contrast, the fibula is not reduced (Fig. 7A), and so this feature may have arisen within Heterodontosauridae pending further information on the affinities of *Pisanosaurus*.

**Character-state matrix:** X = too transformed to score; ? = not preserved; A = states 0 & 1

**Taxa**	**Character states**			**% completeness**
Eoraptor	00X0000101	0010000000	0000000000	100
Eodromaeus	0?X0010101	0000000000	00000?0010	93
Lesothosaurus	00X0000000	0000000000	0000000000	100
Neornithischia	00X0000000	0000000000	0000000000	100
Thyreophora	00X0000000	0000000000	0000000000	100
Echinodon	1101010000	A0????????	10011?????	57
Fruitadens	??01010000	10????????	??011????1	47
Tianyulong	11?10?0000	101???????	1001?000?1	63
Lycorhinus	1101100100	00????????	??01?111??	57
Manidens	???1?11111	00?1111111	1??1?1110?	70
Pegomastax	??11111111	00????1???	11110?????	53
Abrictosaurus	1101001101	01???????1	11110??111	70
Heterodontosaurus	1111111101	0111110111	1111011111	100

## Appendix II

Video of coronal cross sections. (doi: 10.3897/zookeys.226.2840.app1) File format: Apple QuickTime Movie (MOV).

**Explanation note:** Cutaway view showing coronal computed tomographic sections of a partial subadult skull of *Heterodontosaurus tucki* (AMNH 24000) that shows active tooth replacement and wear facet orientation in maxillary and dentary teeth.

## Appendix III

Video of sagittal cross sections. (doi: 10.3897/zookeys.226.2840.app2) File format: Apple QuickTime Movie (MOV).

**Explanation note:** Cutaway view showing sagittal computed tomographic sections of a partial subadult skull of *Heterodontosaurus tucki* (AMNH 24000) that shows active tooth replacement and wear facet orientation in maxillary and dentary teeth.

## Appendix IV

Video of horizontal cross sections. (doi: 10.3897/zookeys.226.2840.app3) File format: Apple QuickTime Movie (MOV).

**Explanation note:** Cutaway view showing horizontal computed tomographic sections of a partial subadult skull of *Heterodontosaurus tucki*i (AMNH 24000) that shows active tooth replacement and wear facet orientation in maxillary and dentary teeth.
